# Supplementary material for: Exploring effects of severe mental illnesses on marriages: A qualitative study from Karachi, Pakistan
Source: PLOS Glob Public Health. 2025 Dec 23;5(12):e0005652. doi: 10.1371/journal.pgph.0005652 (PMC12725543; doi:10.1371/journal.pgph.0005652)
Supplement: S1 Data — (ZIP) [file pgph.0005652.s001.zip › Transcriptions/Case 1 Transcripts/C1-1.docx]

**Case 1**

**C1-1**

The spouse did not allow the interview to be recorded. He was extremely hesitant in doing so. The patient’s name is Rohi-Saba. Bipolar Disorder.

**Interviewer:** How long has your spouse been mentally ill?

**Interviewee:** This episode is present since 15-20 days. However, the problem was happening since 4-5 months.

**Interviewer:** What were the symptoms that she showed?

**Interviewee:** She was extremely restless. And then we got her checked from a friend of mine. He gave her sukoon ki dawai. She did not sleep for too long.

**Interviewer:** all right and what were the kind of problems that were faced?

**Interviewee:** She has gotten in her head that he cannot now sleep without her pills

**Interviewer:** Are your parents aware of her illness?

**Interviewee:** Not really

**Interviewer:** Okay and who provides support to the patient?

**Interviewee:** The financial support is coming from my in-laws (her parents).

**Interviewer:** Does it get stressful?

**Interviewee:** Well no tension as yet because I do not believe in taking tension. Also, I feel it is my responsibility to take care of my wife.

**Interviewer:** Do you get frustrated or exhausted?

**Interviewee:** Not really

**Interviewer:** What additional responsibilities have you taken because of your spouse’s illness?

**Interviewee:** Well I take care of children.. We have two children. I give them breakfast. I feel that this is my problem and I need to support her

**Interviewer:** Hmm okay and does your support help the patient?

**Interviewee:** Yes, it does She acknowledges that I do support her

**Interviewer:** How often do you go and socialize?

**Interviewee:** Once in a week or 15 days

**Interviewer:** Do people question about her illness?

**Interviewee:** Yes they do. They say we should get rohani ilaaj and I will do that, along with the doctor. Of course, I also tell people that they should pray for her to get better. I don’t tell in detail about the illness

**Interviewer:** Have the family dynamics changed since the illness of your spouse?

**Interviewee:** Not really

**Interviewer:** Do your children know about the illness?

**Interviewee:** Yes but they are children so they do not actually get too involved

**Interviewer:** Who encouraged in seeking help?

**Interviewee:** sister in law told us to come and seek help from the doctor.

**Interviewer:** Do you feel that your relationship has changed since the onset of illness?

**Interviewee:** Not really, no.

**Interviewer:** Do you feel that your relationship with others has been impacted due to illness?

**Interviewee:** No

**Interviewer:** Has it led to any mental health problems of your own?

**Interviewee:** No it’s my responsibility so I do not stress myself out.

**Interviewer:** have you ever hit her?

**Interviewee:** No

**Interviewer:** How much time do you take out in your normal day to take care of your wife?

**Interviewee:** I have to give her medications. She is not too compliant

**Interviewer:** Do you feel that you do things that normally your wife should have done?

**Interviewee:** Not really. This is because she still takes care of the kids. In fact, her mother also helps out a lot.

**Interviewer:** What do you know about the mental illness of your spouse?

**Interviewee:** It’s depression

**Interviewer:** Do you feel that it is your spouse’s fault to have the illness?

**Interviewee:** No not really

**Interviewer:** DO you feel that you personally can help in fixing her or him?

**Interviewee:** Yes I can give her less stress and tell her not to take too much stress.

**Interviewer:** When should a couple think of divorce?

**Interviewee:** A couple should not think of divorce generally. If it is outside a person’s bardasht, then they should think of it. “bardasht ki quwat na ho. Eik ko thanda hona chahye. Dunu ko bardasht karna chahye”

**Interviewer:** Has divorce been suggested by family members?

**Interviewee:** Not at all. No one knows I am going to a psychiatrist. I do not want people to ask her what the problem is.

**Interviewer:** What do you think are the essential building blocks for raising a healthy family?

**Interviewee:** There should be “tawun and mohabbat”

**Interviewer:** is it present in your marriage?

**Interviewee:** Yes, it used to be. She used to help me out a lot. She is a tailor so she used to take care of her expenses herself initially

**Interviewer:** How do you see your future?

**Interviewee:** I am hopeful about the future. I do not give it much thought.

**Interviewer:** is the family more important or the marriage (relationship between two people)?

**Interviewee:** marriage is more important because family comes from that only.
