# Supplementary material for: Exploring effects of severe mental illnesses on marriages: A qualitative study from Karachi, Pakistan
Source: PLOS Glob Public Health. 2025 Dec 23;5(12):e0005652. doi: 10.1371/journal.pgph.0005652 (PMC12725543; doi:10.1371/journal.pgph.0005652)
Supplement: S1 Data — (ZIP) [file pgph.0005652.s001.zip › Transcriptions/Case 1 Transcripts/C1-10.docx]

**Case 1**

**Illness:** Bipolar Disorder

*fills out the demographic form* (some important details include the fact that this is her second marriage, and her first husband who was her first cousin passed away. Even her current husband is her cousin, and her ex in-laws had kicked her out after taking her children. She mentions that she was brought up in such a way that she takes things in her stride *aapne uper kuch haawi na karein.* She mentions that she is very upset about her husband’s condition, which led her to undergo a depressive phase. She also went to the doctor for her condition)

**Interviewer:** How long have you been married for?

**Interviewee:** 14 years

**Interviewer:** Inki beemari ko kitna arsa hogaya hai?

**Interviewee:** 2007 mein meiney inko observe kya tha. 2007 mein yeh alag alag rehna shuru kardya tha. Matlab yeh khud gaye thay doctor kay pass. Yeh larai kartay thay.

**Interviewer:** Aap ko inki beemari ka naam pata hai?

**Interviewee:** *pause* yeh mujhe nahi pata. Ub lekin mujhe yeh pata challa hai kay yeh severe depression mein hain.

**Interviewer:** Aur kabhi hospital mein admit hoye hain?

**Interviewee:** Nahi kabhi bhi nahi. Yeh kabhi high hojatey hain, aur kabhi depress hojatey hain. Isski bhi kaafi wajoohat hain. Yeh becharay kuch zyada.. yeh itne kind aur honest hain lekin inko reward mein kuch nahi mila balkey baar baar. Agar unhon ne awaz uthaye tu unko dabaya gaya. Newton ne kya kaha tha kay hur action ka eik reaction hota hai. Yeh reaction hai. Inki jo duty hai wahan per boht issues hain. Hur cheez mein yeh perfect hain. Inko kaha gaya tha kay inki posting hogi ..foreign posting … lekin kisi aur ki hogaye. Koi arab ki taraf bhejwa tha. Inka rank boht high tha lekin dusro ko miljati thee. Jo boht neechay thay unki sifarish hojatee thee. Matlab yeh boht mehnti hai. Inkay jo bosses hain inkay kaam say woh itna khush hotay thay aur yaqeen karein ..eik baar Hajj ka jo saara plan unhon ne kya tha, woh saari kharab hogaye thee. Phr meri dusri beti paida hogaye thee. Aap yaqeen Karein kay uss saal, I think 2003 tha, 50,000 cash unhon ney hur employee ko diye thay lekin Asim ko nahi diye thay. Kyunke inho ne yeh socha tha kay agar hum Asim ko zyada appreciate kareinge tu yeh apney aap ko boht zyada samajhney lag jayeinge. Phr iss waja say…

**Interviewer:** aap ki shaadi shuda zindagi mein maslay masail?

**Interviewee:** Buss yehi maslay masail hain. Haan yeh tha..matlab… inki ami hamari family mein say theen tu inkay bhai ne …inki ami ne jaidad ka case kardya tha. Iss waja say boht problems hogaye thay. Merey bhayon say inhon ne baat karna chordee thee lekin mein inkay saamney nahi keh saktee lekin inkay barey bhai ka qasoor tha. Yeh log boht intelligent thay. Police officer waghera hai woh tu buss boht agay thay. Iss ki waja say boht problems hoye hain.

**Interviewer:** Kya unki beemari ki waja say aap kay rishto pe koi farq para hai?

**Interviewee:** Kabbhi nahi

**Interviewer:** Acha aap ko kaise pata challa kay inko beemari develop horahi hai? Ya aap ne kya notice kya?

**Interviewee:** yeh job pe nahi jatey thay. Kamray tak mahdood hogaye thay. Inhon ne kitabein parhna shuru kardeen theen. And woh yeh kehne lag gaye kay jo uper ka tabqa hai woh neechay kay saath acha nahi kar raha. Baaqi cheezain boht sahi theen lekin is time bachon ko avoid karna shuru kardiya. Ubh mera yeh hai kay mein capable hun. Mein karsakteen hun. Lekin bacho kay liye baap ki shafqat boht zyada zaruri hai. Phr meiney inki bhen say batein discuss kareen. Isski bhen boht zyada acheen hain. Dunu ne mujhe yeh kaha Doctor ko dikhao. Lekin yeh boht late hogaye.

Buss yeh office nahi jaatay thay, ghar kay kaam mein koi dilchaspi nahi thee. Aur apnee sahet ka khayal nahi rakhtay thay.

**Interviewer:** uss time yeh doctor kay pass khud gaye?

**Interviewee:** Haan aur iss time yeh boht larai kartay thay. Apne boss say lartay thay. Inki negative soch hogaye. Phr mujhe shak hua kay kuch horaha hai. Lekin ubh Allah ka shukar hai bilkul sab theek hai

**Interviewer:** Acha aur unko beemari kay barey mein maloom hai?

**Interviewee:** Meiney bataya kay merey walid doctor hain. Jee unko pata hai.

**Interviewer:** Acha tu 2007 mein yeh khud doctor kay pass gaye?

**Interviewee:** haan aur phr jab baad mein hua tu unhon ne kaha kay haan theek hai

**Interviewer:** Acha aur aap ko khandaan ki taraf say madad miltee hai agar unki tabiat zyada kharab hogaye waghera? Bacho ko help chhaye ho?

**Interviewee:** Madad matlab?

**Interviewer:** Financial waghera? Ya emotional?

**Interviewee:** Merey parents kartein hain

**Interviewer:** Aur inkay parents?

**Interviewee:** inkay abbu faut hogaye.

**Interviewer:** Acha aur aap ko inki beemari ki waja say koi pareeshani ka saamna karna parta hai?

**Interviewee:** Boht. Karna parha tha. Matlab mujhe *pause* mujhe yeh hua tha kay yeh kyun aisee hogaye hain, matlab bacho waghera ka khayal nahi rakhna waghera. Aur merey liye kuch nahi phochna. Ghar pe tawaja nahi dena

**Interviewer:** Aur aisee konsi cheez thee jiss say aap ko ghussa aata hai?

**Interviewee:** buss woh yeh kay agar mein kahon kay meri tabiat theek nahi hai aur kahon kay doctor kay pass jaana hai ya yeh bolun kay bacho ki tabiat theek nahi hai. Lekin agar inki political activities say unko call ayegi kay chalo aaj yeh meeting karni hai, wall chalking karni hai ya banners banany hain tu woh chaley jaatey thay. Mujhe afsoos hota tha. Meiney apne bhai ko dekha hai aur apne abbu ko dekha hai. Iskay bhai ko dekha aur inkay liye bacho kay liye koi feelings nahi hai tu iss waja sey

**Interviewer:** Aap ko lagta hai kay aap jo inki madad karteen hain ussay inko madad miltee hai? Kya lagta hai aap inko support kar raheen hain?

**Interviewee:** haan

**Interviewer:** Kabhi yeh kehtay hain kay aap unko support kartee hain?

**Interviewee:** kehtay tu nahi hain lekin mein feel kartee hun. *smiles*

**Interviewer:** Acha aap ka doston mein aur khandaan mein aana jaana aur milna julna hota hai?

**Interviewee:** Milna milana itna nahi hota. Yeh chahtey hain kay hum zyada bahir jayein lekin meri first priority meri family hai. Mein chahteen hun kay mein apni family ko zyada time dun

**Interviewer:** Acha aur yeh kaafi social hain?

**Interviewee:** Haan kaafi social hain

**Interviewer:** Acha kya log inki beemari kay hawalay say aap say sawalat kartey hain?

**Interviewee:** Kabhi kisi ne bataya hee nahi hain

**Interviewer:** Aur acha aap ko lagta hai. Aap ki shaadi 2001 mein hoye thee aur inki beemari 2007 mein huwi thi. Aap ko lagta hai kay aap ko inmein aur aap ki relationship mein koi tabdeeli nazar aye?

**Interviewee:** *pause* Phele mein apney in-laws ki saath thee. Tu mein unkay kaamo waghera mein lag jaati thee. Mein inko time nahi deti hai. Aur yeh apne kaam mein hee masroof hotee theen. Aur 2005 mein hum ney ghar badla. Ussmein meiney dekha kay unhon ne bacho ki boht fikr rahi aur woh mujhe yeh kehtay thay kay hum ney bacho kay liye boht kuch karna hai. Aur hum ney yahan par nahi parhana aur bahir say education dena hai. Jiski waja say humein aur money ki zaroorat hai. Positive sochtay thay

**Interviewer:** Acha aur aap ko beemari kay barey mein pata challa tu aap ka kya radeamal tha? What was your reaction?

**Interviewee:** uswaqt tu meiney itna feel nahi kya lekin jab mein department mein aye idher aur logo ko dekha tu mein boht dar gaye.

**Interviewer:** Acha

**Interviewee:** ussay thora dar laga

**Interviewer:** aap kay bacho ko lagta hai kay inkay abbu ko beemari hai?

**Interviewee:** nahi kabhi nahi. Kyunke inkay bachay bhi inki tarah hain. Khaana kam khatey hain aur kitabeein zyada khateey hain. Lekin apne walid say boht mohabbat kartay hain. Inko boht acha lagta hai jab inkay walid ghar par hotay hain.

**Interviewer:** Hmm aur dusrey khadaan walo ko inki beemari kay barey mein pata hai? Seconds cousins, bhai waghera?

**Interviewee:** Nahi pata

**Interviewer:** doctor ko lekey jaanay ka inki bhen ne bataya? Aur ussay pheley aap kay abbu ne kaha tha kay koi problem hai?

**Interviewee:** Haan inki bhen khud bhee jaati hain. Inki in-laws mein problem hain actually. Tu unhon ne mujhe kaha kay jaana chahye. Theek haina?

**Interviewer:** Acha aur aap inki beemari kay baadh apni aur inki relationship mein koi farq mahsoos kartee hain? Koi change aya hai?

**Interviewee:** Boht boht. Mujhe aisa laga kay mere liye koi kuch nahi karta. Merey husband hee kuch nahi kartay. Jo kuch mujhe karna apne liye khud hee karna hai. Iskay baad meiney apni job shuru ki. Mujh mein ub boht zyada taqat hai kay mein kuch bhee karsakteen hun. Mujhe buss yeh lagta hai acha jab yeh mujhe kehtay hain kay theek hai mein karlunga tum araam karu waghera waghera.

**Interviewer:** Acha ub kartain hain?

**Interviewee:** Phele tu boht zyada kartay thay

**Interviewer:** Acha aur inki beemari ki waja say aap kay dusray rishto pe farq parta hai?

**Interviewee:** Nahi Karachi mein koi hai hee nahi. Bus bachay hain.

**Interviewer:** Aur bacho ko time deti hain?

**Interviewee:** Bachey inki tarah hain. Mera afternoon mein saara time bacho ka hai. Raat ko hum dinner kartay hain

**Interviewer:** Acha aur aap ko lagta hai inki beemari ki waja say aap ko tension aur zehni dabao waghera hota hai?

**Interviewee:** Boht zyada tension aur zehni dabao hai. Meiney aap ko pheley bhi bataya na kay mujhe apney bacho kay liye boht kuch chahye. Apna tu meiney socha kay allah mujhe tu dekh lega

**Interviewer:** Acha aur aap ne bataya tha kay 2007 mein jab yeh pheli dafa hua tha tu yeh boht ghussay mein rehtay thay. Aap par ghussa kartay thay?

**Interviewee:** Haan boht zyada

**Interviewer:** Cheekhtay chilatay thay?

**Interviewee:** Haan boht zyada

**Interviewer:** Kabhi ghussay mein aap ko mara?

**Interviewee:** Haan boht

**Interviewer:** Acha

**Interviewee:** phr meiney bhi mara *laughs*

**Interviewer:** Acha theek hai.

**Interviewee:** Inki family mein nahi hai marna waghera

**Interviewer:** Aap ko lagta hai yeh beemari ki waja say?

**Interviewee:** jee haan aur mein ney eik cheez yeh bhi notice ki thee jo inki activities hain mein unko join karun. Meiney kaha kay humaray bachay hain.

**Interviewer:** Activities matlab?

**Interviewee:** dharna waghera

**Interviewer:** Acha

**Interviewee:** yeh sab ubhi bhi yeh kartain hain. Jab yeh ahl-e-tasheeh ka hua tha. Mujhe keh rahay thay kay mein yahan chali jaon aur mein press club chala jaonga. Meiney kaha kay bachay hain.

*interruption in the interview*

**Interviewer:** Acha aur aap ko lagta hai inki beemari ki waja say aap ne additional responsibilities le lein hain?

**Interviewee:** Haan mujhe lagta hai jab say bachay paida huay hain meiney hee inki zemedari lei hai.

**Interviewer:** theek hai aur aap apney farig waqt mein kya karteen hain?

**Interviewee:** merey pass farig waqt hota hee nahi hai

**Interviewer:** Acha aur aap ko fariq waqt ka shauq hai kay aap sukoon say bethain?

**Interviewee:** haan boht shauq hai. Ubh yeh mera farig waqt hai. Ubhi merey bacho kay exams hain. Kal ka paper ka meiney unko tyaar karwa dya tha. Meiney kaha hai kay isskay baad hum shopping pe jayeinge.

**Interviewer:** Hmm, acha aap ko lagta hai kay aap ko inki beemari kay barey mein eik had tak maloom hai?

**Interviewee:** Mujhe boht zyada pata hai. Meiney kaha na jab mein yahan aye thi tu mein boht zyada dar gaye thee.

**Interviewer:** Aap ne aisa kya dekha tha kay aap dar gaye theen?

**Interviewee:** *pause* bus. Log cheekh rahay thay.

**Interviewer:** kabhi aap ne online search wagera kya?

**Interviewee:** Nahi. Mein nahi dekhti hun. Mujhe buss Allah pe bharoosa hai kay yeh theek hojayeingay. Aur Allah ka shukar hai theek horaha hai. inshAllah.

**Interviewer:** Acha aap ko lagta hai kay eik time aisa ayega kay yeh bilkul theek hojayeingay?

**Interviewee:** InshAllah

**Interviewer:** Acha aur aap ko lagta hai kay aap inko apni had tak sahi karsakteen hain? Ya zaroori doctor kay pass ayein hee?

**Interviewee:** Ub meiney observe kya hai kay mein theek nahi karasktee

**Interviewer:** Acha aap ko jab yeh boht zyada martay thay waghera tu kabhi aap ne socha kay aap chordein? Kay mein kyun chor dun?

**Interviewee:** mein observe kar rahi thee kay yeh chahtey thay kay yeh mujhe chordain.

**Interviewer:** Acha

**Interviewee:** istarah nahi boltay thay.

**Interviewer:** Aap ne kabhi nahi socha?

**Interviewee:** Nahi aisay nahi socha. Meiney socha chalo chal raha hai tu… Lekin mein aap ko eik baat bataon. Hum logo ka yeh tha kay agar mein Quetta (where her father lives), tu meray bacho ki education ka kya hoga. Meri bacho ki parhai kaisee ho. Yeh bhi Pathan hain lekin yeh alag hain thora. Matlab mein sab khuch karteen hun. Bazaar jaana, bacho ki parha kay barey mein sochna, admission karana, unkay liye research karna. Tu iss waja say.. lekin jab sey yeh theek hogaye tu buss..

**Interviewer:** Acha lekin aap ne kabhi aisa socha tha?

**Interviewee:** Hmm haan

**Interviewer:** Acha aur aap ko yeh lagta hai kay inki koi galtee ki waja say unko yeh beemari hai?

**Interviewee:** nahi bilkul bhi nahi. Mujhe yeh pata hai kay PIA mein jitney bhee log hain, inmein say yeh sab say achay hain isliye nahi yeh kay merey husband hain. Mein bari baat nahi kar rahi. Aap yaqeen karein, wahan chaltain hain. Aaj say kuch yeh taqreeban 8 9 saal pehely ki baat hai, jab mein gaye tu log aisay mil rahay thay unsay kay sir sir.. meiney asim say phoocha kay yeh kon hai tu unhon na kaha kay mein inka Sir hun. Mein inka boss hun. Lekin yeh itnay down to earth hain aur yeh itnay achay aadmi hain aur jo bhi mil jayein sab unki tareef kartay thay. Lekin ko inkay uper kay log hain unhon ne paisay khaney ki koshish karee aur inhon ne nahi khaaney diye. Cargo mein inki boht bari job thi, matlab jitnee bhee bari bari companies theen woh inko paisay dena chahtee thee aur in paiso say boht kuch hosakta tha. Boht bari raqam. Lekin yeh nahi lena chahte thay aur yeh pheechay hat gaye. Aur inhon ne chordiya. Tu ubh inka qasoor hai ya iska qasoor hai kay yeh acha hai?

**Interviewer:** Hmm..

**Interviewee:** yeh boht achay hain

**Interviewer:** Acha aur kabhi aap ko aap kay kisi family members ne kaha kay chordo?

**Interviewee:** nahi kabhi nahi *tears in her eyes*

**Interviewer:** Agar aap ko kisi sawal ka jawab nahi dena tu aap na dein

**Interviewee:** Nahi nahi bilkul mein theek hun.

**Interviewer:** Acha theek aur..aap ko kya lagta hai kay eik shaadi shuda joray key beech mein jo rishta hota hai woh zyada ahm lagta hai ya puri family zyada ahm lagti hai?

**Interviewee:** ahh

**Interviewer:** marriage zyada important hoti ya family?

**Interviewee:** Pura family zyada. Phele husband aur wife phr puri family

**Interviewer:** Aur aap ko lagta hai kay aap ki relationship mein hai?

**Interviewee:** haan

**Interviewer:** Acha aur aap ko kya lagta hai aisee kya soretahal mein eik shaadi shuda joray ko elaidgi ikhtiar karni chahye?

**Interviewee:** jab husband bilkul tawaja na dey. Wife bhi nahi tawaja ho. Agar husband yeh sochay kay mujhe wife kay liye kuch bhi nahi karna, yeh beemar hai, aur bacho ka bhi nahi kahyal rakhna ..matlab kuch bhi nahi karna. Acha mujhe yeh hai kay yeh theek hojaingay aur kareingee. Mein aap ko eik baat batati hun. Merei teen betyan hain, merey bacho ne bilkul itna capable hona hai kay woh koi bhi step zindagi mein uthanay ko tyar hun. Kay itna strong hojayein kay woh hur cheez karne ko ready hun. Aisa na ho kay woh yeh sochain kay if I don’t have my husband, I have nothing. You must be capable in your life. Whatever you want to do in your life, you must be strong enough to do anything. Tu yeh hai.

**Interviewer:** Hmmm

**Interviewee:** aur yeh aaj mein boltee hun kay unhon ne mujhe yeh realize karwaya hai hur insaan kuch bhee karsakta hai. Aap yaqeen karein mein zindagi mein kabhi rickshaw mein nahi bethi. Hamaray drivers hotay hain. Aur ubh mein chinchi mein bhi bethti hun aur mein bus mein bhi bethti hun. Aap yaqeen karein mein pheli dafa buss mein bethi thee tu mein gir gaye thee. Mein cheekh rahi thi aur saamne wali aurtain mujh per hans rahee theen lekin ub Allah ka shukr hai mein karleti hun. Bazaar jaana hona hai ya … Merey husband boht achay hain lekin kyunke inkay saath acha nahi hua tu buss

**Interviewer:** Acha aur aap ko lagta hai kay agar nafsiati beemari ho tu elaidgi ikhtiar karni chahye? Kisi ko bhi hoo?

**Interviewee:** Agar eik insaan bilkul sahet mand hai aur yeh sab kuch karta hai apni biwi kay saath ya biwi bilkul saheeh ho, lekin uss time beemari ho tu control mein nahi hota

**Interviewer:** Aap apna mustakbil kaisa dekhtee hain?

**Interviewee:** Acha

**Interviewer:** Acha aap ko lagta hai mazhab ki influence hai beemari mein? Mazhab ka koi taluq hai?

**Interviewee:** Agar aap mazhabi hongay tu saheeh hojayegi?

**Interviewer:** Hmm aap bataye?

**Interviewee:** okay okay please repeat your question

**Interviewer:** Matlab log kehtay haina kay yeh jin bhoot hain?

**Interviewee:** na na bilkul no no. Lekin mujhe yeh hai kay mein Allah say dua karungi kay yeh theek hojayein.

**Interviewer:** Aur aap ko kya lagta hai eik pursukoon khandaan ko raise karne kay liye kya cheezain zaruri hain?

**Interviewee:** patience, and tolerance is also very important

**Interviewer:** Acha aap ne marital counseling kay barey mein suna hai?

**Interviewee:** yeah

**Interviewer:** you think it will be useful when mental illness is the problem?

**Interviewee:** Bilkul karsaktee hai.

**Interviewer:** My questions are done. Do you have any feedback?

**Interviewee:** Mujhe acha laga. Mujhe aisa laga kay mujhe baat kar kay kaafi tension kam hoye. Mujhe boht acha laga. Aur yeh boht saheeh hogayein hai ubh.
