# Supplementary material for: Exploring effects of severe mental illnesses on marriages: A qualitative study from Karachi, Pakistan
Source: PLOS Glob Public Health. 2025 Dec 23;5(12):e0005652. doi: 10.1371/journal.pgph.0005652 (PMC12725543; doi:10.1371/journal.pgph.0005652)
Supplement: S1 Data — (ZIP) [file pgph.0005652.s001.zip › Transcriptions/Case 1 Transcripts/C1-11.docx]

**Case 1**

The wife has bipolar disorder. He was very willing to give the interview and showed no hesitation. In fact, he was quite happy to facilitate and also wanted to know the results of the study. He left his email address with the interviewer.

**Interviewer:** all right, so we want to find out about the impacts of the mental illness on the person who is actually caring for the mentally ill individuals. What are their problems etc.

**Interviewee:** All right, tou mujhe pata nahi tha. It was an arranged marriage. Mujhe tou pata nahi tha and after I got married, I got to know that my father-in-law had the same thing. He was depressed. But I didn’t know that my wife would get it. So after my two children were born, she went into a depression for reasons I don’t know. So we didn’t go to a doctor. 3-4 meheny kay baadh I don’t know automatically she came back to normal. *pause* so we thought it was all good. And then when her family split, she got depressive symptoms.

**Interviewer:** Split, I am sorry, what do you mean?

**Interviewee:** Her family got separated. Not me and my …her other family members. So she went into a depression. And then… we went to a few doctors. Dr. Essa and what not. And then finally we went into a GP. He was not .. what Dr. Moosa is.

**Interviewer:** Murad Musa is a psychiatrist. You went to a regular doctor

**Interviewee:** Yes, he gave her some medications and she put on so much weight. *pause* When my son got married, my daughter in law said that we should go see Dr. murad Musa. So we went and saw him. And she was good. She was …Dr Murad got her back to normal. And then she stopped taking the medicine. *pause* what happened next was that her elder brother had the same thing. But he went a bit berserk. He became alcoholic. And she …passed out…she just could not take that ..that her brother is in such a stage. For her, it was … and she started having all those attacks… So we brought her back to Dr. Murad Musa so he is looking after her.. yeah.. *laughs* And as of today, she is very stable. She had to go through the whole process again naturally and this is where we are today.

*fills out the demographic form*

**Interviewer:** Are you facing any kind of marital difficulties?

**Interviewee:** No not at all. Although it is very hard but now we know how to manage her moods.

**Interviewer:** All right, do you feel that you know about her diagnosis?

**Interviewee:** No not really. I just follow whatever Murad Moosa says.

**Interviewer:** But do you know about bipolar? That’s the diagnosis she has been given?

**Interviewee:** Yaar dekhou, I… am not a doctor..if you ask me what is bipolar..I would not give you a correct answer. If you ask me what is the difference between bipolar and depression, I would not know. I know a little about bipolar but if you ask me..is she a bipolar..then I don’t know.

**Interviewer:** Okay, that’s fine. So she was not diagnosed or never went to a psychiatrist before she got married to you, right?

**Interviewee:** She went to Dr. Shumail

**Interviewer:** Before she got married to you?

**Interviewee:** No no everything was after.

**Interviewer:** Everything was after? There was no diagnosis or signs of this problem before right? Before you guys got married?

**Interviewee:** Well no when I got married I came here a week before my wedding. So there was..how could I find out?

**Interviewer:** Well but did you ever ask her?

**Interviewee:** Never I was in Canada and she was here.

**Interviewer:** But after you guys got married, did you ever ask her if this was there from before?

**Interviewee:** No never. But after my marriage, after a year or two..after a year, I found that her father… I never discussed it with her because of one simple reason. Umm. She really didn’t want me to discuss it so I avoided it.

**Interviewer:** Well, the only reason I am asking you this again and again is because we have separated this study into a number of cases. So one of the cases is that the mental illness was diagnosed after marriage and another case is where the mental illness was diagnosed before marriage but the spouse did not know. So we have questions according to each case. So I don’t know where to put you. All right, so do you feel that the diagnosis was made before marriage and you did not know or do you feel that the diagnosis was made after marriage is more relevant for you?

**Interviewee:** Well all I know is that I did not know her family had this before her marriage.

**Interviewer:** But did she have a diagnosis before marriage?

**Interviewee:** No I don’t know..I don’t think so. Because she was very normal and everything

**Interviewer:** So we are assuming that the diagnosis was done after marriage. So were your parents aware of the spouse’s mental illness?

**Interviewee:** No

**Interviewer:** Any reasons?

**Interviewee:** Well no my parents did not know that and I don’t think my parents would have asked such questions. No. And I don’t think my parents would have *pause* even thought that she and her family would go into this depression and no..because my father was a very ..a very jolly man. He had no qualms. He didn’t hate anybody. So…. He would not have asked such questions.

**Interviewer:** So okay when she had her first episode…what was your reaction?

**Interviewee:** My first reaction was…first I couldn’t believe it… that was our reaction..and since we didn’t go to a doctor, we and my two children *pause* quiet and we didn’t want..especially my daughter..other people to know what my wife was going through. But I was very happy that all of a sudden one day, she was completely normal. It was so strange that after six months to a year, she came back completely normal. I didn’t know what happened. What triggered something in her brain or she fell down or she moved her head up and down..what happened..i don’t really know and she was normal. Absolutely normal. Absolutely

**Interviewer:** Initially, when second time she had her episode and when you people went to the doctor, so did you have any kind of support or help at that time?

**Interviewee:** No no. Only me and my children ..

**Interviewer:** Does it get stressful?

**Interviewee:** yes tremendously.

**Interviewer:** What kind of stresses do you feel? Are there any kind of hassles? Would you mind telling me?

**Interviewee:** Well not hassles. *pause* ummm… eik Abdul Faiz. He is at Aga Khan. We went to see him but nothing was really coming out of it. Stress is actually *pause* more on me trying my children…to keep her happy ..although I used to take the blunt of it. But I wanted my children to be away from it so that they don’t depressed or start wondering what was happening but I knew what was going through their mind. Both my children. And I was only tired then. I spent all my time taking care of her. But it was very difficult at that time. Extremely difficult. You really had to just keep your cool.

**Interviewer:** What were her symptoms like?

**Interviewee:** First time she was depressed, her symptoms were like that she didn’t want to see anybody. She would just sit there and just stare ahead. She wouldn’t go out ..we would force her to go out. Those were the symptoms. Then she came back to normal. Second when our family got separated, she thought that the world had stopped turning. She would sit down and she didn’t want to meet anyone.

**Interviewer:** She was in her own world.

**Interviewee:** Yes she was in her own world. Then we started her medications. Then we went to a GP and that is where she recovered but she had put on a lot of weight but I was pretty afraid and I had a really hard time. So we..after my son’s wedding, my daughter in law suggested Dr. Murad and we came to him.

**Interviewer:** Is it frustrating to come to the doctor hmm and give her medications? Is she compliant with her medications?

**Interviewee:** Oh yes I give her medications.

**Interviewer:** Okay so you give her medications? And take care of her? So does that get frustrating?

**Interviewee:** No no.

**Interviewer:** It’s part of your routine now?

**Interviewee:** Yes it has been part of my routine all my life

**Interviewer:** Okay and do you feel that your support helps her?

**Interviewee:** Yes ..but..what she goes through, only she knows. I can’t tell you what is going through her mind but yes she is…she depends now on me to give her the medicine but she doesn’t like being dictated at all. So I would be like don’t drink that Pepsi and she would be like no I am going to drink that Pepsi

*interruption in the interview*

**Interviewer:** All right, so initially you were saying that she didn’t want to go out but did you ever hesitate in taking her out? In friends?

**Interviewee:** No no

**Interviewer:** because you know there is the concept of stigma in society..

**Interviewee:** No no but I had some very dear friends. And although they knew what my wife was going through, they never… in fact they actually gave me support

**Interviewer:** Do others question about the illness of your spouse? You know how they always ask ..how people probe into personal lives over here?

**Interviewee:** Hmmm I would never tell them about the mental illness

**Interviewer:** Why do you think you never told them?

**Interviewee:** SO basically in the society, we live, there is this stigma. So I didn’t want that when she came out of it, I didn’t want people to say oh that there is something wrong with her.

**Interviewer:** So do you feel that after the mental illness, the family dynamics change in any way?

**Interviewee:** No

**Interviewer:** They were the same?

**Interviewee:** Because my daughter and even my daughter in law and my son..i mean they have understood…they have read more about bipolar and depression than I ever have so they understand what she is going through

**Interviewer:** What was your children’s reaction when they first find out about the illness?

**Interviewee:** AT first they couldn’t believe it.

**Interviewer:** Yeah denial

**Interviewee:** very much so but I had to sit down and explain to them that it was very difficult for me as well. But somehow we managed to get back to that stage and she came back.

**Interviewer:** So first time, if I remember correctly, you did not seek help. But second time, you went to a psychiatrist? So who encouraged you in seeking help?

**Interviewee:** Actually, it was my daughter ..so we went to the doctor and ..I was not very happy with the treatment but I knew very little about it so I went through with it

**Interviewer:** do you feel that your relationship has changed in any way or in any form since the onset of the illness?

**Interviewee:** No no. never

**Interviewer:** Did you have a good relationship before and after the mental illness?

**Interviewee:** Oh yes

**Interviewer:** Okay has it impacted your relationship with others in any way?

**Interviewee:** No

**Interviewer:** and do you feel that her mental illness has led to any mental problems of your own?

**Interviewee:** No. no.

**Interviewer:** Does she get violent ..because sometimes…

**Interviewee:** No.. but the trouble is that when she gets depressed, she wants to hibernate. At times, she goes wild..and she brings her past back.. she went back into the past mode about four or five months ago. She was just in the past ago. Her brain was making her remember what had happened to her from childhood to the marriage.

**Interviewer:** Did she feel that she was in the past or was she just recounting her past?

**Interviewee:** She was recounting her past. Because her mother died when she was 3 to 4 years old. And her step mother was not good to her. So all the past was coming back to her.

**Interviewer:** Hmm, hmmm. So she never gets violent or aggressive? Does she scream?

**Interviewee:** No not at all.

**Interviewer:** All right, now I am going to be asking about your family routine. So how is the day like? And what amount of time do you devote to taking care of her?

**Interviewee:** Oh I go to work at 9 30 am and I come back to 5 30 pm. Then we are just at home

**Interviewer:** All right. So have you taken any additional responsibilities after the mental illness of your spouse?

**Interviewee:** No no. She does all the housework. Because you have to keep her mind active.

*interruption in the interview*

**Interviewer:** All right but when she is in her hibernation mode, then at that time, do you have to take up additional responsibilities?

**Interviewee:** Woh kya hai. Woh meri beti karleti hai

**Interviewer:** Okay and what do you do in your leisure time?

**Interviewee:** Leisure time mein we watch television. We talk. Sit on computer and look at what’s happening and we go out only once a week. That is Saturday nights

**Interviewer:** do you feel that you know enough about the illness?

**Interviewee:** No

**Interviewer:** And what about your children?

**Interviewee:** Yes children know. And I also know. But if you ask me if I am a professor on it, then of course not.

**Interviewer:** Do you at any point in time feel that it is her fault to have the illness?

**Interviewee:** no. not at all.

**Interviewer:** Do you feel that you can fix her?

**Interviewee:** oh yes she has to be fixed.

**Interviewer:** Do you feel that you personally can fix her?

**Interviewee:** You see it’s the whole family that is going to help her.

**Interviewer:** You know there are times that people face so many problems in a marriage whenever there is a mentally ill spouse, they think of separation or divorce. Have you ever thought like that?

**Interviewee:** No never.

**Interviewer:** And your family members never suggested it?

**Interviewee:** no never

**Interviewer:** And in your opinion, when do you feel that a couple should seek divorce? Generally?

**Interviewee:** They should never seek divorce

**Interviewer:** what is the ground that you base your view upon?

**Interviewee:** They should learn to compromise

**Interviewer:** All right, so you chose to stay back in the marriage

**Interviewee:** Yes 100%

**Interviewer:** and what are the reasons that make you want to stay back?

**Interviewee:** Reason is that *pause* how can I let her go in this mental state? She cannot live a normal life. I mean this is my duty to help my wife and my family and there is anybody else, I would do the same thing, even if it was my friend, I would do the same thing

**Interviewer:** But of course with your wife, it is natural bonding that you have with her..right..

**Interviewee:** Of course. How can I leave her? It’s not possible

**Interviewer:** Do you feel that you were burdened at one point in time jab unka hibernation wala phase hota tha?

**Interviewee:** Yes there is no doubt about that

**Interviewer:** all right and what could have helped you at that time?

**Interviewee:** I had the will power to keep myself under control and help her get out of it

**Interviewer:** All right, do you feel that the marriage is more important or the family as a whole holds more importance?

**Interviewee:** Both

**Interviewer:** And what do you think are some of the essential building blocks for raising a healthy family?

**Interviewee:** Communication and understanding. You have to communicate everything to your kids and wife so that they don’t feel you’re hiding anything from them. Likewise, they have never not told me something they have done or hiding it from me

**Interviewer:** and okay how do you see your future?

**Interviewee:** Should be good!

**Interviewer:** So you’re hopeful?

**Interviewee:** Of course

**Interviewer:** And what is your view on marital counseling when mental illness is a problem? So you must have heard of marital counseling right?

**Interviewee:** Well, it depends upon the husband. If he can manage his wife, then I don’t think it is required but if husband cannot, then yes.

**Interviewer:** All right, I am done with my questions. Do you have anything to add?

**Interviewee:** No. Thank you.

***Interview Ends***
