# Supplementary material for: Exploring effects of severe mental illnesses on marriages: A qualitative study from Karachi, Pakistan
Source: PLOS Glob Public Health. 2025 Dec 23;5(12):e0005652. doi: 10.1371/journal.pgph.0005652 (PMC12725543; doi:10.1371/journal.pgph.0005652)
Supplement: S1 Data — (ZIP) [file pgph.0005652.s001.zip › Transcriptions/Case 1 Transcripts/C1-12.docx]

**Case 1**

**Psychiatric Illness:** Bipolar Disorder

*fills out consent form, explains the purpose of research, fills out the demographic form (teaching at a law school as a professor, is a legal practitioner, has been married for 28 years, previously in a joint family system, and now in a nuclear family setting, educated family with a daughter married and a son living, the wife is retired, was in a medical center, she was working as a chief medical officer, she has been taking medications for a long time, he had been worried for quite some time, she has been depressed for long and was also seeking treatment in the United States, she knows medications so she was taking medications for quite some time, in United States, people told her not to take the medications)

**Interviewer:** Jo inki kaifiat hai, yeh kitne arsay say hai?

**Interviewee:** Phele tu bata deti theen kay mein shaadi say pehle bhi medicine khatee thee, shaadi ke baad. Kabhi samajh nahi aye. 8-10 years tu kuch nahi samajh aya.

**Interviewer:** unkay behavior mein kuch nahi pata challa?

**Interviewee:** Nahi, kabhi bhee nahi. 8-10 years ..aur kaafi time tak kuch nahi pata challa, hum kaafi time tak chaltey rahe, jaana ana uthna bethna, kuch taqleef nahi hai, ultimately yehi hua maybe kisi dawai ka side effect tha ya reaction, uskay baad hum kaafi pareshaan huay hum umrah karne gaye huay thay, unhon ne dawai khai aur unko koi sujoon (swelling) agaye. Yeh kya horaha hai. Tu unhon ne kaha kay mein dawai khatee hun. Phr saamne zyada aana. Phr meri zidd hogaee kay aap jab bhi saath jayengi tu mujhe lekey jayenge lekin mujhe nahi leksay jaati theen. Bachay kay saath jaatee theen. Meri beti apni duty chornay ko ready thee aur aana chah rahee thee (she works as a doctor), but humne mana kya kay nahi aya. Phr ghar mein hum ney 10 saal say aisa mahol banaya hai kay hum kisi bhi baat ko rad nahi kartey. Jo bhi baat yeh kehteen hain hum accept kartay hain. Ussi tarah ki baat mein hum apne aap ko mould karletey hain.

**Interviewer:** 10 saal say mein hee aapko yeh problem rahi hai?

**Interviewee:** problem tu lagtee hee thee lekin yeh wohi baat mein arz kar raha hun na kay unhon ne self medication pe kara hua tha, kabhi khud aadhi goli kar dee kabhi 2 kardeen, doctors ne kuch likh kay diya aur unhon ne khud hee kardiya. Thyroid ka masla. Kisi din aankein sooj gaye aur hum hospital bagtey thay, hum ney tu cooperative attitude ikhtiar kya kay jo bhi kaam kar rahee hain hum koi acha tareeka nikalein aur inko koi pain feel na ho. Ghar mein koi masla nahi hai shuru mein problems huay thay jab hum sareey bhen bhai saath rehtay thay zahir hee see baat hai. Walda theen saath hamari. Theek thaak ghar hai, accommodation hai, phase 4 mein ghar hai, bizahar tu koi problem nahi tha. Koi rukawat nahi thee lekin jo meri bhen saath reh rahee theen woh tu u bunko 28 saal hogaye ub American mein hain doctor hain, uss baat..woh ghar say niklein, phr tu bhai thay, unki shaadi huwi aur woh America chaleygaye. Isstarah humaray ghar ka set up change hogaya. Ghar mein log kam hogaye aur usskay baadh woh thora relax hoyein. Unko yehi problem lagta tha kay itnay saarey log hain woh neechay aati theen. Hum ney kaha kay hum ne konsa kuch chupaya tha, eik maa hai, eik bhai hai, mein bara hun ghar mein. Woh mahul jo tha unkay favor mein relax hota gaya. Khaney waghera mein koi problems nahi. Cordial mahol hai. Parhey likay log hain. Lekin yeh shuru kardiya tha tu nikalna muhskil hogaya. 3-4 saal phele dawaiyan saari khatam karadeen theen. Comfortable hogayee theen. 2 saal tak unhon ne koi dawai nahi khaye aur ussi waqt bhi yehi baat kartee theen kay doctors nay fazul mein band kardeen. Unko pata nai kay dawai nahi band karni chahye. Doctor ko resist karteen theen. Baat baat pe. Agar woh samajhtey bhee thay tu sunti nahi theen. Duty mein bhi inko problem huwi thee. Unko shift kya gaya tha. Tu woh eik problem wahan per bhee detect huwi theen. Phir hum ney extraordinary care karna shuru kardee

**Interviewer:** Tu kitne arsay say yeh dawai par hain?

**Interviewee:** Merey khayal hai, at least 20 years tu must hain, but ussay bhee thora upper hai. Lekin kabhi kabhar yeh bhi baat kar jateen hain kay jab mein apni ama kay ghar thi tu uswaqt bhi mein kuch khatee the. Lekin uswaqt tu yeh hua tha kay inki walda faut hogayen aur woh bari attached theen tu uss sadmay ko bardasht nahi kya tha. Humein bari mushkil huwi un dinu unko sambhalna. Neend chalee gaye, unkay upper boht hee tabiat kharab hogaye. Jab uth jaati hain tu ubhi bhi kaam kardeti hain. Koi ghar kay kaam ka koi strain nahi hain. Aisa ghar ka mahol hai lekin pehele tha mushkil lekin buss woh eik time tha. Tu istarah kee beharal baat thee. Tu yeh strain zyada leti hain, boht sochteen hain, kehteen hain kay ghar bhi mera naam ka nahi hai tu ub kya hogaya tu meiney kaha kay kar deingay aap kay naam pe, aap theek tu hojao, aap itna strain kyun leti hain. Tu hum ney boht cooperate kera hai. Bacha bara pareshaan hai in kay hawaly say. Mein jaata hun subah roz kay kya khana hai nashtey mein. Yeh khud bhee hansteen hain kay kyun itna kar rahay hain, meri woh age tu nahi rahi kay itnee seeryan charun. Tu iss tareeqay. 2007 mein phir mera bi-pass operation hua tha yaheen Aga Khan say tu phr bhi. Jis tarah bhee uch neech kay halaat insaan ki zindagi mein aajatey hain, inhun ne bhi boht saath diya. Yeh puri family mein isstarah mashoor hain kay bara khuda tars hain. Eik billi ko bhee agar der say khaana miley tu jaan aajati hain unki. Kay ubhi tak bili ko dhood nahi pilaya. Insaniyat kay upper inki soch boht hee ache hai. Ubh yeh khud baat..kisi cheez ki zaroorat na ho. Yeh purani baatein yaad karkay pareshaan hoti hai. Inki koshish hoti hai kay mein kyun na khaon. Yahan halka sa sar mein dard hota hai tu inki soch wahan chalee jayegi kay meiney yeh goli 10 saal phele khaye thee woh theek hai aur yeh nahi hai. Buss istarah kee soch. Saara din yehi hota hai. Ubhi bhee jo aap logo ne dawai de thee, ismein eik medicine chor kay beth gaye hain. Doctor sahib ne kaha tha kay hospital say nikalne key baadh khaani hai, phr woh kehteen hai aap ko nahi pata yeh goli mujhe tazabyat paida kar rahi hai. Meiney kaha pata nahi kis naam ki. Khud hee band kartee hain.

**Interviewer:** Hmm, psychiatrist kay illawa aap ne kisi aur ki madad lee hai?

**Interviewee:** yeh Dr. Anees ne mujhe hee bataya tha, inki walda waghera lekey jaati hain. Homeopathic ka illaj bhee karwaya tha. Kay buss kisi tarah say yeh dawayan chordein aur yeh kaafi successful hogaye theen. Merey eik class fellow thay unhon ne kaafi effort kay baad kar kay churwai aur bola kay bhabi aap ne kya haal kya hua hai ghar ka. Tu unkay hisaab say working ki. Eik eik din karkey inhon ne dawayan khatam karwa deen. Ubh kuch nahi hun.

Jab beti ki shaadi huwi thee tu sarey gham bhul gayein thee, itna participate kya. Buss pata nahi kya hojata hai. Lekin agar koi taqraney ki koshish karein tu bardasht nahi hota

**Interviewer:** Kya aap ko pata hai kay inka diagnosis kya hai?

**Interviewee:** Kuch bhee nahi pata. Buss yeh golian kha rahi hain.

**Interviewer:** Acha, aur yeh hospital mein kyun admit theen?

**Interviewee:** Yeh tou unfortunately sirion yeh neechay gir gaye theen. Tu isliye. Apne bhen kay pass gayeen theen. Strain boht lejatee hain. Istarah nahi karna. Takhawat mein chor teen. Itnay saarey kaam kiye. Itna strain leliya. Itna travel kar kay gayein, uss per sirion say gir gayein. Itni chotain lageen. Phr hum emergency mein le ayein, phr kamray mein shift kya.

**Interviewer:** Theek hai, jo bhi kaifiat thee tu shaadi sey phele nahi thee?

**Interviewee:** Nahi.

**Interviewer:** unka ghar ka mahol kaisa tha?

**Interviewee:** Walid ki wafat jaldi hogaye thee tu boht attach theen walid kay saath. Tu shaadi key phele inko yeh sadma hua tha. Shaadi kay baadh meiney eik sadma tu dekha tha. Meri saas theen, due respect hoti hai, du teen maheney hum ghar mein inko control karney huay lagay huay thay. Buss issi mein rehjatey hain. Bhen kay yahan dua tha, bhai kay yahan hai. Hum khaufzada hojatay thay kay phr milengay aur royeaingay. Zahir hee see baat hai, afsoos hota hai. Phr jo condition achi howi thee tu reverse hojate hain

Phr walda ka jab intikaal hua Sindhi Muslim mein inka itna bara bangla tha, jab tu bhayon ne beechna shuru kyat ha tu inhon ne itna strain le lya tha kay merey walid kee nesshani thee. Attachment hoti haina insaan ki. Khaney peeney ya movement ki koi pareeshani nahi. Shaadi kay phele din say alag gaari aur driver sab milla. Aana jaana tu istarah ki facilities tu sab theen. But kuch cheezain na puri rahi hun. Tu zyati waghera tu koi nahi. Inki walda bhi boht acheen aur cooperative theen, lekin yeh sadmaat bardasht nahi karsakeen. Tu istarah kay mamlat chalein. Yeh buss zyada strain leleti hain, sadmat ki waghera say medication leleti hain, lekin aisa kuch nahi tha kay neend waghera ka masla tha.

Achi zindagi guzar rahi theen. Dawai kha raheen theen tu lekin kuch pata nahi challa kaafi arsay tak kay liye

**Interviewer:** Hmm, waise aap ka support kaisa hai jab pareeshani hoti hai tou?

**Interviewee:** Boht zyada support hai. Ghar ka mahol hai. Negative attitude tu develop nahi hosakta.

**Interviewer:** Jab aap pareshaan hotay hain tu aap ka kya reaction hota hai?

**Interviewee:** Mein jab pareshaan hota hai tu jaisay aaj meiney subah nashta kya, mujhe khud bhe dawai khaani hoti hai tu goli…apne anday kay saath inka anda bhee boil kya, raat ko meiney insay phoochay kay aap ne kya khaana hai. Kehney lageen kay mujhe bhook nahi lagteen. Meiney aur options diye. Chai waghera bana kay chalee gayein. Tray uthay kay lagayee. Dawai waghera bhee meiney deen. Unhon ne kaha kay nahi abhi mujhe neend arahi hai. Mein buss itna karta hun. Meiney kaha kay buss aap ki marzi hai, yeh idher khana waghera rakha hai. Akhbar waghera parh kya mein upper gaya tu meiney kaha kay aap ubhi tak so rahi hain. Tu isstarah kay mein kaam karleta hun. Dihaan tu idher hee rehta hai. Ubhi merey college mein exams chal rahay thay, aur meetings waghera theen lekin mein inko kabhi mahsoos nahi honay deta kay mera saath attachment mein koi kami horahi hai. Mein yahan ponay 3 baje ka aya huwa hun. Muhammad Ali society mein hamara campus hai. Mein 2 30 kay baadh lunch break mein yahan agaye.

Buss mujhe yeh hota hai kay inko taqleef na ho kistarah say.

**Interviewer:** Acha tu ub ko thakan, pareshani aur frustration hoti hai?

**Interviewee:** kabhi kabhar hoti hai lekin apne upper bardasht karleta hun. Eisha ki namaz parhne chala jaata hun. Eik lamba safar karlya. Tu is tarah ignore kardeti hun.

**Interviewer:** udaasi mahsoos hoti hai kay itnee achi zindagi…?

**Interviewee:** Nahi woh ubhi bhee hai. Dekhain na age factor agaya hai. Agar mein unko kabhi dur say dekhta hun tu meri aankhein bhar jaati hai kay inhon ne boht mehnat ki hai. Sachi baat bataon, kay eik din mein 300 300 mareez dekhteen theen. Non stop kaam karna. Khud sab karteen theen. Hum inko taqleef dena ka tasawur bhee nahi karsaktey. Waqt say pehele eik kaam.

**Interviewer:** Tu kabhi aap ko lagta hai kay over work hone ki waja say unki aisee kafiat hogi?

**Interviewee:** Lekin woh apni marzi say karteen theen. Khud kehteen theen kay merey pass du options hai kay mein State Bank ki doctor ban jatee ya PIDC ke banjatee tu kehney lageen kay haan PIDC ka mujhe flat bhee miljayega Bath Island mein, government ki naukri hai. Tu meiney yeh select kya. Tu meiney kaha kay boht ache baat hai tu apni marzi say woh kaam kar raheen theen. Kabhi kabhar khichao mein aajatee theen tu hum kehtay thay kay aap apni marzi say kar rahi hai tu khushi ke lehr mein lejayein. Tu yeh kabhi kabhi ulajh jaati theen.

Tu meri bhen jo hain woh inki dost theen. Unsay bhee boht ache dost theen. Buss boht zyada log thay ghar mein tu inko acha nahi lagta tha.

**Interviewer:** Acha ubhi aaj kal jo yeh pareshan hai, tu aap ka aana jaana aur ghumna phirna waghera hota hai?

**Interviewee:** Dil tu boht karta hai lekin jis waqt mein inko kehta hun kay chalein Anis bahir tu nahi, mein thak gaye hun. Meri routin different hai. Yeh puri raat jagtee hai inka pura routine kharab hogaya hai. Taqreeban eik saal say. Bari koshish ki lekin nahi set ho pa raha. Chalein meiney chordya kay neend tu puri karein koi nahi agar puray din soyein. Ub yeh jis waqt jagtee hain, mein ponay saath sey utha hua hotay hain, mujhe walk karni hoti hai, mein phr thak jaata hun. Aaj mein main Hamdard University bhee gaya hun j okay 27 miles from here, 50 60 kilometer travel kya hai, kaafi meetings attend kee hain

*interruption in the interview*

Mera acha khaasa zehni kaam hai, mein unko ahsaas nahi honay deta hunk ay mein thaka hua hun. Lekin after all, 10 10 30 mein thak gaye hun. Jab Faisal aur munazzah ja rahey hotay hain film dekhne kay liye tu mujhe bolteen hain chalnay ko tu mein kehta hun kay mein kaisay jaon, sojaonga. Aur mein mahazrat karleta hun. Beti kay saas susr aye huay thay tu hamara Dream world mein tha tu dupher mein inko time nahi suit karta aur mujhe raat mein time suit nahi karta. Zahir hai beti ka mamla tha tu duper mein hee karna parha

Timings buss coincide nahi hua pata.

**Interviewer:** Waise aap tu kaafi supportive hain jo kay husbands nahi hotay, lekin aap ko kabhi ghussa aata hai?

**Interviewee:** Ghussa tu mujhe sirf iss baat per aata hai kay bacha bhe inko dekh kar effect hogaya hai kaafi had tak. Inhun ne dawai khana suru kardee. Tu mein pareshaan hogaye. Apni wife ko tu meiney kuch nahi kaha. Mujhe ghussa aya kay dawai kyun de exercise karleta cholesterol. Mujhe buss hamdardi hojatee hai, mein balance karnay ki koshish karta hun.

**Interviewer:** Kabhi unhon ne ghussay mein koi cheez utha kay pheki waghera ho?

**Interviewee:** Shuru mein tu choti bhen ki waja say kaafi inka cooperative attitude tha. Dukh sukh mein bara saath diya tha. Usko actually breast cancer hua tha. Liaquat National Hospital mein dakhla karwaya. Eik room mein ghar mein rehteen theen, yeh log aik jaga dawat mein thay tu meiney tasveer lee. Tasweer mein meri choti beti aur bhen beti huwi thee. Tu wahan per unko boht ghussa aya. Humein bara khauf hogaya tha. Anis tum khud issko beti kehti hun aur yeh saarey kaam tumharay kartee hai, bell sun rahi. Tu boht friendly dosti thee. Uss din mujhe samajh nahi aye kay unko kya hua, kay meiney eik tasveer khachee aur yeh kursi pe jump karkein beth gayein. Uss baat key liye. Faisal ko boht mushkil sey pakra aur samjhaya kay itna log bethay hain. Yeh kehey lageen kay yahan kya tamasha horaha hai, tasveerain liye ja rahi hain tu ussper… kursi say utha ja bachi ko ghar nahi bheja yeh ishtiaal (ghussay) mein aye huwi theen. Tu yeh achanak kaifiat saal eik tu dafa aajati hai.

**Interviewer:** Yeh istarah ki aur kaifiat kabhi aye?

**Interviewee:** Haan lekin hum ne kuch aisa karnee hee nahi diya. Hum khauf kha jaatey hain kay inko kya hogaya tha. Sirf eik tasveer pe. Phr sahi haalat mein aaney kay liye inko at least 1 2 months lagtey hain lekin yeh kyun hota hai yeh samajh nahi aata. Meri beti bhi bari pareshaan thi. Lekin merey bachay boht cooperative hain. Merey bacha tu daant bhee deta hai kay abbu tu buss mein samajh jaata hun kay iss waqt khuch nahi bolna. Yeh ghussay mein bethi huwi. Hum boht cooperate kartay thay.

**Interviewer:** Acha doctor kay pass aaney ka mashwara kisne dya?

**Interviewee:** Yeh itnee dawaiayan le raheen theen tu mera dimag ghoom gaya. Meiney USA mein bhee dikhaya. Apna opinion form karleti hain aur phr change nahi karteen. Doctor sahib ne kuch galat kardiya. Mein tu dawaiyan khatam karaney kay liye inko leke aya. Mein aane ki koshish karta tha lekin yeh bolti theen bahir betho aap ko khud bulaynge. Saath rakhna aur baat karna kaafi mushkil kaam. Park lane hospital mein doctor ne mujhe koi baat nahi ki aur mera koi aaney ka faida nahi huwi

**Interviewer:** Dr. Murad kay pass kab say arahi hain?

**Interviewee:** Jab se hospitalize huwi hai aur chotain lagee hain tu wahan sey refer kya.

**Interviewer:** Tu yeh recent baat hai?

**Interviewee:** Jee ubhi hee baat hai

**Interviewer:** Aap ka din kaisa guzarta hai?

**Interviewee:** Buss hamara ghar mein kaam ka koi shor sharaba nahi hai. Mera ghar ka system he alag hai. Mein Shaukat Khanam mein legal advisor hun.

Hum boht zyada khayal rakhtain hain. Ghar ka mahol aisa hai kay sab unka khayal rakhay aur koi aisee baat na hu jiss kee waja say unka mood kharab ho. Buss woh tasveer wali baat nahi samajh aye. Afsoos bhi kya jab meri bhen chalee gaye.

**Interviewer:** Farig waqt mein aap kya karteen hain?

**Interviewee:** Hum student activity waghera karleti hain. Tennis waghera khail leta hun. Inkay saath ghumna phirna waghera. Puri dunya waghera dhundhi waghera. Paris utaray, London gaye. Bara acha ussmein hee tha. Yeh ubhi say nahi boht pehele say. By road hum Lahore gaye hain. Hotels mein bhii tehrahay hain. Joint family tha, Quetta waghera bhee chaley jaatey hain, chotay bhai kay saath chaleyga. Tension kabhi bhee nahi. Inn ka aisa zehn nahi hai kay bhai waghera kyun chal rahay hain. Unfortunately, pata nahi yeh mamla kyun hogaya hai. Lekin hum 8 10 saal say tu itna cooperative attitude hai kay dil ka tamana hai kay unko cheenk bhee nahi aye. Lekin yeh dawayion kay asrat say nikaltee hee nahi hain

**Interviewer:** Acha aur dawayon kay illawa kabhi aap ko aisa lagta hai kay inki issmein apni koi galtee hai?

**Interviewee:** Yeh buss inki galtee thee kay yeh kisi pe believe nahi karteen theen na apni walda pe na kuch. Na ghar walon par. Hosakta hai kay ghar walon ne mujhe na bataya hu but itna saal say nahi bataya, tu agar unki walda hee mujhe bata detein kay bhai iss cheez ka khayal karna yeh isstarah say neend ki golian khatee hain, tu shayad hum uswaqt thora aur ahtiat karletey. Tu ache khasi cheezain chup gaye hain. Chupaya nahi gaya, mein blame nahi de raha buss kay chup gayein.

**Interviewer:** Agar aap ki baat mantee tu yeh theek hojatein?

**Interviewee:** Saari batein manti hain meiney yeh bataya kay aaj kal hum sub insay phoochtay hain hur cheez ka. Kay kya karna hai. Takrao per hum nahi aatey. Agar takrao pe aajatey hain tu meiney bataya na kay buss tasveer keench lee, pata nahi kya tha kay meri tasveer nahi kheenchee. Uskay baadh phr eik dawat huwi, woh music bhee suntee gayein aur boht sarey mehman thay, choti bhen bhi wahan thee, Village restaurant mein metropole kay saath, tu participate bhee karte hain. Mein ney kal ka aap ko bataya kay Chinese khana meri bhen kya liye mangwaya. Kahan ukhar jaana hai, yeh baat samajh nahi aati. Buss humein yeh cheez khauf mein lati hai kay yeh eik dum khauf mein ne ajayein. Boht mushkil say samjhana parta hai, aur phr mujhee hee sab kehteen hain kay yeh sab khuch aap ki waja say hua hai, tum ne mujhe beemar kya hai, aur phr mein aur khamoosh hojata hun, kay hosakta hai kay meiney khuch kya hu. Isski waja say keh raheen hai. Beta khud aajata hai unchi awaaz sun kar aur phr unko utha leta hai aur phr samajhta hai. Tu phr tareqaar khatam hojata hai

**Interviewer:** Eik pursukoon khandaan kay liye kya cheezain ahmiat rakhteen hain?

**Interviewee:** Buss wohi baat ajatee hai kay coordination ho, eik cordial thinking, thinking match karjaye aur agar nahi horahi thinking tu eik insaan chup hojaye taakey agar aap tasadil pe ajayein tu phr tou..iss attitude kay saath mein surrender kardeta hun kay buss ubh thori zindagi tu rehti hai. Ubh yehi sochta hun kay jo soch unki hai, unki soch say hee chalta hun mein aur adjust karta hun. Ustarah hum isi tarah chaltey hain. Hum ney boht change kya apne aap ko.

**Interviewer:** Acha aur pheley say aap aisay hain ya inki beemari kay baadh?

**Interviewee:** Nahi pehle mein aisa nahi tha, jab mujhe 10 15 saalo say pata chala hai na , eik hamdardana jazba bhar gaya hai.

**Interviewer:** Phele aap boht strict hotay thay?

**Interviewee:** Nahi itna nahi tha. Aisa nahi tha kay apni hee baat manwani hai, cooperative attitude tu shuru say hee tha. Meiney kabhi nahi kaha kay Anis tum ne aaj khana nahi banaya, kay meiney tum istimal kya ho. Woh khud kehte hain kay kaisay shauhar hai, kabhi khanay peenay pe eik daant nahi piti. Puri umer mein. Namak zyada. Ya thanda hai. Meiney kaha kay in cheezo mein kya rakha hai. Aap ne education le hai, aap kaam layein ussko. Aap kay ma baap ne itnee mehnat karee hai, yeh aap ka zewar hai, aap dusrun ko taaleem agay dein. Aap education dein, aur mashAllah sab khuch system hai ghar mein, khana pakaney wali aati hai, safai wali aati hai, kisi qism ka koi problem nahi hai. Choti choti baatein aajati hain, agar gift dena hai tu agar eik pasand nahi aya tu dusra dedeya. Insaan hun zahir hee see baat hai mein apni raye deney ki koshish karta hun, lekin phr mein chup hojata hun kay meiney fazul ke rai dedi, yeh negative na lelein. Na soch rahi hun.

**Interviewer:** Tu aap pareshaan nahi hotay kay aap ko itna soch samajh kar baat karni partee hai?

**Interviewee:** Boht pareeshan bota hun. Aap phooch rahi hain tu mein waqt say phele sochna parta hai kay kis tarah mu say alfaaz nikale. Buss yeh baat hai kay agar mamooli cheez mein bhi inki baat kay takrao na karein tu yeh theek raheygeen. mashAllah say. Hur cheez samajh rahi hain. Yeh khuch saalo say aisa hua pheley yeh istarah nahi theen.

**Interviewer:** Acha kuch aur akhri sawal hain?

*interruption in the interview*

Aap kay liye kis cheez ke zyada ahmiat hoti hai, puray khandaan ki ya jo mian biwi kay beech main rishta hota hai? Ahmiat aap kis ko zyada detey hain?

**Interviewee:** Ji rishtay ko kynkee dekhain …rishtee insaan hain na..kabhi khatoon soch sakhtee hain, mard soch sakta hain, lekin jab bachay aatey hain tu sab khuch sochna parta hai.. beta hai uska sochna hai.

Bachay hain kaisay socha jasakta hai, aur agar Anis ki life tou miserable hojayegi. Khabhi aisa tasawvar bhee nahi kaya.

**Interviewer:** Tou rishtee ki zyada ahmiat hai?

**Interviewee:** jee zaroor.

**Interviewer:** Aap ka boht hee mazboot rishta hai, tu aap ko kya lagta hai kaisee soretahal mein talaaq leni chahye?

**Interviewee:** Tu dekhye agar isstarah kee halat mein agar koi istarah ka sochta hai, agar aisay halat mein koi insaan yeh soch raha hota hai kay meri betyan hee horahee hain tu usko meri soch koi aur hai, kyunke agar betian horahi hain tu uski qismat mein hee yehi hai, khair khai hai, tu yehi qabool karna chahye. Tu istarah ka mera zehn hai aur istarah ki positive soch hai. Agar wife kisi ki beemar hojaye aur shadeed tareen bhe beemar hojaye tu khudanakhusta bed ridden bhee hojatee hain tu mein kehta hun unko tu aur zyada zaroorat hai. Uswaqt tu shauhar ko apnee wife pe aur zyda qurbaan hojana chahye. Bajaye yeh kay woh khawab banata rahey kay mein eik aur shaadi karun. Mein bikul reverse gear mein hun iss baat per. Meri jo training hai bachpan ki aur barey hokay waghera, acha kaam karna chahye, bura kaam karna tu asaan hai tu burai ki taraf kyun jao, mushkil kaam karu tu woh acha kaam hoga na. Mein tu buss dua karta hun kay Allah unko boht khush rakhe.

**Interviewer:** aap apna mustaqbil kaisa dekhtain hain?

**Interviewee:** Humein tu ubhi bhee kaam karna hai. Mujhe tu ache jaga mil gaye hai. Jab tak haath paon salamat hain kam kareingay aur mein Anis ko kekhta hun kay woh relax karein. Bachay kee bhee shaadi ho. Isstarah ghar ka mahol ho buss. Bari different qism ki shaksiat hai meri, bilkul saadgi ki. Allah rasool ka khauf aur buss mazabhi soch kay kisi aur pe zydatee na hojaye chahye wife ho ya beti ho.

**Interviewer:** eik akhri sawal. Aap ne marital counseling kay barein mein suna hai?

**Interviewee:** Jee jee

**Interviewer:** Eik dusray ki agar baat nahi suntay. Tu aap ka kya khayal hai kay yeh helpful hoti hai?

**Interviewee:** Haan boht zyada. Agar dispute iss baat ka ho kay mian biwi bilkul…kay unhon ne buss soch liya ho kay yeh boht bari galtee hogaye aur khatoon ne bhee soch lya kya boht hogaya yeh tu hota hai kay hamari khaandani jung chal rahi thee, 3 murder yahan huay thay aur 3 wahan. Tu yahan per inevitable hojata hai kay agreement say bikul alag hojate hain. Counseling baaz dafa kaafi nakaam hote hain. Inkay apne bhai ki dusri shaadi huwi thee. Larai jhagra hota tha. Inko bari tension rahi hai ghareelo mamlaat ki waja say. Dusri shaadi jab hwui tu aaj bhi aulaad nahi hoti. Hur cheez mein inka boht attachment rehta hai.

But wahan jahan nahi ban rahi hun, wahan tu religion ne bhee kaha hai kay alag hojao. Lekin agar biwi ki naak cut gaye hai, baazo cut gaya hai, ya kuch aur hua hai tu wahan kuch kya karne ke zaroort hai, wahan tu aur zyada chahat ki zarorat hai. Mashira ne itna zulm kya aur phr iss khambakhat ne talaaq dedi agar kisi ne tazaib phaink dya waghera. Halanaky aap tu woh zyada haqdaar hai. Taleem aur soch agar. Counseling ka uswaqt haan, lekin be maqsad talaq mujhe theek nahi lagtee.

***Interview Ends***
