# Supplementary material for: Exploring effects of severe mental illnesses on marriages: A qualitative study from Karachi, Pakistan
Source: PLOS Glob Public Health. 2025 Dec 23;5(12):e0005652. doi: 10.1371/journal.pgph.0005652 (PMC12725543; doi:10.1371/journal.pgph.0005652)
Supplement: S1 Data — (ZIP) [file pgph.0005652.s001.zip › Transcriptions/Case 1 Transcripts/C1-13.docx]

**Case 1**

**In-patient (Ward)**

**Illness: Bipolar Disorder**

*briefs about the purpose of the interview*

**Interviewer:** shaadi say pheley diagnose hua tha ya nahi?

**Interviewee:** Nahi yeh tou first time hai. Hamari shaadi ko 10 saal hogaye hain.

**Interviewer:** Theek hai, aur shaadi say phele kuch pata he nahi tha?

**Interviewee:** nahi shaadi say phele kuch tha hee nahi. Yeh bilkul theek thay. Buss inko nazla zukaam zyada hojata tha. Aisee koi cheez nahi thee. Pyaar mohabat karne walay thay. Aisa bhee nahi tha kay alag rehna pasand tha unko. Mashallah sab kay saath beththay.

*fills out the consent form*

*fills out the demographic form* (details are in the demographic form)

**Interviewer:** Beemari ko itna arsa hua hai?

**Interviewee:** 3 months

**Interviewer:** Unki treatment uswaqt say horahi hai?

**Interviewee:** haan yeh boht zyada ro rahay thay. Taizi wali kaifiat thi. Hum ney Aga Khan mein clinic OPD mein dikhaya tha tu doctor ne kaha kay inko ward mein admit kardein. Lekin hum ney admit nahi karaya. Doctor ne tablet dee thee woh hum ne khilai raat ko lekin subah jab woh uthay tu torna porna waghera kya. Phr iss kay baadh hum unko Karachi nafsiat le gaye. Unho ne kaha kay Liaquat le jayein. Wahan per woh qaboo mein hee nahi arahay thay. Dimag unka bilkul sun hogaya tha pata nahi unhon ne kya diya tha. Ghar pe jab Liaquat National se discharge hokay gaye tu na kisi say boltay thay na baat kartey hain. Utha liya tu uth gaye, bitha liye tu baith gaye. Khila liye tu kha liya. Kabhi kuch nahi bola. Koi ehsaas nahi tha. Aisee kafiat thee.

General physician kay pass bee gaye but unhun ne galat guide kya. Kaha kay dimag mein infection hai.

**Interviewer:** Parhna parhanay walay kay pass gaye?

**Interviewee:** haan unkay pass bhi gaye. Dam wagera bhi karwaya.

**Interviewer:** Koi manshiat waghera ka istimal?

**Interviewee:** nahi lekin hamaray family doctor ne unko eik tablet dee thee sonay kay liye kyunke woh so nahi pa rahey thay eik maheny say. Tabiat unki phele say hee kharab thee. Unko ghabrahat horahi thee. Ronay lag jaatey hain. Kehtay thay kay dil nahi lag raha zindagi say dil uchaat hogaya. Khuch kardunga mein, apne aap ko mardunga. Aisee kaifiat hogaye thee. Phr daant nikalwaya tu yeh sochtay rehay key meiney daant kyun nikalwaya, mera mazboot daant tha, mein jitney bhee paisay kharch karlun mera daant wapis nahi ayega. Uskay barey mein pura din aur puri raat sochtay thay. Aankh band kartey thay tu foran uth jatey thay. Iss waja say neend na honay kee wajah say tabiat zyada kharab hogaye.

**Interviewer:** Acha aap ki koi shaadi shuda zindagi mein koi problems horahi hain?

**Interviewee:** Nahi mashAllah ubhi tu bilkul theek hai.

**Interviewer:** kisi say larai jhagra waghera?

**Interviewee:** nahi buss ghussay kay taiz hain tu kisi ko boldya.

**Interviewer:** acha tu beemari mein aur kya hua tha?

**Interviewee:** Buss baat cheet unhon ne kam karde thi. Bus yeh tha.

**Interviewer:** tu beemari kay barey mein kaisay maloom hua? Aisa kya hua tha?

**Interviewee:** yeh beemari tu accept hee nahi karna chahye. Hum koi bhi nahi karna chah rahey thay. Lekin jab unki itni severe condition hogaye, buss aisa samjhein kay inki saansein chal rahi theen aur body mein khuch nahi tha. Eik ungli tak nahi hil rahi thi. Makhian beth rahee thee unkay chehray per lekin yeh hatta nahi rahey thay. Itni severe condition thi kay woh khuch mahsoos hee nahi kar rahay thay. Body mein khuch mahsoos nahi horaha tha.

Phr Dr. Qurat ayein tu unhon ne dikha aur phr unhon ne kaha kay mein inka ilaaj karsaktee hun. Mujhe umeed hai kay yeh saheeh hojayeingay. Hum ECT waghera say ghabra rahey thay. Hum emergency mein bhi laye thay, jab condition serious thi, tu phr Liaquat National bhee legaye. Kaha dimag ka infection aur reaction hai. Buss apne charges waghera banaye. Neuro kay chakar mein lagaya tha. Test waghera kya aur phr unko heavy dose dediya tu bilkul sun hogaye. Phr hum inko wahan say nikal kar idher le aye emergency mein. Phir doctor ki treatment say woh kaafi behtar hogaye. MashAllah say yeh uth kay beth gaye aur sab ko phaichanay lag gaye, aur baatein karna lag gaye. MashAllah say saheeh hogaye. Phr unhon na kaha kay admit kardo. ICU mein bhi rakha heavy dose ki waja say. Injection waghera bhi lagey. Ubh unko injection nahi lag rahey aur medicine pe shuru hogayein

**Interviewer:** Kya aap kay waldeen ko inki beemari kay barey mein maloom hain?

**Interviewee:** merey walid tu hayat nahi hain aur walda hain aur haan sab ghar walo ko pata hai

**Interviewer:** tu aap ko apne khandaan walo say aur inkay khandaan walo say kisi qism ki madad miltee hai? Jab sey yeh admit hain?

**Interviewee:** Haan mashAllah hamara pura khandaan agay pheechay hai. Kisi ne nahi chorha. Jab bhi kisi ko bulatay hain woh foran hazir hojatey hain.

**Interviewer:** Tu jaisee pareeshani tu hoti hogi. Aap ko kis qism ki pareeshani ka saamna karna parta hai?

**Interviewee:** ubhi tak tou koi pareeshani ka saamna nahi karna parta kyunke hamari joint family hai. Saas susr boht achay hain. Mujhe ubhi khuch nahi pata kay paiso ka kis tareekay say hua hai waghera waghera. Jo khuch bhi horaha hai woh log hee kar rahay hain

**Interviewer:** lekin aap ko aana jaana waghera

**Interviewee:** Yeh bhi wohi log kar rahay hain.

**Interviewer:** doctor waghera say?

**Interviewee:** Sab wohi log kar rahay hain. Mujhe kisi qism ka bhoj (burden) nahi hai

**Interviewer:** Aur aap kay bachay waghera?

**Interviewee:** Woh log hee sambhal latey hain.

**Interviewer:** Tu aap apni shauhar ki zahir hee see baat hai madad kartee hongi. Aap ki madad say unka kis qism ka faida milta hai? Matlab phuchta hai?

**Interviewee:** Buss ub unki zaroorat hogaye hai. Khana peena jo bhi hai woh sab chahye unko. Tu din meheny tu meiney hee haath say khilaya hai. Khana pilana mein hee kar rahi thee. Yeh kisi aur kay haath say letey bhi nahi hain.

**Interviewer:** Aap ko kisi qism ki koi museebat waghera?

**Interviewee:** nahi nahi shauhar kay kaam karne mein kya museebat?

**Interviewer:** Tu jab sey beemari huwi hai, tu doston aur khandaan walo mein aana jaana hai ya khatam kardya hai?

**Interviewee:** Nahi sab aatey jaatey hain, miltay jultay hain mashallah say. Balkey ub log zyada aatey hain. Shaam mein tu yahan mehfil lag jaatee hain

**Interviewer:** Acha aur agar log sawal waghera kartay hain tu aap kya jawab deti hain?

**Interviewee:** Hum yeh kehtay hain kay yeh boht zyada crisis say guzray hain tu jism mein boht kamzoori hai, ubhi unko yeh nahi pata kay unka paani du daafa nikal chukka hai (referring to the treatment at Liaquat). Woh khud kehtay hain kay chalnay mein problem horahi hai, weakness hai

**Interviewer:** hmm lekin jo log aap say alag sawal kartay hain

**Interviewee:** Woh tu koi chupaney wali baat hai hee nahi. Bata detey hain kay yeh masla hai. Koi kehta hai kay kisi ne khuch karwa diya hai aur koi khuch kehta hai kay buss tabiat theek nahi hai. Hum ne woh ikaj bhi karwaya hai aur woh bhi karwaya hai.

**Interviewer:** Log nafsiati beemari kay barey mein phochtay hain?

**Interviewee:** Koi bhi aata hai unko yeh pata hee hota haina kay psychiatric ward hai

**Interviewer:** Tu koi zyada phoochtay hain?

**Interviewee:** Nahi bilkul nahi hai

**Interviewer:** Phichlay 3 maheney mein khandaan kay mahol mein khuch farq aya hai?

**Interviewee:** Humein pata hee nahi tha kay marz kya hai uswaqt.

**Interviewer:** tu ghar kay mahol mein koi tabdeeli?

**Interviewee:** Nahi buss ghar kay mahol mein..hum zyada dar gaye thay jo sachi baat hai. Itni severe condition thi. Kisi ko phaichaan nahi rahey thay, samajh nahi rahey thay. Koi cheez phainki woh khuch paink diya. Humein handle karna nahi araha tha. Iss waja say ghar ka mahol thora khofzada hogaya tha.

**Interviewer:** Aur ubh kaisa mahol hai?

**Interviewee:** Ubh Allah ka shukar hai

**Interviewer:** aur jab aap ko maloom hua kay inko yeh beemari hai tu aap ka kya radeamal tha?

**Interviewee:** Hum tu buss yeh soch rahay thay kay unko kya hogaya hai, aisa kya soch liya hai inhon ne, hum unko yehi kehtey thay kay itna mat socho aur dimag pe itna zor nahi daalo. Magar yeh nahi suntay thay. Unki buss soch atak gaye thi. Dimag say nikal nahi paati thee

**Interviewer:** Aur aap kay bachay..aap kay teen bachay haina, tu unko maloom hai?

**Interviewee:** Bachay itnay chotay hain kay inko ubhi khuch pata nahi hai

**Interviewer:** Kahbhi phoochtay hain kay kya hua?

**Interviewee:** nahi. Buss yeh pata hai ka baba beemar hai

**Interviewer:** Waisey aap ko kisne mashwara diya doctor ko dikhanay ka?

**Interviewee:** Doctor ko dikhana ka mashwara tu kisi ne nahi diya. Meray eik phoopa hain, Allah unka bhala karey. Unkay eik dost hain, unhon ney doctor ko page kya tha, humein pata bhee nahi tha kay emergency mein aap jayein. Inhon ne dikha aur kaha kay haan mein handle karsaktee hun. Warna hum tu umeed kho betay thay kay yeh kabhi saheeh nahi hongay. Humein yeh dart ha kay yeh kisi ko phaichaan nahi payeingay. Allah ka shukar hai kay yeh theek hogaye. Buss yeh hai kay ubhi inko ne medicines shoorat ki hai tu ubhi kam kee hai kyunke unki heart beat taiz hojatee thee. Warna koi masla nahi hai.

**Interviewer:** Tu aap ka apna jo zaati rishta hai beemari kay baad usmein koi changes aye hain?

**Interviewee:** Nahi nahi Allah ka shukar hai. Beemari mein yeh aksar boltay hain apni pheechli girlfriend kay barein mein. Sab cheezain keh rahay thay tu doctor ne kaha kay yeh beemari ka hisa hai.

**Interviewer:** Acha aur beemari ki waja say unkay dusray rishto mein koi changes aye hain?

**Interviewee:** Nahi nahi. Buss hansi mazaq kartay hain, sab pheley ki tarah hain

**Interviewer:** Unki beemari ki waja say aap kay zehn pe koi farq para hai? Koi udaasi aur pareeshani?

**Interviewee:** Udaasi tu huwi thi zahir hee see baat hai, zindagi bhar ka saath hai, udaasi huwi thi.

**Interviewer:** Acha tu aap ko lagta hai kay iss udaasi kay liye aap ko kisi doctor ko dikhana chahye? Kisi say baat karni chahye?

**Interviewee:** Buss Allah ka shukar. Jab tabiat sahi nee hoti tu tab zyada issue hota hai. Ubh buss bacho per tawaja unki kam hogaye hai. Pheley kehtay thay kay yeh karna hai yeh nahi karna. Saari fikrain agaye hain. Bacho ko school chorhnay jaana hai, bacho ko tuition chorna hai waghera. Zyada ahmiat apne bacho ko detey hain

**Interviewer:** tu ubh shuru mein aap keh rahi theen kay tankhua mein chalatay hain

**Interviewee:** Tankhua say buss hum bacho ka kartey hain. Aur jo hamara khaana waghera, ghar mein

**Interviewer:** Tu ubh yeh aaj kal kaam pe nahi jaatey tu..?

**Interviewee:** Ghar walee sambhal letey hain. Inkay dada daadi boht karletey hain

**Interviewer:** Aap bata reheen theen kay yeh aksar ghussay mein ajatay hain

**Interviewee:** Aksar nahi kabhi kabhar lekin ghussa zyada hota hai. Normal say zyada.

**Interviewer:** Ussmein aap ko dantey thay

**Interviewee:** Haan daant diya waghera

**Interviewer:** kahbhi maara waghera?

**Interviewee:** Nahi kabhi nahi marra

**Interviewer:** Please bura na manaye

**Interviewee:** Ghussa hojata hai lekin kabhi mara nahi. Kabhi tabiat say hutt kar cheez hojati hai tu chirchira pan ..waghera

**Interviewer:** Religion waghera ka aap ko lagta hai inka role hai nafsiat mein?

**Interviewee:** Mujhe tu nahi lagta, buss yeh tu sukhar ada kartey hain Allah ka.

**Interviewer:** Aap ka din kaisay guzarta hai?

**Interviewee:** Aaj kal tu hospital mein hee guzar jaata hai? *laughs*

Buss subah sey lekey shaam tak hospital mein hee hotay hain.

**Interviewer:** Acha tu khana waghera kon pakata hai?

**Interviewee:** Ami paka leti hain (referring to her mother in law). Meri eik nand hain unhon ne boht saath dya hai. Shuru say lekey aakhir tak. Paiso kay lihaaz say bhi aur hur lihaaz say

**Interviewer:** beemari kay baadh aap ki koi zeemadariyan bhari hain?

**Interviewee:** Ghar walon ne sirf unko sambhalney ki zimidaari de hai warna koi aur zimidaari merey uper nahi hai. Na bacho ko lekey, balkey mera eik 5 months ka baby hai, unko bhi inki dadi sambhal leti hain. Subah say lekey shaam tak feeder dedeti hain aur phr shaam mein mein feed kartee hun

**Interviewer:** Acha aur aap apney farig waqt mein kya kartee hain?

**Interviewee:** Fariq waqt milta nahi hai *laughs* ubh nahi milta, jab pehely milta tha tu kapray waghera see liye ya bazaar mein chaleegaye. Bhehen ki shaadi hai, sab kaam waghera karna hai

**Interviewer:** Tu aap ko lagta hai kay aap ko iss beemari kay barey mein maloom hai jo? Jo diagnosis hai?

**Interviewee:** bipolar ka diagnosis hai jiss mein kabhi kabhar taizi aati hai, lekin humehin hee dihaan rakhna pareyga jab taizi wali kafiat ayegee tu isskay liye bhi tu slow hojatein hain tu bhi. Ubhi normal hain

**Interviewer:** Tu doctor ne aap ko enough bataya hai? Aap ne sawalat waghera kya hain?

**Interviewee:** Jitna unhon ne bataya aur jitna hamari samjah mein aya, haan utna tu pata hai.

**Interviewer:** Acha buss mein aisay hee phooch rahi hun kay jab insaan pareeshan hota hai, jaisay beemari kay bareey mein aap ko pata chala hoga tu aap nay socha hoga agay ki zindagi kay barein mein.

**Interviewee:** Kay kaisi guzaroongi? Saheeh baat hai

**Interviewer:** tu aisee kya wajoohat hain kay inki beemari kay bawajood aap ne faisla kya kay mujhe isi shaadi mein rehna hai, isko barqarar rakhna hai?

**Interviewee:** zahir hai teen bachay hain MashAllah, aur isskay illawa pyaar mohabat husband say. Husband wife.

**Interviewer:** Acha, tu aap ko kisi waqt aisa laga kay inki koi galtee hai jis kee waja say unko beemari hai?

**Interviewee:** mein inko yeh samjhatee thee kay aap itna hyper nahi hua karu. Dararsal inkay jo walid hain woh bhi kabhi kabhar hyper hojatey hain. Tu sister in law bhee hyper hojatee hain. Yeh boht zyada hojatey hain. Inka dimag boht chalta hai. 5 cheezon ko dimag mein chal jatey hain. Tension letey hain. Hum inko kehtay thay kya itna hyper nahi hua karu, choti see baat par bigar jaatey hain aur phr aur choti see baat per hyper hojatey hain, ya phr chup hojate hain.

**Interviewer:** Tu aap ko lagta hai kay aap inko theek karsakteen hain?

**Interviewee:** Haan inshAllah, inshAllah

**Interviewer:** Acha tu meiney jaisay ubhi baat ki kay jab kabhi kabhar aisay maslay hotay hain tu log sochtay hain kay hum shaadi mein rahein ya na rahein tu aisee kabbhi aisa socha?

**Interviewee:** nahi kabhi bee nahi, kabhi bhee nahi

**Interviewer:** Kisi ne bola? Khandaan walo ney ya dost ne kabhi mashwara diya?

**Interviewee:** Nahi

**Interviewer:** Acha aap kay khayal mein, eik shaadi shuda joray ko kab talaaq leni chahye, kis sorehtahal mein?

**Interviewee:** Mera khayal hai kay jab tak bansakey insaan ko banani chaye hai. Koshish karne chahye. Insaan kay andar jitna bardasht ho. Jitna bardasht kartey ho, choti choti baat ko itna high nai karna chhaye. Ignore karna chahye choti choti baton ko aur agar ignore karney kay baadh samney wala banda bilkul nahi samajh pa raha hota hai aur aap ki mohabat ka koi asr nahi horaha hai tu uswaqt karna chahye. Aurat mein itnee taqat hoti hai kay woh apne husband ko control karsakey.

**Interviewer:** Aap apney future ko aap kistarah dektee hain? Aap adjust karsakteen hain?

**Interviewee:** Adjust tu karna pareyga

**Interviewer:** Tu aap agar beth kar sochteen hain kuch saal ka sochteen hain tu aap kay dimag mein kya khayal aata hai?

**Interviewee:** Buss yeh sochtee hun….

*interruption in the interview*

**Interviewer:** Jaisay eik khandaan hota hai, tu aap logo ka jo zaati rishta hota hai woh zyada important hota hai ya puri family?

**Interviewee:** Joint family

**Interviewer:** Aap ko kya lagta hai kay khandaan zyada ahmiat rakhta hai ya jo du logo kay beech mein jo rishta hota hai woh zyada important hota hai?

**Interviewee:** du logo ka, jo mian biwi ka rishta hota hai woh sab rishto say bhar kar hota hai. Uskay baad khandaan waghera aatey hain. Sab ki apni jagahein hoti hai.

**Interviewer:** Tu aap kay khayal mein eik pur sukoon khandaan kay liye kya cheezain zaruri hoti hain? Kin cheezon kay liye ahmiat hoti hain?

**Interviewee:** Hyper na hun. Relaxation honi chahye. Tension zyada na ho. Zyada baton ko apne dimag pe nahi lena chahye

**Interviewer:** Aap ne marital counseling kay barey mein kya soch hai?

**Interviewee:** Agar zaroorat parey tu karna chhaye. Marriage life is very important. Marriage life happy honi chahye.

**Interviewer:** okay, hamaray questions finish hogaye hain. Aap ko koi sawal karna hai?

**Interviewee:** Nai theek hai.

***Interview Ends***
