# Supplementary material for: Exploring effects of severe mental illnesses on marriages: A qualitative study from Karachi, Pakistan
Source: PLOS Glob Public Health. 2025 Dec 23;5(12):e0005652. doi: 10.1371/journal.pgph.0005652 (PMC12725543; doi:10.1371/journal.pgph.0005652)
Supplement: S1 Data — (ZIP) [file pgph.0005652.s001.zip › Transcriptions/Case 1 Transcripts/C1-14.docx]

**Case 1**

No one in the family is aware of the illness. Her mother and brother help

It’s annoying- she’s forgetful, I have to do double work in many things. Like “taking care of my daughter”

She’s not very social with people – I’d say she’s a-social and it embarrasses me because people will gossip.

There’s always a sudden change of behavior and that is what frustrates me the most

There isn’t much communication between us

- Does your support help her?

-I’m doing more than what most husbands do

**🡪**How often do you socialize?

Socialize every second day but I don’t like taking her out because since the last 6-7 months she has developed fixation of eyes.

“She’s not that ill”

Sometimes she talks to the wall

Daughter is more under stress

**🡪** What was your first reaction?

-I thought she was stressed out when she started talking to walls

-I became very concerned

-brother helped, he had seen somebody like this before

- As a family unit we have become a-social. I’ve withdrawn from other people.

My own mental health problem 🡪 stress- I have to do double the work

She knows she has a problem- she tries to fix herself- but she usually internalizes everything

Ever since my daughter started going to school I make the breakfast

- I buy her books, and her uniforms etc
- What do you do during your leisure time?

I’ve actually started to indulge in my leisure activities more (internet, TV)- I’ve moved away a bit because it keeps me okay. If I’m around my wife a lot or all the time I might lose control.

- Do you in any way feel it’s her fault?

I think it’s not her fault but she doesn’t or hasn’t utilized her brain of socialized in the last 10 years- hence she is vulnerable.

No to divorce- I’ve thought about it but now I have a daughter so not anymore

Marriage is for personal reasons. But with children a good family is essential- a good safe environment, money, and tolerance (in our modern society)

- How do you see your future?

No one knows the future, but yes we want to go abroad and are planning to do so

- Views on marriage counseling?

For younger couples marriage counseling could work wonders, but not for people of our age- its hard to change thinking and habits
