# Supplementary material for: Exploring effects of severe mental illnesses on marriages: A qualitative study from Karachi, Pakistan
Source: PLOS Glob Public Health. 2025 Dec 23;5(12):e0005652. doi: 10.1371/journal.pgph.0005652 (PMC12725543; doi:10.1371/journal.pgph.0005652)
Supplement: S1 Data — (ZIP) [file pgph.0005652.s001.zip › Transcriptions/Case 1 Transcripts/C1-15.docx]

**Case 1**

**Illness:** Obsessive-Compulsive Disorder

**Ward**

*fills out the consent form, and fills out the demographic questionnaire (he has two wives and his first wife was admitted in the hospital* poor audio quality though so some information may be lost.

**Interviewer:** Aap ki shaadi ko kitna arsa hua hai?

**Interviewee:** batees saal. 32 years hogaye hain

**Interviewer:** inko beemari kab sey hai?

**Interviewee:** Inko beemari tu hai 1994 say

**Interviewer:** Hmm inko aisa kya hua hai kay inki beemari diagnose huwi?

**Interviewee:** jab sey hamari shaadi huwi thee tu yeh bilkul theek theen. Hamaray bachay waghera paida huay. Sarey kaam saheeh kartay hain. Laikan phr yeh boht zyada haath waghera dhunay lag gayein, aur bartan boht dair tak dhonay lag gayein. Mein chup kay dekhta tha aur pareshaan hota tha. Napaki ka inko boht zyada hojaye. *inaudible*

Phr mein boht pareshaan hua tu meiney apne dost se discuss kyat ha tu unhon ne kaha kay dusri shaadi karlo. Meiney kaha kay kon shaadi kareyga mujhse tu dost ne kaha kay meri bhen haina. Shaadi kay baadh pata challa kay meri dusri biwi tu kaafi slow waghera hain. Woh beemar hain. Dhoka dya mujhe. Ab mein chor tu nahi sakta.

**Interviewer:** Acha yeh kabse dawayon per hai?

**Interviewee:** 1994 say hain

**Interviewer:** 1991 say beemaar hain aap ki wife?

**Interviewee:** Jee haan.

**Interviewer:** tu shaadi say pehele kuch nahi tha?

**Interviewee:** Nahi

**Interviewer:** Acha aap ko kisi qism ki koi madad miltee hai inki beemari ko manage karne mein? Koi madad karwata hun?

**Interviewee:** koi nahi karna

**Interviewer:** Aap ko pareeshaani ka saamna karna parta hai?

**Interviewee:** Haan

**Interviewer:** Sab sey zyada kya mushkil lagta?

**Interviewee:** Mushkil yeh lagta hai jab yeh suntee nahi hai aur apne aap dawayan kahteen hain.

**Interviewer:** aap logo ka milna malana hota hai? Doston aur rishtedaron mein

**Interviewee:** Nahi boht kam

**Interviewer:** Acha aur rishtedaron aur doston ko inki beemari kay barein mein maloom hai

**Interviewee:** Haan.

**Interviewer:** log sawal kartay hain?

**Interviewee:** Nahi itna tou nahi. Mein control karleta hun

**Interviewer:** Acha aap ki 1979 mein shaadi huwi thi , tu inki beemari kay baad aap ki shaadi shuda zindagi kis tarah tabdeel huwi?

**Interviewee:** Badli kya, buss boht problem hua.

**Interviewer:** Acha aur aap ko jab inki beemari kay barey mein pata chala tu aap ka kya radeamal tha?

**Interviewee:** Kya?

**Interviewer:** aap ne kya socha tha?

**Interviewee:** Buss kuch itna nahi samajh aya tha. Mein isko karwan-e-hayat waghera bhee lekey gaya tha.

**Interviewer:** Aap kay bacho ko inki nafsiati beemari kay barein mein maloom hai?

**Interviewee:** Haan maloom hai, buss zyada khayal nahi rakhtey.

**Interviewer:** acha aap ko kis ne kaha kay doctor ko dikhaye?

**Interviewee:** Inkay walid nay kaha tha.

**Interviewer:** aap ne dusri shaadi ke kya wajoohat theen?

**Interviewee:** unhon ne khud kaha tha kyunke bachay nahi sambhalay ja rahay thay

**Interviewer:** Acha aur aap ki mian biwi ki relationship mein kya farq para?

**Interviewee:**  Boht farq para.

**inaudible**
