# Supplementary material for: Exploring effects of severe mental illnesses on marriages: A qualitative study from Karachi, Pakistan
Source: PLOS Glob Public Health. 2025 Dec 23;5(12):e0005652. doi: 10.1371/journal.pgph.0005652 (PMC12725543; doi:10.1371/journal.pgph.0005652)
Supplement: S1 Data — (ZIP) [file pgph.0005652.s001.zip › Transcriptions/Case 1 Transcripts/C1-16.docx]

**Case 1**

**Illness:** Bipolar Disorder

*fills out the consent form and the demographic questionnaire, live in a nuclear setting, they have 3 children, and all of them are married and live separately*

**Interviewer:** Shaadi ko kitnay saal hogaye hain taqreeban?

**Interviewee:** 33

**Interviewer:** Aap ko khud hai kisi qism ka koi psychiatric masla?

**Interviewee:** I don’t know

**Interviewer:** Any other sort of pareshaani?

**Interviewee:** Of course, life ka andar stress tu rehta hai. Aur agar saheeh tareeqay say uskay face nahi karein tou problem hojata hai. Yeh frustration anxiety create karta hai, aur anxiety say insaan pareeshan hojata hai. Aur *pause* jo bhi stress parta hai usko divert karlena chahye warna woh aap kay andar beth jayega. Mind mein tu hoga hee. Agar aap ne bilkul usko andar corner mein bitha kay rakha hai woh aap ko distract karlega kisi tarah say bhee aap kay lifesyle say waghera. Mein taqreeban *pause* mein khud eik psychiatrist ban chuka hun, studies kar kay aur jab sey inko beemari huwi hai. Meiney kaafi studies keen aur doctors say bhee mila hun.

**Interviewer:** Jee hum yeh bhee jaana chah rahay hain kay logo ko kitnee information hai.

**Interviewee:** Because inko jab first time yeh hua tha tu it was in 1982 and 1981 mein shaadi hogayee. Yeh apni family kay pass thee. Yeh delivery karwanay gaye theen aur mein country mein nahi tha. Jis waqt mein usko lena gaya tu uskay ma baap ne bataya kay usko jinn char gaya hai. Meiney nahi mana kyunke aaj kal aisa kuch nahi hota hai. Yeh eik saal kay baadh challa, lekin after she told me that she suffered from this before also. Tabiyat kharab hoti thee tu uskay parents isko mullao kay pass lejatey thay. Mujhe bhee inhon ne kaafi mullao kay pass phanswaya. Yahan tak kay black magic wala bhee garbar karaha tha aur ussney bhee kya. Mujhe koi asr nahi hua pani ka jo inhon ne diya. She was hospitalized since five days in labor pain, but parents ne care nahi kya jisski waja say yeh hua. Care nahi kya aur akela chor ka chaleygaye. Akele jab theen tu uski water bag burst huwi tu complications hogayeen. Koi hota ghar pe tou bhagta dur kay. Hospitalized karke. Subah hospital lekey gaye. Iss halaat mein aurat internal jo guzartee. It was too late. Superstitious cheezain keen inhon. Bacha lucky tha jo bach gaya. Warna tou woh chal basta, aaj uski mashAllah shaadi bhee hogaye hai. *pause* jab mein phuncha tu uski haalat theek nahi thee. Yeh log kehrahay thay kay shaitan char gaya, meiney kaha kay aap logo ko dikha nahi aap logo ne pakar nahi liya. Meiney unko kaha kay aap ne care nahi kya. Mein eik aur baat batadun. Inki mother bhee suffer kartee hain.

**Interviewer:** past psychiatric history hai family mein..

**Interviewee:** yes but kabhi unki treatment nahi huwi. Number 2 her husband and her issues. They separated many times during their lifetime. Six months, 1 year to 2 years. Bachay pareeshaan hojatey thay, kharcha nahi bhejta tha baap. Aur yeh suffer kartay thay *pause* Ubh iski waja say chota bhai, yeh log 4 bhai bhen hain, yeh sab sey bari hai, chota bhai bhi suffer kar raha. Woh bhi treatment karwa raha hai. Jab mein doctor kay pass lekey gaya tou unhon ne kaha kay lithium deficiency hai

**Interviewer:** Kiss year mein yeh hua?

**Interviewee:** 82 mein hee hua tha. Lithium deficiency hai. Bipolar Disorder kaha. Karachi mein lakay dusray doctors ko dikhaya. Unhon ne Bipolar kaha. Schizophrenia bhee kaha. Dusre doctors kay pass jaata tha, Dr. Anis theen woh. I disliked her. Reason was that she used to talk to both of us at the same time and she used to object to what I would say and then I would face problems back at home. Separately nahi baat karteen theen. Buss unko woh dawai deti theen aur kehteen theen kay woh regularly ate rahain. Mein inko kehta tha bhai aap suffer nahi kar rahee hain, mein kar raha hun. Tu patient se tu pact bana liya but mujhe boht zyada disturb kar rahi ho. Unhon ne eik dafa aisa hee kaha tha, yeh 3 saal ki baat hai, meiney kaha kay mein second opinion lena chahta hun. Pehele bhiee eik doctor thay nazimabad mein, naam mein bhool gaya hun unka.

*interruption in the interview*

Meiney itnay doctors say discuss kya kay itnay problems. Eik physician sey baat ki thee tu unhon ne kaha kay neurosis hai. Phr meiney check kya, jo isska pattern hai, beemari ka pattern hai woh match kar raha tha. Rivotril dawai acha affect kar rahee thee. Inhon ne kaha issey boht neend aarahi hai. Tu stop kardee dawai aur dusri shuru ki jo kay theek nahi thee. Yeh 2 maheney ki baad hai ya shayad 3 meheny, mein homeopathic kay pass bhi check karwaya. Drops dediya thay. Phr I said lets try this. Yeh baat manti nahi hai. Yeh kehtee hai kay tum doctors ko rishwat detey ho dawai deney kay liye. Phr meiney unko prescribe kya aur phr meiney kaha kay improvement bhar rahi hai. 10 drops raat ko and 10 drops in the morning, jo standard hai, woh de rahay hun. mashAllah kaafi farq par raha hai.

**Interviewer:** Aap log aapis mein rishtedaar hain?

**Interviewee:** Nahi. Dur kay hain

**Interviewer:** Acha yeh kabhi hospital mein admit huwi hai?

**Interviewee:** Yahan kabhi Karachi mein nahi hun. Haan shayad eik baar. Islamabad mein du baar huwi theen

**Interviewer:** Acha tou koi episode hota hai kya? Kyun admit huwi?

**Interviewee:** Kaafi violent hojatee theen

**Interviewer:** Acha tu taqreeban kitni dafa hua hai?

**Interviewee:** 50 dafa hua hoga. Treatment nahi mil rahee thee. Doctors samjhtey nahi thay. Lifestyle hamara different hai, woh usko catch nahi kar pa rahay thay. Pehle kehte thay lithium deficiency hai, bipolar disorder hai phr schizophrenia pe aye, yeh sab kuch mix horaha tha. Eik doctor bhee nahi hota tha case mein. Transfer hogaya. Wapis history samjhao waghera.

**Interviewer:** Kitnay arsay say yeh total dawaiyon per hain? Jaisee 1982 say?

**Interviewee:** 1983 say.

**Interviewer:** Kaafi logo kay pass gaye hain, jaadu waghera, homeopathic kay pass bhee?

**Interviewee:** Well yeh tou mein lekey gaye thee. Inki violence pe control aya hua.

**Interviewer:** Koi nasha waghera?

**Interviewee:** Na na

*many things unrelated to the interview however the spouse mentions that he smokes and is a heart patient and has had multiple heart attacks and angioplasty etc*

**Interviewer:** Aap ko beemari kay barey mein kab maloom hua?

**Interviewee:** Mein beemari tu samjha 3-4 saal baad. Interest leta gaya.

**Interviewer:** Shaadi kay 3 saal baadh

**Interviewee:** Nahi shaadi kay 3 saal baad nahi, attack kay 3-4 saal baadh. Meiney boht research karee. Yeh attack delivery kay baad hua tha

**Interviewer:** Acha aur delivery shaadi kay kitnay arsay baad huwi?

**Interviewee:** 1981 mein shaadi huw and 1982 mein delivery huwi.

**Interviewer:** Aur aap kay waldeen ko maloom hain inki beemari kay barey mein?

**Interviewee:** Mere walda hayat nahi theen uswaqt. Walid sahib ko pata challa, walid sahib humaray saath rehtey thay lekin iski condition ki waja say, mein tou bardasht karleta tha, lekin merey walid sahib ko taqleef horahi thee, woh kehtey tu nahi thay lekin meiney barey bhai ko kaha kay apne pass shift karlein taaqey problem na ho.

**Interviewer:** Hm

**Interviewee:** Bacho ko meiney paala hai, nilaya aur dulhaya bhee meiney hai, kaafi problems ka saamna karna par raha hai. Sab mujhe karna parta tha. Koi mujh sey, office mein agar meri secretary utha le phone woh lady hai tu museebat hojatee thee. Ubhi bhee kisi bhi larki say baat karta hun, koi bhi hai, usmein kaafi problem huwi thee. Parosion say kehtee thee kay tumhein merey husband say kya chahye tha.

**Interviewer:** Theek hai, waisay aap ka support system kaisa hai? Aap ko kisi qism ki madad miltee hai jab pareeshani hoti hai ya boht zyada kaifiat kharab hoti hai? Tu phr koi support system?

**Interviewee:** Nahi

**Interviewer:** Bachay waghera?

**Interviewee:** bachay kartay hain lekin control nahi karsaktey. Mujhe tu marna waghera bhee parta hai.

**Interviewer:** Kitni pareshani hojatee hai? Kitna stressful hojata hai? Kabhi ronay waghera ka dil karta hai? Ya kuch?

**Interviewee:** Ronay ka tu dil nahi chhta. Afsos hota hai iski condition ko dekh kay, ghum hota hai. I feel it. Kay yeh isko khud nahi maloom kay yeh kitni taqleef mein khud hai.

**Interviewer:** Theek hai aur aap ko pareshani hoti hai?

**Interviewee:** Haan kaafi pareshaani hoti hai. In and out meiney different jobs change ki hain. Ub bachay na kaha hai kay aap araam karein, usko handle karein, aur aap fikr na karein, hum buss guzara karleingay jitna bhee hai

**Interviewer:** Tu aap ko thakan waghera mahsoos hota hai aur mayoosi waghera?

**Interviewee:** mayoosi waghera nahi hota hai. Jab bhee koi pareshaani hoti hai mein isskay ma baap ko bula letey hun. And baaki khuda say dua mangta hun kay khuda issko sukoon dey. Yeh realize kartee hai kabhee kabhar kay mein beemar hogaye thee aur mein yeh kar rahi thee lekin issko usually hosh nahi hota. Many times, logo ne mujhe kaha kay kyun divorce nahi detey ho.

**Interviewer:** Yehi agay hum phoochangay.

Acha aap ko lagta hai kay aap ne jo inko sahara diya hua hai, inko kisi qism ki madad miltee hai inko?

**Interviewee:** Yeh mujhe nahi pata. Woh feel kartee hai usko. Isskay bhai log kehtay hain kay you’re doing a lot but iskay ma baap ne kabhi iss cheez ko samjha nahi. Aur mein ghar kay inkay mahol ko dekh kar isko chor nahi sakta. Yeh jayegi aur yeh aur pareshaan hojayegi. Jab phehle iskay liye kuch nahi karsakey tu ubh kya kareingay? Aur ub yeh hai kay yeh feel kartee hain. Isko hit kar jateen hain thori throi cheezain. Mein sab ko nahi samjha sakta kay yeh beemari hai tou koi na koi choti cheezain inflict karjateen hain.

**Interviewer:** Aap ne kaha nay kay aap sab ko nahi bata saktey tou hamaray aas pass logo ko sawal karney ki adat hai. Tu jaisay aap say koi phoochta hai inki beemari kay barey mein tou aap kya sawal ka jawab detey hain?

**Interviewee:** Mein kehta hai hun kay neurosis hai

**Interviewer:** tu aap bata detey hain? Aap ye nahi sochtay kay log kya sochaingay?

**Interviewee:** Nahi. Logo ka mujhe koi farq nahi parta. Parosion ko bhee meney bata diya hai kay yeh beemari hai. They know how to ignore. They distance themselves.

**Interviewer:** Saheeh. Tu aap logo ka ghoomna phirna hota hai? Doston mein milna milana hota hai?

**Interviewee:** Jab inki condition theek hoti hain tu haan hur jaga jaatey hain. Picnic pe jaatey hain.

**Interviewer:** Maheney mein kitni dafa aajatey hain?

**Interviewee:** Hur week hee chaley jaatey hain. Akele hain hum log. Khanay waghera lejata hun. Nahi paka rahee 15-20 din tak tou bahir lejata hun. Dawat dedeti hai aur mujhe nahi batati. Discuss nahi kartee. Kyunke woh kaheygee tu mein mana kardunga, kyunke pareeshani khud uthatee hai. Aur saath mein mujhe bhee pareshaan kartee hai. Tyarian lekin eik two din phele karleti hai. Kabhi mein kahoonga kay chai banao tu nahi banayegi.

**Interviewer:** Waise tu aap keh rahay hain kay shaadi kay shuru say hee inki yeh kaifiat hai tu mahol aisa hee hai shuru say ya tabdeel hua hai?

**Interviewee:** Pheley zyada muhskil thee. Mujhe bacho ko bhee dekhna hota tha, aur usko bhee dekhna hota tha. Kaam ko bhi dekhna hota tha. I was working in Treasury, accounting and finance. Effect mujhe boht karta hai.

**Interviewer:** But aap ko kabhi aisa laga kay aap dusray khandano sey mukhtalif hain ya kaash kuch alag hota?

**Interviewee:** Nahi kabhi mujhe aisa nahi hua. Mujhe kisi aur say shaadi karni thee tu nahi huwi tu koi masla nahi.

**Interviewer:** Acha jab aap Kuwait say wapis aye aur unka yeh haal that u aap ka kya reaction tha?

**Interviewee:** Mein merey bachay ko dekh raha tha. Isko nahi dekh raha tha. Agar meri walda hayat hoteen tu mein bachay ko lejata. Mein paal leta. Aur bachay ko iss umer mein, 2 saal tak tu ma ki zaroorat hoti hee hai. Koi bhee nahi istarah pyaar kareyga merey ma kay bajaye ya iski ma kay bajaye. Mujhe kaafi offers milee shaadi ki lekin sab ne kaha kay talaaq dedou isko.

**Interviewer:** acha tu aap kay bacho ko kya maloom hai kay kya exactly masla hai?

**Interviewee:** Haan inko pata hai

**Interviewer:** Uswaqt tu chotay thay. Tu jab barey huay tu phr unka reaction tha?

**Interviewee:** Achay bhee rehtay thay aur ghussa bhee hojata tha. Bachay jab barey hotay hain tu bardasht nahi kartay thay. Eik normal see cheez hai. Mein tu apne bacho kay liye survive kya hun. Isko sambhala isliye. Acha mein apne bachion say baat nahi karsakta tha, kyunke inko acha nahi lagta. Mujhe yeh cheezain kaafi buri lagteen theen aur mujhe pata hai kay yeh khud nahi keh raheen iski beemari ki waja say yeh tha. Mujhe usko aksar control karne kay liye voice raise karnee partee thee or I also used to raise my hand. Merey bhai waghera sab ko pata hai kaisee tabiat hai. Iski family ko bhee pata hai kay kaisi tabiat hai. Aur mein sab ko kehta hun kay phele mujh sey akey baat karu phr ussay baat karna. Agar ussay baat karoge tu khud tabiat kharab hogee ussay.

**Interviewer:** Tu waise aap ne mashwara kisne diya tha kay doctor ko dikhain?

**Interviewee:** Mein khud doctor ko dikhaya. Meiney khud dekha aur talaash kya. Mein insay boht dur hun, sometimes I discuss with her. Ghar ka mahol aur jo understanding family ka mahol hota hai, kuch hai nahi. Doctor patient should understand each other tu idher mujhe yeh milla hai. 1 ghanta idher doctor bethay hai, mujhe 2 maheny uskay saath uthna bethna aur khayal rakhna hota hai. Kya problem mujhe hota hai woh aap ko nahi pata. 10-15 din ki baat cheet say aap ko kuch nahi pata chaleyga. He understands that. He knows when to change the medicine. Ubhi jo dawayan de rahay hain woh bilkul theek hain. Meiney unko kaha hai kay dawayan ubh change na karein.

**Interviewer:** Aap ne thora apney rishtay key barey mein bataya. Aap ko kya lagta hai kay inki beemari ki waja say aap logo kay zaati rishtey pe kya farq aya hai?

**Interviewee:** Well *pause* zyada khaas nahi sab ko inki beemari kay barey mein pata hai… yeh hai kay

**Interviewer:** Aap dunu kay jo mian biwi ka rishta hota hai jo emotions ka? Ya jo dosti aur understanding?

**Interviewee:** Sirf change yeh aya hai, attraction, sexual attraction nahi raha. She feels it. I try to satisfy her sometimes, orally I make her satisfy. I can’t do it sometimes. She needs it more. Mein usko guide kardeta hun. Otherwise I don’t feel like having sex with her. Tou yeh farq hai, baaki milna julna, hug karna, kiss karna hansi mazaq waghera sab hai. Kabhi ultee baat karte hain tu mein serious hojata hun tu kehtee hai kay mazaq kar rahi hun, naraz kyun hota hun

**Interviewer:** Dusray rishton kay saath kaisay hai?

**Interviewee:** Buss aisa hee hai.

**Interviewer:** Aap kehtay hain kay aap ko udaasi tu nahi hoti. Pareeshani hoti hai.

**Interviewee:** Buss insaan kabhi kabhar tu yeh sochta haina kay kya karein waghera. Itnay saal say theek nahi horahi hun. Jab mein musalay pe rab say baat karta hun, and I tried to make friends waghera

**Interviewer:** Hmm acha aap ne mention kya yeh violent hojatee hain. Tu kabhi aap ko inko control karne kay liye haath uthana parta hai? Kabhi inhon ne aap pe haath uthaya hai ghussay mein?

**Interviewee:** Yes. Beemari kay waqt yes. Pehele boht kartee theen ub nahi kartee

**Interviewer:** Aap kya kartay thay uswaqt?

**Interviewee:** Mein defend karleta tha apnay aap ko. Aur yeh kay…*pause* koshish kartee hai mujhe marne ki. Nahi marne deta. And I also slap her. Uskay baad mujhe taqleef hoti hai mera blood pressure high hojata hai. Aur jab mera blood pressure high hojata hai tu meri saari body shake karne leti but then I relax myself by taking my dose. But I control my diet and I take care of myself.

**Interviewer:** Waise aap ka din kaisa guzarta hai? Jab uthtay hain waghera?

**Interviewee:** Subah mein uthha hun jaldi, waise puri raat meri 2 2 ½ gantee baad aankh khultee hai. Utha eik sip paani piya aur cigarette pee lee phr wapis sojata hun. Mein sojatu hun, kuch parwa nahi karta hun. Aur jab mujhe uthna hota hai tu phr koi nahi sula sakta. Uth kay namaz waghera parhlee and dhoodh ka glass banaya, phr TV waghera dekh liya. Yeh 10 baje uthtee hain, yeh nashta banati hain aur dawai khateen hain

**Interviewer:** Aap inko dawai detey hain, ya yeh khud?

**Interviewee:** Nahi yeh khud hee khatee hain.

**Interviewer:** But aap check karte hain?

**Interviewee:** Haan mein nazar rakhta hun. Phr 10 30 tak hum saath nashta kartay hain, 11 baje tak mein na duh karkay, mein phr nikalta hun. Mein eik dost kay show room mein betha hun. Takey two teen ganthay mujhe milain to be away from everything. Kyuke agar ghar mein betha hun tu yeh karlein, woh karlein *laughs* yeh khud karsakein issliye mein nahi rehta. Mein nahi kehta kay inko taqleef nahi hoti lekin yeh isko bharati zyada hain. Uss apne problems ko. Mein phechlay eik saal sey inko neglect karta hun takey unko khud khayal ho kay mein khud take care karun.

**Interviewer:** sahi tu, aap apne farig waqt mein kya kartay hain?

**Interviewee:** Lunch waghera sham mein karleta hun kyunke mujhe dil nahi chahta jaldi khana khanay ka. Mein avoid karta hun. Eik hee meal leta hun 5 baje. Agar bhook lag gaye tu kha liya. Agar zyada kha liya tu so nahi sakta mein. Mein avoid karta hun raat ko. Mein chai khud bana leta hun, but yeh nahi banati. Kabhi meheney mein eik dafa agar mood mein hun tu banadiya. Waise normal… chalta hai issi tarah

**Interviewer:** Thoray say aur sawal hain. Aap ne kaha tha kay inki beemari ki waja say aap parh parh kar aaday doctor ban gaye hain tu aap ko kya lagta hai kay aap ko inki beemari kay barey mein kitna maloom hai? Kyunke hamari society mein itna pata nahi haina in beemarion kay barey mein

**Interviewee:** mein yeh keh sakta hun kay mein eik patient say baat karta hun, mujhe itna samajh mein ayega kay yeh beemaar hai kay nahi. Aur yahni mein psychiatrist tu nahi hun, lekin doctors khud bhee direct diagnose nahi karta. Insab mein eik thin line hai. Doctors medicine test karkay hee dekhtay hai. Mujhe andaza hojata hai kay yeh theek hai ya nahi hai. Mein iskay barey mein parh lya hai

**Interviewer:** Acha aur aap ko kya lagta hai kay aap ko kaheen aur say information miljaye? Aur bataye?

**Interviewee:** Knowledge is always acceptable. Jahan say bhi mile it’s always accepted. Knowledge ko accept karna boht mushkil hai

**Interviewer:** Acha aap ne mention kya kay boht sarey logo ne aap ko kaha kay divorce kyun nahi detey ho. Kabhi aap ne khud zaati tor pe socha hai kay nahi chordun aur divorce dedun?

**Interviewee:** *pause* Khayal aya hai lekin mein nahi de raha hun iskay du reasons hain. Kay merey bacho ko kon dekhaiga jab chotay thay. Aur uswaqt sab sey zyada yeh reason nahi dena ka yeh tha kay yeh zyada suffer kareyge apne maa baap kay yahan. Eik eik saal apne ami abbu kay ghar rahi hai, mein bhej chukka hun jaan booch kay. They never cared about her. Koi nahi bataya tha. Yeh recently baat hai, 2000 ki. Meiney kaha kay aap dekhlou issko, meri age bhar rahi thee, mein nahi dekh sakta tha zahir hee see baat hai, bacho ko bhee dekhna tha. *pause* Phr meiney iska phupa say baat ki, aur phr meri saas aye, iski choti bhen thee merey pass aye aur kaha kay mein leney aye hun. Meiney kaha shauq say lekey jayein. Tu bachi ne mujhe akey bataya kay yeh ami ka sara sona (gold) lekey ja rahi hain. Meri beti 10 saal kee hee thee, aur meiney kaha kay pura packet dedein, aur yahan say eik cheez nahi lekey jayein. Meiney kaha yeh inka haq hai. Meri saas mujhe sey boht lari. Meiney kaha kay mujhe majboor na karu kay mein batameezi karu, sharafat say lejao apni beti ko lekey. 1 ½ saal rehi wahan, but meri saas boht tung kartee thee usko. Merey susr nay faida uthaya aur uskay pass boht paisa tha, aur woh ayashni karta tha. Issko eik naya shauq lagaya, beti ko eik alag flat dilaya, shop bhe laga diya.

Yeh merey bacho ki maa hai buss.

**Interviewer:** Aap ko kya lagta hai kis soretahal mein talaaq leni chahye?

**Interviewee:** Uss soretahal mein leni chahye, dekhain there is nothing wrong in taking divorce, samaji tor pe. Yeh itnee bhee pagal nahi hain kay mein bilkul bhi na reh sakun inkay saath. Tu uskay saath zindagi guzarna mushkil hai jo bilkul pagal hai. Lekin uski yeh condition nahi hai. Isney tumhara saath diya jab tum beemar thay, jab tumhein bhukaar tha. She used to take care of me. When she is sick, I take care of her. Theek hai mujhe problem huwi, dunya walay hansey bhee merey upper, lekin unhon ne tu nahi support kya na, or na woh kareinge

**Interviewer:** Waise generally, kis soratehal mein talaaq leni chahye?

**Interviewee:** Jab tak kay *pause* yahni bilkul koi rasta nahi bana. When something can be done to survive tu karna chhaye. Kisi ghar mein bhe aap jayein tu eik dusray say tasal waghera chalta rehta hai. Saas bahu ki waja say husband wife mein apas mein hojata hai. Agar ma nahi chahtee aur merey betay ki biwi chahte hai. Usko avoid karna chhaye jitna insaan kar sakey. Agar 1 percent bhee chance hai tu insaan ko grab karna chahye.

**Interviewer:** Sahih. Tu aap ko kabhi bhee aisa laga hai kay aap ki biwi ki apni koi galtee hai beemari mein?

**Interviewee:** Nahi. Mein iskay bhai bhen ko bhi bata chukka hun and now they agree with me. Ubh yeh kehtay hain kay inkay ma baap ki galtee hai, mein bhee yehi kehtee hun

**Interviewer:** Aap ko kabhi aisa lagta hai kay aap inko theek karsaktey hain?

**Interviewee:** Nahi. Theek mein nahi karsatee. Mein assist karsakta hun. Mein maloomat karkay aur mein direct intervention say koi faida nahi hota tu mein doctor say assistance leta hun.

**Interviewer:** Acha aap ko lagta hai aap ko agar kaheen say madad ho tu aap ki pareshani kam hogi?

**Interviewee:** Nahi koi madad kar nahi sakta. Usko pata hee nahi hoga kay woh karey kya.

**Interviewer:** Eik purskoon khandaan kay liye konsi cheezain ahmiat rakhteen hain?

**Interviewee:** khandaan ka acha hona, ab kon kaisay keh sakta hai kay khandaan acha hoga ya bura

**Interviewer:** Matlab kuch cheezain aur qualities.

**Interviewee:** Dekhain..eik family mein sabr aur shukar aur ehsaas hona chahye, yeh teen cheezain boht zaruri hain. Aap ko eshaas hona chahye, sab ka khayal rakhna chahye.

**Interviewer:** Acha aap ko kya lagta hai kay khadaan important hai ya marriage important hai?

**Interviewee:** Pura khadaan important hai

**Interviewer:** Aap apni zindagi kaisee dekhtee hain?

**Interviewee:** Alhumdililah mujhe koi shikait nahi hai apne rab say. Jo manga hai woh diya hai, jo chalees saal phele nahi tha woh sab hai. Chalees saal pehle meri wife nahi theen, bachay nahi thay, merey damad nahi dein, mein ubhi dada nahi bana, tu buss Allah ki raza hai. Bachion kay koi problems hotay hain tu mein inko samjhatu hun aur kehta hun kay aurat ko hee dekhna hota hai. Aurat ghar sambhaltee hai. Yeh mera concept hai.

**Interviewer:** Acha aap ne marital counseling kay barein mein kya khayal hai?

**Interviewee:** Hmm, counselors counsel tu kareinge but issmein boht important hai language, environment aur khandaan, woh kis tarah say sochta hai. Mian biwi ki individual cheezon ko samjhna hai aur phr unkay khandaan ko samajhna hai. Agar koi family member koi samjhanay bethay tu yeh zyada acha hai. Zyada behtar raheyga, Unka zyada already pata hoga. Counseling ka matlab yeh hai kisi seniors kay saath beth kay discuss karein. Discuss karein aur fight na karein.

**Interviewer:** acha koi religion ka influence hai inki beemari ka?

**Interviewee:** Shuru shuru mein meiney yeh samjha tha. Meiney kaafi parha tu jab meri knowledge bhari tu mein nahi kehta. Mein jinns pe believe karta hun, good jin hotay hain aur buray bhee hotay hain. Mein nahi believe karta kay inko jinn charh gaya hai. We have to control and contain it because it’s an illness. Mujhe Allah himat dein aur Allah isko sahet dey.

**Interviewer:**

**Interviewee:**

**Interviewer:**

**Interviewee:**

**Interviewer:**

**Interviewee:**

**Interviewer:**

**Interviewee:**

**Interviewer:**

**Interviewee:**

**Interviewer:**

**Interviewee:**

**Interviewer:**

**Interviewee:**

**Interviewer:**

**Interviewee:**

**Interviewer:**

**Interviewee:**

**Interviewer:**

**Interviewee:**

**Interviewer:**

**Interviewee:**

**Interviewer:**

**Interviewee:**

**Interviewer:**

**Interviewee:**

**Interviewer:**

**Interviewee:**

**Interviewer:**

**Interviewee:**
