# Supplementary material for: Exploring effects of severe mental illnesses on marriages: A qualitative study from Karachi, Pakistan
Source: PLOS Glob Public Health. 2025 Dec 23;5(12):e0005652. doi: 10.1371/journal.pgph.0005652 (PMC12725543; doi:10.1371/journal.pgph.0005652)
Supplement: S1 Data — (ZIP) [file pgph.0005652.s001.zip › Transcriptions/Case 1 Transcripts/C1-17.docx]

**Case 1**

**Psychiatric Illness: Bipolar Disorder**

**Inpatient**

**Interviewer:** We will be recording this interview so I hope you’re fine with that.

*fills out the demographic form* (only earning member, and live in a nuclear setting, with two children)

**Interviewer:** How long have you been married for?

**Interviewee:** Since 2007 years

**Interviewer:** Okay and how long has she been ill for? Duration of illness

**Interviewee:** This is the first time that this episode has happened. We have never seen anything like this. Uhh just want to exclude the psychiatric issue. Everything is just excluded. She is not a psyche patient. I would say that she has a lose temper. So I want to take everything into account so that she gets proper treatment, but otherwise there is no psychiatric illness. This duration …illness is like last one week. This episode is the first time and when you’re married, such episodes do happen, but they are generally very small.

**Interviewer:** But is she currently on any treatment, like anti-depressants?

**Interviewee:** Yes, they have kept her on treatment but that is to relax her.

**Interviewer:** So previously she was not seeking any treatment?

**Interviewee:** Exactly

**Interviewer:** And so you did seek help from a psychiatrist?

**Interviewee:** This is the first time

**Interviewer:** Okay what have the doctors told you? Have they said that there is any diagnosis or are there just anger spells?

**Interviewee:** They have just told me that they are keeping her under observation right now. Maybe I have asked them to fix a meeting with this consultant or phr mujhe we will seek what is the actual reason. I still have to meet the consultant.

**Interviewer:** Who is the doctor assigned to your wife’s case?

**Interviewee:** I don’t know..ummm… I am not sure

**Interviewer:** Alright so have you told your parents about this current episode?

**Interviewee:** Yes my parents have been with me. They are in Peshawar. And my mother is here right now.

**Interviewer:** And what was her reaction?

**Interviewee:** Buss she keeps on saying that my wife should just relax and just get her to the hospital. We calmed my wife down and got her to the hospital. Whether it’s a medical issue or psychiatric issue or any issue that has to be treated.

**Interviewer:** All right, so if you don’t mind telling me, can you tell me what triggered this episode?

**Interviewee:** Yeah sure, issmein aisa tha, thora mein aap ko background batata hun. Yeh du bheney hain aur du bhai hain. So my elder brother is with her elder sister and she is with me. Same day hamari shaadi huwi and since then her sister hasn’t had her babies. Kuch dunu say she was like.. bachay nahi hai ussko and merey walid alag rehtay hain.. and blah blah. And then she couldn’t sleep. So I asked her that if she cannot sleep, I will get her a tablet. Neend puri hojayegi tu depression theek hojayega. But she was like no I won’t take any drugs. And then after this it started. And after that, yeh issue thora pheechay raih gaya and it kept on starting kay tum ne yeh kaha tha aur tum ne yeh kyat ha.

**Interviewer:** Yes, so a normal couple fight?

**Interviewee:** Yes and phr woh trigger hotay hotay unhein ne boht zyada lelya aur phr matlab ussney aisee harqat----- aisee batein shuru kardeen. Matlab puri raat eik hee baat ko repeat karna. Subah hotay hee asmaan ko dekhna. Freezer say paani nikal ke bacho ke uper daalna.

**Interviewer:** And yeh kabhi bhee pehel nahi huwa?

**Interviewee:** No this is the first time

**Interviewer:** There was nothing like this ever before?

**Interviewee:** Nope. She used to fight normally. That was definitely there.

**Interviewer:** All right, so she has been admitted since a week?

**Interviewee:** No she was admitted 3 days back but the whole issue was happening since a week.

**Interviewer:** Okay did anyone recommend you to come to the doctor or did you decide it yourself?

**Interviewee:** dekhain merey eik bhai …Inki walda say meri baat huwi. My mother in law so she told me to consult a psychiatrist. Ahh thora sa kuch arsa phele larai huwi thee so she went to her mom. Theek hai. Wahan per she was there 2-3 months and she was mentally disturbed. Okay and when the issue finished so she was ready to come but her parents were not willing to send her. Theek hai. They were reluctant. So I talked to her mother. And I asked her why because she was calling me 3 times a day that she wants to come back. Tu ussey woh kehteen hain kay jo wahan per time guzara hai ussey yeh mentally disturbed hain. Yeh basically.. eik saal hosaka iiss event ko.

**Interviewer:** All right so between that event and this episode, nothing at all happened, everything was normal?

**Interviewee:** Yes there were small fights but other than that nothing.

**Interviewer:** So since the past one week, aap kaafi pareeshan hongay. So do you feel a lot of hassle etc?

**Interviewee:** Exactly. In the sense that since the past two weeks, I was very busy because there was a foreign delegation at work. So I was unable to give her time. Subah 7 baje nikal jaata tha. And raat ko 10 baje ghar aata tha. So yeh jo saara issue hua hai basically she was alone. She couldn’t share it with anyone. I was not there. Ghar mein bacho ki school say chutyian theen so unhon ne pe unko pareshaan kya and she was already stressed tu woh cheezain karte karte yeh saara hogaya. This is the threshold jis pe yeh achuki hain.

**Interviewer:** Do you feel very tired?

**Interviewee:** Yes yes obviously. Today I couldn’t go to office and the kids are of course with me. Ami hain but again one should be with them. Ya ma ho ya baap ho. Obviously pareeshani hoti hai

**Interviewer:** Do you feel frustrated?

**Interviewee:** Not exactly frustrated because I know that this episode ..I am not frustrated but I am very worried, I would say. Frustrated is not the right word but worried, yes.

**Interviewer:** okay and have you told anyone else in your family about your wife’s condition?

**Interviewee:** Merey dekhain yeh jo ubhi sab saara hua hai tu bacho ko tu meiney inn saari cheezon say dur rakha hai. Kyunke agar yeh cheezain dekhaingay tu obviously negative effect hoga. Aur woh unkay zehn mein ajata hai. Tu merey dost hain office colleague hain. Unko meiney yahan bulaya tha aur bachay unkay pass chorain. Jab mein yahan aata hun inkay pass tu bacho ko colleague kay pass chordeta hun. When I am going back home, so I pick them up.

**Interviewer:** And this colleague that you’re talking about, has he questioned you about your wife’s condition?

**Interviewee:** Yes obviously. They inquired kay kya hua.

**Interviewer:** Okay and what’s your response?

**Interviewee:** Well he is obviously a very good friend of mine so I have told him things. Unko thora sa phele see hee pata tha.

**Interviewer:** How do you feel that your family dynamics have changed?

**Interviewee:** A lot.. I would say a lot..a lot in the sense that batein jo inhun ne kardein aur jo meine kardein, yes we will try to overcome but still woh cheezain aap kay black box mein rehteen hain. Maybe not today after some days it might come out. I hope that whatever this is, it was just one stress episode and not any psychiatric illness. Stress tu khatam hojayega but jo psychiatric issue hai will linger on. I wish and I hope inshAllah that things would change.

**Interviewer:** Okay and when you found out that you might have to take her to a psychiatrist so what was your reaction?

**Interviewee:** Ahh dekhain isssay pehele jab yeh issue hoye thay jab yeh phele gaye theen tu usspe bhi inki walda ne kaha tha kay aap consult karein eik psychiatrist kay pass. Issko apne uper control nahi rehta aur yeh boht ghussa kartee hai tu uss time pe mujhe zyada hua tha. I got angry at her kay aap ubh bata raheen hain kay I have to consult a psychiatrist. Jab yeh ghussa mein hongee aur mein psychiatrist kay pass lekey jaonga tu yeh aur ghussa hongee. Jab yeh kabhi calm hongee tu haan tab banda baat karsakta hai kay yes we can consult a psychiatrist. Uss time pe I was a bit..it was a bit weird. But this time I knew I had to consult a psychiatrist. Jo yeh episode hogaya things would not settle.

**Interviewer:** Do you feel that your relationship with others have been impacted due to her illness?

**Interviewee:** Yes..uhhh… other people in the sense apne jo in-laws hain merey woh keh rahay hain kay zarur koi stresser hua hoga jo trigger kya. But it is very easy to judge, and apne comments pass kardiye. Ussay obviously hogaya hoga but ..as far as my family is concerned, they are very concerned. Isliye meri ami bhee agayein. My brother was like if things don’t settle…

**Interviewer:** Okay and how do you feel your relationship has changed after the illness?

**Interviewee:** I think I will try to be more close and I will have to give her more time.

**Interviewer:** Acha, I am sure you feel very stressed out and tired. So do you feel because of that you might have to consult a doctor?

**Interviewee:** You see, yehi cheez meiney apni ami say discuss ki hai that once things settle down, I will also see a psychiatrist. Theek hai kyunke you will never know your weak area if you don’t have another opinion. Tu yes I think meiney yeh already soch liya, I will also consult a psychiatrist. I want to see that whether meri taraf say koi aisee hurqat huwi ho jis say inki tabiat aur bigri ho.

**Interviewer:** Also, if you don’t mind me asking, is it getting financially taxing for you? Hospital is expensive.

**Interviewee:** No I don’t think so.

**Interviewer:** So you mentioned that she gets very angry. So does she scream?

**Interviewee:** Yes screams a lot. She becomes very sensitive.

**Interviewer:** Has she ever hit the children or has she ever hit you?

**Interviewee:** I have hit her kyunke she used to get very violent. Bacho ko ubhi tak ussney kuch nahi kya BUT this episode mein yeh jo saara hua issmein bacho ko bhee thora kya hai. Kabhi bacho ko nahi maara. She is a very concerned mother.

**Interviewer:** Okay and I also want to know how your family routine has changed? I mean, I know it has only been one week but can you tell me that before this episode, how was your day like and after this episode, how was your day like?

**Interviewee:** I am now a working husband *laughs* that has what has changed

**Interviewer:** Okay, so you’re taking care of the kids and you’re taking care of the house. Are you going to office?

**Interviewee:** I have taken an off

**Interviewer:** And are your work people understanding?

**Interviewee:** I have not informed, I have just told them that I am not feeling well.

**Interviewer:** All right, and what additional responsibilities have you taken after this episode?

**Interviewee:** Even I have to take my kids to bathroom and I have to give them a shower. And change their clothes. I have to give them feeders. Because they are not close to my mother as such. My mother is taking care of the food and everything but rest I am doing all by myself. All the activities my wife was doing.

**Interviewer:** Generally, before this episode, what did you do in your leisure time?

**Interviewee:** I don’t think so I had any leisure time

**Interviewer:** Before that?

**Interviewee:** before that, you see, I am a workaholic kind of guy. So after I finish all my work, when I am home, so I try to spend my time with my kids. Watching baby TV or just playing with them or talking to them. But sometimes she complains, you have more …she says chordou TV dekhna, khabrein chordun, drama dekhna hai. Yeh tou hur normal wife karsaktee hai but rest leisure time was with my kids. Kabhi watching TV, Saturday and Sunday ko sindbad legaye. Almost every week, hum bahir khaana khaney jaatey hain, shopping pe jaatey hain.

**Interviewer:** Acha aap ne bataya that the doctors have not given you any diagnosis. But aap ne online kya inkay symptoms waghera kay barey mein parha?

**Interviewee:** Nahi dekhain, jo first doctor jo Dr. Ayesha then, jab inko meiney history de tu unhon ne kaha kay mujhe lag raha hai Bipolar Disorder, which is a mental disorder. I discussed this with my brother but he told me that let it be now, but let it be confirmed before. I will tell you later. Kay yeh hai kya. So I would say that I am more concerned that whatever comes first and then I will dig out more information. Kay kya hai. Whether I should consult any other doctor. But woh keh rahay hain kay eik cheez samney ajaye phr hum dekhaingay. Ubhi tak tu yeh keh rahay hain kay we are giving medications and that will relax her mind. Kyunke jab yeh last huwi thee tu yeh keh rahe theen kay mein teen din say soye nahi hun. Theek hai, she hadn’t sleep. Woh keh rahay hain kay hum phele inko thora normal pe le ayein. And then maybe..

**Interviewer:** Hmm, okay and what do you think of this diagnosis? I want to know about your subjective perspective? Regarding this situation.

**Interviewee:** Mein samajhta hun kay yeh eik glass hai jo paani se bharha tha and now its coming out. And she has no issue …she is not critical.. She has come to this position where she is now exploding. I don’t think so koi psychiatric issue hai. Inhon ne eik galtee ki hai aur she was trying to fix this mistake so usko kartey kartey yeh aur galtee mein agayein. Let’s say that kisi ne eik ilzam laga diya aur ussko prove karne kay liye inhon ne aur ilzaam laga deye. Uskay issues ka. Unko samajh nahi araha kay mein kya kar rahi hun. Eik normal banda tu samajh sakhta hai lekin jo kar raha hota hai woh soch raha hota hai kay mujhe prove karna hai. And usko prove karney kay liye, they think everything is right. So yeh mein samajhta hun baaqi I don’t think there is any other issue. Kyunke first jo mein..she was like this… She has slept now and neend puri huwi hai, tu unka attitude bilkul hee, pheley woh meri mother ko bhi naam say pukaar rahi theen, tu thora sa I would say, better hua hai.

**Interviewer:** acha kisi qism kay ilzaam lagaye thay unhon ne? Kis qism kay ilzam?

**Interviewee:** Han matlab wohi na kay tumne merey saath yeh kya tha. I was like isstarah nahi bolu. Yeh basically hua tha

**Interviewer:**  All right, aap ko kabhi lagta hai kay inki galtee hai kay inko yeh episode huwi hai, I mean, do you in any way feel that it is her fault to have the illness?

**Interviewee:** Yes, it is. Completely, completely. Mein tu yehi samjhta hua kay meiney yeh bhi bola kay koi galtee hai aur agar mein yeh samajhta hun tu …sukoon karlo and I am sorry merey say galtee hogaye. She was nahi ub tou nahi. It is 100 percent..100 percent

**Interviewer:** Acha and do you think you can fix her and that she will be fine?

**Interviewee:** I will at least try. Whole heartedly.

**Interviewer:** And you plan on staying in the marriage?

**Interviewee:** Exactly

**Interviewer:** And working it out

**Interviewee:** Right. I didn’t leave her when she had no baby. How could I leave her when she ..It’s out of the question.

**Interviewer:** So for you, is the marriage more important or the family as a whole is more important?

**Interviewee:** I didn’t get it

**Interviewer:** In the sense do you think that the relationship between you and your wife is more important or..the whole family, like your kids?

**Interviewee:** I think the whole family is important. Otherwise its useless.

**Interviewer:** aap ko friends and family ka support mil raha hai. Do you think it has made your burden less?

**Interviewee:** Well to some extent, yes, but jis pe guzarta hai uss hee ko pata chalta hai. Everybody is…like bara afsos horaha hai, bhen bhai, theek hai, bhai abbu sab, but again.. I am praying that everything goes back..

**Interviewer:** I would just like to ask, in what situation should a couple generally seek separation or divorce? Like generally and not related to your marriage obviously?

**Interviewee:** Jab compromise nahi hota mainly from the female side, then I would say…then I think things ..usmein phr issues ajatey hain. Yeh mera apna personal issue hai. Compromise dunu taraf say hona chahye but I would say the ratio jo hota hai woh larki ki taraf say zyada hona chahye. Nahi hoga then…

**Interviewer:** Acha, how do you see your future? Objectively in a year or so with your wife?

**Interviewee:** She will be maybe expecting another baby. She is my family.

**Interviewer:** Do you feel that there is any kind of influence of religion in her condition?

**Interviewee:** Religion

**Interviewer:** Jo episode hua hai do you feel kay kuch log boltay hain..

**Interviewee:** Merey dost ne eik boht ache baat kahi kay mein inn cheezo pe believe nahi karta ..taweez pe waghera, but I think you should consult a scholar or something or hai nahi, lekin when you are eliminating so many things tu isko bhi eliminate kardu. Sometimes insaan kay zehen mein aatee hain yeh cheezain but I don’t think so.

**Interviewer:** All right, right now your wife is in the hospital but when she comes back home

**Interviewee:** Inshallah

**Interviewer:** Yes inshallah then do you think you would consider marital counseling to sustain your marriage?

**Interviewee:** I don’t think so. I think we are mature enough that inko inn cheezo ki zarurat na pare. I don’t think so. Counseling kay liye we can go to parents.

**Interviewer:** To raise a happy and pursukoon family, what do you think are some of the essential ingredients?

**Interviewee:** Respect..to have respect and to give respect and it should be with love and care. But respect is the first thing.

**Interviewer:** I think it’s very obviously pre-mature, but just asking, so for example, if this is a psychiatric illness, how would you deal with it?

**Interviewee:** If it is a psychiatric illness and if it is diagnosed, then there will be medications and we will stick to the medications.

**Interviewer:** Do you think there is a cure to psychiatric illness?

**Interviewee:** I think science has ..

**Interviewer:** Do you believe in psychiatric illnesses?

**Interviewee:** yes

**Interviewer:** Do you feel that it is probable?

**Interviewee:** yes

**Interviewer:** And will you take her to a scholar, like your friend suggested?

**Interviewee:** No, I will consult a psychiatrist.

**Interviewer:** And all right, you answered that you would stay in the marriage but right now, it’s too premature. This is just because we usually interview people who have been going through this for quite some time, and you have been going through this for a week. So for example, if she does have a psychiatric illness, do you still see yourself staying with her?

**Interviewee:** I will try. I will try my best to last the marriage.

**Interviewer:** Thank you so much for answering the questions for us. I hope your wife feels better.

**Interviewee:** Thank you.

**Interview Ends**
