# Supplementary material for: Exploring effects of severe mental illnesses on marriages: A qualitative study from Karachi, Pakistan
Source: PLOS Glob Public Health. 2025 Dec 23;5(12):e0005652. doi: 10.1371/journal.pgph.0005652 (PMC12725543; doi:10.1371/journal.pgph.0005652)
Supplement: S1 Data — (ZIP) [file pgph.0005652.s001.zip › Transcriptions/Case 1 Transcripts/C1-18.docx]

**Case 1**

She did not allow the interview to be recorded. Her husband had Schizophrenia. The woman spoke in Urdu but the interviewer wrote down the details in English; hence the interview is in English only.

**Interviewer:** How long has your spouse been mentally ill for?

**Interviewee:** He has been ill since 2004. However, I always felt that he had some problems. This is because he used to beat me up. And he was working at that time and he used to threaten that I will leave the job. He has seen a lot in his life. All of his friends went ahead and did great things, but he did not even specialize after doing MBBS. So he was always disappointed with his life. He also tried to do CSS but he failed two times. He wanted to specialize in pathology but he was unable to do so, because his family did not support him. And then we got married and we had one child. At that time, he was earning only Rs. 6000. I was a primary teacher as well. I transferred from a village in Sindh to Mirpurkhas. My salary was also low. My father-in-law also made me a junior teacher. Initially, he was earning so low that I had to support myself and my child. And I am still putting up with him until now. How will I leave him? He is my husband.

**Interviewer:** How did you find out about the mental illness?

**Interviewee:** In 200, he tried to commit suicide. We took him to a psychiatrist known as Dr. Iqbal Afridi. He took medications and was fine for a week. But he decreased the dosage himself without even telling anyone. The symptoms obviously returned and then we went back to the doctor. After that, we took him to another doctor, but he was injected with something and he became extremely weak. Eventually we came to Dr. Murad Musa.

My father in law thought that I had done jadoo or black magic on him. He said that “meiney wazifa parh diya hai aur woh ulta hogaya hai”. I said that fine I have done it and let’s do ‘rohani ilaj’. But it did not prove to be effective, that’s why we also come to the doctor.

**Interviewer:** Okay what was your reaction to the illness?

**Interviewee:** I didn’t have much of a reaction. We always fought a lot. I was under a lot of tension and stress. I thought “ub kya hoga, jhelna hee pareyga.”

**Interviewer:** Were your parents aware of the spouse mental illness?

**Interviewee:** Yes, they were but now they have passed

**Interviewer:** What’s your support system? Do you have any kind of help?

**Interviewee:** Yes, my brother in law helped us financially initially, but when my husband’s salary increased, he stopped helping and we also stopped asking him for help.

**Interviewer:** Does it get quite stressful managing your husband?

**Interviewee:** Yes, it gets very stressful “boht pareshani hoti hai”. He has no interest in me. He does not even talk and honestly now even I don’t feel like talking. Whatever I say to him, he takes wrong meaning for it. We have zero social interaction.

**Interviewer:** And what is most frustrating? Coming to the doctor etc?

**Interviewee:** Yes, it is very stressful. Because we live in Mirpurkhas and coming in the bus is a big issue, especially because he often gets violent on the bus and initially, he used to abuse me. Also he has a urine problem, so he has to go the bathroom often and the bus does not stop for everyone. This becomes a hassle and a source of embarrassment because all the passengers look at us and laugh at us. Even though his brothers have cars, they do not help us out in this way. They can help us with this because taking the bus becomes an issue. I also have arthritis problem so I cannot keep coming again and again.

**Interviewer:** Does your support help the patient?

**Interviewee:** yes it does

**Interviewer:** Okay and does your child help?

**Interviewee:** Yes now he has come to Karachi for his studies. He is doing ACCA and I deliberately sent him away to Karachi because I did not want him to become mentally ill, as well living with his father. In fact, he has been quite helpful throughout.

**Interviewer:** All right, do you guys go out often to socialize?

**Interviewee:** Yes sometimes we do. But mostly, we are at home. I am also scared to go out and socialize with others because he fights with everyone. I am scared that his mood will get worse and he would react negatively. At my nephew’s wedding, he beat my nephew. However, my sisters live in Hyderabad and because he gets love from there and attention, he often wants to go over there. However, he does not like coming to Karachi because his brothers have not helped at all

**Interviewer:** What was your child’s reaction to the illness?

**Interviewee:** He used to get very disturbed but he never expressed and kept it himself. His cousins used to say that “tumhara baap pagal hain”

**Interviewer:** All right who encouraged in seeking help?

**Interviewee:** My husband’s brother told me to come to the doctor.

**Interviewer:** All right how has your relationship changed since the onset of the illness?

**Interviewee:** It has changed quite a lot. We do not have any sex life and honestly even my sex drive has decreased. It does not matter to me anymore

**Interviewer:** Do you think that your relationship with others has changed after the onset of the illness?

**Interviewee:** Yes I feel extremely reluctant to go out and also because I feel that “log phuchay gain” about the illness.

**Interviewer:** has the mental illness of your spouse led to any mental health problems of your own?

**Interviewee:** Yes, I have blood pressure problems. I get anxious and depressed.

**Interviewer:** Are there any kind of financial problems?

**Interviewee:** Well, there used to be problems before when he was earning only Rs. 6000 but now we are doing well.

**Interviewer:** All right, you mentioned that he gets violent. Does he get violent to the extent that he hits you?

**Interviewee:** he used to hit me before but now he does not. However, he only gets angry now when I also say something to me. I also get hyper.

**Interviewer:** All right, out of your normal everyday routine, how much of your time do you devote to take care of your husband?

**Interviewee:** I do not have to take care of him a lot because he is mostly sleeping. He has also started praying a lot these days so that also keeps him occupied.

**Interviewer:** Okay what additional responsibilities have you taken after the illness of your spouse?

**Interviewee:** I have always done the grocery shopping and went to submit the bills and going to the banks. Now he goes sometimes after I send him saying that I am not feeling well.

**Interviewer:** what do you do in your leisure time?

**Interviewee:** I either watch television or pray. We do not go out a lot. I also do not want him to go out with friends because he gets upset. He gets depressed because they have gone ahead in their lives and he has not been able to.

**Interviewer:** Okay and have you ever been suggested about divorce or separation by friends or family?

**Interviewee:** No. I did run away once, but my child stopped me. He told me not to go. Now I think that there is no point “hum kahan jayeinge ub. Iskay saath nibhana he pareyga”. I have to bring him up like a child so it gets very difficult.

**Interviewer:** Okay, and have you ever thought of divorce?

**Interviewee:** No, I have not.

**Interviewer:** Okay, and in what circumstances do you think a couple should get divorced?

**Interviewee:** I don’t think they should because the children suffer. I think “nibhana chahye. Jaisay bhee hai, shauhar hai” And I say this to my colleagues who are complaining about their husbands. I tell them that I have suffered so much yet I am still living with them. He never even gave me his salary so I had to take care of my expenses along with my son’s.

**Interviewer:** All right, what kind of support would have helped ease your burden?

**Interviewee:** If I had gotten support from his family, it would have helped.

**Interviewer:** Okay and what do you think are some of the reasons that you continue to stay married to him?

**Interviewee:** I am married to him because of my child.

**Interviewer:** How do you see your future?

**Interviewee:** “kharaab hee hai. Allah say bus umeed hai. Bhurapa kya gul khilayega, kya pata”

**Interviewer:** Okay and do you feel you know enough about the illness?

**Interviewee:** yes. I know it is an illness and it is not magic.

**Interviewer:** Do you feel it is your spouse’s fault to have the illness?

**Interviewee:** No. but I think he would not have gotten ill in the first place if he would have support from his family

**Interviewer:** Do you think the marriage is more important or the family as a whole?

**Interviewee:** Family because if family is doing well, then the marriage gets support itself.

***Interview Ends***
