# Supplementary material for: Exploring effects of severe mental illnesses on marriages: A qualitative study from Karachi, Pakistan
Source: PLOS Glob Public Health. 2025 Dec 23;5(12):e0005652. doi: 10.1371/journal.pgph.0005652 (PMC12725543; doi:10.1371/journal.pgph.0005652)
Supplement: S1 Data — (ZIP) [file pgph.0005652.s001.zip › Transcriptions/Case 1 Transcripts/C1-19.docx]

**Spouse**

**Case 1**

***Transcribed from 01:05.***

**Interviewer: Takreeban aap ki umer kitni hogi?**

Interviewee: Meri umer hogi 59.

**Interviewer: Aur aap ne parha hua kahan tak hai?**

Interviewee: *Inaudible, 1:15-1:18.*

**Interviewer: Aur abhi kaam karte hain aap?**

Interviewee: Main karobar karta hun.

**Interviewer: Aap ka apna hai?**

Interviewee: Jee.

**Interviewer: Aur kab se kaam kar rahay hain aap, takreeban?**

Interviewee: Takreeban 25saal.

**Interviewer: Aur aap ki shaadi ko kitna arsa hua hai?**

Interviewee: Do saal pehle, 27 saal, yaani ke karobar se do saal pehle, 27 saal.

**Interviewer: Aur abhi aap log joint family system mein rehte hain ya aap dono rehte hain?**

Interviewee: Building ek hai, uss mein hum 3 familiyan hain.

**Interviewer: Acha joint ki tarhan hai?**

Interviewee: Lekin separate separate hain. Separate, separate hain, khaana peena.

**Interviewer: Nuclear, theek hai. Aur takreeban aap ke ghar mein kitne log rehte hongay?**

Interviewee: Mere ghar mein mere teen bachay aur do hum, paanch.

**Interviewer: Paanch, aur kamanay walay aap ek hain?**

Interviewee: Nahi, meri betiyan hain, woh bhi job karti hain.

**Interviewer: Kitney log ghar mein kamanay walay hongay?**

Interviewee: 3.

**Interviewer: Agar batana pasand karengay toh maheenay mein takreeban kitna jaisay amdani ya kitna kama letayhongay?**

Interviewee: Wese amdani ka koi nahi hai hisaab, kharchay kitnay hain, bachon ki taleem hai, *inaudible [2:45-2:51]*doctor ki feezein, dawai waghera *inaudible [2:43-2:46].*

**Interviewer: Aur aap ke bachon mein say betiyan kitni hain aur betay kitnay hain?**

Interviewee: 3 betiyan, 1 beta.

**Interviewer: Theek hai, 4 bachay hain aapkay?**

Interviewee: Ek married hai, beta meri bari wali.

**Interviewer: Un kay walid sahb ne parha hua hai? Unki kya education hai?**

Interviewee: Nahi.

**Interviewer: Aur aap ki apni koi, ya aap ke apnay khandan mein kisi qism ke nafsiyat ke koi problems?**

Interviewee: In ki walda ko hai *inaudible [3:30-3:36]*

**Interviewer: Theek hai, walda thay aur nana thay.**

Interviewee: Jee.

**Interviewer: Aur inki jo bemari hai, takreeban kitna arsa hai?**

Interviewee: Yeh pehle bemari hui thee, phir beech mein gap aya tha, theek hogaye theen.

**Interviewer: Aur yeh shaadi ke baad sab tha?**

Interviewee: Yeh shaadi ke baad tha.

**Interviewer: Shaadi ke baad?**

Interviewee: Shaadi ke baad. Shaadi se pehle kamzor theen, kafi lekin phir shaadi ke baad, *inaudible [4:08-4:12]*phir ek dum inko depression ka attack hua, phir mukhtalif haspatalon mein, roohani ilaaj bhi karwate rahay, toh takreeban beech mein, 8-10 saal jo hai bilkul continuous medicine use karti raheen, phir medicine chor di ke bhai ab toh mein set hogayee hun, phir sara ghar ka kaam karti hun, neend bhi aati hai, *inaudible [4:37-4:40],* phir uskay baad dobara inhein attack hua. 3 mah.

**Interviewer: Toh abhi jo attack hua, woh3 maheenay pehlay hua tha?**

Interviewee: Jee.

**Interviewer: Theek hai. Aur yeh jo dawaiyon pe hain takreeban kitnay arsay se dawaiyon pe hain?**

Interviewee: Takreeban 2 saal.

**Interviewer: Aur kisikism ka jese nasha ya tambakoo?**

Interviewee: Nahi.

**Interviewer: Aur koi kuch batana chaheingay? Kisi aur kism keh koi aur problems jese koi pareshani, health ke havalayse ya parhai ki bachon ki ya financial?**

Interviewee: Nahi bas yehi pareshaniyan hain. Yeh theek hojayein toh saari pareshaaniyan khatam.

**Interviewer: Acha matlab ghaliban shaadi se pehle unko depression waghera ka koi problem nahi tha na?**

Interviewee: Nahi.

**Interviewer: Theek, shaadi ke baad he diagnosis hua? Acha theek hai. Aur aap unko khud le kay gaye thay doctor ke paas?**

Interviewee: Jee,

**Interviewer: Acha aur ap ke waldain hayat hain abhi?**

Interviewee: Nahi, lekin uss waqt hayat thay.

**Interviewer: Acha toh un ko pata tha in ke bemari ke baray mein? Aur un ke waldain ko pata tha? Aap ki wife ke?**

Interviewee: Jee.

**Interviewer: Aur un ki taraf se koi support waghera milta tha aap ko kisi kism ka?**

Interviewee: Nahi, yeh hamaray boht kareebi rishtay hain.

**Interviewer: Aap rishtedaar hain?**

Interviewee: Jee, meri phuphi zaat behn ki beti hain.

**Interviewer: Acha, apko kisi kism ki help hai? Aap ki wife ki take care karnay mein? Aap toh zahir si baat hai kartayhongay, phir ap ki betiyan, beta waghera madad kartay hain apko?**

Interviewee: Bilkul.

**Interviewer: Phir kisi aur kism ki madad? Bhai ke khandan? Bhai behn hogaye?**

Interviewee: *Inaudible [6:35-6:50]*

**Interviewer: Aap ko kis kism ki pareshani ka samna karna parta hai inki beemariyon ki waja se? Agar aap batanachahein toh?**

Interviewee: Pareshani bas yehi hai, dekho, ghar ka saara nizaam jo hai na, bigar jata hai. Yeh jo kar sakti hain ghar mein, koi aur nahi kar sakta, mein nahi kar sakta. Meri betiyan woh (interviewee pauses, emotional)

**Interviewer: Agar aap nahi jawab dena chahtay toh theek hai. Paani dedun apko? (Some noise due to technicalproblems, patient emotional)**

**[Interview resumes after pause] Aap jese sochtay hain ke kisi qism ki madad miljaye jis se yeh theekhon? Konsi cheez se behtar hongi?**

Interviewee: *Inaudible [8:08-8:13]*

**Interviewer: Inkay ilaaj mein koi behtari aye hai pehlay se?**

Interviewee: Jee, pehlay se boht behtar hain. Pehlay jab ayee theen, tab bhi doctor ne dekha tha. Unhon ne khud bhi mehsoos kiya aur hum ne bhi mehsoos kiya.Meri bhi esi halat iss liye hojati hai keh boht arsa hogaya inn halaat se guzartay huay.*Inaudible [8:39-8:42]*

**Interviewer: Yehi hum samajhtay hain, hum chah rahay hain ke jo khayal rakhnay walay hotay hain, unkay kya masaylhain, un ko sahi kism ki madad mil sakay, kisi bhi *way* mein, yeh hamari *research* ka *purpose* hai *basically.* Matlab, aap kafi, unki waja se routine upset hojata hoga jab unki tabiyat kharab hoti hogi?Toh aap ko zahir si baat hai pareshani hoti hogi aur matlab?**

Interviewee: Meri apni zaati pareshani ho toh uska koi masla nahi hai. Bachay upset hojatay hain, ghar ka poora nizaam jo hai, woh upset hojata hai, bas yeh pareshani hai.

**Interviewer: Aur doctoron ke paas lana, lejana aap khudi kartay hain?**

Interviewee: Jee.

**Interviewer: Toh aap ko kya lagta hai, aap jis kism ki itni madad kartay hain, iss se unko help mil jati hai? Jo bhi inkoproblem horahi hai, jitna aap kar sakte hain-**

Interviewee: Zahir hai, woh koi bhi hai, itna toh samajhta he hoga, keh itna taawun mere sath horaha hai.*Inaudible [9:47-9:52]*

**Interviewer: Aap ko lagta hai ke unka faida horaha hai, aap jo inka itna khayal rakhtay hain aur aap lekay atay hain.**

Interviewee: Agar hum nahi karengay yeh saari baatein unkay saath, toh unka theek hona boht mushkil hojayega.

**Interviewer: Aur ghar mein bhi jese unka kaun, bachay matlab ap ke baray hongay MashaAllah, toh woh khayal wayalrakh letay hongay ammi ka?**

Interviewee: Jee unka, khanay peenay ka, inkay aram ka, *inaudible [10:16-10:20],* kabhi esa hamara esa mahol nahi hai ke matlab hum is ki kisi zaroorat ko *inaudible [10:24-10:30].*

**Interviewer: Aap batayein gay, unka jab active phase hota hai, jab ese ap ko lagta hai ke yeh beemar par rahi hain,toh uss mein unkay kya, matlab kis tarah behave karti hain? Kya kefiyat hoti hai? Kya kehti hain?**

Interviewee: Bas yeh kefiyat jo hai na, ek dum se nahi ati. Yeh slow slow mein*.* Pehle inki tabiyat mein bechaini si paida hoti hai, jis ka kabhi kabhi, haftay mein ek baar, do baar esa hojata hai, in keh khanay ka nizam jo hai, woh kharab hojata hai, kabhi inki androoni jo *Inaudible [11:00-11:03]*hai uss mein shikayat hoti hai. Yeh tamam baatein apnay taur pe yeh batati rehti hain, phir yeh hai keh hum zaroori samajhtay hain ke inko forun doctor ke paas lejayein, ab ye hiss baar bhi ese he hua tha, lekin yeh hai ke iss mein mareez khud beemar hai, toh khud bhi ziddi kism ka hojata hai. Sarrialkism ka.

**Interviewer: Esa kabhi hua hai keh inko awazaein sunayee deen ho?**

Interviewee: Yehi toh saari inki beemari hai, depression mein yehi kuch hota hai, awazaein sunayee deti hain, mukhtalif kism ki aur yeh woh khauf sa lagta hai, agar koi cheez nahi lagti, kisi se milna jhula napasand ho. Iss kism ki boht saari baatein hoti hain, phir unkay khanay peenay, uthnay bethnay, chalnay phirnaysab cheezon ke andar jo hai na woh tabdeeli ajati hai.

**Interviewer: Phir aap log bahar jatay hain ghoomnay phirnay? Jese kitna maheenay mein?**

Interviewee: Hamari MashaAllah relationship itni hai keh jo mujhay woh poochti hain “Aaj kisi ka kya horaha hai? Aaj kisi ka kya horaha hai? Aaj yahan jana hai, aaj wahan jana hai.”

**Interviewer: Toh aap inko lekay jatay hain? Yeh jaati hain?**

Interviewee: Haan jab meri zaroorat hoti hai, mein bhi saath jata hoon, jab meri zaroorat nahi hoti, toh yeh apni bachiyon ke saath, ya khud chali jaati hain.

**Interviewer: Log beemari ke hawalay se poochtay hain? Matlab aap ko pata hai na hamari society mein itna nafsiyatibeemari ko kaha jatay hai keh nahi yeh toh pagal hai. Esa kabhi?**

Interviewee: Nahi, pagal toh mareez ko kehna he nahi chahiyay.

**Interviewer: Nahi log boltay hain, koi aap ko kabhi bola hai kisi ne? Aur jab log aap se poochtay hain toh aap unkosahi batatay hain? Keh yeh masla hai ya bas keh detay hain keh “nahi beemar hain?”**

Interviewee: Woh khud bhi samajhtay hain iss baat ko.

**Interviewer: Nahi kyunki hamare mein zyada tar log nahi samajhtay iss baat ko.**

Interviewee: Jo matlab ke inn cheezon ke mutaliq ilm nahi rakhtay, woh iss kism ki baat karte hain.

**Interviewer: Aur yeh sab keh jinn char gaya ya?**

Interviewee: Yeh matlab log apna apna ek, matlab achi baat hai, roohani ilaaj bhi karwana achi baat hai, abhi doctor saab ke saath mulaqat hui toh unhon ne khud yehi advise kiya keh aap namaz parhatay rahain, *ayat al kursi* parhatay rahain, *soorah yasin* parhatayrahain, theek yeh hai keh hum bhi aap ko dawaiyon se help kar rahay hain, lekin inn cheezon se bhi aap ko boht madad milaygi.

**Interviewer: Acha, toh aap ko kya lagta hai keh pehlay kuch arsay tak toh apki wife sahi raheen theen na, shaadi kekuch arsay tak? Kitnay saal?**

Interviewee: Takreeban 4 bachon ki pedaaish tak jo hai na yeh sahi theen.

**Interviewer: Toh aap ko kabhi nahi laga tha keh yeh-**

Interviewee: Nahi.

**Interviewer: Toh phir kiss tarah aap ki life change hogaye ke matlab you know zahir si baat hai khandani zindagi pefarq parta hai keh itnay saal MashaAllah rahay, woh bhi sahi raheen, achanak se unki tabiyat bigarrgayee, matlab kiss tarah se life change hui aapki?**

Interviewee: Life jo hai hamari itni high level pe life hoti nahi hai hum logon ki. Ek middle class life hai. Hamari toh logon ke beech *Inaudible [14:12-14:16]*aur life bhi itni nahi hoti keh jo hum isko-

**Interviewer: Lekin agar ghar ka zaati-**

Interviewee: Haan ghar ka mahol jo hai woh change hojata hai kyunke inn ke honay se jo hai na boht ghar mein ronak hai, agar yeh nahi hain na, toh ghar ki ronak jo hai woh khatam. Baap betiyon mein *Inaudible [14:34-14:36]*nahi karsakta hai jo maa kar sakti hai. Betiyan baap se nahi kar sakteen jo maa se kar sakteen hain. Iss kism ki boht saari baatein hain. Ab pehlay meri bachiyan jaati theen shopping ke liyay, walda ko lejateen theen, aur agar yeh nahi jateen toh mujhay unke saath jaana parta hai.

**Interviewer: Toh aap ka kya rad-e-amal tha jab apko ek dam se yeh kefiyat aye aap ne kis tarah apnay aap kosambhala ya aap ko kya laga tha?**

Interviewee: **Inaudible.* Hum ne in ko sambhal liya, yeh bari baat hai. Hum ne iss cheez ko itna nahi diya keh woh hum bhi pareshan hojayein, lekin waqti pareshani toh hoti hai, har kisi ko hoti hai lekin jo saath rakhtay hain,uss kay saath yeh mamlaat hain, jo sath nahi rakhtay hain un kay liye toh koi nahi. Woh toh kehte hain doh din toh aur sahi, doh din toh aur sahein. *(Please revise this segment from 15:00-15:30.)**

**Interviewer: Aur aap ke bachon ko inki beemari ka pata hai? Matlab woh toh itnay baray hogaye hain ke unki dekh bhaal bhi kartay hongay aap bata rahay thay. Un ko sab pata hai dawaiyon ka?**

Interviewee: Jee, mein toh sara din ghar rehta he nahi hun. *Inaudible [15:43-15:48]*inn kay khanay peenay ka, dawaiyon ka.

**Interviewer: Toh bachon ka kya rad-e-amal tha jab bachon ko pata chala keh pehle toh bilkul theek theen?**

Interviewee: Woh aksar batatay rehtay hain yaar ammi achi theen toh *Inaudible[15:58-16:02]*pehle esa hota tha ab esa hota hai.

**Interviewer: Aur udaas hotay hain*,* bachay udaas hotay hain ya kuch?**

Interviewee: Hojatay hain. *Inaudible.[16:11-16:15]*

**Interviewer: Bachon pe kya farq para, betiyon ki zindagi pe koi farq para hai?**

Interviewee: Ek din, doh din, teen din, hafta, maheena. Nahi esa kuch nahi, mein unko itna matlab ke woh karta hunk eh, support karta hun, baton se bhi aur un ki zarooraton se bhi *Inaudible [16:34-16:36]*

**Interviewer: Aur aap ko doctor ke paas anay ka kis ne mashvara diya tha?**

Interviewee: Doctor ke paas toh agar mashvara diya gaya tha, nafsiyati hospital janay ka, phir hum nazimabad gaye thay, wahan se ilaaj karwaya. Phir inki ek bhanji hain, woh *Inaudible* course waghera kar rahi thi, unhon ne phir ek doctor *Inaudible* ka bataya, un ke sath jo hai woh * saath milti rehti theen, *Inaudible,* unko dikhaya, acha treatment kiya, theek hogayeen. Ab bhi Dr. * ko dikhanay ke liye *Inaudible [Whole segment was inaudible 16:47-17:46]*

**Interviewer: Aap ko kya lagta hai, keh jo aap ka personal rishta hai, woh kis tarah badla hai, aap keh aur aap ki wife kebeech, unki beemari ki waja se?**

Interviewee: Rishta toh koi khaas matlab change nahi hua. *Inaudible [18:02-18:05]*

**Interviewer: Kabhi aap ko zehni uljhan ya zehni dabao ya kisi kism ka nafsiyati masla unki beemari ki waja se hua haiapko personally?**

Interviewee: Shuroo mein hua tha, lekin abhi nahi.

**Interviewer: Aur aap ko lagta hai keh inki beemari ki waja se aap ke kisi kism ke aur jo rishtay waghera hain, aap batarahay thay aap ke bhai waghera jo saath rehtay hain, kabhi un se koi pareshani, kisi tarah unkirelationship mein koi farq para ho?**

Interviewee: Nahi.

**Interviewer: Acha aur ap ko dawaiyon wayaiyon waja se mehnga, koi financial problems kisi kism ki, doctor ki visitsaur yeh sab?**

Interviewee: Nahi esa kuch nahi hota hai, dawaiyan jitni hain hum unko afford kar sakein, *Inaudible [18:52-18:55]*

**Interviewer: Kabhi jese inko agar episode huay, esi kefiyat hui, boht ghussay mein koi utha ke cheez phenki hai ya kisiko maara hai? Kabhi jese aap kokabhi toh nahi maara hai?**

Interviewee: Kisi ko nahi *Inaudible [19:12-19:16]*Agar zyada unko tang karo, ke aap ko chai peeni hai *Inaudible [19:20-19:27]*kabhikabhaar.

**Interviewer: Kabhi aap ne, jese banda kabhi boht pareshan hota hai, toh aap ne toh inn pay kabhi nahi haath uthaya? Aisa kabhi hua hai?**

Interviewee: Nahi esay haath waghera uthanay ki naubat toh nahi aye magar *Inaudible [19:38-19:44]*toh kabhi na kabhi toh koi na koi esi koi baat ho he jati hai jis se banda… lekin esa nahi ke koi haath uthaye.

**Interviewer: Acha ab hum ap ki roz mara zindagi ke baaray mein maloom karna chaheingay, matlab aap ka normal ekroutine ka day hota hai, kiss tarah ka hota hai?**

Interviewee: Uthtay hain, subha shop pe chalay jaatay hain. Aur namaz parh li, khana wana khaya.

**Interviewer: Aap ki wife apko nashta deti hain?**

Interviewee: Jab theek hoti hain, de deti hain. *Inaudible [20:18-20:20].* Har cheez. Raat ko mein der se aata hun 12 bajay, woh 1 bajay tak mera wait karti rehti hain.

**Interviewer: Phir lunch kartay hain, phir namaz parhtay hain, phir?**

Interviewee: Phir thori der *Inaudible [20:32-20:34]* Raat 11 bajay, 12 bajay.

**Interviewer: Aap ko lagta hai keh aapnay koi nayee kism ki zimedaariyan le li hain, jab se aap ki biwi ko koi bhiepisode hoti hai iss tarah ki, jab woh beemar par jati hain?**

Interviewee: *Inaudible [20:45-20:50] lekin* yeh hai zimedaariyon mein izafa hogaya hai. Jese dekho abhi mein shop close kar kay aya hun. Doctor ke paas le aya hun. Ab yahan se jaun ga, zohr hojayegi, namaz, khana wana khaya, lunch kiya, aram kiya, phir sham ko gaye. Phir inki dawaiyan le leen, *inaudible [21:04-21:07],* hamari ghar mein bachiyan baghair zaroorat ke bahar nahi jateen, hamara gharana he esa hai, matlab ke deeni gharana hai. Zaroorat hai boht zyada, phir jaati hain.Phir hum khudi koshish kartay hain ke apnay kaam khudi karein. Un ko takleef na dein. Bas iss kism ki baatein hain. Soda woda lana, markeet waghera jana. Pehlay yeh khud karti theen, ab hum kartay hain*.* Yeh cheezein hain.

**Interviewer: Toh farigh waqt mein kya kartay rehtay hain? Thora time mila tou?**

Interviewee: Thora time mila tou shop, sports dekh liyay, news parh li, *Inaudible [21:45-21:48]*.

**Interviewer: Bas ab kuch aur thoray sawalaat hain, humein lag raha hai hum aap ka time le rahay hain.**

Interviewee: Mujhay apni nahi, mujhay bas unka hai.

**Interviewer: Bas thoray se aur.. Aap ko lagta hai keh aap ko iss beemari ki baari mein achi maloomat hai, jab ap kopata chala ya doctor batatay hain?**

Interviewee: Han jee mujhay boht kuch pata hai inki beemari ke baray mein.

**Interviewer: Aap alag se parhtay hain jese kabhi kuch iss ke baray mein, yeh kya hai?**

Interviewee: Nahi mein in cheezon keh baray mein toh nahi parhta, lekin yeh hai maloomat keh liyay, general knowledge ke liyay, kuch bhi haath mein agaya toh-

**Interviewer: Lekin aap ke jo bachay hain, unhon ne Internet pe kabhi dekha beemari ke baray mein, unhon ne kabhikese handle karna hai, jab koi problem ho, aap ke bachon ne kabhi dekha hai?**

Interviewee: Han woh internet pe dekhtay hain.

**Interviewer: Aur aap ko pata hai beemari ke baray mein? Aur apko pata hai kis tarah sambhalna hai unko?**

Interviewee: Zahir hai, meri aadhi zindagi toh guzar gayee hai.

**Interviewer: Theek hai, wese toh MashaAllah aap kafi understanding hain lekin jab aisay nafsiyat ke ke problemshotay hain, toh khandaan matlab logon ko samajh nahi atay na kiss tarah handle karein, kya karein tohjese kaafi log yeh bhi mashwara detay hain ghar walay ke inko chor dein, ya talaakh dedein, kabhi aap neiss tarah socha hai?**

Interviewee: Nahi, hum ne kabhi talaakh ka nahi socha.

**Interviewer: Jese ghar walay boltay hain kabhi kabhi jese jo larkay walon ke ya larki walon ke…**

Interviewee: Koi nahi. Jab tak zinda hain toh jeena marna hai. Baki iss keh ilava koi ilehdgi ka nahi hua.

**Interviewer: Warna wese batana pasand kareingay, esay kya wajoohat hain? Ke talaakh ke baray mein nahi socha?Koi zaati, social?**

Interviewee: Hum rishton ko boht ehmiyat detay hain. Yeh talaakh dena jo hai na yeh boht hi ghalat kism ki baat hai. Iss mein samajh lo kisi ki zindagi khatam hi hojati hai. Life mein kya hai? *Inaudible [23:56-24:03].* Mard ke liye itna masla nahi khara hoga, lekin aurat ke liye boht masla hai.Agar bachay hon toh aur bara masla hai.

**Interviewer: Toh aap ko kabhi bhi esa laga hai kese inki beemari mein inki apni hee koi ghalti hai, jese unki kefiyatmein apni koi ghalti hai?**

Interviewee: Dekho khud se toh koi nahi kehta keh mein beemar hun. Koi nahi kehta hai mujhay bukhar, nazla, zukaam ho. Esa koi nahi chahta ke mein itni bari beemari mein par jaun.

**Interviewer: Aur aap ko lagta hai keh aap unko kisi bhi tareeqay say theek kar saktay hain?**

Interviewee: Koshish kar sakte hain.

**Interviewer: Aap ke liyay zyada ehmiyat kya cheez rakhti hai, jese dou miyan biwi ke beech ka rishta hota hai yapooray khandan mila ki ehmiyat zyada hoti hai?**

Interviewee: Bhai mian biwi mustehqum hongay toh koi masla hi nahi hoga. Agar yeh dono mustehqum nahi hain, toh phir uska rasta alag hai, apna rasta alag hai.

**Interviewer: Toh ussi baat pe phir, esi kya pursukoon khandan honay ke liye, esi kya zaruri cheezein honi chahiyain kepursukoon khandan ho ya healthy khandan ho?**

Interviewee: Kum bolein, yeh sab se.

**Interviewer: Aur bachon ke liyay kya pursukoon khandan kesa hona-**

Interviewee: Bachon ke liyay acha mahol qaim karna, hum khud achay rahein gay, humein dekh kay hamaray bachay bhi achay raheingay. Hum lartay jhagartay raheingay toh unko bhi…

**Interviewer: Aap apna mustaqbil kese dekhtay hain abhi?**

Interviewee: Alhamdulillah boht acha.

**Interviewer: Apni wife ke saath?**

Interviewee: Jee.

**Interviewer: Aap ko lagta hai ke religion ka koi iss pe, jese mazhab ka jo inki beemari hai, uss pe mazhab ka koi kismka kuch-**

Interviewee: Yeh ek mareez hain, uska jo zehn hai woh matlab ke yeh hai, namaz waghera ki paband hain, quran tilawat karti rehti hain, toh inka zehn bhi jo hai na ussi tarah ka hai. Yeh doctori ilaaj se yada roohani ilaaj ko zyada ehmiyat deti hain. Lekin jahan tak ham samajhtay hain, jahan tak hamari maloomat hai, theek hai, woh bhi esi baat nahi hai keh uss say kuch nuksaan hoga, karwana chahiyay insaan ko*,* lekin doctor jo doctor ke ilaaj se theek hosakta hai woh uss say nahi hosakta. Jo uss say hosakta hai woh iss say nahi hosakta. Lekin mareez ko mutmayeen karnay ke liyay uski bhi baat rakhni parti hai.

**Interviewer: Aap ne kabhi marriage counselling ka suna hai? Jese agar, wese aap ka toh MashaAllah bohtunderstanding wala rishta hai, lekin inn beemariyon ke waja se mian biwi mein boht jhagray hojatayhain.**

Interviewee: Jin logon ke andar jo hai bardaasht ka (maada?) nahi hota, ya jo log azaad khayal dimagh ke hotay hain, ya jo kisi se lagao nahi rakhtay, ya jo zindagi ka ek plan nahi banatay, woh jo hai phir…

**Interviewer: Toh esay mein marital counselling ek hoti hai, woh thora matlab help kartay hain keh aap iss tarah seinki baat sunein, yeh karein, toh aap ko lagta hai yeh ek achi cheez hai, jese marital counselling, koimadad kar sakti hai ek mian biwi ke rishtay ko mazeed mazboot karnay ke liye? Jab dou log ek doosray kibaat nahi suntay, toh teesra banda akay bolay toh phir, toh samajh ajati hai baat. Toh aap ko lagta hai issse madad mil sakti hai logon ko?**

Interviewee: Apnay halkay mein, doston mein, rishtedaaron mein boht saaray log hain jo acha bhi chahtay hain keh inke sath acha ho. Suno sab ki, karo apni. Aap ne mashwara diya, aap ne mashwara diya, aap ka bhi suna, aap ka bhi suna, hamari apni kitni *inaudible [28:24-28:28]*keh hum aap ke mashwaron ko kis had tak…kitna matlab samajh saktay hain ke aap ke mashwaron se humein kitna faaida hoga.

**Interviewer: Hamaray sawalaat complete hogaye hain. Agar aap kuch add karna chahein? Shukriya aap ne hamarayliye waqt nikala.**
