# Supplementary material for: Exploring effects of severe mental illnesses on marriages: A qualitative study from Karachi, Pakistan
Source: PLOS Glob Public Health. 2025 Dec 23;5(12):e0005652. doi: 10.1371/journal.pgph.0005652 (PMC12725543; doi:10.1371/journal.pgph.0005652)
Supplement: S1 Data — (ZIP) [file pgph.0005652.s001.zip › Transcriptions/Case 1 Transcripts/C1-2.docx]

**C1-1**

**Case 1**

**Note:** She did not want the interview to be recorded, hence everything was written by the interviewer (Sualeha). However, of course, because writing is difficult and the interviewee was pressed for time, not all the answers are written in great detail. Some of the essence may have been lost.

Her husband is diagnosed with Schizophrenia. Patient was admitted in the ward.

**Interviewer:** How long has the spouse been mentally ill?

**Interviewee:** A couple of months into the marriage, I realized that there were some problems with him (check from the demographic form)

**Interviewer:** When did you find out about the illness?

**Interviewee:** Just about the time when we got married.

**Interviewer:** Are your parents aware of the mental illness?

**Interviewee:** Yes. In fact, it was my father who first stated that my husband has schizophrenia and made us go for a check-up. This is why my husband does not like my father.

**Interviewer:** Okay, do you feel that you get adequate support?

**Interviewee:** Yes, Thank God, I have an ample amount of support. From my family, as well as my in-laws. In addition, I also have government support in Canada. My husband is often unemployed so we do get benefits. We also had some financial problems initially.

**Interviewer:** All right, so what kind of problems do you face in this marriage? How stressful does it get?

**Interviewee:** there is a lot of hassle actually. There are many problems. My husband has an aggressive side which leads to increased frustration on my side. But my mother tells me to let it be and compromise because my husband has schizophrenia and it is out of his control. In fact, I was also so stressed out that back in Canada, which is where I live, I had to undergo through counseling because it was getting very difficult for me to cope up especially with my son.

**Interviewer:** All right, and what do find more frustrating?

**Interviewee:** Well my husband had adjustment problems when he went to Canada initially for his job before we got married. Some of the problems actually started from there. And whenever even now, he has an active phase, we have to come back and that gets very frustrating. Coming from and going back to Canada. It gets very problematic because her brother helps her and her husband to come to the doctor. Even today, I have come with my brother. My entire schedule gets disturbed so that is a lot of hassle.

**Interviewer:** All right and do you think your support helps the patient?

**Interviewee:** Yes it does but sometimes I also feel guilty because I get angry at him, as well and get frustrated with him. I also scream and shout. I become quite angry. I got pregnant and he took a lot of care for me during my pregnancy. But then it became very troublesome after my baby was born and I thought of leaving him after 4-5 months.

**Interviewer:** All right and what do you think were your reasons that you did not leave him?

**Interviewee:** well, I have a child with him so I don’t want my child to grow up without a father. Also, I do have some attachment and love for him, especially because when he is feeling well, he is extremely caring. At times, I feel I do not provide enough support and I should provide more. In fact, sometimes I get so stressed that often I feel I am forgetting things and my memory is getting weaker.

**Interviewer:** All right, do you feel that people ask a lot about your husband’s illness?

**Interviewee:** Well, in my in-laws, they tend to ask a lot but not from my mother’s side. In fact, no one even knew about the severity of the problem until this time he was admitted in the hospital.

**Interviewer:** How do you think the family dynamics have changed?

**Interviewee:** Well they have changed a lot. I also got very stressed out and depressed.

**Interviewer:** What was your first reaction when you found about the illness?

**Interviewee:** I don’ really remember.

**Interviewer:** who encouraged in seeking help from the doctor?

**Interviewee:** my brother-in-law took him to the doctor.

**Interviewer:** How has your relationship changed since the onset of illness?

**Interviewee:** Well there were some problems from before actually. My husband already had problems.

**Interviewer:** Has the illness affected the relationship that your spouse has with other people?

**Interviewee:** not impacted a lot actually.

**Interviewer:** Has the mental illness of your spouse led to any problems of your own?

**Interviewee:** yes I was very depressed and stressed out which is why I was going to a counselor.

**Interviewer:** Okay and you said he gets aggressive. Aggression in what manner? Does he scream at you?

**Interviewee:** Yes he screams at me quite a lot. And sometimes he is extremely sarcastic and would say things in a very sarcastic manner. Sometimes he has even manhandled me, but not too violent that I was extremely hurt.

**Interviewer:** All right, and can you tell me about your day? And your responsibilities?

**Interviewee:** Well I do mainly cooking and cleaning, but my husband actually helps out a lot. He helps me in cleaning and cooking and also facilitates in taking care of the child.

**Interviewer:** Did you have to take any additional responsibility because of the illness of your spouse?

**Interviewee:** Well, not really, except I had to often pay the credit card bills because my husband was unable to take care of it.

**Interviewer:** Okay, and do you get leisure time?

**Interviewee:** Yes I do.

**Interviewer:** Do you think you know enough about the illness?

**Interviewee:** Yes, I actually searched a lot of the Internet and whatever I read on the Internet actually depressed me, as well. But I know a lot about the illness.

**Interviewer:** Do you think it’s your husband’s fault that he has the mental illness?

**Interviewee:** Well not his fault but when he went to Canada, there were problems which triggered the illness. He was away from family and he also faced some financial problems. Furthermore, there was harsh weather.

And then when he got ill, he thought it was some form of badua (ill-wishing) from his ex-fiance, because he had dumped her.

**Interviewer:** Do you think you can fix your husband?

**Interviewee:** No, I cannot change him at all, but I just can help him whenever he has his mood swings.

**Interviewer:** All right, do you ever think of divorce?

**Interviewee:** Yes quite a lot. Whenever we have a fight, I always think if divorce

**Interviewer:** Was it ever suggested by anyone in the family?

**Interviewee:** yes the first time my mother suggested it when we found out about the illness.

**Interviewer:** Do you think religion has an impact in the illness of your spouse?

**Interviewee:** yes

**Interviewer:** All right, do you think the family or the marriage is important?

**Interviewee:** Well, marriage is important more than the family

**Interviewer:** How do you see your future?

**Interviewee:** I see a bleak future

**Interviewer:** All right, thank you for your time.

***Interview Ends***
