# Supplementary material for: Exploring effects of severe mental illnesses on marriages: A qualitative study from Karachi, Pakistan
Source: PLOS Glob Public Health. 2025 Dec 23;5(12):e0005652. doi: 10.1371/journal.pgph.0005652 (PMC12725543; doi:10.1371/journal.pgph.0005652)
Supplement: S1 Data — (ZIP) [file pgph.0005652.s001.zip › Transcriptions/Case 1 Transcripts/C1-20.docx]

**Case 1**

**Patient was admitted in the ward**

**What’s your name? Full name?**

Sana Anas.

***And aap ki education kitni hai?***

Masters in English Literature.

**And do you work?**

Yes.

**As?**

Teacher.Kindergarten teacher, Haq Academy. Don’t mention the school there.

**OK, OK. I won’t mention it. How long have you been working?**

8 years.

**And how long have you been married for?**

14**.**

**Do you live in a nuclear family set up?**

Joint.

**Alright, how many people are there in the house at the moment?**

My mother-in-law, me, my husband and 2 daughters.

**So that makes?**

5.

**5 people, yeah. And your husband and you both earn right?**

Currently, he’s not working.

**Oh, would you mind telling, like,the estimate income of the household, overall? That is, if you don’t mindtelling me. Monthly?**

Around 1 lac.

**And you have 2 daughters, you were saying, right? No sons. All right, and how much has your husband studied?**

He’s an MBA.

**And what about your father,*your* father?**

My father. His education? He studied till Class 8.

**In your family was there any kind of psychiatric history?**

He’s my cousin, my husband is my cousin. My *mamu’s* son. My *mamu* was a patient of depression.

**Alright. And have you experienced any psychiatric illness in your life?**

Not yet, but during this period of 2 years I’ve taken a pill for 3 months, around 3 months.

**Anti-depressant? Prescribed by the doctor???? So how long has your husband been admitted in the ward for this time?**

This is the 3^rd^ time.

**And how long, duration?**

Like here he was admitted on Friday but 8 days before that he was in another hospital.

**So you’re saying that the illness has been occurring since 2 years, right?**

But this is a relapse. After one year.

**Do you know how many times he has been hospitalized?**

3 times. This is the 3^rd.^

**Alright so you went to the psychiatrist for help and a psychologist. Did you go to a faith healer, you know,*jo dumkarana aur yeh sub?***

Alongside my mother-in-law. But that started way back after we got into all this. When all the medications and nothing helped. Then she came into this ??? let’s go to a religious person.

**Would you mind telling me if your husband has any trouble with alcohol or drugs?**

No, he’s never had it but in the last 4-6 months he confessed that yes I was taking an alcoholic beer but I’m not addicted, once in a while, but he’s not addicted, he can live without it.

**Would you mind telling me if there’s any kind of problems you experience, financial at the moment?**

Yes definitely.

**??? Are there any kind of marital difficulties at the moment?**

Marital? Yeah there are times when I’m so desperate and I feel like I can’t carry it on, no matter after 14 years of relationship.There are times I feel like that. I feel like I can’t carry on.

**Has your husband had any relation problems with other family members, your daughters or your mother-in-law?**

He’s generally very aggressive but very soft-hearted and loving person.

**I’ll ask a couple of questions. You found out about the illness after marriage. You never knew about it.**

???

**You never knew about it?**

Not even about my father-in-law, who was my *Mamu*. ???

**So how long has your spouse been mentally ill?**

First time, he had this attack 2011. Carried on for 10 months. Then for a year, he was alright, all fine. Then, a relapse occurred in 2013.

**When did you find out about the illness, like what incident occurred?**

2011 was the first time that he was actually diagnosed. ??? Those 10 months he wasn’t admitted. That wasn’t that severe form of depression. It was just cured with the medicine.

**Are your parents aware? ???**

Yes definitely.

**What was their reaction *jab unko pata chala*?Did they know about this before marriage?**

No they never knew. Nobody knew definitely. Definitely,it was a shock for all of us.

**What’s your support system like? Do you have any sort of help in terms of?**

My parents live in Lahore. So if I think about my family,it’s not even here.My mother-in-law at my place, 2 sisters-in-law. They’re not here, one is in Canada, one is in Islamabad. He doesn’t have a brother. No father-in-law.Only 3 relatives, both mine and Anas, my khala is his phupho, they are there at times when do need them, they do come up, but it’s basically me and my mother-in-law who are with him.

**So in terms of financial help you’re pretty much independent. ???**

We have the deposit in the bank. That’s what’s working in these days, in the last 2 years

**How stressed does it get?**

Very stressful.

**What kind of hassle do you face, emotional or financial?**

Though I work it’s hard for me to carry it on with everything. My daughter was going to another school but now shifted her school with me. And now, we three, all go together in one campus. But there have been many times when I thought of quitting the job. My family might supportor maybe somebody else, I wanted to give up but then I realized that no, it’s actually a blessing for me. When I go there I have someplace that is an outlet for me. But yes, when you are working there is a lot of workload, that stresses me out.

**??? Do you guys have doctor visits?**

For him? Anas? Yes, definitely.

**So how does your support help the patient? Has your husband *kya* in any way has it helped?**

I just have to maintain my positivity all the time, no matter how negative I get but yes I’m human and there are times when even I also blast???.

**Do you feel that youplay a role in his recovery?**

Yes, I do play a role but I can’t change the circumstances,can’t change the disease. My helping can’t 100 percent change the disease.It will go on, the way it is, it will have its phases. I just need to cope up with it.But I can’t change the scenario, even if I change the scenario or circumstances that are feasible for him, his illness has, it has root causes definitely along with genetic factors, even if we try to figure it out but now I realize. Initially, I used to think if all gets sorted out he might get better, he might get better. But things don’t go the way we thought.It’s hard for me to realize. My mother-in-lawis still not admitting, she thinks *nahi, inhein sahi karengay sahi hojayega*. I try to tell her but elderly people remain fixed to what they believe in.

**How often do you guys go out to socialize? Do you people go out?**

??? Since he’s not well butyeah I try to make it so that on the weekend, maybe we can go out. I try but it’s not always there sometimes he would sometimes he wouldn’t.

**Do people try to interfere, do people ask you *“in ko kya ha*i, *in ko kya problem hai*”, what response do you give, do you tell them?**

People here in Karachi, they all know. Our relatives. They don’t ask “*kya hai”* but “how’s he feeling now?” Just to keep themselves updated. But I’m not the spokesman. I can never do that. Very hard for me to keep repeating. I can’t say much, this is something else, I can help you out with this.I don’t want to repeat my story, I don’t want to talk to people. Ihave stopped picking up my PTCL, don’t want to talk to anybody. “*Ab kesa hai? Ab kya hua hai? Ab doctor kya bol raha hai?”*

**We’re trying to ask more about you, you are the caregiver, might get really stressful for you. Might want to help people like you who face the burden of disease. Our research has caregiver burden is also an aspect that is otherwise ignored.**

**What about your friends? Do they know?**

My school has been very cooperative with me. Don’t mention the school name.

**We won’t even mention anything about you.**

I just worry because I did say the name in the beginning.

**Don’t worry. The recording won’t go anywhere.**

The administration, the only person I talk to there. Yes, I talk to them.They’ve been very cooperativewith me since the beginning when he used to … they used to give me … in case I’m not here for a whole year.??? Theyhave been really supportive throughout and even till now. What was your question?

**Friends? You can answer it if you like (phone ringing).**

I don’t. No I don’t want to. Yes, it’s my mother.Don’t wanna pick up right now.She’s the most important person in my life. I do talk to her, at night, hardly for 3-4 minutes. There are people who feel better when they vent it out but I’m not that sort so I stay silent.That’s my way of…

**Do you feel there is a stigma surrounding mental illness?**

Yes, definitely. To an extent that I don’t even want my children to know. My elder daughter is 12 years old. I only confided in her last year. She was very upset, her first year in this school was pathetic. She’s better now,because people, even we have a psychologist in school, we had some sessions with her and even she used to guide me to talk to her because she’s not at the age where I should be silent with her. So yes, I keep talking to her. But she remains quiet about it, she doesn’t have questions or maybe she has many questions and doesn’t want to. My younger one has no clue. I don’t want her to know.

**How old is she?**

6.

**Since you found about the illness how has it changed your family dynamics? *Kya change aya hai?***

Especially in this episode, the second, I feel that I’m a single parent raising my children. This is my actual feeling. To an extent that I feel now, even when Anas is not home for 10 days, my little one who is really very close to her father, she doesn’t even ask me much and this was quite a shock for me, *keh, why is it like that?* Now I realise that his interaction with the children have been so missing in the house, and what he was doing for them, physically, maybe that’s the reason children are already so detached. Definitely, they know it from inside but *ek hota hai na*, *they keep asking from me, normal ghar mein hota hai* and maybe they’re used to it, and he doesn’t play any rolein the house. Nowadays it’s all about him and what he’s doing,what he’s up to. It’s very sad to realize.

**So who encouraged you in seeking help?**

Nobody, I knew it myself.

**And as per your own relationship with your husband? Anything, A relationship between husband and wife how has thatchanged since the onset of the illness?**

It has changed, yes it has changed.

**If you would like to answer…**

Or maybe I can say it hasn’t changed much.We’ve never had a very healthy relationship actually.

**You did mention initially, he has thora aggressive attitude, but he’s soft at heart how has his relationship been with others since onset? Before episode and now?**

Almost the same.

**So you said that for like 3 months you were taking anti-depressants, you feel his illness has caused you to have some sort of mental healthproblems of your own?**

Not mental health. But yes, I need help, I need guidance. And previously I was having these therapy sessions at my school that the school admin arranged for me. But now when the year began, I didn’t seek their help. But I feel now, the illness is getting worse so am I. But I feel that I also need coping strategies. But I don’t want medicines, it never helped me, people think that it did but I don’t personally feel. I feel it’s my willpower and my faith in God, because I personally feel that yes, if any patient or anyone has a firm belief, any religion, whoever be their God, they know there is somebody to look up to.You know, actually adhere to their beliefs and values, and my husband is not at all a religious person. This is something which I instill in my children a lot. I want them to be *namazi,* they go to Quran School, this is something very basic that needs to be there in every human being and then when I think of them having a father like this,then definitely have to look at all aspects and this is what I can do for my daughters, thatthey are really close to their God, they know everything happens from up there. This is my own reflection I think.

**Just a hypothetical question. Do you think that if someone knows about a person’s mental illness, do you think *ke unko un se shaadi karni chahiyay?* Should they should get married to them if they have a mental illnes?**

I don’t think so. Ends with disasters. OK, I’m here, maybe, whatever reason I’m willing to be with him. I don’t want to leave him. I know it’s not his mistake, it’s the illness. And how can I leave him in this situation? No matter how brutal he’s been with me when he was OK for first 7 8 9 years ofhis life. But I’ve forgiven him, I can’t leave a sick person like that. But it’s not always going to be like that. There would be people who l would leave their spouses. And then there would be children. It’s for parents to know and understand ???. They should not be you know put in this situation. That they have a family, a backup system, that they have to??? I don’t personally recommend.

**If you would like to answer, you mentioned the word brutal.**

Yes he’s physically abused me, and to an extent that that I left him, went to my mother’s place and came back after 6 months and I was the one that said OK I want to continue because I had a daughter 1.5 year old. For her.

**Now it’s happened but if you would have known, do you think you would have married him?**

No.

**What’s your general day like? Morning to night.**

Wake up at 6, rush hour, going to school all 3 of us. Anas, sometimes he’s awake sometimes he’s not. Leave around 7:30 go to school come back around 2:15. 45 minutes, clothes, have lunch, go to Quran School, I drop them and my friend’s children too.Then around 4:30, she picks them, then drops them back around 4:30. Around 5:30,I sit with my little one for her homework and studies. Around 8, she goes to sleep. My older one sleeps around 11, then we have our dinner. But since the last 2 years, nothing has been, no set timetable, no routine, sort of disrupted.

**And your mother-in-law helps?**

Yeah she does. All the cooking, takes care of everything, although she didn’t used to do it before, but now after the illness, so much burden on you. Moving around the house, there is no driver, I do everything. Along with Anas, taking him to the doctor and everywhere. So yes, she does. But she is herself taking anti-depressants for so many years. So she has a very negative approach towards her life so I personally feel she’s not well so not just the situation itself but actually another personback home. She has actually experienced a lot of stress in her own life, her husband and now her son. Too much for her too.

**Whenever you get leisure time, although I doubt, what do you like to do?**

I have said no to so many outings andso many social gatherings in the past 2 years. I hardly go out with my friends, I just have to give an excuse, although I can go, I just don’t want to go. Always a no. Although I was such a fun-loving and the kind of person who wanted to go out every day, but now I’ve totally changed.

**???**

In our society not such a big thing. Although I thought it was a big thing that was the reason why I left him. But in our society very common especially in feudal system in Sindh more so from Lahore I would say. But he was always a very, not a stable mood I would say. ??? He would sway both poles. One moment he’s doing something really bad then next moment,he’s asking for forgiveness. That was something I didn’t find alarming because people are like that, they live throughout their live, but there was no signs. The illness was to come so it came.

**Were you aware of psychological illnesses before? Did it ever strike you of as a possibility?**

My father-in-law passed in 2003, I got married in 2000, so still I never thought, I never wanted to think that this is the reason why the son is acting like this.

**Following from this, once you found out, do you feel you know enough about the illness, have you ever researched online?**

Yeah I do but there are times I don’t want to know more. Because the more you know the more actually you feel depressed. But then when you know there’s lots of information out there and you’re not accessing it... Ido keep going back to it. ???

**You did mention that you’re aware that it’s the illness and not particularly your husband’s fault for being this way, and again, any other thing???**

But yes, I would say that children brought up from the parents major role. Always a lot of negativity. The air was negative in our house. Whoever would come, relatives, people, for my mother-in-law or father-in-law, they would always talk bad about them. So when your child has been hearing all this since childhood, at this age at least if you are saying“*beta nahi who toh esa nahi hai”* how can you change? You can never. Still I don’t think *ke* this is the reason why he’s sick or ill, there may be little triggering factors but not the actual reason.

**What would you say are the personal and social reasons for you to stay in this marriage?**

Personal and social reasons… Most important is, I can’t take my daughters away from their father.This is the major reason. I’m nobody to do that and I would never want it like that. Like what if my daughters grow up and ask me “Why did you leave him? He wasn’t well. It wasn’t his fault.”So I never want to hear this from them. Whatever is destined for him by God will eventually come. No matter how hard I’ll work or don’t. People keep telling me “*ese nahi kehte, you have to work hard”* We’re working for so many years and now I feel it hasn’t helped at all. I feel that this is all destined. My feelings, my beliefs,??? I started feeling like this.

**So at any point in time, did you ever feel that it is your spouse’s fault?**

No never.???

**And do you feel that solely only you yourself can fix him?**

No, not at all. Not even with my whole support system. It’s an illness, it will take its toll and take its time.

**In your opinion, in what circumstances should a couple seek divorce?**

Considering thisillness, you’re talking about that?

**Even in terms of illness and generally, divorce is a subject. Do what extent should something go for them to actually consider?**

When both agree to it.

**Any situation?**

Whatever situation comes, but if they’re not readyor willing to go on, they can seek.

**Did you ever in your life ever consider divorce?**

Yes.???

**Has it been suggested to you by family members and friends?**

Yes did happen in Lahore when I left him. My family wasstrongly against it. It was me who took the decision, I have to go back.

**Generally,if you think about it… what do you feel could be the one thing that could easeyour burden? Government support?*Chalein, Pakistan mein toh maybe not.* But family support? Financial support?**

These things are going to grow more and more as time passes. ??? Not depression, this is bipolar. This is a very big deal for me. Now I know that this will go on. It will have its stages. We are actually thinking about various, whether we need to continue in this city or not. Whether we need to… what kind of work we should pursue or start, something new, where not just my husband but me and my mother-in-law are also involved. There might be times when he may not be working. So my people, back in my family, are actually very surprised *keh tum itni jaldi accept kar rahi ho and how you’re coming upto these realities and you are actually the one telling us that yeh hai aur ab mujhay yeh dekhna hai.* But I think the more I am realistic, the more it will help me better.

**What do you think is more important? Marriage between 2 people or the family as a whole?Daughters, joint family system? Or both have their…?**

Marriage between 2 persons and, sorry?

**Or family as a whole? Daughters? Joint family system? Or just the relations between a husband and a wife? What is more important?**

It can only survive if the family is good, letting you grow. Only then the relationship between the 2 persons can go on. The background of the family and you may say the couple is.

**Based on that, what do you think are the essential building blocks of a healthy family?**

Children play a major role. Trust. Trust is something very important I would say.

**Marriage counselling, you’ve probably heard of it? What are your views? Do you think it would be helpful for a couple to seek marriage counselling in Pakistan?**

Not in this scenario, it isn’t a marriage problem. It is an illness that I need to know cope and know more about it. And ways how to go about it, with a person who’s not well. I don’t need a marriage counsellor. If I start blaming the person but then yeah I would need help. But since I know it’s not the person’s fault. ??? Yes that time yes I needed one but not now.

**Do you feel that religion has some sort of influence on his illness?**

Religion? Yes, lack of religion. Lack of belief and faith in the oneness of any creator plays a major role.

**On the other side of it, people say “*jaadu hogaya hai*.”**

My mother-in-law tries to prove to me that there are people who have done it.*Hum ne maulvi saab se pata kiya hai.* Those stories I don’twant to repeat,*jo unhon ne sunayee hain mujhay.* I don’t say much to her, it’s okay, she feels better believing that. I just listen and ignore.It’s OK for me.I can’t change her belief system. Whether there is any point in changing… I can’t even do that. But yeah,*han kuch had tak* I also feel *keh mein yeh sochti hun kehagar Mohammed pe jaadu hosakta tha toh kisi bhi insaan pe hosakta hai.* But I am such a firm believer in Allah,that I know that even if it is *jaadu* done by any person in our family,but it is also coming from the will of God. When it is the will of God,then He is the one who’s going to do anything about it, the illness, the jaadu, whatever, it’s coming from above, and nobody else can heal except God.

**Sualeha would you like to add something else? You mentioned you’re very apprehensive about future because the financial constraints, how do you feel…**

It’s becoming very unpredictable for me. I don’t know. Because my father-in-law. That was the cause of his death??? My sister who is actually not related to my husband at all in that way…

**They’re cousins though right?**

They’re cousins but he’s my Mamu’s son, so my sister, the fathers are not the same, so not the same genes, my father is totally different, and my Mamu is from a different family, not coming from my mother, but she also suffers from schizophrenia in America ??? at the age of 35. With this history in my background,definitelyfor me to feel pessimistic about it is understandable. But yes, I know and I believe in miracles and it varies from situation to situation, person to person, my mother-in-law keeps telling me“*nahi wohi horaha repeat horahi hai kahani, wohi mere saath hua tha”.* I don’t believe in all that but yes it bugs me but then I keep thinking that whatever is coming from above is the will of God and if I feel content that is better for me no matter if I cry I have to face it. Easy said than done. But I try to do it most of the time.

**Would you like to add anything about the topic?**

I would like to have some tips or guidelines, maybe some literature is available things like that. Although do you know enough about bipolar, well there are a lot of illness and resources online, han definitely, I should also take advantage.
