# Supplementary material for: Exploring effects of severe mental illnesses on marriages: A qualitative study from Karachi, Pakistan
Source: PLOS Glob Public Health. 2025 Dec 23;5(12):e0005652. doi: 10.1371/journal.pgph.0005652 (PMC12725543; doi:10.1371/journal.pgph.0005652)
Supplement: S1 Data — (ZIP) [file pgph.0005652.s001.zip › Transcriptions/Case 1 Transcripts/C1-3.docx]

**Case 1**

**Location:** Ward

**Diagnosis:** Schizoaffective disorder

The subject did not allow the interview to be recorded. Therefore, following is a summary of the interview. The verbatim is in italics.

**Interviewer:** How long has your spouse been ill for?

**Interviewee:** Since 5 years

**Interviewer:** Okay and what were the symptoms?

**Interviewee:** Ghabhrahat, bechaini and insomnia. We took him to Dr. Asif and he used to very quiet then. He did not interact with anyone at that time. 2 years later, we got ver upset because he became completely catatonic. He did not eat anything. He went completely stiff. And then we took him to Liaquat. He has been hospitalized four times since then. He has also been on medications since 5 years

**Interviewer:** Okay and do you face any kind of financial difficulties?

**Interviewee:** Yes we have been facing many financial difficulties but *Allah kardeta hai.* We have some income and savings but this month’s salary has not come, so that is an issue.

**Interviewer:** Do you face any kind of marital difficulties?

**Interviewee:** Yes, he was always angry which created issues. He would also scream at times.

**Interviewer:** What was your first reaction to the illness of your spouse?

**Interviewee:** *Mein ghabra gaye thee.* The first time the children also started crying, but I tried to stay strong. He started eating himself after a few days.

**Interviewer:** What was your parents’ reaction to the illness?

**Interviewee:** *dua kartein hain but boht pareshaan hain*

**Interviewer:** All right, and what do you see your future?

**Interviewee:** I do not think about the future. *Buss Allah pe chordya hai.*

**Interviewer:** What do you know about the illness?

**Interviewee:** (did not know the name of the illness) *meiney suna hai boht buri beeemari hai. Lekin Allah ki marzihai. Jis key naseeb mein jo beemari ho.*

**Interviewer:** Do you get any kind of support or help?

**Interviewee:** My mother helps me out because I often get very stressed out.

**Interviewer:** What kind of hassles do you face?

**Interviewee:** *Pareeshani hoti hai. Bechani hoti hai.*

**Interviewer:** Do you think that your support helps the patient?

**Interviewee:** Yes it does.

**Interviewer:** How long has he been admitted in the ward for this time?

**Interviewee:** Since 4-5 weeks

**Interviewer:** Do you socialize as a couple?

**Interviewee:** Not a lot. He does not like going out a lot. *Inko dil nahi hai tu mein chup hogaye hun. Meiney bardasht karliya.*

**Interviewer:** Okay and do others question you about the illness?

**Interviewee:** Yes I tell everyone about his illness

**Interviewer:** Do you think that the family dynamics has changed post illness of your spouse?

**Interviewee:** *aana jaana kam hogaya hai.* The house atmosphere normally remains quiet.

**Interviewer:** Okay so when he has gotten angry with you, has he ever screamed at you and hit you?

**Interviewee:** Yes he has hit her before but not after the diagnosis. *Inko shaadi kay baadh shak tha.* *mentions an incident when she went to visit someone in the family right after the wedding and someone put make up on her, so he did not like that. And he did not talk to her for days but she started talking to him herself and she always takes care of him*

**Interviewer:** What is your children’s reaction to the illness?

**Interviewee:** Well, my younger daughter gets very sad when her father gets hyper. So they do not like it when he is very hyper

**Interviewer:** Who recommended you to take him to the doctor?

**Interviewee:** The elder brother recommended to take him to Dr. Asif

**Interviewer:** Has your routine changed after the illness of your spouse?

**Interviewee:** Same routine *bus meiney khana pina dediya tu theek hai*

**Interviewer:** Okay and has this diagnosis impacted relationship with others?

**Interviewee:** He used to very talkative, but now he normally stays quiet

**Interviewer:** Do you feel that his mental illness has led to any mental problems of your own?

**Interviewee:** Yes. *Udasi rehti hai.* I cry a lot *burdasht karleti hun. Dil behlati hun betion say baat karkey*

**Interviewer:** Okay and have you taken any additional responsibilities after his illness?

**Interviewee:** I always used to take care of everything before. Things outside and inside the house. I took care of my sons and daughter *unko jab chup lag gaye thi.* I give medications to him on time, as well.

**Interviewer:** Do you think you do those things that a husband would normally do?

**Interviewee:** Yes at times

**Interviewer:** What do you do in your leisure time?

**Interviewee:** I take care of the house. I also watch television

**Interviewer:** Do you feel that you know enough about the illness?

**Interviewee:** I do not think that I have enough knowledge. I want to know more so that I can understand it and then manage it accordingly.

**Interviewer:** Have you ever thought of separation or divorce?

**Interviewee:** No *bachay barey hogaye hain, ubh kya sochna.*

**Interviewer:** What do you think are the personals reasons for you staying back in the marriage?

**Interviewee:** *Bacho ki waja say*

**Interviewer:** Has divorce been suggested to you by anyone in the family?

**Interviewee:** No one has suggested anything

**Interviewer:** In what situations do you think a couple should seek divorce?

**Interviewee:** *Kisi ko bhi nahi leni chahye. Beemari mein chordena. Yeh tu koi baat nahi huwi.* Beemari should not be a reason. *Awal sochna hee nahi chahye*

**Interviewer:** Do you think it is your spouse’s fault to have the illness?

**Interviewee:** Yes, he thinks a lot. He gets tense over little things. When people make fun of him at work because of his illness, he gets very upset.

**Interviewer:** Do you think you can fix him?

**Interviewee:** *Koshish tu karungi, lekin yeh suntay hee nahi hain*

**Interviewer:** Do you feel that the marriage is more important or the family as a whole?

**Interviewee:** Marriage is more important

**Interviewer:** What do you think are the essential building blocks for raising a healthy family?

**Interviewee:** *Shauha acha ho, khayal rakhe. Dunu mil ke rahein.*

**Interviewer:** Do you think that marital counseling is a good solution?

**Interviewee:** He will think that *meiney shikait ki hai.* Lekin koi faida nahi hoga

**Interviewer:** How do you see your future?

**Interviewee:** Future *pause* *laughs* *Iska jawab nahi desakti.*

***Interview Ends***
