# Supplementary material for: Exploring effects of severe mental illnesses on marriages: A qualitative study from Karachi, Pakistan
Source: PLOS Glob Public Health. 2025 Dec 23;5(12):e0005652. doi: 10.1371/journal.pgph.0005652 (PMC12725543; doi:10.1371/journal.pgph.0005652)
Supplement: S1 Data — (ZIP) [file pgph.0005652.s001.zip › Transcriptions/Case 1 Transcripts/C1-4.docx]

**Case 1**

**12^th^ February, 2015**

**In-patient**

**Bipolar Disorder**

The wife did not allow for the recording of the interview; therefore, brief notes were taken. Due to the time constraints, some of the information may have been lost although there was an attempt to write down as much information as possible. Apostrophes have been used where a direct comment from the subject has been recorded. However, the interview was done in Urdu but for easy noting down of the data, the entire transcript is in English (apart from the direct quotes)

*during filling out the demographic questionnaire, the spouse reported that the patient was a civil servant. He had been first working in Civil Hospital but then he served in the revenue board. When the spouse was asked when the patient was diagnosed, she mentioned that she always found him strange since he was always quiet. She found that strange and she questioned her in-laws about it. They told her that he was always like this but before 2 years of the marriage, he had been very talkative. He got married when he was 24 years of age, whereas she was 22 years. Some of his symptoms included over-spending and he was extremely over-talkative. He also did not sleep for nights. Then someone her brother knew recommended Dr. Hanif Mesiya, and they took him for a check-up. This was in 2013. When they were based in Hyderabad, her mother-in-law also did Taweez. However, now they have shifted to Tando Alam. According to her ‘teezi khatam hogaye’ after a few months of taking medications. This is the first time that he has been admitted in the ward because he had become uncontrollable. He went extremely high. He started praying late into the night without ablution etc. The kids started getting disturbed. The dosage was increased but he was able to sleep only for two days. He also went to Hala to his parents without telling her anything and stayed there for around a week. Even during his stay in Hala, he used to be out of the house, used to take a rickshaw (auto) and roam around, meeting random people and his old friends. In fact, overspending also increased. This was before her brother’s wedding where she had to leave. Perhaps because of her absence, in which he did not take the medicine, he got high.. They had also previously taken him to a neurologist because they were extremely worried about the fact that he was unable to sleep for nights. She also mentions that this happens right after she had gotten Hepatitis injections. She firmly believes that this kind of illness has occurred because of these injections. When asked about the kind of problems that she faces during the course of her marriage, she mentions that they have been in debt a lot of times. In fact, he owned a bhains ka bhara (a sort of farm) which went into a huge loss and debt. Her brothers paid back the loan which amounted to 2 lakhs. Other problems that she faces during the course of her marriage is the fact that he is extremely over-sexual. He wants to have sexual intercourse at least 3-4 times a day, especially after breakfast and at night (when the children are not home). When she refuses, he gets angry. In fact, he even complained to the doctor that his wife did not indulge in sexual intercourse but the doctor stated that this act is also at her discretion and he should not force her. She herself mentions that she cannot take out time all the time*

**Interviewer:** aap ko lagta hai kay yeh beemari ka hissa hai?

**Interviewee:** Jee yeh beemari ka hissa hai. Yeh cheez boht zyada ahm hogaye ha

**Interviewer:** kya aap log cousins hain

**Interviewee:** cousins istarah kay yeh meri phupo kay betey kay betay hain

**Interviewer:** Acha, aap ko kis tarah ka support milta hai?

**Interviewee:** jee support milta hai bhai ki tarf say. Who hee doctor kay pass lekey aatey hain.

**Interviewer:** does your support help the patient?

**Interviewee:** yes, it does. I have to take care of him

**Interviewer:** Is he compliant with his medications?

**Interviewee:** No he is not. I have to give it to him. He in fact lies that oh I have taken the medicine but then I would find it elsewhere and even then he would deny saying he has taken them.

**Interviewer:** do you guys go out to socialize a lot?

**Interviewee:** Yes we go out a lot. We go out every week to Hyderabad to eat out.

**Interviewer:** Do people question you when he is in such a state?

**Interviewee:** yes when he is in such a state, people in Tando Alam also asked.

**Interviewer:** What did you say to them?

**Interviewee:** buss tabiat kharab hai. Beemari ki waja say aana jaana kam kya hua hai

**Interviewer:** All right and prior to the diagnosis, what was your relationship like? And what was his relationship with his family?

**Interviewee:** He was always quiet. In fact, my mother-in-law used to say “biwi kay aaney kay baadh chup lagi hai”. But one of my relatives used to defend me and used to say that this was there before he got married, as well.

**Interviewer:** acha and when he was diagnosed and before he got treated, how did the family atmosphere change?

**Interviewee:** It changed a lot because there was a lot of swearing and abusing. The kids got disturbed of course. He also used to fight a lot. The kids got shocked. They used to ask what has happened to their father.

**Interviewer:** what was your reaction when you found about the illness?

**Interviewee:** Nothing as such. Buss mujhe yeh pata tha kay ub humein ilaj karwana hoga.

**Interviewer:** acha when he gets angry, does he hit you or throw things at you?

**Interviewee:** No he just shouts but he has never gotten angry or thrown things at me.

**Interviewer:** have you done the same?

**Interviewee:** No

**Interviewer:** all right, what is your day-to day routine like?

**Interviewee:** well I do house work. And then I also have to attend to him. He always wants sex when he is normal (not just when he is high). This becomes an issue for me.

**Interviewer:** What do you do in your leisure time?

**Interviewee:** I go to my mother’s house or I watch television. Or I would also do some extra work such as cleaning cupboards etc. I try not to stay too much with him. I go to my mother’s because I don’t want him to ask me for sex all the time. But I cannot stay at my mother’s because I have to give him medicine. So I go in the afternoon but come back home after my kids come from tuition. And he also gets mad that I do not spend time with him. He threatens me with second marriage. I tell him “ja ka karlein. Mujhe koi dar nahi. Merey liye merey bachay kee kaafi hain”. Even my brother says that since we have supported you so much by getting her the plot and getting the house made, along with support for the treatment, we can also take care of you. “Mera rishta isliye hua tha kyunke merey abbu ki bhen khud chal kay ayen thee’. There wre other proposals as well, which were also better-suited.

**Interviewer:** all right and have you ever thought of divorce?

**Interviewee:** no I cannot.

**Interviewer:** has divorce been suggested to you by family members?

**Interviewee:** Yes my mother especially when he is like this says “chordou. Ubh sahi nahi hoyega” Also I am the only daughter of five brothers who are doing very well, so my mother often says that I should leave him.

**Interviewer:** What are the reasons for you then staying back in the marriage?

**Interviewee:** “Shauhar hai. Zahir hee see baat hai. Humaray bachay hain. Unkay liye zaruri hai kay hum dunu hun”

**Interviewer:** Acha and have you never thought of divorce?

**Interviewee:** “hum iskay barey mein nahi sochtay. Inkay naam hee hai humari zindagi”

**Interviewer:** do you think it’s your spouse’s fault to have the illness?

**Interviewee:** Yes definitely. He is overly sensitive so he thinks a lot which is why he gets this problem. He often thinks more when people say that he is being supported by the family.

*during the interview, her husband came a multiple times in the room. Once, her husband also said that he has not spent a single penny and he is being supported by her*

**Interviewer:** What do you think is important, marriage or family?

**Interviewee:** “waise tou marriage zyada ahm hai, lekin jahan aisee beemarian hun wahan family zyada importance rakhi hai”

**Interviewer:** Acha aur aap ney kabhi eladgi kay bareey mein nahi socha?

**Interviewee:** Nahi. Bacho kay liye nahi. Bacho kay liye boht zaruri hai kay ma baap eik saath rahein”

**Interviewer:** if you knew about the mental illness before marriage, would you have married him?

**Interviewee:** No

**Interviewer:** What are the essential building blocks for raising a healthy family?

**Interviewee:** There should be a futuristic outlook. We should look forward to doing more and better. It would not do good if we do not do well for the future. And he does not do that. He is happy with what he has but this is not the right vision.

**Interviewer:** how do you see your future?

**Interviewee:** “mein dekhteen hun” *laughs* “boht bura dekhteen hun. Sochteen hunk ay yeh theek hongay ya nahi. Bacho pe boht bura asr parta hai” I sometimes have to tell him to brush his teeth and this leaves such a bad effect on the family and the children

**Interviewer:** Do you know about marital counseling? And do you think this could be of help when mental illness is the problem?

**Interviewee:** I’ve heard about it but I don’t think if two people can’t resolve their own problems a third person would.

**Interviewer:** Do you think there is an influence of religion?

**Interviewee:** I don’t believe in it but he does. He goes to mazars and pir fakirs etc.

*additional comments. The girl has a masters and she has been married off to a person who has done his matriculation only*
