# Supplementary material for: Exploring effects of severe mental illnesses on marriages: A qualitative study from Karachi, Pakistan
Source: PLOS Glob Public Health. 2025 Dec 23;5(12):e0005652. doi: 10.1371/journal.pgph.0005652 (PMC12725543; doi:10.1371/journal.pgph.0005652)
Supplement: S1 Data — (ZIP) [file pgph.0005652.s001.zip › Transcriptions/Case 1 Transcripts/C1-5.docx]

**Case 1**

The interviewee refused for the recording of the interview. She was also hesitant in signing the consent form, but after much assurance that her name would not be used anywhere did she sign the consent form.

**Interviewer:** When did you find out about the illness?

**Interviewee:** When he tried to commit suicide, this is when I found out about the illness.

**Interviewer:** Did you know whether he had an illness before the marriage?

**Interviewee:** No, he always used to feel depressed though even before the diagnosis. I do not know whether he went to a psychiatrist or a psychologist.

**Interviewer:** When was the mental illness diagnosed?

**Interviewee:** 13 years after getting married.

**Interviewer:** And when the illness first occur and how did it occur?

**Interviewee:** He was suicidal and he had left, so everyone found out because we thought he had been kidnapped. In those days, kidnapping had become very common.

**Interviewer:** All right, so did your parents know?

**Interviewee:** Yes everyone found out about it because of him running away.

**Interviewer:** All right, so did you have any kind of support?

**Interviewee:** Yes my family helped and also because the psychiatric illness was present in their family, the in-laws also helped.

**Interviewer:** What kind of difficulties do you face because of his mental illness?

**Interviewee:** Not a lot now, but before when he was first diagnosed in 1993, it was extremely stressful. He generally is good when he is stable but he keeps to himself. However, I do have to work according to his mood so sometimes that gets stressful.

**Interviewer:** So there are no financial difficulties?

**Interviewee:** No Alhamdulilah

**Interviewer:** What tends to be quite frustrating?

**Interviewee:** Well, it is not too much of an issue, I think.

**Interviewer:** do you have a good relationship with him?

**Interviewee:** Yes, we do. But I cannot share my problems with him. That sometimes becomes an issue. Even if I have called a carpenter or something, I have to call him when he is not around because he gets stressed over little issues so I have to do everything on my own. That sometimes gets very frustrating. He sometimes gets angry, but everyone has temper issues so it does not really matter.

**Interviewer:** Are doctor visits frustrating for you?

**Interviewee:** He usually comes for routine follow-ups alone. Whenever he is not feeling well, like for now, I come with him.

**Interviewer:** Okay, and what usually are his symptoms?

**Interviewee:** He mostly shows depressive symptoms instead of mania. Generally, he is on anti-depressants. I cannot leave him alone so I don’t have a social life because I have to watch for his moods. I cannot go abroad to my children because he cannot be left alone.

**Interviewer:** Do you feel that your support helps the patient?

**Interviewee:** Yes

**Interviewer:** How often do you go out to socialize?

**Interviewee:** Not very often. But that’s not because of him. Even before marriage, I used to socialize very less.

**Interviewer:** Okay, do others question about the illness because you know there is this whole idea of stigma associated with mental illness?

**Interviewee:** No one really asks now because it is in the family so it is pretty much expected.

**Interviewer:** Do you feel that the family dynamics have been changed due to mental illness?

**Interviewee:** Not really, no.

**Interviewer:** what was your first reaction when you found out about the mental illness?

**Interviewee:** I don’t really remember now but I was stressed out because he had run away. I also did not know at that time how serious it was.

**Interviewer:** All right, and your children were old then, so what was your children’s reaction?

**Interviewee:** I don’t remember because I was too involved in myself. I did not even pay much notice to it.

**Interviewer:** Okay, who encouraged in seeking help?

**Interviewee:** Well, my sister’s husband recommended that we should seek the help of a psychiatrist. Even the in-laws suggested that this should be done because his brother goes to a psychiatrist, as well.

**Interviewer:** Has your relationship changed since the onset of the illness?

**Interviewee:** Not much because he was always quiet and kept to himself.

**Interviewer:** Has your relationship with others been impacted because of the illness?

**Interviewee:** Not really, no.

**Interviewer:** All right, and has his mental illness led to any mental health problems of your own?

**Interviewee:** Not really, apart from the fact that I am stressed out.

**Interviewer:** Okay, does he ever get violent in one of his moods?

**Interviewee:** Not really. And he has only hit me once and that was because he was getting high at that time and the doctor did not notice. The dosage of anti-depressants kept on being increased and I told the doctor this problem but he did not listen to me.

**Interviewer:** Okay and what were his symptoms when he gets high?

**Interviewee:** He usually starts eating a lot. He has his dinner but then he would keep on going back to eat. I can see it from his eyes. There is a change in his personality. He would get Pepsi cravings also. And there is a lot of restlessness.

**Interviewer:** All right and what was the instance when he hit you?

**Interviewee:** Well, he hit my daughter first because she was on the phone and he kept on getting irritated and he hit her. I intervened and said that if you do not come in control, we will take you to the doctor. So he hit me then.

**Interviewer:** Okay, do you mind telling me about your daily routine?

**Interviewee:** Well, I do not do much generally. I go for a walk in the morning, come home and make breakfast for my husband and he takes lunch to the office, as well.

**Interviewer:** How much of your time daily is devoted to taking care of your husband?

**Interviewee:** Not a lot. I just have to take care of his medications. He usually is in his own shell and he gets super irritated if I bother him too much.

**Interviewer:** Okay and what additional responsibilities have you taken post-illness?

**Interviewee:** Well, it did not make much difference because I was doing a lot of work already from before. He was never involved in the family from the beginning. Even right now, I do not even have a lot of responsibility

**Interviewer:** Okay, what do you do in your leisure time?

**Interviewee:** Well, stitching sometimes and now WhatsApp (laughs)

**Interviewer:** Okay, and what about when your kids were young, did you get time for yourself?

**Interviewee:** Not at all. I was always taking care of everyone. Initially, I used to feel lonely but he was always like that, so now it does not make a difference.

**Interviewer:** Okay, and do you think you know enough about this illness?

**Interviewee:** yes quite a lot. Even my mother has this problem.

**Interviewer:** All right, and what do you think are the reasons for this illness?

**Interviewee:** Well, I think *pause* it’s a combination of hereditary and environment.

**Interviewer:** Do you feel that it is your spouse’s fault to have this illness?

**Interviewee:** Well, not really fault but yes it is in his hands. He was pampered too much by his mother because he was born after the death of a few sons. He always had this idea that oh I need help, I need help and I am not too strong. He has this need for dependency. His elder brother also used to bully him.

**Interviewer:** Do you feel that you can fix him?

**Interviewee:** Not really, because it is the chemical in his brain that has led to this problem. But therapy can make a difference in the sense that he always wants to be alone, and this will help him.

**Interviewer:** Okay and have you ever thought of separation?

**Interviewee:** never.

**Interviewer:** and was it suggested by friends or family?

**Interviewee:** no.

**Interviewer:** All right and when he was one of his moods, did you ever think of separation then?

**Interviewee:** Well, at times like these, I often felt like running away. To be honest, I am more relaxed when he is not at home.

**Interviewer:** All right and in what circumstances do you think that a couple should seek divorce?

**Interviewee:** well, if it is uncontrollable and there is a lot of beating, then a person should seek divorce.

**Interviewer:** Do you feel that the marriage is important or the family as a whole is important?

**Interviewee:** The marriage is more important.

**Interviewer:** Okay and what do you think are some of the essential building blocks for raising a healthy family?

**Interviewee:** There should be caring amongst the people. There should be understanding and respect for each other. Husband and wife should give each other proper time

**Interviewer:** Hmm and do you feel that this is present in your marriage?

**Interviewee:** No we do not give each other time. I don’t know. Buss kaam chal raha hai (it is just coming along). He does not insult me but then he is also involved in his own life. We do not have any kind of intimacy or closeness and this has been there since the beginning of the marriage.

Generally in a marriage, husband and wife should also give space to each other and that’s present. I would suffocate if he would not give me space.

**Interviewer:** Do you feel that marital counseling can be of any help when mental illness is a problem?

**Interviewee:** Yes.

**Interviewer:** How do you see your future?

**Interviewee:** Well, I would be in my grave maybe. Who knows? I am already 57! *laughs*

***Interview Ends***
