# Supplementary material for: Exploring effects of severe mental illnesses on marriages: A qualitative study from Karachi, Pakistan
Source: PLOS Glob Public Health. 2025 Dec 23;5(12):e0005652. doi: 10.1371/journal.pgph.0005652 (PMC12725543; doi:10.1371/journal.pgph.0005652)
Supplement: S1 Data — (ZIP) [file pgph.0005652.s001.zip › Transcriptions/Case 1 Transcripts/C1-6.docx]

26^th^ May 2015

**Case 1**

**Illness:** Bipolar Disorder

The spouse did not allow the interview to be recorded. She seemed quite upset during the course of the interview. Specific verbatim is in italics.

**Interviewer:** When was your spouse’s mental illness diagnosed?

**Interviewee:** 2 years back

**Interviewer:** What happened when you realized that you needed to take him to a psychiatrist?

**Interviewee:** He was not in his *Hosh. Depression mein chaleygaye thay. Apne aap ko barey aadmi samajhtey thay.*

**Interviewer:** Okay, And do you have any financial constraints?

**Interviewee:** yes when he was not feeling well, he overspent all of his savings. And the income is used up in all the expenses, which has become problematic.

**Interviewer:** Do you have any kind of relationship problems with others?

**Interviewee:** *Jab insaan beemar hota hai, khandaan waley dimag mein ghalat batein bithatay hain. Zaruri nahi saari families strong hee hun.*

**Interviewer:** Inki family mein koi psychiatric illness hai?

**Interviewee:** Yes, *lekin mujhe nahi andaza tha kay itni buri hogi.*

**Interviewer:** Was it an arranged marriage?

**Interviewee:** Yes

**Interviewer:** Do your parents know about his illness?

**Interviewee:** Yes, they do

**Interviewer:** Do you feel you have support at home?

**Interviewee:** No I don’t. I live alone with my two children. And whenever he is hospitalized, then I have to manage home as well as visits to the hospital. Children are home alone. Coming to the doctor is also quite difficult at all times.

**Interviewer:** Do you feel your support helps the patient?

**Interviewee:** *laughs* *inko nahi lagta kay mein inki madad karte hun. Woh humeisha yehi kehtey hain kay aap meri care kahan karteen hain. Mein khud hee chala jaonga doctor kay pass. Aap nahi aye.*

**Interviewer:** Does his family help?

**Interviewee:** No. His family actually says I have made him ill. Even he says *yeh bhee kehtay hain kay aap ne mujhe beemar kya hai.*

**Interviewer:** Aap ko kya lagta hai kay yeh kyun boltay hain?

**Interviewee:** *Beemari ki waja say boltay hongay* *tears in her eyes*

**Interviewer:** Do your parents help out?

**Interviewee:** No they are actually in Hyderabad

**Interviewer:** Do you socialize as a couple?

**Interviewee:** Yes

**Interviewer:** Do people know about the illness?

**Interviewee:** yes

**Interviewer:** And what is their reaction?

**Interviewee:** *Log kehtay hain. Pagal hogaya hai.*

**Interviewer:** What is your response to that?

**Interviewee:** *Mein kehti hun beemari hai. Kabhi low hojatay hain, kabhi hyper hojatay hain. Jo log educated hotay hain woh understand kartay hain warna nahi kartay*

**Interviewer:** What is your general reaction to the illness?

**Interviewee:** *Allah ki taraf say hai yeh beemari. Beemari mein koi pagal nahi hota*

**Interviewer:** Do you feel that the family dynamics have changed post illness of your spouse?

**Interviewee:** Yes, my children have gotten very disturbed. They did not get good marks in the exams. His family says I have done something. *Mein kya karungi?*

**Interviewer:** Do you have to manage medications etc?

**Interviewee:** Yes, I have to take care of that. Sometimes he does not take it so I have to feed him through his tea or curry

**Interviewer:** All right, does it get stressful for you managing his illness?

**Interviewee:** Yes, it gets very tough and I have no support. And he listens to his family.

**Interviewer:** all right, what motivated you to seek help and who encouraged you?

**Interviewee:** In 2013, he went into complete depression, *awazein sunai deti theeen. Koi hosh nahi tha. Depression mein agaye. Meiney khud AKU mein admit karwa diya inko.*

**Interviewer:** Do the children know about the illness?

**Interviewee:** yes my elder one is 14-15 years old. They know it’s depression. *Inko itna kuch nahi pata hai. Mein inko zyada dihaan nahi deney deti hun*

**Interviewer:** Do you feel your relationship has changed?

**Interviewee:** Yes

**Interviewer:** How?

**Interviewee:** *yeh galat baatein boltay hain.* *******stars crying*

**Interviewer:** Kistarah ki?

**Interviewee:** Shakes her head

**Interviewer:** Acha aap nahi bataye

**Interviewee:** *Buss eik farq aajata hai dil mein*

**Interviewer:** Acha aap nahi bataye. Koi masla nahi hai. Agar interview nahi dena, tu koi masla nahi hai

**Interviewee:** *Guzar chuki hai mujh pe. Agar kisi aur ko madad miljaye tu koi masla nahi hai*

**Interviewer:** Okay so how was your relationship before the illness?

**Interviewee:** We had a very good relationship. Before the illness, we had never fought.

**Interviewer:** Acha and after the illness, has he ever hit you?

**Interviewee:** yes *boht* *starts crying*

**Interviewer:** What about shouting and hitting the children?

**Interviewee:** He shouts at me, and at the children but does not hit the children

**Interviewer:** Do you have financial problems? And is he going to work these days?

**Interviewee:** No he has taken an off from work and yes, we have financial constraints

**Interviewer:** What is your daily routine?

**Interviewee:** It’s normal. I wake up at 6 am and I send children to school. I go for a walk and come back home and then I sleep for a while and send him to office after waking up. Then when kids come, I do their work. And in evening, we go to jamat khana

**Interviewer:** Okay and do you think you have taken any additional responsibilities after the illness of your spouse?

**Interviewee:** *Ubhi normal hain tu dekh letey hain bahir kay kaam but jab yeh hyper hotay hain, tu saarey kaam mujhe karne partay hain. In fact, bachi ko agar stationery bhee chahye hoti thee, tu mein hee jaati thee. Bacho ka dihaan bhi mujhe hee rakhna parta hai*

**Interviewer:** What do you do in your leisure time?

**Interviewee:** *Kapro ki silai waghera karleti hun*

**Interviewer:** do you feel you have knowledge about the illness?

**Interviewee:** *ubhi kuch maloom hua hai*

**Interviewer:** Aap ne online waghera search kya tha?

**Interviewee:** Haan kya tha

**Interviewer:** tu aap ne manage karne ka tareeqa waghera dekha?

**Interviewee:** haan *ub mein unko jawab nahi deti jab woh hyper hotay hain. Khud hee theek hojata hain.*

**Interviewer:** Have you ever thought of divorce?

**Interviewee:** No I have not.

**Interviewer:** has anyone suggested divorce?

**Interviewee:** No

**Interviewer:** Okay why do you continue to stay in the marriage?

**Interviewee:** *Humari zindagi tu guzar gaye. Bacho ki hai ubh. Inko parhana waghera hai. Dusri shaadi tu nahi karne tu kyun talaaq lein. Beemari apne haath mein tu nahi hoti. Allah kay haath mein hoti hai.*

**Interviewer:** Do you feel it is your spouse’s fault to have the illness?

**Interviewee:** No

**Interviewer:** What are the essential building block in raising a family?

**Interviewee:** *Understanding honi chahye*

**Interviewer:** Is it there in your marriage?

**Interviewee:** *Pehle thee, ubh nahi hai. Haalaat inki beemari kay baad boht kharab hogaye hain*

**Interviewer:** Has he ever thought of divorce during his hyper phase?

**Interviewee:** yes he has and I always tell him that I will not take it, if you want to give it, then you can give it.

**Interviewer:** Aap ne shaadi ko kyun barqarar rakha hua hai?

**Interviewee:** *Merey bacho ki waja say*

**Interviewer:** How do you see your future?

**Interviewee:** *Acha hee dikhtee hun. Beemari tu aati jaati rehti hain. Bachay hain*

**Interviewer:** Do you feel relationship between a married couple is more important or is the marriage more important?

**Interviewee:** Relationship is more important

**Interviewer:** Do you think marital counseling can help?

**Interviewee:** Yes

***Interview Ends***
