# Supplementary material for: Exploring effects of severe mental illnesses on marriages: A qualitative study from Karachi, Pakistan
Source: PLOS Glob Public Health. 2025 Dec 23;5(12):e0005652. doi: 10.1371/journal.pgph.0005652 (PMC12725543; doi:10.1371/journal.pgph.0005652)
Supplement: S1 Data — (ZIP) [file pgph.0005652.s001.zip › Transcriptions/Case 1 Transcripts/C1-7.docx]

**Case 1**

Out-patient clinics

The subject did not allow the interview to be recorded. Therefore, this lists down the pointers that were gathered during the course of the interview. The specific verbatim is listed in italics.

**Interviewer**: When was your husband diagnosed?

**Interviewee:** 1 year after our marriage

**Interviewer:** Are your parents alive?

**Interviewee:** No

**Interviewer:** Do you receive any kind of help or support?

**Interviewee:** No. The medicine is very expensive and we have a hard time.

**Interviewer:** Okay, what kind of hassles do you face?

**Interviewee:** Well, he screams a lot so that gets troublesome. In fact, he only does it at night. And during the day, he is fine. And because of this, I get very scared. We do not sleep in the same room. Once, he hit me a lot after waking up. After that, I have become very afraid.

**Interviewer:** All right, what kind of problems do you face in particular? Do you get angry?

**Interviewee:** I get angry at times. *Ghussa aata but kuch bolti nahi hun*

**Interviewer:** Okay and do you face financial difficulties?

**Interviewee:** Yes a lot of financial difficulties because his brothers also don’t support us at all.

**Interviewer:** Do you people go out to socialize?

**Interviewee:** I go a lot. I have friends etc

**Interviewer:** Do you guys socialize as a couple?

**Interviewee:** No Mansoor does not like it. He keeps to himself.

**Interviewer:** Okay and does that bother you?

**Interviewee:** Yes, lekin *iski bhi tu marzi hai*

**Interviewer:** All right, do people know about the illness in your family and your friends circle?

**Interviewee:** Yes *chupana kee kya baat hai*. There are people who say it is saya but I think it is an illness.

**Interviewer:** All right, and what other kind of problems do you face?

**Interviewee:** His brothers do not let us enter the house. After my mother in law died, they do not allow us because they say that Mansoor screams a lot. And they are very *ganday log.* They say that this is our house and we say that no we do not need the house. *Humein sahara chahye*

**Interviewer:** Okay and where do you guys live?

**Interviewee:** I have rented a room in my sister’s house and we pay money for that. My sister is single and she has to take care of our another sister who is handicapped.

**Interviewer:** Okay and what was your first reaction to the illness?

**Interviewee:** I got worried of course, but I knew it was an illness. So I said foran doctor ko dekhatain hain.

**Interviewer:** Okay who advised in seeking help?

**Interviewee:** My elder brother in law told me about Dr. Hanif Mesiya who worked at Kharadar before, so we used to go to him. Now we go to Dr. Hena. Maybe inkay haath mein shifa hogi. It is important to show the doctor. We live in a flat and he claps at times and screams. *Hosh mein hee nai hotay. Acha tu nahi lagta.* I also thought of getting a divorce from him but he said he does not have anyone and even his brothers do not help him. That is why I am continuing in the marriage.

**Interviewer:** Has your relationship changed since the onset of the illness?

**Interviewee:** Yes, we do not have any sexual relationship. We sleep in separate rooms. I have gotten very scared after he tried to hurt me. I would have died. I had to escape from him by scratching him with my nails. After that, I sleep with my sister because I cannot get over my fear. *Pehlay jaisa pyaar aur mohabat nahi hai. Aur yeh sab beemari ki waja say hai. Lekin inki bhi galti nahi hai. Allah ne mujhe nahi zindagi di warna yeh tu mujhe khatam kardetea. Mein tu shaadi zabardasti chala rahi hun. Dil ko kaisay sukoon ayega agar meiney chor diya. Aap ko lagta hai kay mujhe chor dena chahye? Mujhe sukoon tu milta hee nahi hai. Mein andar jaldti rehti hun. Khud bhi dar gaye hun. Khauf kaisay nikle.*

**Interviewer:** Has it affected your relationship with others?

**Interviewee:** Yes it has. My sisters ask me *kab tak yeh chaleyga.* We live in the same flat so they wake up when he is screaming at night, so they get annoyed.

**Interviewer:** Do you feel his mental illness has led to any mental problems of your own?

**Interviewee:** Yes. *Mujhe hurwaqt khauf rehta hai.*

**Interviewer:** Has he ever hit you apart from one episode?

**Interviewee:** *nahi kabhi haath nai uthaya.*

**Interviewer:** Okay, what is your day to day routine?

**Interviewee:** I wake up at 6 30 am. He goes to work at 7 30 am. He comes back at 6 pm. And then we have food, then we go to Jamat Khana, and afterwards we sleep around 11 30.

**Interviewer:** Have you taken any additional responsibilities post-illness of your spouse?

**Interviewee:** *Nahi, mujhe lagta hai meiney aur kam kardya hai. Dil hee nahi chahta. Hur waqt khauf laga rehta hai.*

**Interviewer:** What do you do in your leisure time?

**Interviewee:** *bachay kay saath lagi rehti hun*

**Interviewer:** what do you know about the mental illness? Have the doctors told you?

**Interviewee:** Yes they have told me but *naam zehen say nikal gaya hai?*

**Interviewer:** Okay why do you continue to stay in the marriage?

**Interviewee:** *agar mein nahi support karungi tu yeh bhatakta rehay ga. Lekin mein khush nahi hun*

**Interviewer:** Do you think it is your spouse’s fault to have the illness?

**Interviewee:** No

**Interviewer:** Has divorce been suggested to you by your family members?

**Interviewee:** *Nahi buss yeh kehtay hain kay kab tak chaleyga.*

**Interviewer:** Is the marriage more important or the family as a whole?

**Interviewee:** Family

**Interviewer:** How do you see your future?

**Interviewee:** I hope to have a good future. I want to give the best to my child.

**Interviewer:** In what situation should the couple seek divorce?

**Interviewee:** Jab husband marta ho

**Interviewer:** What do you think are the essential building blocks in raising a healthy family?

**Interviewee:** *Chup rehna chahye*

*Interview Ends*
