# Supplementary material for: Exploring effects of severe mental illnesses on marriages: A qualitative study from Karachi, Pakistan
Source: PLOS Glob Public Health. 2025 Dec 23;5(12):e0005652. doi: 10.1371/journal.pgph.0005652 (PMC12725543; doi:10.1371/journal.pgph.0005652)
Supplement: S1 Data — (ZIP) [file pgph.0005652.s001.zip › Transcriptions/Case 1 Transcripts/C1-8.docx]

**Case 1**

**26^th^ May, 2015**

**Illness:** Bipolar Disorder II

The husband did not allow the interview to be recorded. He was highly suspicious because he was a government officer. Before the interview started, he kept on saying that he does not believe that any illness will cause problems within the marriage. He is very happy with his wife and he will always support her. He also stated that this was not really an illness but his wife just feels low at times.

The following is a summary of what occurred during the interview. Verbatim is in italics.

**Interviewer:** What were the symptoms like when she was first taken to the psychiatrist?

**Interviewee:** She used to feel very sad. *Tabiat mein maza nahi hai. Chichirapana tha.* Her brother told him to take her to the psychiatrist.

**Interviewer:** All right, and when was her illness first diagnosed?

**Interviewee:** It was diagnosed in 2003

**Interviewer:** Do your parents know about your spouse’s mental illness?

**Interviewee:** Yes, and they are also very worried and they say that she has everything. Kids, a good husband, a good house and a car, so why does she feel like this? But *kher Allah behtar kareyga*

**Interviewer:** Do you feel that you have taken up additional responsibilities after the illness of your spouse?

**Interviewee:** No. she does everything. She is able to manage everything but when she is sad that is when I have to do some work. However, it is not an issue. I can cook because I learned it during my BSc so it does not matter to me.

**Interviewer:** Hmm do you think it gets stressful for you managing her illness?

**Interviewee:** Yes, sometimes it does. *Mein pareshani mein ajaata hun.* I have to go on different trips for evaluation which is why it often gets tough because then I cannot go. Since I have to take care of her.

**Interviewer:** Are doctor visits stressful to you?

**Interviewee:** *It is a job.*

**Interviewer:** Do you feel that your work gets affected due to her illness?

**Interviewee:** Yes, for example, today I am waiting since morning for the appointment, so of course I didn’t go to work

**Interviewer:** Do you feel that your support helps the patient?

**Interviewee:** yes yes

**Interviewer:** And does she acknowledge it?

**Interviewee:** Yes she does.

**Interviewer:** Do you people socialize as a couple?

**Interviewee:** Yes we go out a lot

**Interviewer:** Okay and do people question you about her illness?

**Interviewee:** Yes in family people know and they know she has low moods.

**Interviewer:** Do you feel that your relationship has changed since the onset of the illness?

**Interviewee:** Relationship has not changed *at this point, the spouse had gotten quite defensive*

**Interviewer:** What was your reaction when you found out about the illness?

**Interviewee:** My reaction was nothing. It was normal. *Disease hai Allah ke taraf sey di huwi. Sahi hojayegee.* Positive thinking is important.

**Interviewer:** Okay but you know how in our society, people can create issues when there is a mental illness. There is this stigma that oh she goes to a psychiatrist etc.

**Interviewee:** Yes, you’re right. That is true in our society but that’s not the case over here. Mujhe farq nahi parta

**Interviewer:** Okay and do your children know about the illness?

**Interviewee:** Well, they have an idea that she gets *beemar.* When I am at work, they tell me that *aaj ami pura din soye hain, tu hum unkay saath bethay thay aur sar dabaya tha.*

**Interviewer:** Has her illness affected relationship with others?

**Interviewee:** No.

**Interviewer:** Do you feel any kind of stress due to her illness?

**Interviewee:** Sometimes, there is stress but not a lot.

**Interviewer:** Does she take her medications herself?

**Interviewee:** Yes she takes them easily. Sometimes, she might delay it but she takes them always and I don’t have to tell her to take medications.

**Interviewer:** do you feel you have taken any additional responsibilities post illness of your spouse?

**Interviewee:** Not really. I do outside work and she manages inside work quite easily. So it is not an issue. I myself do not let her do a few things myself when she is not well. I can take care of it. I also take her to go and take a walk etc.

**Interviewer:** What do you do in your leisure time?

**Interviewee:** I am off on weekends but our family also has a business, so I go there on the weekends with my brother

**Interviewer:** Do you feel that you possess enough knowledge about the illness?

**Interviewee:** *God is great. Dekh lete hain.* *he didn’t know much*

**Interviewer:** Do you feel at any point that it is her fault that she has the illness?

**Interviewee:** No, not at all.

**Interviewer:** Do you feel you can fix her?

**Interviewee:** *Mein kahan say karonga. Doctor saheeh kareingay*

**Interviewer:** have you ever thought of divorce as a possible option?

**Interviewee:** No no. not at all. *“Love one, like one, hate one, yeh galat hai”*

**Interviewer:** Have you ever thought that because of the illness you would want to leave your spouse?

**Interviewee:** No no. *life hai, bachay hain, paisa hai, beemari aani jaani cheez hai. Humari mental understanding hai.*

**Interviewer:** Has anyone suggested divorce to you?

**Interviewee:** No

**Interviewer:** Okay and under what circumstances do you feel, in general that a couple should seek divorce?

**Interviewee:** *Yeh galat baat hai, Mein nahi manta isko*

**Interviewer:** Would you like to give a comment about divorce?

**Interviewee:** No, not at all. *Eladgi ka naam hi galat hai.*

**Interviewer:** How do you see your future?

**Interviewee:** Bright

**Interviewer:** is a marriage more important or the family?

**Interviewee:** Full family unit is more important.

**Interviewer:** What are the essential building blocks for raising a healthy family?

**Interviewee:** Khaana peena, asaish and good education

**Interviewer:** Okay thank you so much for your time.

***Interview Ends***
