# Supplementary material for: Exploring effects of severe mental illnesses on marriages: A qualitative study from Karachi, Pakistan
Source: PLOS Glob Public Health. 2025 Dec 23;5(12):e0005652. doi: 10.1371/journal.pgph.0005652 (PMC12725543; doi:10.1371/journal.pgph.0005652)
Supplement: S1 Data — (ZIP) [file pgph.0005652.s001.zip › Transcriptions/Case 1 Transcripts/C1-9.docx]

**Case 1**

**Husband diagnosed with Bipolar Disorder**

**Interviewer:** So this is basically a research where we are trying to help out those jo support provide kar rahay hotay hain patients ko.

*fills out demographic form*

**Interviewer:** Acha aap ko pata hai kay unko kya diagnosis diya hai?

**Interviewee:** jee Bipolar Disorder

**Interviewer:** Acha tou kitne arsay say hai?

**Interviewee:** Mujhe lagta hai unko shaadi say pehle bhee yeh beemari thi. Pheley unko ulcers thay. Tu meiney Karachi kay sarey doctors ko dikhaya. Skin specialists dentists aga khan kay doctors ko bhi check-up karwaya. Mujhe hairat ki baat yeh huwi kay kisi ko bhi peptic ulcers kay barey mein pata nahi challa. Yahni jab doctors steroids shuru kartey thay tou mein discontinue kardeti thee.

**Interviewer:** Side effects ki waja say?

**Interviewee:** Jee. Itna masla tha kay woh kha nahi saktey thay aur bol bhee nahi saktey thay.

**Interviewer:** Acha tou aap ko yeh feel hota hai kay yeh shaadi say pheley bhi yeh beemari thi?

**Interviewee:** Haan

**Interviewer:** Acha aur kya shaadi say phele unki family mein kisi ney yeh bataya tha ya nahi?

**Interviewee:** Nahi kisi ko pata hee nahi tha. Kisi ne bhee gor nahi kya. Shaadi kay baadh mein unko ulcers kay liye hakeem kay pass bhi lekey gaye. Lekin koi faida nahi hua. Phr Aga Khan kay dentist ne bhee kaha kay meiney aisey chaley kabhi zindagi mein nahi dekhey. Phr merey relative hain woh bhi doctor hain. Unhon ne bola kay kisi proper doctor ko dikhao. Unhon ne kaha kay bhai eik boht problem hai tu usko dikhana chahye hai aur unhon ne yeh missal le jo mein bhi share karleti hun kay yeh chaaley tou rehengay kyunk two qism kay log hotay hain. Eik aisay log hotay hain jin kay walid ki agr death hoti hai aur woh bhookay hotay hain tou woh kisi masjid mein jakey chupkay say kha letey hain aur eik aisey log hotay hain jo meheno rotay hain. Jab yeh phr diagnose hogaya tou …. *inaudible* phr meiney eik aur doctor ko dikhaya jo hamari community mein hotay hain tou unhon ney kaha kay psychiatric treatment karalein. Phr mein Dr. Baqar kay pass lekey gayein. Phr unkay treatment mein rehey.

**Interviewer:** Acha unhon ney diagnose kya?

**Interviewee:** Jee aur ussey pehely mein aur doctors ko bhi dikhaya. Dr Zaki, Dr Haroon …sab ko dikhaya Dr. Baqir kop hr dikhaya. Aur unhon ney bataya kay yeh problem 30 percent logo mein hoti hai aur yeh isska treatment aap ko bilkul regular karna hai aur yeh bipolar hain aur yeh bhi hosakta hai kay woh matlab extreme conditions bhi asakti hai aur phr meiney kaha kay yeh tou ub diagnose hua hai aur merein medicine karti gaye baar baar wohi treatment hota raha aur usski waja say boht cheezain kharab hoti gayein.. acha aur ussey pheley bhee shopping boht kartey thay. Unhon ney mujhey bataya kay yeh boht zyada shopping kartey hain tou usska khayal rikhyega. Phr Dr. Haroon ko dikhaya. Usse control tou hogaya. Lekin koi cure nahi hua

**Interviewer:** Acha aap cure chahteen hain kay koi dawai nahi lene parein?

**Interviewee:** Haan. Practically koi cheezain honi chahye.

**Interviewer:** Acha aap Dr. Murad ko kab se dikha rahi hain?

**Interviewee:** 6-8 months hogaye hai

**Interviewer:** Acha aur unsay pehely kisko dikha raheen thee?

**Interviewee:** Dr. Baqir.

**Interviewer:** Acha aisa kabhi hua hai kay unko pakarna para ho aur hospital mein dakhil karna para ho?

**Interviewee:** Haan jab mera beta chota tha..eik dum sey mujhe lagta tha kay unki tabiat taiz honi lagti thee. Woh different hojatey thay *starts crying*

**Interviewer:** agar aap nahi batana chahti tou koi masla nahi

**Interviewee:** Nahi, mujhe batana hai. Mera beta boht chota tha. Shaadi thi. Sab bethey huay thay. Gold pe koi bhais hogaye. Lekin mein phr unkay saath wapis agaye kyunke isliye wapis agaye kay voice mein different tha. Tou jab mein aye … yeh bhe problem hota hai kay jab aisa hota hai tou saari buri baatein yaad ajateen hain tumney yeh kaha tha..jab mein unkay liye pani lekey aye tou unhon ne lock kardiya tha darwaza. Ub mujhe tou idea nahi hosakta tha kay aisa kuch bhi hosakta tha. Shukar hai meri mother in law kay kamray mein aisa tha kay window say nazar ajata tha. Tou meinney dekha kay woh ropes nikal kar apne aap ko hang karney ki koshish kar rahay hain.. yeh 2 minute ki baat … meiney sab ko bulaya..father in law ney betey ko kaha kay tum awaaz du tou jab merey betey nay awaz de kay “baba, baba” tou phr unhon nay lock khol diya lekin 1 minute kay aisa laga kay agar woh darwaza na kholengey tou pata nahi kya hua. Tou buss yeh dar laga rehta tha

**Interviewer:** Aur yeh kitne arsay pheley ki baat hogi?

**Interviewee:** Mera beta mashAllah 24 saal ka hai aur uswaqt woh 8 years ka tha

**Interviewer:** istarah ka kabhi aur hua hai kay dakhil karana parey?

**Interviewee:** haan haan. Dr. Baqir ney isswaqt bhe foran kaha kay unko le ayein. And unko ECT bhee karna para. Koi bhee ghar mein tyar nahi tha lekin mujhe step lena para

**Interviewer:** Acha eik baar hee admit huay hain?

**Interviewee:** Nahi 3-4 martaba. ECT bhee 3 dafa hua hai

**Interviewer:** aur yeh dawaiyo pe kitney arsay say hai?

**Interviewee:** 20 saal

**Interviewer:** Acha aap ko kisi qism ki maali mushkilat hain?

**Interviewee:** Jee bacho kee fees waghera hoti hai aur phr Aga Khan ka ilaaj bhi expensive hai. 2000 rs tou fee hai. Aur phr medicine bhee utnee hai. Pheley bank waghera reimburse hojata tha jo bhi medical treatment hota tha. Lekin ub unhun ne psychiatric treatment ki reimbursement band kardi hai. Jis ki waja say kaafi mushkilaat ka samna karna parta hai halanka main problem yeh hai

**Interviewer:**  Acha aur kisi ghar walo say koi masla waghera?

**Interviewee:** nahi sab sey theek rehtey hain sirf merey hee say aisay hotay hain *laughs* Sab ka boht care kartey hain aur sab unki bhi boht care kartay hain lekin zahir hee see baat hai mujhe stand lena parta hai kay yeh nahi karna waghera tou mein kharab hojati hun aur mujh pe sara ghussa utartha hai.

**Interviewer:** Acha aur family waghera mantay hain kay unko bipolar hai? Or unko maloom hai?

**Interviewee:** haan haan

**Interviewer:** Acha aap kay waldeen zinda hain? Tou unko pata hai?

**Interviewee:**  Jee unko pata

**Interviewer:** Acha aur aap ko unki madad aur support miltee hain …ya financial support waghera ..

**Interviewee:** Nahi aisa kuch zaroorat nahi hai

**Interviewer:** Acha aur emotional support waghera milta hai?

**Interviewee:** Haan Allah ka shukar hai

**Interviewer:** Acha phr jab aap ki shaadi huwi thi aur aap ko pata challa kay unko bipolar hai tou aap ne apney waldeen ko bataya tha?

**Interviewee:** Unko discuss karkay hee dikhaya tha. And jo bhi concerned log thay, un sab ko dikhaya tha. In-laws waghera bhi. Yeh merey first cousin hain. Mamoo kay bête. Aur woh boht achi theen meri mother-in-law. Unki waja say hee ghar mein ronaq thi. Lekin kabhi koi conflict bhee nahi hua unkay saath

**Interviewer:** Acha generally aap ki life mein unki waja say stress hojata hai..aur unki condition ki waja say?

**Interviewee:** haan jee hojata hai

**Interviewer:** Acha kis tarah sey stressful hojata hai?

**Interviewee:** Acha jaisey abhi unhon ne itni shopping karle thee …11,000 ki. Aur phr kehtey hain kay meiney sarey paisay tumhein meiney dediye. Aur ubhi meri bachi LUMS mein hai aur uski fees due hai.. matlab ussay financial aid milti hai. Scholarship miltee hai lekin phr bhee acha khasa amount dena hota hai. Mein gin gin kar paisay kharch karteen hun aur woh eik minute mein ura detey hain

**Interviewer:** Overspending problem hain?

**Interviewee:** Haan aur kabhi boht zyada hi fi life hoti hai aur phr 15-20 days baad depression hojata hai. Aur merey liye asal problem hai hee yeh fluctuation. Mood ki fluctuation

**Interviewer:** Acha, aur aap kay jo baitay hain woh 24 years kay hain woh kya kar rahay hain?

**Interviewee:** Nahi woh ubh kaam kar rahay hain. Usney Lums say parha hai aur ubh woh Engro mein job kar rahay hain. Meiney yeh humeisha kaha hai kay bacho ko acha parhaon

**Interviewer:** Hmm. Acha acha Aur kis qism ki pareshaani ka saamna karna parta hai? Inki condition ko letey huay?

**Interviewee:** Wohi fluctuation ko letey huay

**Interviewer:** Aur aap ko sab say zyada..kya doctor kay pass baar baar ana frustrate karta hai?

**Interviewee:** Nahi mujhe koi cheez frustrate nahi karti. Allah ka shukar hai.

**Interviewer:** Acha aur woh dawaiyan time pe le letey hain ya unko force karna parta hai?

**Interviewee:** haan. Matlab unko pata nahi konsi medicine khani hoti hai..tou unko zabardasti deni parti hai. Dupher ki do. Kher ub tou doctor ne kaha hai kay dupher wali na bhee khayein tou theek hai. Lekin jab zaroori hoti hai tou dupher mein deni hoti hai. Lekin jab unkay kapray wash mein ja rahay hotay hain tou usqaqt pata chalta hai kay medicine pocket mein hai. Istarah

**Interviewer:** Woh bhool jaatey hain?

**Interviewee:** Nahi bhooltey naheen buss unko fikr nahi hai

**Interviewer:** Kya unko yeh pata hai kay unkay saath bipolar disorder ka problem hai?

**Interviewee:** Haan haan

**Interviewer:** Accept kartay hain?

**Interviewee:** Haan buss woh kehdetey hain kay galti hogaye

**Interviewer:** Acha aur aap ko lagta hai kay jo aap madad karteen hain unsay unko help hoti hai aapki support say?

**Interviewee:** Haan definitely hoti hai lekin agar aap unsay phoochay tou nahi. Buss woh kehtay hain kay hur waqt tanqeed aur rok tok hoti hai meri taraf say.

**Interviewer:** Acha aap ka doston mein aur family mein milna milana hota hai?

**Interviewee:** Haan haan

**Interviewer:** Aap dunu as a couple jaatey hun?

**Interviewee:** haan

**Interviewer:** acha family ko tou pata hai but doston waghera mein pata hai?

**Interviewee:** Woh khud bata detey hain. Ubhi merey bhai bhabhi kay ghar deketi huwi thi. Kaafi cheezain legaye. Meiney jakey buss batadiya kay mujhe call aye hai aur mein ja rahi hun. Lekin jab unhun nay aakey apne jazbaat daaley tou family mein sab pareshaan. Meiney kaha meiney bataya tha lekin woh kehtay hain kay tum ney itna flatly bataya *laughs* kay kuch laga hee nahi. Unhun ney bataya kay isstarah gun rakh dee thi waghera waghera ..bhabi ko aisa hua tha. Woh boht expressive hain aur unko yeh complain hai mein expressive nahi hun.

**Interviewer:** Acha aap ko pata hoga kay nafsiati beemari kay barey mein log aisa kehtey hain kay na yeh tou pagal hain waghera waghera. Tou aisa koi response kabhi milla hai?

**Interviewee:** nahi

**Interviewer:** Acha family mein nahi tou kisi bahir waghera mein?

**Interviewee:** *inaudible* nai buss family mein koi problem hota hai tou unko nahi batatey aur unpe koi decision making nahi daala jaata kyunke woh pareshaan hojatey hain

**Interviewer:** Iss waja say ghussa ata hai?

**Interviewee:** Haan bhai. Aksar aisa hota hai kay woh kehtay hain kay khana nahi khatey roti nahi khatey ..kehtay hain kay daant mein dard hai ..apni sehat ka khayal nahi rakhtey tou zahir hee see baat hai mein kehti hun. Haan tou is qism see baton sey irritation hoti hai

**Interviewer:** Acha aap ko lagta hai kay unki beemari ki waja say khandan kay mahol pe farq aya hai?

**Interviewee:** Zahir hee see baat hai merey bacho pe tou boht farq aya hai.

**Interviewer:** Acha kiss terms mein? Kya bachon ko pata hai kay unkay abbu ko bipolar hai?

**Interviewee:** haan. Doctor kay pass aatey hain. Aur meri beti bhi unko medicine deti hain.

**Interviewer:** Acha aur aap ko jab pehli baar beemari kay barey mein pata chala tou aap ka kya rad-e-amal tha? Kya khayalat huay?

**Interviewee:** Buss mujhe yeh tha kay medicine say theek hojayega lekin uskay baadh humari activity boht dull hogaye thi. Even hamara relationship bhee jo tha woh nahi raha. Lekin doctors yahi kehtey thay agar hum ney activity ko activate karnay ki koshish ki tou saari cheezain activate hojayegeen.

**Interviewer:** Aur aap kay bachon ka kya reaction tha? Or dusray khaadan walo ka kya reaction tha?

**Interviewee:** Sab boht supportive aur caring hain. Tou itna farq nahi para.

**Interviewer:** Acha lekin kabhi aap kay bacho ko feel hota hai ya kabhi woh yeh boltay hain …

**Interviewee:** unki feeling ko mein samajh jaati hun. Eik dafa yeh log jab chotay thay tou mein apni ami kay yahan thee aur unki tabiat thori kharab thee. Tou woh unhon ne kya kya kay woh kundi jo hoti hai uspe meray dunu betey aur beti ne cotton liya aur tape liya *starts crying* cover kardiya… aisa na ho kay woh lock kardein. Tou mujhe yeh boht taqleef hoti hai kay itni choti age mein ...lekin Allah ka shukar hai kay woh negativity nahi hai lekin phr bhee mujhe taqleef hoti hai kay … *keeps on crying* buss mujhe iss mein taqleef hoti hai..bachon kay hawaley say baaqi sab theek hai .. buss liekin iss waja say merey bachay mera boht care kartey hain

**Interviewer:** Acha waisey aap apni shaadi shuda zindagi ko kaisay describe kareinge? Achi, buri ya theek?

**Interviewee:** Achi hai. Jab shuru shuru mein problems hoti theen tu mein doctor say complain karti thee, tou buss unhon ne eik dafa mujhe chup karakay yeh phoocha kay aap ko saath rehna hai ya nahi… tou buss uska tou phr koi sawal hee nahi hai. Tou buss phr unhon ne kaha kay phr koi baat karni kee hai hee nahi buss unhee ko baat karne dein.

**Interviewer:** Acha aur aap ko lagta hai kay aap ka rishta mein koi change aya jab se diagnosis hua?

**Interviewee:** *pause*

**Interviewer:** Koi rishtay mein tabdeeli? Jaisay aap na kaha kay dawaiyon say kuch activities repress hogayein?

**Interviewee:** jee 15-20 saal say woh hai nahi hamari relationship mein.

**Interviewer:** Aur aapki shaadi ko 26 saal huay hain?

**Interviewee:** Jee

**Interviewer:** aur diagnosis ka pakka pakka pata 20 saal say pata chala hai?

**Interviewee:** haaan

**Interviewer:** Tou un 6 saalo mein kaisee thee relationship?

**Interviewee:** uswaqt problem tha lekin buss diagnosis nahi tha

*interruption due to phone conversation*

**Interviewer:** acha aur aap kay beech mein communication waghera hai? Jab say unki beemari huwi hai kya aap ghabrati hain unsay baat karney mein?

**Interviewee:** Unsay baatein chupani parteen hain kyunke woh sab ko jakey bata detey hain. Aur boht time say pheley bata detey hain. Ubhi hum admission key liye try kar rahay thay tou unhon ney sub ko jakey sub bataya diya tha. Khud khaitay hain kay hum yeh kareinge aur woh kareinge

**Interviewer:** Acha waisay aap dunu baatein waghera kartey hain?

**Interviewee:** Mein hee zyada karti hun

**Interviewer:** Aur aap ko lagta hai key iss beemari ki waja say aap kay dusray rishto pe kisi qism ka asr para hai?

**Interviewee:** nahi

**Interviewer:** Aap ka apne bacho say aap ka acha relationship hai aur aap kay saath ju dusray log rehtay hain?

**Interviewee:** Haan boht acha relationship hai. Balkey jo meri nand hai woh uper walay portion mein rehti hain. Tou jo bhi koi problem hoti hai mein unsay complain karti hun. Kay yeh ab aisa aisa kar rrahay hain aur phr woh akey dictate karteen hain kay yeh karlein.

**Interviewer:** Acha mein eik aur question phoongee. Aap ko lagta hai joint family system kee waja say aap ko kaafi help mill gaye hai?

**Interviewee:** Joint family system say boht positive help mili hai. Mera relationship shuru say he dusray logo say positive raha. Merey mother-in-law tou yeh kehti theen kay meri phehli bahu achi hai tou baaqi bhee achi hee ayein.

**Interviewer:** acha aap ko lagta hai unki beemari ki waji say aapko kisi qism ka zehni dabao ya uljhan ka saamna karna parta hai?

**Interviewee:** Haan who tou hur waqt rehta hai. Mujhe nahi pata hota kay ub yeh kya karney walay hain. Acha arey yeh tou mein batana hee bhul gaye. Ubhi unhon ne 2-3 lakh udhaar diya. Kuch computer waghera leney kay liye. Mera beta 3^rd^ or 4^th^ mein tha. Tou woh apney naam pe dila rahay thay aur 50,000 per month kuch deduction tha. Betey ko tou chaley dila diya but jo parosi ko kyun diya..mein ney kaha kay agar woh bhaag gaya tou? Unho ney kaha kay kyun bhaagey ga? Meiney kaha agar woh mar gaya tou… tou unhon na kaha tou kya hua.. jo hoga dekha jaiega. Merey mana karne kay bawajood karliya. Aur phr hur meeheney salary sey paisay kathay thay. Uswaqt itna amount boht ahmiat rakhtey thay. Unhon ney apne naam pe eik colleague ko bank pe loan nikal wa diya aur phr uski aap ko pata hai kitni qistey hoti theen ..aur humein pareshani ka saamna karna para. Yeh loan without guarantee. Unki marzi thee kay woh colleague paisay dein ya nahi phr paisay jama karwane kay liye mujhe ya merey dewar ko leker jayeingee. Phr woh baat kartey thay. Tou yeh wali cheezain..

**Interviewer:** acha aur aap ko lagta hai kay jo yeh unkay habits hai jo aap biyaan kar rahay hain ya beemari ki waja say hain?

**Interviewee:** Haan bilkul beemari ki waja say hain

**Interviewer:** Acha aur aap ko inki beemari ki waja say kabhi khud doctor ko dikhana para ho?

**Interviewee:** Nahi aisa nahi hua tha.

**Interviewer:** Acha aap ne kaha kay counseling honi chahye tou aap ko lagta hai kay koi buss aap ki baath sunle waghera? Koi aapki baat karle?

**Interviewee:** Haan eik Dr. Ayesha Qureishi hain..psychiatrist hain..shayad aap jaantey hun. Unko bhee dikhaya tha. Buss yeh baat hai kay unkay liye change boht zarori hai. Doctors change kartay hain. Shuru mein 6-8 meheny bilkul theek hotay hain eik doctor kay saath phr wohi change karnay ki baat kartay hain.

**Interviewer:** Acha tou who sahi hotay hain ya unko lagta hai?

**Interviewee:** nahi sahi ho bhee jaatey hain woh.. lekin jaisee medical nahi milte tou tabiat kharab hojati hai. *inaudible, mumbling*

**Interviewer:** acha aur aapki woh care waghera kartey hain?

**Interviewee:** haan care tou kartey hain. Ubhi merey liye itni shopping waghera ki.

**Interviewer:** Acha jab unki mijaaz mein tezi aati hai tou unka ghussa aata hai?

**Interviewee:** haan ghussa aata hai

**Interviewer:** acha aisa hua hai kay ghussay mein cheezain utha kay pheenki hun waghera?

**Interviewee:** Haan

**Interviewer:** kabhi mara ho tu?

**Interviewee:** Haan aisa hota hai kay woh boht rash driving kartey hain tou merey haath jo dashboard pe hota hai tou usspe martey hain… aur kehtay hain kay utar jao gari say..yeheen utar jao

**Interviewer:** Kabhi aap pe haath uthaya ho?

**Interviewee:** Ghussa mein hee jaise gaari mein ney bataya aur kehnegay kay merey saath nahi betha karu *laughs* eik tou dafa jab medicine chori thee tou haan..eik dafa hum shaadi mein gaye thay tou woh achanak itna hyper hogaye kay meiney doctor ko phone kya..balkey jo merey cousin hai.. unhon ney bola kay foran doctor ko phone karun. Aur phr woh admit huay thay aur phr ECT hotay hain. Woh kehtay hain jab ECT hota hai tou aisa lagta hai kay jaisay burden khatam hojata hai lekin yeh khuch dino kee baat hoti hai.

**Interviewer:** Hmm acha aur aap ka din kaisay guzarta hai? Aap kitna waqt deti hungi unka khayal rakhney mein?

**Interviewee:** nahi ub khair itna nahi rakhna hota

**Interviewer:** Acha aur jab naye naye beemari samney aye thi tou uswaqt?

**Interviewee:** meri mother in law kaafi karletei theen tou itni problem nahi hwui

**Interviewer:** Acha aap ko lagta hai kay aap ne koi additional zemaadariyan lee hain apni husband kee beemari ki waji say? Jo eik normally aurat nahi leti?

**Interviewee:** Caring tou boht hai. Khud hee subah uth kay chorthay thay. Lekin haan mujhe buss ye problem tha kay jo eik seniority hoti hai woh kabhi nahi thi

**Interviewer:** Acha aur aap ko lagta hai kay aap ko Bipolar Disorder kay bareey mein sab khuch pata hai? Aap online parha tha? Doctors nay bataye tha? Kisi ney aap ko properly samjhaya tha?

**Interviewee:** Dr. Baqir say isshee liye withdraw kya tha kay buss woh dawaiyan detey thay aur samjhatey nahi thay.

**Interviewer:** Acha aap ne kaha tha kay dr. Baqir ne aap say phoocha tha elaidgi kay bareey mein…

**Interviewee:** Nahi eledagi kay barey mein nahi…. Woh boht fine personality kay aadmi thay. Unho ney yeh phoocha tha kay aap ko saath rehna hai. Meiney kaha kay haan. Mujhe buss unhon ne yeh bataya tha kay kuch cheezon mein aap hee ko taqleef hogi aur aap ko chup rehna hoga. Jisskay pas bhee aap rehengi, chot aap ko hee ayegi.

**Interviewer:** Acha aap ne kabhi bhee eladgi kay barey mein nahi socha?

**Interviewee:** Nahi nahi

**Interviewer:** Acha aap ko kya lagta hai kiss soretahal mein eladgi kay barey mein sochna chahye?

**Interviewee:** Mein tou kehti hun kay kabhi bhi nahi sochna chhaye. Kyunke kaheen na kaheen Allah hee nay aap kay liye socha hai. Theek hai. Aapko option bhee diya hai. Aur phr bachay jo hain naa. Aap agar unkay liye itna nahi karsaktey tou phrr ….

**Interviewer:** Hmmm…acha jab unki beemari huwi thi tou aap kay dunu bachey paida hochokay thay?

**Interviewee:** Jee haan. Beti ..haan ..hochuki hogi

**Interviewer:** acha bacho kee waja say aap ko lagta hai kay main reason hai kay aap ne eladgi kay bareey mein nahi socha?

**Interviewee:** Nahi meiney kabhi socha hee nahi

**Interviewer:** nahi generally kya aap ko lagta hai kay bacho kee waja say hee eladgi nahi ikhtiar karni chahye?

**Interviewee:** Haaan. Haan eik kee waja say 3 aur logo ko tu suffer nahi karna chahye. Buss apna nafs control karna chahye

**Interviewer:** acha aur aap ko lagta hai kay aap unko kisi tareeqay say theek karsakti hain? Kay mein theek kardungi? Kay buss ilaaj chordo aur mein theek kardungi?

**Interviewee:** Nahi aisa tou nahi hosakta na. Mein buss yeh koshish karti hun kay woh walk karein. Masla yeh hai kay yeh pehle khud hee boht active hotay thay. Lekin buss inko har jaga khaana waghera ka shauq tha. Kharadar ka khana aur achi say achi jaga ka aur mein unko mana karti thee. Balkey merey bhai kehtey thay kay kahan gaye tumhare sarey nakhray *laughs*

**Interviewer:** Acha aap ko kya lagta hai eik shaadi shuda joray kay beech mein jo relationship hoti hai woh zyaada important hoti hai ya family puri zyaada important hoti hai?

**Interviewee:** Nahi.. buss mein unko yehi kehti hun kay mein jo bhi karti hun aap ki waja say karteen hun. Aap kay ami hain. Aap kay abbu hain. Aap ki family hai. Aap kay liye karteen hun

**Interviewer:** Acha aap batana chahenge kay aisee konsi wajoohat hain jin ki waja say aap shaadi ko barqarar rakhti hain? Bacho ki waja say?

**Interviewee:** nahi nahi

**Interviewer:** aisee koi jaati wajah hai jiske waja say iss soch mein boht mazbooti aye hai?

**Interviewee:** Buss yeh baat hai kay meri unsay shaadi huay hai, meray unsay bachay hain .. woh beemar hain.. agar woh doosri shaadi kartey hain ya kisi aur say involve hotay hain tou theek hai *looks slightly offended*

**Interviewer:** Acha actually boht sarey log hotay hain kay nafsiati beemari hai tou chor detey hain isliye phooch rahay hain. Aap bura na maney.

**Interviewee:** Haan buss yehi baat hai. Agar woh beemar hain tou kya karein?

**Interviewer:** acha aap ko kya lagta hai kay eik pur sukoon khandaan aur healthy family kay liye kya cheezain zaroori hain?

**Interviewee:** fair relationship ho. Dusra yeh communication ho.

**Interviewer:** Aur aap apna future kaise dekhti hain? Bachay barey hogaye hain, tou aagey kya future dekhti hain?

**Interviewee:** Buss mujhe yeh hai kay bachay aur parh lein. Bachi meri LUMS mein third year mein hai tou woh dono aur parhein aur phr mein unkay faraiz say farig hojaon. Aur apney liye yeh hai jab tak Allah ne sehat aur zindagi de tou, na umeedgi merey nature mein nahi hai. Isska credit merey father ko jaata hai

**Interviewer:** Acha aur aap ko lagta hai kay agar nafsiati beemari problem ho tu marital counseling help karegi? Aap ne marital counseling kay barey mein suna hai?

**Interviewee:** haan meiney suna hai aur meiney sab cheezain try bhee karein hain. Reiki waghera. Kuch nahi chora. Expensive hojata tha. Dusri baat yeh hai kay 3^rd^ 4^th^ times jayeingee lekin phr nahi jayenge tou phr kya karein

**Interviewer:** Eik aur sawal yeh hai Aap ko kya lagta kay religion ka koi kirdaar hai..kay kuch saya hai ya jinn hai..kay mujhe aiseee koi madad mil sakti hai?

**Interviewee:** Nahi aisa kuch nahi hai. Log kehtey hain aur Allah kay qalaam mein takaat kisi aur cheez mein nahi hai.

**Interviewer:** lekin aap saya waghera pe belief rakhti hain?

**Interviewee:** Nahi

**Interviewer:** Aap kuch add karna chahenge?

**Interviewee:** Mujhe lagta hai kay jo mein treatment karney aati hun wahan main counseling ki zaroorat hai. Matlab mujhe problem hoti hai tou mein apni nand ho kehti hun. Tu woh unko samjhatee hain. Kyunke jab inka yeh wala phase aata hai tou yeh bilkul nahi suntey. Ubhi unko doston kay saath buss Attock jaana tha. 15 din kee choti bhee leli. Unho ney kaha tum bhi chalo but meiney kaha kay mein nahi aasakti ghar chor kay aur job chor kay. Agar jaana hai tou baadh mein chalenge lekin unhon ney suni..ticket waghera sab kar kay agaye. Shopping ki gift kay liye 35000 kay liye. Junaid Jamshed say jakey sub kuch leliya. Phr baadh mein jab wapis aye tou kehtay hain kay tum roq leti mein tou beemar tha ..meiney roka tha lekin buss jab unka jaana tha tou woh chaleyegaye. Ub kya keh saktein hain?

**Interviewer:** Yeh jo beemari hai, zahir hee see baat hai fluctuation aati hai lekin kabhi aisa hota hai kay aap ki bilkul normal relationship hoti hai? Matlab theek hojatein hain?

**Interviewee:** Haan aisee baat nahi hai. Hum eik dusrey kay uper dependant hain. Aisee baat nahi hai. Buss thori see problems yeh hain kay baaz aukaat mein unko time nahi desakteen jistarah..agar meri mother ki tabiat kharab hai ya waghera.. agar mein kabhi ghar ko time de rahi hoti hun tou unko lagta hai kay unka time kam horaha hai..matlab boht demanding hain.

**Interviewer:** Eik last sawal phoochnge. Aap ko kabhi yeh pareshani hoti hai kay merey bachay mein yeh agay ayega? Yeh bipolar?

**Interviewee:** haan meiney boht dua mangi thi aur Allah ka shukar hai ubhi tou nahi pata lekin meiney isskiliye tou meiney boht jhoot bolein kay ECT kay barey mein kisi ko nahi bataya. Kisi ne yeh bhee phoocha kay electric shock tou nahi laga lekin mein ney kaha nahi..kabhi kisi ko nahi bataya. Openly nahi discuss karna chahye. Merey dewar ki jab shaadi huwi tou unkay jo father in law hain unko bhi yeh problem hai. Arranged marriage hai lekin koi masla nahi hai…

**Interviewer:** Aap ko kabhi yeh pareshaani hoti hai kay aap kay bacho mein yeh tendency aye hai? Ya koi symptoms? Dar lagta hai agar aisa kabhi ho?

**Interviewee:** Buss Allah pe bharosa hai kay agar eik taraf say takleef de hai tou dusri taraf say thanda kareyga. Matlab beemar tou koi bhi hosakta haina. Agar bipolar hai tou Allah na karein ..face tu karna pareyga. Matlab ubh yeh aisee baat hai jo mein bacho say nahi karna chahongee lekin meiney kabhi is baat pe bhi kabhi kisi se discuss nahi kya shaadi ki koi life nahi. Matlab kisi ko nahi pata. *voice gets shaky* matlab uski koi zaroorat nahi hai..two bachey hogaye hain. Theek hai..lekin kehney ko tou hota hai.

**Interviewer:** Hmm theek hai. Thank you.

**Interviewee:** Acha hua na mera bhee gubaar waghera nikal gaya. Mein boht behtar mahsoos kar rahi hun

**Interviewer:** Jee boht boht shukrya.

***Interview Ends***
