# Supplementary material for: Exploring effects of severe mental illnesses on marriages: A qualitative study from Karachi, Pakistan
Source: PLOS Glob Public Health. 2025 Dec 23;5(12):e0005652. doi: 10.1371/journal.pgph.0005652 (PMC12725543; doi:10.1371/journal.pgph.0005652)
Supplement: S1 Data — (ZIP) [file pgph.0005652.s001.zip › Transcriptions/Case 2-6 Transcripts/Case 2/C2-1.docx]

**Case 2**

Note: The recording hung in the middle and the data was lost. However, the information was quickly written down to preserve the data. The remainder of the interview was recorded. The interview was done in two parts because the patient had to leave for her appointment.

Patient had schizophrenia and was recruited from the clinic. Initially, she had problems of depression before the marriage, but the diagnosis of Schizophrenia occurred after the marriage.

**Interviewer:** when did you separate?

**Interviewee:** 2 years after getting married.

**Interviewer:** when did you find out about illness?

**Interviewee:** I had depression before but I got cured of depression back in 2007. Then I found out I had schizophrenia in 2011.

**Interviewer:** How long was it after getting married that you were diagnosed?

**Interviewee:** Around 1.5 years after getting married, I was diagnosed with schizophrenia.

**Interviewer:** Did your spouse’s parents know about the illness?

**Interviewee:** No, they did not know.

**Interviewer:** Did your parents know about the illness?

**Interviewee:** Yes they did

**Interviewer:** What was your first reaction to the illness?

**Interviewee:** Mujhe boht duray partay thay. Mein bilkul out of control hogaye thi. I got very emotional

**Interviewer:** What was your spouse’s reaction to the mental illness?

**Interviewee:** Well, he never said anything to me directly. But he used to say bad stuff about my parents and he also said to my parents “Aap ne mujhe apni pagal beti dedi hai”. There was a lot of emotional torture. He never hit me, but there was emotional psychological torture all the time.

**Interviewer:** Okay did you get support from your spouse?

**Interviewee:** he did not give me any support at all. Only my parents support. In fact, my father bought a house as well, because my husband demanded that if I would remain married to her, you will have to give me a house in Clifton or Defence and you would also pay for the household expenses. My father got us a house in North Karachi. I paid for the household expenses with my savings from the time I had done my house job. He always used to torture me that if I leave you, what will happen to your sisters? Who will marry them? I am the oldest of all the daughters.

**Interviewer:** How often did you guys go out to socialize?

**Interviewee:** We did not go out because my husband did not want anyone to find out about the illness.

**Interviewer:** All right, how would you describe your life before the mental illness occurred?

**Interviewee:** well, we did not have that much of a difference. We also lived in separate rooms. We simply did not have a proper life. I also did not want to get married to him. I am much at peace now because I knew it would not work. Even after the engagement, I used to tell him we will not work out but he told me that he would. I also told my parents that I did not want to get married. Our relationship was already quite bad.

**Interviewer:** how did your relationship change since the onset of the illness?

**Interviewee:** We never had a good relationship. He always used to then say to me “What will happen to you once I divorce you?”

**Interviewer:** All right, and how did it affect your relationship with others?

**Interviewee:** It did not. My illness often made me beat my son, so that was an issue with my husband.

**Interviewer:** all right and what actually led to the divorce?

**Interviewee:** Well, I was alone in my apartment one night. My in laws did not like me so my husband used to be with them. I called him at 3 am in the morning and he did not come. I had a screaming fit; I was out of control. I called him and he did not come until a long time and I was beating my son and that is when he got very mad and he gave me the first divorce. Even I used to get very angry.

*recording now begins after the break in the interview*

**Interviewer:** Aap ko lagta hai kay agar aap ghussa kum karteen ya unki taraf say thori cooperation hoti tou aap shaadi bacha sakteen?

**Interviewee:** Jee jee.

**Interviewer:** Acha woh doctor kay saath aap kay pass aatey thay?

**Interviewee:** Nahi.

**Interviewer:** Kahbhi bhi accompany nahi kya?

**Interviewee:** Eik baar bulaya tha doctor ney unko. Tou ghar mein tou itnay buray hulayay mein rehtay thay kay unko insaan dusri nazar utha kay na dekhey. Bilkul hee aisee halaat mein kay jaisay koi worker ya mazdoor nahi hota. ustarah kay hulaye mein rehtay thay. But jab woh doctor kay pass aye tou tie phen kay briefcase lekey ,..jaise koi boht barey businessman nah un jinko dekh kay kuch acha hee samjha jaye..aisa huliya bana kay aye. Kay doctor kay uper impression parey. Job waghera thee nahi. Aisa hulaya bana kay aye jaise kay boht busy hain. Dhoka deney kay liye jab kay ghar mein boht hee buray hulaye mein phirtay thay.

**Interviewer:** Acha, aap ko kya lagta hai..matlab aap ney kabhi socha hai kay aap ko bemari kis waja say huwi hai ya kya reason hai?

**Interviewee:** Bachpan say hee mein hasas thi. Lekin mujhe nahi pata kay mujhe bachpan say beemari hai. Choti choti bataon pe mein 2 2 ghante ro rahi hoti.

**Interviewer:** Aap ko kabhi lagta hai kay iss mein aap ka qasur hai ya buss hai bemari?

**Interviewee:** buss mein yeh sochti hun kay mujhe hee kyun honi thee.

**Interviewer:** lekin aap ko yeh tou nahi lagta na kay aap ka koi qasoor hai?

**Interviewee:** Nahi

**Interviewer:** Acha aap yeh bata rahi theen kay jab aap kay ex-husband jab nahi kar rahay thay tou aap kay parents aap ko support kar rahay thay, tou koi maali muskhilat theen?

**Interviewee:** Shaadi kay 1 saal tak mein apne shauhar key saath reh rahi thi. Meray pass six months jo meinney house job ki thi uski sari savings theen. Wohi sari meiney kharch keen. Meray shauhar mujhe koi kharcha nahi detey thay.

**Interviewer:** Aur parents detey thay?

**Interviewee:** Jee

**Interviewer:** aap kay shauhar kay friends waghera beemari kay barey mein phuchtay thay? Kyunke aap bata rahi theen kay aap kaafi cheekteen theen?

**Interviewee:** mujh say tou nahi phuchtay thay. Merey husband say phuchtay thay

**Interviewer:** haan aap kay husband say phuchtay thay tou unka kya jawab hota tha?

**Interviewee:** Mujhe nahi pata. Meri gair maujdigi mein phuchtay thay.

**Interviewer:** Aap ko kya lagta hai eik shaadi shuda joray ko konsi sorehtehal mein alaidhgi ikhtiar karni chahye?

**Interviewee:** Merey tou khayal mein alaidhgi tou ikhtiar karni hee nahi chahye. Shaadi mein bilkul meri marzi nahi thi. Mujhe merey shauhar bilkul pasand hee nahi thay. Meri marzi kay khilaaf meri shaadi huwi thi. But jab meri shaadi huwi tou meiney kaha kay mein usko nibahungi. Mera pura iradha tha kay yeh shaadi kamyaab jaye. Lekin shaadi kay shuru week mein hee mujhe pata chal gaya kay yeh nahi hoga. Meiney namazo mein ro ro kay dua mangi kay meri shaadi chal jaye. Agar mujhe 1 meheney mein talaaq mil gaye tou kya log kahaingay. Ubhi shaadi huwi aur talaaq hogaye. Mein ney manatee mangi huwi theen kay 1 meheney tou shaadi chal jaye.

**Interviewer:** Waisay aisee kon si soretahal hokay du log talaaq lein?

**Interviewee:** Jab woh bilkul hee eik dusray kay saath cope up nahi kar pa rahay hun. Matlab misunderstanding horahi hai. Understanding bilkul hee na ho.

**Interviewer:** Aap kay husband ne khud talaaq de thi ya aap ne mangi thee?

**Interviewee:** Meiney mangi thee tou unhun ne de di thee.

**Interviewer:** Acha, aap na kaha tha kay phele talaaq unhon ne de di.. tou woh ghussay mein de thi?

**Interviewee:** Nahi. Meiney boht zidain ki theen tou unhun nay kaha acha theek hai de raha hun.

**Interviewer:** Baaqi papers kay through thi ya zabani thee?

**Interviewee:** Written ubhi tak nahi de hai.

**Interviewer:** aur aap ko kisi nay maslan aap ki dost ya family mein kisi ney kaha tha kay elaidgi ikhtiar karlou?

**Interviewee:** haan shaadi kay eik mehney baad hi meri doston aur family na kaha kay jab woh itna bura hai tou chordo ussay chordo ussay. Tou meiney kaha kay phele tou meri marzi kay khilaaf shaadi karwaye aur ubh jab mein shaadi nibhana chahti hun tou aap mujhe force kar rahay hain kay mein chordun ussay.

**Interviewer:** Aap ko kya lagta hai eik sahet mand aur khushaal family ko parwarish karney kay liye kya cheezain zarori hoti hain?

**Interviewee:** Larai jhagra tou bilkul bhee nahi hona chahye. Ussay bachon ki nafsiat pe boht bura asr parta hai. Ubhi bhee yeh (pointing to her child) apne baba say milney jaata hai kay papa ganday haina tou yeh kehta hai kay nahi papa ganday nahi hain..tou mujhe boht dukh hota hai kay kaash hum alag na huwe hotay tou ussay suffer nahi karna parta. Ub yeh bara hoga tou yeh kya sochayga kay merey papa nahi hain or istarah say..

**Interviewer:** Acha aap ne marital counseling kay barey mein sunna hai?

**Interviewee:** Haan

**Interviewer:** Aap ko lagta hai kay aap ko marital counseling milti tou aap ki shaadi mein behtri aati?

**Interviewee:** jee jee.

**Interviewer:** aur agar mental illness ho tu marital counseling help karsakti hai?

**Interviewee:** Jee

**Interviewer:** Acha, merey sawalat ko khatam hogaye. Agar aap ko iss hawalay say kuch add karna ho tou?

**Interviewee:** Nahi.

**Interviewer:** Boht boht shukria aap ne waqt nikala.

***Interview Ends***
