# Supplementary material for: Exploring effects of severe mental illnesses on marriages: A qualitative study from Karachi, Pakistan
Source: PLOS Glob Public Health. 2025 Dec 23;5(12):e0005652. doi: 10.1371/journal.pgph.0005652 (PMC12725543; doi:10.1371/journal.pgph.0005652)
Supplement: S1 Data — (ZIP) [file pgph.0005652.s001.zip › Transcriptions/Case 2-6 Transcripts/Case 2/C2-2.docx]

**Case 2**

**Outpatient:** Dr. Murad

**Bipolar Disorder**

She did not allow the interview to be recorded.

**Interviewer:** When did you get married?

**Interviewee:** I got married when I was 15 years old, and the marriage lasted for around 26 years.

**Interviewer:** Okay and who supports you financially?

**Interviewee:** I am the only earning member in the family. And I have real estate investment work so I get my income from there and I use my savings, as well.

**Interviewer:** When were you first diagnosed?

**Interviewee:** My mood swings started after I got married

**Interviewer:** When was the diagnosis done?

**Interviewee:** 20 years back, which was in 1998. However, when I was a child, I always felt disoriented and depersonalized.

**Interviewer:** What do you feel was the trigger for your mental illness?

**Interviewee:** I think it was getting married

**Interviewer:** Have you ever been hospitalized?

**Interviewee:** Yes I was hospitalized in July 2014

**Interviewer:** When did you separate from your husband?

**Interviewee:** We separated in 2011.

**Interviewer:** What do you feel was the cause of your diagnosis?

**Interviewee:** My marriage. My husband and in-laws were very abusive. They used to beat me up as well. And it was like as if *there was a lava inside me*. After I got pregnant, I got extremely depressed, which later developed into severe Obsessive compulsive disorder. I went to Dr. Onaiza. I used to sit at one place and I did not even take care of my daughter. My current diagnosis is however bipolar disorder and I get severe mood swings.

**Interviewer:** Okay and what was your husband’s attitude towards your illness?

**Interviewee:** My husband supported me as far as the illness was concerned. He was good to me and he used to take me to the doctor.

**Interviewer:** So the mental illness was not the cause of the separation

**Interviewee:** No, it was not. My husband later on got into bad habits such as extra marital affairs. He also used to go to prostitutes. He got into bad company and this is what eventually led me to seek divorce.

**Interviewer:** Did your in-laws know about the illness?

**Interviewee:** No they did not know about the illness.

**Interviewer:** Okay and what about your parents?

**Interviewee:** Yes they knew about my illness but I was stigmatized. I was accused of hysteria. And everyone said it was a drama. Later on, my daughter grew up and she also called me mad.

**Interviewer:** what was your reaction when you found out about the illness?

**Interviewee:** My first reaction to the illness was that I found *sukoon.* Because I had found an answer to my problems. I realized I could be treated. Dr Onaiza explained the illness to me quite well and she also explained very well to my husband.

**Interviewer:** What was your husband’s reaction to the illness?

**Interviewee:** He did not say much. He was supportive since he also used to take me to the doctor from Hyderabad to Karachi.

**Interviewer:** Do people in your family know about the illness?

**Interviewee:** Yes they know and they are quite embarrassed about it, as well.

**Interviewer:** Okay and what was your family’s reaction to the divorce?

**Interviewee:** Everyone knew about his character so it was not a surprise to anyone.

**Interviewer:** What was your children’s reaction to the divorce?

**Interviewee:** They supported me throughout and they also take care of my medications and take care of me quite a lot.

**Interviewer:** Okay and what is your support system like?

**Interviewee:** There is emotional support but no financial support. My brother, sister and mother are all in the United States.

**Interviewer:** Okay and did you as a couple ever socialize?

**Interviewee:** No my husband was very strict. *Hasrat he reh gaye.* He used to go abroad but he never took her along.

I feel very worthless at times remembering the torture I went through. He used to scold me a lot. My husband was not good to my daughters which is why I left him, so in a way it is a good thing that separation happened.

**Interviewer:** Okay and who encouraged you to seek treatment?

**Interviewee:** I always used to tell my mother to take me to the doctor but she never took me and eventually my husband took me.

**Interviewer:** Why do you think the illness occurred to you?

**Interviewee:** Well I was like this since childhood. My mother never paid attention to me. So I felt abandoned and I think it started from there. And then my abusive marriage. They kicked me out of the house three times. My husband also used to beat me up. *I was very kamzoor* during that time,

**Interviewer:** What are your views regarding divorce?

**Interviewee:** I thought divorce was a taboo *insaan marjaye lekin divorce na le.* But my husband eventually divorced me. But now I have realized it was actually good. When religion allows it, why not? I also think that why did I not take divorce before? I am very happy now.

**Interviewer:** Okay and the divorce was his idea?

**Interviewee:** It was entirely his idea, and he had kicked me out of the house.

**Interviewer:** Okay and why did you not initially seek divorce?

**Interviewee:** My family discouraged it. They told me that I should just compromise. This is back in 1986 when my husband used to beat e up. I said that please disown me but let me take divorce. Just send me to boarding and I will still be happy. But they did not agree. And I blame my family a lot.

**Interviewer:** What are the essential building blocks for raising a family?

**Interviewee:** Everyone should take care of each other. They should share happiness and there should be no fights and shouting

**Interviewer:** Do you think marital counseling would have helped in your situation?

**Interviewee:** Yes it would have helped quite a lot.

**Interviewer:** Do you have something to say?

**Interviewee:** Yes I feel that there are a lot of wrong marriages in society. Parents should give attention to the character of the spouse properly. Marriage is an integral part of life. I know that my daughters have an issue because of separation. Everyone thinks *shaadi is a solution for everything* so this concept needs to be changed.

**Interviewer:** Okay and do you think one should tell about the mental illness before getting married?

**Interviewee:** no, because we live in Pakistan. *Shaadi hogaye baad mein dekha jayega.* If the husband knows, then he will take advantage of the girl and the girl can be mistreated.

***Interview Ends***
