# Supplementary material for: Exploring effects of severe mental illnesses on marriages: A qualitative study from Karachi, Pakistan
Source: PLOS Glob Public Health. 2025 Dec 23;5(12):e0005652. doi: 10.1371/journal.pgph.0005652 (PMC12725543; doi:10.1371/journal.pgph.0005652)
Supplement: S1 Data — (ZIP) [file pgph.0005652.s001.zip › Transcriptions/Case 2-6 Transcripts/Case 2/C2-3.docx]

**Case 2**

**Diagnosed with Bipolar Disorder**

**Divorced**

The patient did not allow the interview to be recorded. There was no primary caregiver accompanying her. The entire exchange more or less occurred in Urdu but a summary is presented over here. The verbatim is in Italics. The patient was in a hurry as well so the interview lasted approximately 30 minutes. Due to time shortage, further probing which would have been extremely beneficial could have been done.

**Interviewer:** When was your illness first diagnosed?

**Interviewee:** It was diagnosed around 2005 to 2006

**Interviewer:** When did you get married?

**Interviewee:** I got married in 1998

**Interviewer:** And when did you separate?

**Interviewee:** I separated in 2007. The illness was diagnosed during the time when I was asking for divorce

**Interviewer:** Who encouraged in seeking help initially?

**Interviewee:** My brother is a doctor so he took me to Dr. Haider Naqvi and I was under treatment for a long time over there

**Interviewer:** What are your symptoms like?

**Interviewee:** I just get fits of anger a lot

**Interviewer:** All right, did your spouse’s parents know about the illness?

**Interviewee:** No

**Interviewer:** What was your first reaction to the illness when you found out?

**Interviewee:** Jo bhi ho merey saath dhoka hua tha. Problems were already pre-existing in the marriage before the diagnosis. He used to beat me up and the family was also very bad. They said that they are very rich whereas they were not. He physically and mentally abused me and tortured me a lot which is why I got this illness.

**Interviewer:** Okay, and did he ever question you about the illness after the diagnosis had been made?

**Interviewee:** I used to go to the doctor in front of him. There were problems existing in his family as well, as far as mental illness was concerned, so he knew about it.

**Interviewer:** Okay and what was your marriage like?

**Interviewee:** Well, it was very bad because he was very abusive. And then I had children so I stayed back but when it got too much, then I went to my parent’s house. My mother passed away three to four years back. I tried to compromise but then it got too much. Of course, I cannot share everything with you. I have shared this with my doctor.

**Interviewer:** Hmm yes that is all right, so do you think you have emotional support from your family now?

**Interviewee:** Yes everyone supports me a lot.

**Interviewer:** DO your children know about the illness?

**Interviewee:** Yes now they understand it a bit. And my children suffer a lot during my mood phases. I left my marriage because my husband used to beat up my children a lot.

**Interviewer:** Okay and what was your ex husband’s reaction to the illness?

**Interviewee:** He used to say that *tumhein doray partey hain. Eik aur excuse milgaya tha unka.*

**Interviewer:** Okay do you often go out to socialize?

**Interviewee:** No I prefer staying inside and I don’t like anyone coming in my house either. I just want to be alone

**Interviewer:** What was people’s reaction to the divorce?

**Interviewee:** You know the usual. And of course, when I used to see my cousins and siblings married and happy, so I used to get very upset. I wanted to marry again but I didn’t because of my children. *Merey saath kyun hua. Pehele mein aisee thee kay mein choti choti khushyan celebrate kartee thee lekin ubh mein nai karte.*

**Interviewer:** Okay and why do you avoid going out?

**Interviewee:** Everyone says I have gained weight and the fact that they use the word ‘fat’ is very annoying.

**Interviewer:** Did you and your ex husband socialize as a couple?

**Interviewee:** Yes we used to socialize

**Interviewer:** And once the illness was diagnosed?

**Interviewee:** Then they used to make the excuse that she is ill and then people started asking that why is she always ill. And I don’t know what else he did.

**Interviewer:** Do you think it has impacted your relationship with others?

**Interviewee:** Yes, *bacho aur ghar walon ne boht suffer kya hai* *laughs*

**Interviewer:** Did your ex husband provide any support at all?

**Interviewee:** No he didn’t. He was very abusive. He used to use street language with me. *Ubhi bhee bachon ko poison karte hain merey khilaaf.*

**Interviewer:** Does he talk to the children?

**Interviewee:** Yes

**Interviewer:** Does he financially support them?

**Interviewee:** thora boht

**Interviewer:** hmmm

**Interviewee:** Buss it was a very bad relationship. He was also involved with another woman. This is why I took this step. *Hur kisi burai mein woh thay.*

**Interviewer:** Okay do you feel you could have saved your marriage somehow?

**Interviewee:** *mujhe rakhnee hee nahi thi. Aisee relationship mein kon raheyga?*

**Interviewer:** Why do you think this illness happen to you?

**Interviewee:** *pause* *merey halaat ki waja say.* Before I got married my sister had some marital problems so I got very depressed and my family took me to Dr. Haroon and he said that there is nothing wrong. He said *shaadi kardu.* I was also of the marriage age at that time. *Phr shauhar aisa nikla*

**Interviewer:** Did you tell your husband about this episode of depression?

**Interviewee:** Haan I told him and his was one other thing he held against me. This problem was present in his family as well but the mentality of the men over here is such that *Khud jaiseey bhee hain aurton ko aisay accept nahi kartay.*

**Interviewer:** And under what circumstances do you think that a couple should think of divorce as an option?

**Interviewee:** *jab bilkul guzara hee nahi horaha hai. Jaisey meiney apney aap ko marney kee bhee koshish ki*.

**Interviewer:** Was it your decision to go for the divorce?

**Interviewee:** Yes, I didn’t want to go

**Interviewer:** What do you think of marital counseling if such problems exist?

**Interviewee:** *nahi woh normal logo kay liye kaam karsaktee hai. Hum jaisey logo kay liye nahi.*
