# Supplementary material for: Exploring effects of severe mental illnesses on marriages: A qualitative study from Karachi, Pakistan
Source: PLOS Glob Public Health. 2025 Dec 23;5(12):e0005652. doi: 10.1371/journal.pgph.0005652 (PMC12725543; doi:10.1371/journal.pgph.0005652)
Supplement: S1 Data — (ZIP) [file pgph.0005652.s001.zip › Transcriptions/Case 2-6 Transcripts/Case 2/C2-4.docx]

**Case 2**

**Schizophrenia**

**Ward**

The patient did not allow the interview to be recorded. Notes were made. Specific verbatim is in italics

She had been married twice. Her first marriage ended because her in-laws were cruel towards her. Her illness was diagnosed after her first marriage. She doesn’t have any children, although she mentioned she would like to adopt. She was 21 years old when she first got married. Her first mother-in-law was a lot into black magic and according to her, they made her eat something which forced her to tell she was suffering from a mental illness. She was suspicious of black magic. Her first marriage ended because her ex husband had a girlfriend back in America and his mother had forced him to marry with her. 2^nd^ marriage ended because her father did not give the property in her name, and her second husband was ‘lalchi’ or ‘greedy’ according to her father and he forced her to leave him. However she mentioned that she was happy with her second husband, better than she is at the moment. She seemed confused at that time, as well. Her illness was diagnosed after her first marriage. And her in-laws knew about it because she told them herself and this is the mistake that she made She did not want to tell them. Her parents also knew about the illness although her mother has now passed away. Moreover, when she heard about the illness for the first time, she was very shocked and very disturbed. Her first thoughts were *zindagi kaisay guzraygi.* Her mother at that time supported a lot and her paternal grand mother also helped quite a lot. Her second husband did not know about the illness either before the marriage and she mentioned that she also did not know that he suffered from diabetes and when he found out, he was not very happy about it. And as far as her family’s reaction goes, it was very bad. In her words *oh my god it was terrible, family rishteedar*. They were terrible. They used to force her to go to functions after her first marriage broke off so she could get another husband. *Mazaq bana liya tha, I didn’t like it.* When asked about the level of support, she said that financially, her father supports her but emotionally, she does not have anyone. She mentions he was always quite aggressive. Her father wanted to get married himself but he felt that he had a responsibility towards his daughter, which is why he did not. *Yeh sar par bethi hai.*

Socialization was present in both 1^st^ and 2^nd^ marriage, especially in the 2^nd^ one. And no one knew about her illness so there were not a lot of questions. Her relationship changed once her second husband found out about the illness. The second one said *mujh sey chupaya kyun.* Her parents used to fight a lot when she was young. Her illness was diagnosed when she went to a gynecologist because she was unable to conceive and she recommended the patient to seek psychiatric help. When asked about the reasons for her two divorces, she said the first marriage ended because of incompatibility, whereas the second one ended because of her father. When asked as to why the illness occurred to her, she mentioned that *honestly, I don’t know.* She feels marriage counseling does not work because she went to Dr. Uzma Ambareen for counseling and felt that it was not that helpful.
