# Supplementary material for: Exploring effects of severe mental illnesses on marriages: A qualitative study from Karachi, Pakistan
Source: PLOS Glob Public Health. 2025 Dec 23;5(12):e0005652. doi: 10.1371/journal.pgph.0005652 (PMC12725543; doi:10.1371/journal.pgph.0005652)
Supplement: S1 Data — (ZIP) [file pgph.0005652.s001.zip › Transcriptions/Case 2-6 Transcripts/Case 2/C2-5.docx]

**Case 2**

**Psychiatric Illness: Schizophrenia**

**In-patient**

*fills the consent form and the demographic form --- She got married in 1992 and got separated in 2002. She has done Intermediate and does not work currently. She lives alone at the moment. She has three children (two sons and one daughter). Her ex-husband had done B-Com. She was born in 1954 so she is 62 years old. She was admitted in the ward for six days at the time of the interview. She has been admitted numerous times in the hospital due to her illness. She was diagnosed after five years of the marriage. She went to *dargahs* as well for her illness. She suffers from financial problems, as well. She is currently looking for a job so she can take care of herself. And she feels lonely at home. She mentions that she used to first spend her time with her children and doing household chores and now there is no one living with her so she feels that a job will help her out. She feels it would be a good way to pass her time.

**Interviewer:** Aap ko beemari shaadi kay baad huwi thi?

**Interviewee:** Jee

**Interviewer:** Tu aap kay husband ko nahi pata tha?

**Interviewee:** Yeh problem shaadi kay baad mujhe huwi thi. Pheley nahi thi. Thori boht problem hotee thee, lekin asal problem pregnancy kay waqt hua tha. Second pregnancy kay waqt. Phr hum ne check-up karwaya.

**Interviewer:** aap ko shaadi kay kitnay arsay baad beemari kay barey mein maloom hua?

**Interviewee:** 2000

**Interviewer:** yahnee 8 saal kay baad. Uswaqt aap kay bachay thay?

**Interviewee:** Jee 2 bachay thay.

**Interviewer:** Acha aap kay jo ex husband hain unkay parents ko aap ki beemari kay barey mein maloom tha?

**Interviewee:** Unkay tu abbu ne inki ami ko chordya tha aur dusri shaadi karlee thee. Shaadi kay baad hum bilkul hee separate reh rahay thay.

**Interviewer:** Aur aap kay jo bachay hain woh aap kay husband kay saath rehtey hain?

**Interviewee:** Haan

**Interviewer:** Aap kay parents hayat nahi thay, tu kya unko aap ki beemari kay barey mein maloom tha?

**Interviewee:** Yeh beemari nahi thi, yeh eik saya tha. Aur eik khauf tha. Buss hur waqt khauf rehta tha tu mein tension mein rehtee thee, jiski waja say psychic problem hojatee hai aur phr woh patient ban kay mein agayein.

**Interviewer:** Jab aap ko saye kay barey mein pata challa aur aap ko saye ki waja say psychic illness hogaye hai tu aap ka kya radeamal tha? Kya feelings thi aap ki uswaqt?

**Interviewee:** Hmm uswaqt hum Garden mein rehtey thay tu kisi ne humein bataya kay wahan eik aurat illaj kartee hai. Phr wahan hum jaatey rahay. Boht tension aur khauf hota tha, aap Akeley hain lekin aap ko lag raha hai kay aap ko koi hur waqt dekh raha hai. Aap apne kaam mein busy hain aur aap ko mahsoos horaha hai kay aap kay barabar mein koi khara hai. Tu boht disturbance horahee the. Tu saya nazar aana, aur uski waja say tension paida hona aur phr psychiatric illness hojana.

**Interviewer:** acha aur aap apne ex husband ko batatee theen kay aap ko kya feel horaha hai?

**Interviewee:** Haan unko mein batatee thee

**Interviewer:** acha aur unki kya feelings hoteen theen?

**Interviewee:** Unhon ne mera ilaj karwaya tha aur woh yeh bhi kehtey thay kay buss saheeh hojayega

**Interviewer:** Acha aur aap kay bachay ubh tou barey hogaye hain tu unka iss pe kya radeamal tha?

**Interviewee:** Samajh jaatey thay kaafi acha samajh jaatey thay and khayal rakhta tha

**Interviewer:** aur aap ko kisi qasm ki madad miltee thee kay kisi ne aap ko beth kay samjhaya aur aap kay saath time spend kya?

**Interviewee:** Nahi buss roohani ilaaj waghera karwadiya.

**Interviewer:** Aap ko aap kay mekey walon ne support kya tha?

**Interviewee:** Bhai waghera ne thora boht kya.

**Interviewer:** acha aur aap kay jo ex husband thay woh aap ko support kartay thay?

**Interviewee:** Jee kartay thay.

**Interviewer:** aur ubh aap apne aap ko khud support kartee hain? Aap kay ex-husband koi aap ki madad kartay hain ubhi?

**Interviewee:** Nahi

**Interviewer:** Aap aur aap kay ex-husband ka milna julana hota tha? Aap logo ka?

**Interviewee:** Kam hota tha

**Interviewer:** aur aap ko problem hotee thee?

**Interviewee:** Jee

**Interviewer:** aur aap kay husband ko hotee thee?

**Interviewee:** Nahi unko nahi hotee thee

**Interviewer:** acha aur aap kay saye kay barey mein logo ko maloom tha?

**Interviewee:** Nahi hum ne kisi ko bataya nahi tha.

*inaudible*

Tension iss qism ki beemari ko aur bharatee hai. Aur humari beemari bhar bhee saktee hai, aur kam bhee ho sakhtee hai.

**Interviewer:** Acha aap ki elaidgi say pheley aap apne khandaani zindagi ko kaisay batayengee? Kaisee guzartee thee?

**Interviewee:** Parents kay saath boht achee guzartee thee. Waisay even shaadi shuda zindagi achee thee

**Interviewer:** Acha aur jab shaadi kay 7 sa al baad aap ko saye ka problem shuru hua tu aap ki shaadi shuda zindagi kis tarah tabdeel huwi?

**Interviewee:** Uskay baad tu sab upset hogaya tha. Life boht disturb hogayee. Husband ki huwi and bacho ki bhi disturb huwi. Agar eik insaan bhi beemar hojaye tu bhi ghar tou disturb hoga na. Family members dusre bhi disturb hongay. Eik hee ghar mein reh kar aur eik hee chat kay neechay rehtey hain, eik saath beth kay khatay hain tu disturb kaafi hogaya tha.

**Interviewer:** aur aap bata raheen theen kay saya aap ko kamo kay duraan bhi tung karta tha, aap ko lagta tha kay koi aap kay pass khara hai? Tu kya aap kaam kar patee theen? Cook waghera karliya?

**Interviewee:** Mein apne mind ko divert kartee thee tape recorder chalakay. Agar mein ghar mein akelee hotee thee tu mein kaam mein busy rakhtee thee lekin eik khauf hota tha.

**Interviewer:** acha tu aap ko psychiatrist kay pass jaaney ka kis ney bola tha?

**Interviewee:** nahi. Merey husband lekey gayee thay. Kyunke woh chahtay thay key mein theek hojaon. Agar mard beemar hojata hai tu woh tou office ki chutti karleta hai, lekin agar aurat beemar hogaye tu pura ghar disturb hota hai. Inhon ne ilaaj karwanay ki koshish karwaye

**Interviewer:** aur aap ki khandaani zindagi kistarah tabdeel huwi? Jab aap ko yeh problem shuru hua?

**Interviewee:** boht tension hogaye thee, bachay boht chotay thay. Kosish yeh hotee thee kay larai kam ho, kisi bhi mamlay mein. Mein aksar inkay kaam nahi kar patee thee.

**Interviewer:** Tu aap ka routine disturb hua tha?

**Interviewee:** Jee kaafi disturb hota tha aur mein apne aap ko change karnay kay liye meiney kaha na kay mein tape recorder istemaal kartee thee.

**Interviewer:** aap ki jo aap kay husband kay saath relationship thee woh kis tarah change huwi? Aap ko lagta hai koi tabdeeli agaye?

**Interviewee:** Saya jo hota haina aapis mein disturbance paida boht karta tha. Inkay disturb karne ka bhi koi disturb hota hai tu kaafi disturb hojatee hay. 5 saal hum ne istarah nikala.

**Interviewer:** agar aap batana pasand karein tu phr aap logo ne kyun decision liya kay aap elaidgi ikhtiar karlein?

**Interviewee:** Mera decision nahi tha, unka decision tha. Woh merey saath chal chal kay thak gaye thay. Mein yeh nahi keh rahee kay inki ghaltee thee. Koi bhi nahi karsakta manage. Woh aksar ghar kay sarey kaam kartay thay. Isliye

**Interviewer:** aur aap ki jo dusro kay saath relationship thi us pe koi farq para tha? Bhai hogaye ya bhen hogaye?

**Interviewee:** Pareeshani hoti thee tu kabhee unkay pass chalee gaye warna koi aisa nahi tha. Hospital mein thora boht dekh liya. Disturb tu sab huay thay meri waja say.

**Interviewer:** aur aap kay bachay ko ubh aap ki beemari kay barey mein maloom hai?

**Interviewee:** jee unko maloom hai

**Interviewer:** aur unko yeh pata hai kay elaidgi iss beemari ki waja say huwi thi?

**Interviewee:** jee barey betey ko pata hai. Chote bachay ko aap explain nahi karksate na.

**Interviewer:** aap ki problem ki waja say aap kay ex husband ko kabhi koi nafsiaati problem ya koi chirchirahat waghera huwi?

**Interviewee:** Unkay uper burden tu kaafi tha. Tension kaafi letey thay yeh bhi. Inhon ne bhi du teen sessions waghera attend kya thay.

**Interviewer:** aap kay aur aap kay ex husband ka saye ka problem say pheley din kaisa guzarta tha?

**Interviewee:** acha guzarta tha. Mein apne sarey kam kartee the aur phr jab yeh wapis aatey thay tu phr hum dunu jamaat khane jaatey thay.

**Interviewer:** aur aisa kabhi hua kay beemari kay baad inko aap ka kaam karna para?

**Interviewee:** Jee kaafi kaam kartay thay. Ghar kay kaam kartay thay.

**Interviewer:** agar aap ko bura na lagey tu mein aap say sawal karun kay elaidgi key pheechay kya wajoohat theen?

**Interviewee:** *pause* eik wajoohat tu nahi thee. Eik tou yeh kay hamaray parents ne apni marzi say shaadi ki hai. Arranged marriage thi. Financially husband ka strong na hona aur expense ko cover karnay mein kaafi pareeshani hotee thee. Boht zyada family kay liye struggling karna aur phr tension akey bachay waghera. Tu struggling marriage hojatee hai. Aur phr treatment waghera.

**Interviewer:** Aur financially problems hoteen theen?

**Interviewee:** Jee hoteen theen. Mujhe lagta tha kay theen

**Interviewer:** Kya woh aap kay medicine waghera kay kharcha uthathay thay?

**Interviewee:** Woh tu medical allowance miltee thee. Dawai waghera tu khareedletey thay

**Interviewer:** Tu yeh aap ko saya waghera kyun hua hai aur jo yeh tension hoti hai jiski waja say aap hospital mein admit hoti hain, woh kis wajja say? Aap ko lagta hai isski waja say koi waja hai?

**Interviewee:** Hosakta ho kay merey ex-husband second marriage karna chahtey hun tu socha hokay first wife ko itna disturb kardein. Kuch bhi karein, taakey araam say second marriage karsakein.

**Interviewer:** aap kay bachay kabhi aap ko kehtey hain kay aap ki galtee hai?

**Interviewee:** Nahi kabhi aisa kaha tu nahi hai. Bachay tou kaafi achay hain aur yeh hai kay bachay tu father kay pass hee rehtey hain. Bacho ki parhai saheeh waja say hosaktee hai.

**Interviewer:** acha. Aap ka jo choti bachi hai woh kitnay saal ki hai?

**Interviewee:** 9 saal ki

**Interviewer:** aap ko kiss qism ki mushkilat ka saamna karna parta tha?

**Interviewee:** *inaudible* isko lagta tha kay iss ki biwi fazul kharchi kartee thee.

**Interviewer:** acha aap ki beemari kay barey mein koi unsay sawal karta tha tu unka kya jawab hota tha?

**Interviewee:** Kuch bhi nahi, nahi batatey thay. Yeh beemari nahi hai, yeh saya hai

**Interviewer:** acha jab aap kay husband ne aap ko bataya kay elaidgi ikhtiar karna chahte hain tu aap ne foran say maan liya tha?

**Interviewee:** Nahi, accept nahi kya tha. Yeh tou direct Nikkah kar kay agaye thay aur mein accept nahi kar rahee.

**Interviewer:** aap logo ne elaidgi pheley ikhtiar karletee thee phr divorce de thee?

**Interviewee:** Jee

**Interviewer:** aap ko kya lagta hai kay kis waqt eik couple ko elaidgi ikhtiar karni chahye hai?

**Interviewee:** Jab understanding khatam hogaye ho. High level tak khatam hogaye ho. 3 months duration detey hain aur ussmein bhi na bansakey.

**Interviewer:** aap ko lagta hai kay aap kay husband ka decision ghalat tha?

**Interviewee:** Jee ghalat tha. Unko saheeh tareeqay say baat karni chahye thee.

**Interviewer:** aur aap marital counseling kay barey mein sunna hai?

**Interviewee:** Nahi

**Interviewer:** Aap ne try kya tha?

**Interviewee:** Nahi, lekin agar inkay parents hote ya merey parents hote tu ghar kay andar hee yeh baat hojatee. Inkay parents merey parents ko lekey bethtahy. And solve kartay aur inko samjhatey.

**Interviewer:** aapko lagta hai kay agar marital counseling hotee tu aap ki shaadi chal saktee?

**Interviewee:** kya aap ka kehne ka matlab hai kay jisne hamari counseling ki, uss ne saheeh nahi ki?

**Interviewer:** Nahi merey kehney ka yeh matlab nahi hai. Yeh alag qism ki counseling hoti

**Interviewee:** haan agar counseling hoti aur phr hum aapis mein beth kay decide kartay tu shayad saheeh hojata. Unhon nee hee nahi try karna. Jab unhon ne divorce ki baat ki tou meiney bacho kay barey mein phoocha tu unhon ne kaha kay mein dusri shaadi karunga.

***Interview Ends***
