# Supplementary material for: Exploring effects of severe mental illnesses on marriages: A qualitative study from Karachi, Pakistan
Source: PLOS Glob Public Health. 2025 Dec 23;5(12):e0005652. doi: 10.1371/journal.pgph.0005652 (PMC12725543; doi:10.1371/journal.pgph.0005652)
Supplement: S1 Data — (ZIP) [file pgph.0005652.s001.zip › Transcriptions/Case 2-6 Transcripts/Case 3/C3-1.docx]

**Case 3**

**27^th^ October, 2015**

**Psychiatric Illness: Bipolar Disorder**

**Outpatient Clinic: Dr. Murad Moosa**

**Interviewer:** Aapki umer?

**Interviewee:** 29

**Interviewer:** aap ne kitna parha hua hai?

**Interviewee:** mein graduate hun. Meiney BA kya hua hai

**Interviewer:** Kaam kar raheen hain?

**Interviewee:** Nahi bilkul housewife hun. Time hee nahi hai job karne ka.

**Interviewer:** Kitney bachay hain?

**Interviewee:** 2 hain, eik 5 saal ki beti hai aur 1 4 months ka beta hai mashAllah

**Interviewer:** Theek hai, aap nuclear family setting mein rehtay hain? Aap kay saas susr saath rehtey hain?

**Interviewee:** Jee bilkul saath rehtey hain. Joint family hai

**Interviewer:** Theek hai. Shaadi ko kitna arsa hua hai?

**Interviewee:** shaadi ko ubh November mein 7 saal hojainge

**Interviewer:** MashAllah. Theek hai. Aur acha, kitne log ghar mein rehtey hain?

**Interviewee:** 7 log

**Interviewer:** Kitne log kamatey hain?

**Interviewee:** Buss merey husband

**Interviewer:** aap kay husband ne kitna parha hua hai?

**Interviewee:** merey husband ne BCS kya hua hai

**Interviewer:** theek hai, aur aap ki family psychiatric history rahee hai?

**Interviewee:** Nahi bilkul bhi nahi hai

**Interviewer:** Aur inki family mein inkay illawa?

**Interviewee:** Actually unhon ne mujhe initially shaadi say pheley yeh bataya tha kay inko depression aur mein clearly samajh nahi paye thee. Aur mein tu yehi sochtee thee kay depression tu normally sab ko hee hota hai. Tu isliye clear nahi kya tha

**Interviewer:** Acha aur inkay illawa inki family mein kisi aur ko hai?

**Interviewee:** Nahi I don’t think so kay hai

**Interviewer:** Theek hai, aur yeh kitne arsay say beemar hain

**Interviewee:** Jee jo mujhe bataya gaya tha kay 1992 say inko problem hai. Kyunke yeh America mein thay uswaqt. Studies karne kay liye gaye huay thay. Iskay baad hua tha. 1990s say hee start hua tha

**Interviewer:** Aur aap ki shaadi kab hoye hai?

**Interviewee:** Meri shaadi hoye hai 2008 mein

**Interviewer:** 2008 mein aap ki shaadi huwi hai aur aap ko pata tha kay inko thori si koi problem hai

**Interviewee:** Haan depression ka kaha gaya tha

**Interviewer:** Lekin ub aap ko pata hai kay kya problem hai?

**Interviewee:** Ubhi bhi mujhe nahi pata kay kya problem hai *laughs* saheeh samajh nahi aata. Kabhi bilkul saheeh hote hain aur kabhi kabhar lagta hai kay haan waqaye koi problem hai

**Interviewer:** Hmm tu inko exactly hota kya hai?

**Interviewee:** waisay inko sirf yeh asal problem hai kay yeh absent minded boht zyada rehtey hain. Aisee koi baat horahee hai tu yeh foran say nahi samajh paatey hain. TV dekh rahay hain aur aisay beth jayengay kay sab bethay huay aur unko pata hee nahi hoga kay sab bethay huay hain. Buss isstarah kay masail hain.

**Interviewer:** Saheeh, kaam kartey hain?

**Interviewee:** jee job kartay hain.

**Interviewer:** acha aap ko income ka idea hai?

**Interviewee:** mujhe isska idea nahi hai

**Interviewer:** theek hai, tu kya shaadi kay baad yeh kabhi hospital mein admit hoye hain?

**Interviewee:** nahi shaadi kay baad tu kuch nahi pata. Shaadi say pheley agar hua ho tu mujhe saheeh say nahi bataya gaya hai

**Interviewer:** theek hai. Aur dawai letey hain?

**Interviewee:** jee jab say unko hua hai tu medicine tu letey hee hain.

**Interviewer:** saheeh. Theek hai aur psychiatrist kay pass hee atey hain?

**Interviewee:** jee shaadi kay kuch dinon baad aye thay aur kuch dino pheley aye thay. Medicine ka koi problem chal raha hai tha ..mil nahi rahee thee

**Interviewer:** acha smoking, drugs or alcohol ka isteemaal?

**Interviewee:** nahi, smoking kartay thay lekin mujhe lagta hai ubhi bhi chup chup key kartay hain

**Interviewer:** *laughs* aur koi financial issues?

**Interviewee:**  nahi financial issues tu nahi hain

**Interviewer:** aur dusre rishtey daaron kay saath masail?

**Interviewee:** jee meri hotee hain.

**Interviewer:** saas say?

**Interviewee:** saas say nahi, nand say hotee hain *laughs*

**Interviewer:** acha aur aap ki koi marriage mein problem waghera?

**Interviewee:** nahi waisay tu humari married life theek hai. Lekin agar koi aur involved hojata hai tu husband aur wife ki relationship mein thora tu farq parta hee hai tu woh hai

**Interviewer:** aap ko pata tha kay inko psychiatric problem hai lekin exactly kya thi yeh nahi pata tha?

**Interviewee:** Jee mujhe exactly nahi pata tha

**Interviewer:** acha inhon ne bataya tha ya inki family ne bataya tha?

**Interviewee:** actually family hee nay bataya tha..meri nand ne bataya tha kyunke yeh rishta lekey ayein thee. Inhon ney hee bataya tha kay inko depression ka problem hai aur mujhe nahi pata tha kay depression kiss had tak hai. Mujhe buss yeh laga tha kay jistarah mein Pakistan mein rehti hun istarah sub ko hi depression hota hai

**Interviewer:** Tu aap ko kya lagta hai kay inka depression kis tarah say alag hai? Aur jistarah aap ne socha tha aur jo actually hai?

**Interviewee:** Dekhain shaadi kay shuru mein mujhe lagta tha kay inko koi problem hai kyunke yeh kaafi ghussa kartey thay. Shuru mein mujhe yaad hai kay mein NIC banwane gaye thi aur kaafi lag raha tha aur yeh bahar wait kar rahay thay aur andar aana allow nahi kya tha, sirf eik person allowed tha jiska NIC ban raha tha. Thoray late hogaye tu yeh darwaza khol kay agaye aur ghussay mein sunaye tu usswaqt yeh boht ghussa kartay thay, shaadi kay shuru mein, Jab tak kay hamari beti nahi huwi. Jab tak mein na inko samajh pa rahi thee aur na yeh mujhe understand kar rahay thay. Lekin jaise hee beti huwi sab theek hogaya hai. Aur response late kartay hain ubhi tak.

**Interviewer:** Tu aap ko kya mahsoos hua tha jab aap ne inki beemari kay barey mein pheli dafa sunna tha?

**Interviewee:** Wohi baat hai kay mujhe clear nahi hua tha aur ubhi bhi mujhe nahi samajh aata lekin mujhe lagta hai kay yeh 90 percent theek hain. Kyunke meiney kaha kay shuru mein ghussa kartay thay tu mujhe buss eik depression sa hogaya tha, tu mein depression ko lightly leti thee, lekin ubh sab settled hai, bachay bhi hain, job bhi hai, zindagi achee guzar rahee hai

**Interviewer:** acha aur aap kay parents ko bhi pata tha

**Interviewee:** jee merey parents ko bhi pata tha

**Interviewer:** aur unko koi masla nahi tha?

**Interviewee:** actually itna clear bataya nahi tha jitna clearly mujhe baad mein pata chalta gaya kay itna severe hogaya tha, aur ami ne mujhe bataya tha, inhon ne tu phr bhi mujhe nahi bataya tha

**Interviewer:** kya severity thee?

**Interviewee:** matlab kay itnay hogaye thay kay America say sub kuch chor kay aana para tha, apni studies aur apni job, sab kuch chor kay ami inko wapis le aye theen aur phr yeh Saudi Arab mein thay aur kabhi Karachi mein thay, aur inki tabiat bilkul theek nahi thee, ghum sum bethay rehtey thay, yeh ammi ne mujhe bataya tha

**Interviewer:** acha aap logo ka rishteradon aur dosto mein aana jaana hota hai?

**Interviewee:** Bilkul hota hai

**Interviewer:** aur kya log inki beemari kay barey mein phoochtay hain?

**Interviewee:** nahi woh mujh say kuch nahi phoochtay because sab ko pata hai, family mein sab ko pata hai, tu koi sawal mujh say nahi kartay

**Interviewer:** aur inkay dost waghera?

**Interviewee:** dosto say inka nahi raha. Kyunke family mein hai buss

**Interviewer:** aur aap ki dostein waghera?

**Interviewee:** frankly bataon tu shaadi kay baad meri koi dost nahi rahee kyunke time hee nahi hai aur phr mein Saudi Arabia chalee gaye thee

**Interviewer:** Tu aap log recently shift hoye hain?

**Interviewee:** haan ubhi 4-5 years hoye hain

**Interviewer:** Acha aap ko lagta hai kay aap ki family dusri families say different hai?

**Interviewee:** Bilkul hai

**Interviewer:** acha kis tarah lagta hai?

**Interviewee:** matlab meri mother ki family ka keh rahee hain?

**Interviewer:** nahi matlab generally, aksar aap ne suna hoga kay nafsiati beemari hai tu problem hoksatee hai.

**Interviewee:** nahi iss type ka kuch bhi nahi hai

**Interviewer:** aap ka din kaisa guzarta hai?

**Interviewee:** yeh subah office chale jaatey hain aur mein bacho kay saath hoti hun aur boht acha guzar jaata hai aur phr sham mein inka wait kartee hun kyunke yeh ayengee aur chai banaye jaati hai aur khaana waghera dena hota hai. Istarah guzar jaata hai

**Interviewer:** Acha aur aap ki beti ne kabhi aisa notice kya kay abbu ki tabiat upset hai?

**Interviewee:** nahi kabhi aisa notice nahi kya. Kyunke mein nahi samajhtee kay ubh inko severe hai. Phele hua hoga tu theek hai. Settle hogaya hai

**Interviewer:** acha aur aap ko lagta hai kay aap ne zyada zeemadarian le huwi hain jab yeh zyada ghum sum waghera hojatay hain aur aap ko jawab nahi detey?

**Interviewee:** nahi agar inko koi flu waghera hojata hai tu mein inki boht take care kartee hun kyunke inkay upper saari zeemdarian hotee hain

**Interviewer:** and jo yeh dawaiyan letey hain, khud leletey hain?

**Interviewee:** haan khud leletey hain aur time pe bhi leletey hain. Saalon say le rahay hain lekin phr bhi sonay say pheley mein jag rahee hoti hun aur yeh kamray mein aatey hain tu mein phoochtee hun kay aap ne dawai lelei? Aur haan

**Interviewer:** aur aap ko lagta hai..kuch logo ko farq parta hai na kay depression hai aur shaadi karlee hai, aap ko kabhi aisa feel hua hai?

**Interviewee:** actually shuru mein meiney itna seriously nahi lya tha. Kyunke depression kay barey mein mujhe detail nahi pata thee. Inko ghussa aajata tha aur mujhe bhi ghussa ajata tha aur mein upset hojatee thee kay shaadi kay shuru mein aisay problems horahay hain. Phr boht problems bhar gaye aur phr pregnancy hogaye tu aur zyada bhar gaye. Merey husband say nahi bharay thay jitna kay family kay dusre logo ne create kardiye thay. Itnay issues create kya kay mujhe laga kay buss ubh agay nahi chal payega. Humari shaadi shayad khatam hosaktee hai aur phr meri bhen ne boht zyada samjhaya kay nahi bacha honay wala hai aur meri ami ne bhi samjhaya aur meri saas bhi nahi chah rahee theen kay rishta khatam ho. Woh nahi chahtee theen kay inkay bachay kay saath koi aur dusra mishap ho, tu inki bhi koshish thi aur phr merey ghar walon ke bhi koshish thee tu phr shaadi chal gaye. Aur phr boht achee rahee hai aur achee guzar rahee hai.

**Interviewer:** Theek hai, aur aap ko lagta hai kay aap ki shaadi expectations kay mutabiq hai?

**Interviewee:** *laughs* waisay mujhe lagta hai kay koi bhi completely satisfy nahi hota lekin mujhe lagta hai kay ubh 90 percent behtar hai

**Interviewer:** lekin jo aap ne depression ka jo socha tha aur jo aap ne dekha hai ussmein expectation mein farq tha?

**Interviewee:** jee shuru mein tha lekin ubh nahi hai kyunke shayad meiney khud hee inko itna change kardiya hai. Tu woh saheeh hogaya hain

**Interviewer:** Acha tu aisee konsee cheezain theen jo aap ki expectations say bhar kay theen kay nahi meiney nahi socha tha kay yeh bhi hosakta hai?

**Interviewee:** Aisa meiney kuch socha nahi tha buss shaadi kay baad mujhe proper ghar nahi diya gaya tha. Fmaily wahan rehtee thee aur mujhe yahan shift kardiya tha. Aur shaadi kay baad yeh chalay gaye thay tu mein ami kay yahan wapis agaye thee tu mujhe yeh cheezain nagawar guzartee theen kay yaar ghar hee nahi hai, kaisi shaadi hai

**Interviewer:** Inkay saath aap ki koi problems..

**Interviewee:** inkay saath koi problem nahi rahee, aur agar family mein mujhe koi kuch kehta tha tu inpe saara ghussa nikal jaata tha aur yeh samajhtey bhi hain. Ghussa nahi kartey ubh

**Interviewer:** theek hai, aur aap ko kya lagta hai kay eik shaadi shuda joray ko elaidghi kay barey mein sochna chahye hai?

**Interviewee:** wohi jab dunu eik dusre ko understand nahi kar pa rahay hote hain. Nahi samajh rahay hotein lekin shukur hai kay aap understanding hogaye hai, bachay hogaye hain tu ubh tu kuch nahi sochtee iss barey mein

**Interviewer:** pheley aap ne socha tha?

**Interviewee:** shuru mein socha tha haan. Even kay meri 3 months ki pregnancy hogaye thee tab bhi meiney soch liya tha. Kay shayad ubh nahi chal sakta aur woh bhi husband ki waja say nahi, family ki waja say.

**Interviewer:** lekin aap ko kya lagta hai kay inki beemari ki waja say aap ki married life mein koi farq para hai?

**Interviewee:** nahi as such koi farq nahi para, nahi mujhe nahi lagta. Haan yeh hain kay istarah ki activities nahi hain kay hoteling waghera horahee hai, shopping horahee hai, aur bachay bhi hogaye hain ubh aur ubh mujh mein bhi itna nahi hai kay mein kuch karun

**Interviewer:** tu shuru mein aap expect kartee theen?

**Interviewee:** jee shuru mein kartee thee jab tak meiney conceive nahi kya tha, aur mujhe hota tha kay mein ghoomo phiron lekin ubh nahi hai

**Interviewer:** aap ko lagta tha kay yeh inki beemari ki waja say hua tha?

**Interviewee:** jee eik tarah say beemari ki waja say hee keh lein kyunke ami darteen theen kay yeh Akeley nah bheja jaye matlab mein bhi larki hun thora ghabratee theen, shuru mein boht zyada hota tha

**Interviewer:** hmm jee samajh gaye, acha aap ko kya lagta hai kay eik pur sukoon khandaan ko parwarish karne kay liye kis cheez ki zaroorat hoti hai?

**Interviewee:** Buss mohabat chahye hai

**Interviewer:** acha aur aap ko lagta hai kay aap ki shaadi shuda zindagi mein yeh hai?

**Interviewee:** jee bilkul hai.

**Interview Ends**
