# Supplementary material for: Exploring effects of severe mental illnesses on marriages: A qualitative study from Karachi, Pakistan
Source: PLOS Glob Public Health. 2025 Dec 23;5(12):e0005652. doi: 10.1371/journal.pgph.0005652 (PMC12725543; doi:10.1371/journal.pgph.0005652)
Supplement: S1 Data — (ZIP) [file pgph.0005652.s001.zip › Transcriptions/Case 2-6 Transcripts/Case 3/C3-2.docx]

**CASE 3**

**Married since 4 years**

**Red =Interviewer**

**Black = Interviewee**

**Inki bimaari kab se hai inko?**

**Chaar saal**

**Taqreeban in chaar saalon mein kitni dafa instable hue hain?**

**Shaadi ke baad thora sa ye tha keh hojatay thay. Matlab ye ke ek do dafa toh hue thay. Chaar saal pehle ki baat hai. jab meri shaadi hui thi nayi nayi toh mein inke mizaaj ko nahin samajh paa rahi thi.**

**Aur shaadi ke doraan aisa kabhi hota hai keh ekdum se kuch hojata hai?**

**Nahin. Koi aisi baat nahin. Bas ye hai keh ye teen behnon ke baad hue thay toh bahot zada naazon mein palay hain. Toh itna zada tha keh choti si baat ko bahot jaldi feel ker jaate hain keh bas koi batameezi nahin ho. Koi mazaq na keray**

**Takqreeban inko kitna arsa hua hai treatment lete hue ya dawaiyan lete hue?**

**Aath-das saal ke pehle ki jab tak baat hai. Jab ye urooj ko pasand kerte thay tab inhon ne suicide kerne ki koshish keri thi toh uswaqt mere susar jo hain wo hanif mesiya ke paas leke gaye thay jab wo chotay clinic mein thay toh unhon ne uswaqt bola tha keh ek tablet hai jo suicide ke liye deni pereigi par wo unhon ne 15 se 20 din khayi thi phir unhon ne chor di thi. Usko baad jo hai inko chaar saal pehle ye wala hua tha uske liye 15 se 20 din hospital mein rakha tha**

**Toh abhi pichlay chaar saal se dawaiyan le rahe hain?**

**Lete hain..**

**Theek hai. Aur aap ne aur kisi se madad li hai is hawalay se ya sirf dr. hanif mesiya se?**

**Sirf Dr. Hanif Mesiya se**

**Theek hai. Sahi hai aur ye koi kisi kism ka nasha ya sharab peeti hain?**

**Kuch bhi nahin. Kuch bhi nahin**

**Iske elawa aap ko kisi aur kism ke problem, koi maali mushkilat ya kisi aur health ka apka apna koi problem ho? Aap ko lagta hai ke aap ko kisi kism ki help ki zarurat hai, kisi pareshani ke hawale se?**

**Nahin, koi problem nahin.**

**Aur Aapki shaadi shuda zindagi mein koi masla?**

**Koi masla nahin hai Allah ka Shukar, bas baby ka thora sa hai ke hojaye. Baqi Allah phir chordia hai. Treatment kerwaya tha but koi issue nahin hai, doctor kehte hain Allah ki taraf se hai.**

**Chalein ab mein aap se kuch sawaalat kerna chahungi, agar aap ko lagta hai koi aap ko sawaal ka jawab nahin dena toh aap beshak nahin diye ga**

**Acha. Jab aap ko bimaari ke baaray mein maloom hua, jaise aap engagement ke doraan baattein kerti theen unse, toh aap karad-e-amal tha yaani aap ka reaction kya tha?**

**Mujhe toh ye tha ke bhalay kitni bhi bari bimari kyun na ho saath toh dena hai inka, mein paghal thi uswaqt bahot zada out hogayi thi. Par mujhe koi pachtawa nahin hai keh mein ne ghalat kiya, mein shuru se thi keh bhalay koi bhi problem ho. Halaan keh meri family chahti thi ke engagement khatam kerdun, to mein ne kaha agar engagement khatam kari toh phir mein kisi se nahin kerungi. Umeed nahin rakhna mujh se.**

**Aap ko mushkilat ka andaza tha keh ke aap ko is tarah ki mushkil hogi?**

**Mujhe pata tha keh mujhe mushkil hogi, Allah ka shukar hai hogaya sab ke saath. Actually jo mere susraal wale thay Allah ka shukar hai keh mujhe susraal wale bhi achay mile, cooperative thay keh unhon ne bhi mera ye tha ke saath diya, toh koi itna mushkil nahin tha**

**Toh aap ki family ne ye baat ko accept nahin kiya tha?**

**Uswaqt nahin kiya tha phir ahista ahista..**

**Aur aap ko unki taraf se support milta hai?**

**Haan sursral ki taraf se milta hai haalan ke mein ne toh apne family ko ye bhi nahin bataya ke chaar saal se inki medicines chal rahi hai mein ne toh apne ghar walon ko relax kerne ke liye bola ke inki medicines, koi dawai kuch bhi nahin hai aur ab jab unki condition kharab hui hai toh char saal ki jo sachayi hai wo ab samne aayi hai ghar walon ke samne nahin toh kuch bhi nahin pata tha mere maikay walon ko.**

**Ab unko lekin maloom hai, toh ab unka kya rad-e-amal tha?**

**Unka abhi rad-e-amal nahin hai, unko hairat bhi hoti hai aur phir khushi bhi hoti hai keh chalo jaisa bhi hai ye chala rahi hai sab kuch ker rahi hai ab ye Allah ka shukar hai keh wo log to cooperative bahot zada hogaye hain, ab aisi koi baat nahin hai**

**Acha,aap ne jab shadi kerli uske baad kabhi aap ne socha ke nahin aap ko nahin kerni chahiye thi kabhi pachtawa kisi kism ka?**

**Kabhi bhi nahin. Allah ka shukar kabhi pachtawa nahin hua, kabhi nahin hua**

**Wo aap ke saath sahi/theek rehte hain?**

**Allah ka shukar, ye mere saath bahot achay hain. Bahot achay hain. Nature wise. Aur mujhe itni khushi hoti hai keh bhalay choti si bimari ho koi masla nahin, who ek taraf bas ye tha keh ye har waqt mera saath zarur dete hain. Mushkil waqt acha waqt bura waqt. Taqleef mein hoti hun yahan tak ke zera sa khoon bhi nikle toh ye pereishaan hojate hain. Bahut zada care kerte hain toh mujhe kabhi afsos nahin hua keh mein ne ghalat faysla kiya allah ka shukar hai ye bhi bahot khayal kerte hain koi aisi baat nahin hai.**

**Acha. Aap logon ka risthedaron mein aana jaana milna milaana hota rehta hai?**

**Hota rehta hai**

**Aur rishtedaron ko maloom hai?**

**Kisi ko bhi nahin maloom, kisi ko bhi nahin maaloom**

**Kabhi unhon ne koi is tarah ki harkat ki ho jis se rishtedaron ne kabhi poocha ho keh inko koi problem hai, kabhi aisa hua hai in chaar saalon mein?**

**Nahin aisa toh koi problem nahin hua. Kyunke kisi ke samne koi aisi baat ayi nahin thi. Hum ne aanay bhi nahin di. Ye tha ke hamari jab joint family thi toh hamare chacha wagera thay dada dadi thay toh unko ye baat pata thi unhon ne toh khair apne thay mere samne toh koi aisi baat nahin keri per jab ye bimar hue toh chacha log mazaq zarur uraate thay**

**Jo aap logon ke saath rehte hain?**

**Haan jo saath rehte thay wo peechay peechay ye tha keh bolte rehte thay samne se bahot achay thay per peechay se ye tha keh bolte rehte thay hanste thay haansi mazaq kerte thay**

**Acha. kabhi kisi ne aapke doston ne ya kisi aur maikay walon ne, ami abu bhai behan apke cousins hogaye maikay wale side se unhon ne kabhi poocha iss bimaari ke baaray mein?**

**Haan unhon ne poocha tha toh mein ne kaha keh koi aisi baat nahin bilkul theek hain. Balke mein toh inke baaray mein jab batati thi toh sab kehte thay bahot achi baat hai sumbul bahot acha ghar mila hai sumbul bahot acha shauhar mila hai sab khush hote thay yahan tak keh abhi tak meray jo mekay walay hain jo meray khala wagheira unki betiyan toh who yehi kehte hain ke sumbul ki life toh bahot achi hai. Allah ka shukar hai mere inlaws wale thoray paisay wale thay toh allah ka shukar hai mujhe kisi cheez ki taqleef nahin thi koi problem nahin thi aur mein hamesha in logon ko mein achi dikhti thi sab ke aagay halaan ke mere saray behnon ki shadiyan hain lekin un ke aagay mein sab ko achi lagti hunk eh sumbul sab se achi hai susraal mein sab se zada khush hai toh koi aisi baat hoti nahin thi unlogon ko mauqa hee nahin mila hai baat kerne ka mauqa hee nahin diya hai koi aisi baat kerne ka**

**Acha aap ko lagta hai keh aap ki jo family hai, aap ki jo shadi shuda zindagi hai aur aap ki jo family hai wo kisi tara dusri family se different hai, mukhtalif hai kisi tareeqe se?**

**Bas ye hai keh susraal wala mera maamla nahin hota. Jaise ke ek hota hai na keh susraal mein ek nand hai saas hai susar hai. meri family mein koi aisa problem nahin hai, sab mil ke rehtay hain beti banaya hua hai unhon ne bahot zada kerte hain ye log mera koi perieshani ki bat nahin hoti. Bas haan mujhe ye hai keh maikay ki kami nahin hoti itni zada allah ka shukar hai bahot acha hai bas ye hai k eek choti si taqleef hai bas aaulad nahin hai baqi sab kuch acha hai bas ye problem hai aur kuch bhi nahin hai. abhi jab meri tabiyat kharab hui hai toh ek do logon ne kaha hai keh shayad aulad na honay ki wajah se kharab ho. Tum kuch kero tum kuch kero kisi aur tareeqe se baby conceive jo kerte hain is tareeqe se kero kuch kero mein kehti hoon ye sab kuch Allah pe chordo. Par log kehte hain ke shayad iski tabiyat, kyun ke chaar saal hogaye hain. Kyunke hamara umrah pe jaanay ka jo iraada tha ham ne yahi socha tha ham ne doctor ko dikhaya tha doctor ne kaha tha koi aisi baat nahin hai toh inhon ne bola tha ke jab ham umrah pe jayenge toh inshallah wahan dua mangein ge to humein aulad hojayegi . inhon ne wahan jaakay itni shidaat se ibadaat ki hai shayad mein ne bhi itni nahin ki hogi jitni inhon ne ibaadat ki hai. toh inko pura yaqeen hai abhi bhi yahi yaqeen hai keh aap ne test kerwaya hai dekho positive toh nahin aya hai abhi bhi hospital mein honay ke bawajood ye baar baar ye poochtay hain ke doctor ko check up kerwaya. Mein kahan se check up karwaaon aap aa toh jao phir checkup bhi kerwa lungi bas yehi tha aur koi wajah nahin thi. Aur ye baar baar is tarah ki baat kerte hain toh Sab ko ye lagta hai ke shayad aulaad ki wajah se iski tabiyat kharab hai.**

**Acha. mein aap ke kuch din ke baaray mein kuch maloomat kerna chahungi. Keh aap ka poora din kaise guzarta hai, subah se raat tak jo aap ka routine hota hai woh kaisa hota hai?**

**Mein subah uth jaati hun saaray aath bajay**. **Saray aath bajay inlogon ka** **Ye log dukaan ye log das bajay dukaan jatay hain toh tiffin waghera tayar kerna hota hai khana banana hota dupahar ka khana waghera nashta waghera diya phir maasi aagyi maasi se kaam waghera kera ke saaray gyaara bajay farigh uske baad mein ne kapre istri kerliye chawal chunliye thora bahot apna kuch kaam kerliya phir namaz perhi roti banayi khana khaya 3 bajay tak hai keh tv waghera dekh ke sogayi phir sham ko saaray paanch che bajay uth ke asr ki namaz per ke uske baad phir raat ke khaane ki tayari phir ye log ajate hain raat ko khaana khaya wohi inke saath chaat pe chalay gay ek dair ghanta walk kerli**

**Aur aap apne miyan ka is dauran pooray ek din mein kis tara khayal rakhti hain matlab aap ko medicine wedincine deni perti hai?**

**Bas ye tha ke subah nashta jab ye kerte thay toh nashta keh saath inko dawai deni hoti hai aur raat ko sotay hue bas dawai deni hoti hai.**

**Dupair ki koi medicine nahin?**

**Dupair ko koi medicine nahin**

**Aur wo aqsar mana kerte hain medicine lene se? yaani tang wang kabhi..**

**Shadi se chaar saal hogaye hain ek baar bhi dawai ka mana nahin kiya. Han kabhi ye hota hai keh mein dawai dena bhool jati hoon toh khud le le te thay. Dukaan walon mein se mein dena bhool jati hoon toh dukaan se waheen se khareed ke le lete thay phone kerdeti thi aap ne dawai nahi li toh le lete the dawai. Raat ko bhi mein agar mein dena bhul gayi toh aadhi raat ko ankh khulti thi toh le lete thay dawai mujhe dawaiyon ke koi problem nahin hua. Haan ye hai keh umrah pe jaane ke baad wahan jakay kehte they mein ne allah taalah se dua mangi hai keh mujhe dawaiyon ki zarurat nahin hai mein ne allah taalah se maangi hai toh mujhe dawaiyan ki zarurat nahin hai mein dawaiyan dustbin mein daal deta hun mein ne kaha dawaiyan dustbin mein nahin dalta ham ghar jaakay dekhein ge. Toh inhon ne ye tha keh ek do din ye tha keh dawai wahan pe nahin khai. Baqi toh dawaiyan toh already ye chaar saal se le rahay hain.**

**Aur aap ki shaadi ki dauraan hospitalization kitni dafa hui hai inki?**

**Ek hee dafa hui hai**

**Abhi jo hai ya usse pehle bhi ek dafa hui hai?**

**Meri engagement ke time, meri shaadi se do maheenay pehle hi das din ke liye admit hue thay**

**Toh shaadi ke dauraan pehli dafa hui hai?**

**Kuch bhi nahin. Jeee ye pehli dafa hui hai**

**Toh aapki zimidariyan bhaar gayi hain kya?**

**Thori bahot toh barh gayi hain. Abhi inki tabiyat aisi hai ab pata nahin aagaya jakay inko kis tareeqe se sambhalna paray kuch idea nahin hai kyun abhi mein umre pe gayi thi toh mujhe bahot problem hui thi kyunki inki baatein ye bilkul out hogaye thay bilkul out hogaye thay toh inka haath pakar ke rakhna perta tha har waqt. Yahan tak ke jab jahaz mein ham loh baithne walay thay toh ham airport paunche toh airport peh kehte hain hain pia mein se smell se aarahi hai pia mein dhamaka hojaye ga ham Saudi airline se jayenge. Mein ne kaha dekho pia ab aagayi hai hamein ghar jaana hai abu hamara airport pe intezar ker rahe hain. Nahin mere ko ghar nahin jaana mujhe ghar walon se nafrat hai apun Mumbai chalte hain. Mein ne kaha ham log Mumbai kaise jayeinge abhi ham Pakistan jaarahe hain wahan se Mumbai chalte hain ghar walon se milke phir apun chalte hain. Kehte hain mein pia mein nahin jaunga Karachi mein Saudi airline mein jaoonga mein ne kaha acha chalo theek hai mein ne inko bithadiya mein ne kaha theek hai jaise merzi. Phir jab sab chalay gaye jahaz mein tab jakay baith gaye mein ne kaha dekho mein ne inko ghuma diya inko kaheen aur le gaye phir wapis le gaye mein ne kaha dekho Saudi airline agayi apun chalte hain. Jab ham ander jaanay lagay jaisi jahaz ke darwazay pe jaisi enter hone lage kehte hain mujhe jhoot bol ke layi ho na pia mein samaan waheen phaika aur wapis bhag rahay thay keh mein jaaraha hun mein nahin jaoonga mein nahin jaaongai mein yaheen rahunga mujhe Karachi nahin jaana. Toh itni mushkilon se pakar ke mein ne inko jahaz mein bithaya hai. jahaz mein bhi keh rahe thay mere saath jhoot bol ke layi ho acha nahin kiya tum ne. mein ne kaha mein kya kerun ghar walay airport pe intezar ker rahe hain ghar jaakay phir apun log Mumbai chalay jayenge. Mein ne chay saath botlein paani ki lee theen, wo saari khatam hogayeen. Itna paani pee rahay itna paani pee rahay itna paani pee rahay ke samajh hee nahin aarahi. Paani pe rahay aur washroom jaa rahay hain aur baar baar washroom jarahay hain ke wahan jo air hostess wo pareishan hogayi aur mein bahot zada tension mein aagayi keh mere maide mein bahot taqleef horahi thi mein aisi taik laga ke baithi hui thi aur kab aankh lagi mujhe pata nahin ab wahan ki airhostess ya pata nahi kis ne mere barabar walay jo baithe hue thay unko shikayat keri keh bahot perishan ker raha hai isko bithao . mere ko uthaya unhon ne ke aap ka husband bahot pereishan ker raha hai ye log complain ker rahay hain keh apne husband ko bithao ye kya baar baar uth ke bathroom jaraha hai. mujhe toh samajh mein hee nahin ayi mein pareishaan phir mein ne inko kaafi dair bithaya kehte hain mere ko zaberdasti layi ho mein chorun ga nahin. Puri raat uswaqt bhi jagay hue thay pareishan puray jahaz mein chakar laga rahay thay bahot zada bilkul samajh mein nahin araha tha ke inko hua kya hai log bhi kaafi pareishan hogaye kabhi ro rahay thay kabhi tension mein kabhi chakar laga rahay thay. Kyun ro rahay thay ye nahin samajh araha tha**

**Jab jahaaz land hua tou inko chakar arahay thay, phr meiney saara saaman liya tou uswaqt bhi yeh boht ghussay mein thay kay buss mein chala jaon. Phr jab unko pata challa kay abbu ko inki beemari kay barey mein maloom hai tou yeh dar gaye, merey maikay walo ne abbu ko batadiya tha, and bhai ko bola kay isski tabiat boht kharaab hai tou issko kisi tarah bhi ghar le jao. And ajeeb ajeeb see baatein kar rahay thay, kay aaj Karachi mein boht bara dhamaka hoga.**

**Tou aap kaisa mahsoos karteen hain jab inki aisee kaifiat hotee hai?**

**Tension boht hotee hai, kyunke inkay saath yeh problem tou hai buss Allah tallah mujhe himat dey key mein inko sambhal lun. Merey jo susr hain na insay itnay zyada attach hain kay yeh boht rotay hain inki tabiat dekhtee hain aur iski wajah say mein boht pareshan hotee hun kay mein inko sambhalon ya unko dekhoon aur phr sab mujhse sawal karte hain kay doctor ne kya kaha, ubh koi baat batao tou tension letey hain aur nahi batao tou pareeshan hote hain. Abbu yehi phoochtay rehtay hain kay aaj hospital mein kya hua tou mein ney kaha kay aaj bilkul relaxed thay tou yeh baar baar phoochtay hain kay koi tou baat huwi hogee. Aur mein kehteen hun kay aap tension kyun dhoond rahay hain, koi aisee baat nahi hai. Allah meri sunta nahi hai, mein marnay wala hun, merey susr bhi aisee baatein kartay hain, ghar boht zyada disturb hogaya hai. Susr chah rahay hain kay doctor innko discharge kardein, aur doctor kehtey hain kay mein discharge nahi karunga jab tak yeh properly theek nahi hojate. Abbu nee aaj bhi mujhe bheja hai kay inko discharge karke lao lekin meiney inko kaha kay aaj tou nahi, kal koshish karte hain, agar tabiat behtar hungee. Ubh abbu ko kon samjhaye lekin abbu boht zyada inteezar kar rahay hain kay bus yeh ajayein. Aur abbu properly khaana bhi nahi kah rahay tou ghar pura boht disturb hogaya hai. Ghar ke barey baite hain tou yeh samjhtey hain kay yeh kamzoor hogaye hain kay bsus ghar aajeye tou theek hojaeinge. Aur himaat ajayegee.**

**Tension boht hai kay achanak tabiat agar kharab hojayegee tou. Yeh keh rahay thay kay aisa umray pe kya hua jou tabiat achanak kharab hogaye. Yehi phoochtay hain kay koi larai waghera hoye thee tou meiney kaha kay nahi koi larai nahi howi. Hamari waisay eik du din mein koi na koi larai hotee rethtee hai, lekin wahan tou hamari koi larai nahi huwi.**

**Aap ki chotee mote laraiyan hotee theen ya bari laraiyan hotee theen?**

**Chotee mote hotee theen jaisay kay meiney yeh bola kay aap itna zor say kyun cheekh rahay hain tou yeh mujhe kehtey hain kay aap ne cheekhna kyun bola, aap ne mujh say batameezi ki hai. Aap ne zor say kyun bola, and yeh choti choti baatein boht zyada note kartay hain kay aisa kyun bola, waisa kyun bola, batameezi kyun kareen aur kamray say nikal jaatey hain.**

**Acha tou aap kay uper yeh cheektey hain?**

**Cheektay nahi hain woh mujhe is tareeqay say kyun bola, mein jar aha hun**

**Nahi mera matlab hai kay aap ko lagta hai aisa kay yeh aap kay uper cheekh rahay hain?**

**Haan**

**Boht ghussay mein aajatey hain isliye?**

**Haan jee. Yeh ghar mein sab kay saath aisee hee hain, barey hain na tou boht laad pyaar mila hai, order boht detey hain. Mein kabhi manna kardeti hun tou mood kharab hojata hai.**

**Kabhi ghussay mein bataeemzi, kabhi haath wgahera uthaya ho?**

**Haath kabhi nahi uthaya.**

**Acha waisay aap ki arranged marriage thee ya pasand kee thee?**

**Arranged marriage thee**

**Acha tou aap kisi bhi cheez ka manna kartee hain tou yeh ghussay mein aajatey hain?**

**Interviewee:** Haan agar mein jawab saamney dun tou ghussay hote hain lekin agar mein khamoosh hojaon tou khud hee samajh jaatey hain aur thori dair baad aatey hain, lekin agar mein jawab dedun jaisay agar mein mehndi laga rahi hun tou yeh kehteey hain kay shauhar ki baat nahi manogee na tou meine kehteen hun nahi maani mujhe shauhar kee baat, mein thak gaye hun, pheley tou ghussay mein ayengee lekin phr kaheenge kay chalo theek hai jaisay aap ki marzi. Inka ghussa bhi aisa hai, lekin thori dair baad thanda hojata hai aur phr maafiyan maangtey hain, puri family key beech mein maafiyan maangtey hain kaan pakar kar *laughs* itna bhee hota hai.

**Interviewer:** Ubh inki mein beemari kay bareey mein aap ki maloomat hasil karna chahoongee. Aap ki aisee kya zaati wajoohat theen kay aap ne socha kay chalo mein shaadi karlunge, kyunke aap ko pata hai nafisiati marz mein problems hotee hun, acceptable nahi hota

**Interviewee:** Nahi meri boht choti umer thee tou mujhe idea nahi tha kay mujhe aagay jaakey itna face bhi karna pareyga. Mushkil hogee, aur phr baat cheet shuru hogaye thee tou interest bhi boht zyada bhar gaya tha, naya naya pyaar ka khumaar tha tou karlengee, baaki Allah malik hai, dekha jayega, Koi aisee baat nahi thee, zehen mein aisa kuch nahi tha, mein samajh rahee thee kay sab acha hoga. Kay hum jo phone pe baat kartay thay, barey barey khuawab dikhaye thay woh sab sach honge. Shaadi kay baad yeh sab aisa nahi tha, inko samjhana boht mushkil tha aur sambalna bhi kaafi mushkil tha, neend ki kami aur time pe nahi sotey thay tou bhi problem hotee thee aur kabhi kabhar dukaan pe bhee nahi jaatey thay, merey ko neend arahee hai aur subah uthnay mein boht masla kartay thay kyunke dawaiyon ka asar hota tha. Raato ko late late jaagtey thay. Abbu bhi pareshaan hogaye thay. Inhon ne bilkul karoobar ko chor diya tha, bhai sambhal raha tha lekin 6-7 meheny baat theek hogaye

**Interviewer:** acha lekin aap apne barey mein zyada bataye kay aap ki expectations kay hisaab say tha ya expectations say bhar kay tha, boht zyada mushkilaat ka saamna karna para?

**Interviewee:** Nahi aisa koi problem nahi hai, Allah ka shukar hai. Mujhe zyada mushkil nahi uthani pari kyunke shaadi say pheley jo du maheney thay jab yeh hospital mein thay, jab shaadi honay wali thee tou merey ghar walon ne boht mushkil uthaye thee aur susral walon ne bhi boht mushkil uthaye thee, jab meri shaadi hokay aye thee tou buss yeh sotay rehtey thay aur buss interest khatam hogaya tha. Inki apnee life thee, politics tha, karoobar tha, sogaye, kha piye liya. Meri nand ki bhi delivery thee tou mein khud bhee masroof hotee thee tou mein bhi inko itna time nahi deti thee. Tou phr inkay pass bhi merey liye time nahi hota tha tou mujhe yeh hota tha kay meiney shaadi sirf kya kaam kay liye kee hai. Ghar walon ko sambhalne kay liye kee hai, in kay pass tou merey pass time he nahi hai. Shuru shuru mein mujhe boht ghussa aata tha aur hamari lariyan bhee hoteen theen aur mein ney apni ami ko bhi bol diya tha, tou meri ami ne mujhe kaha, aur meri tabiat bhi uswaqt kharab hogaye thee kyunke merey pait pe daana nikal gaya tha tou mujhe zyada betha nahi jaata tha aur mein aur mujhe kaam bhi nahi ho pata tha kyunke bhukaar tha tou unkay zehn mein yeh baat agaye thee aur ubhi bhee hai kay shuru kay dino mein aap ne merey ghar ko badnaam kyat ha apne mekey mein, kay susral walay boht kaam karwatay hain aur shauhar time nahi deta. Phr meiney ahista ahista inkay nature ko samjha tou phr yeh set hogaye hain. Phr starting problems tou inko bhi nahi pata tha kay yeh kistarah say manage karein. Ahista ahista proper saheeh hogaye thay waqt kay saath. Expectations kay hisaab say kam tha kyunke ghar walon ko bhi inki tabiat kay barey mein pata tha tou yeh zyada ghoomney nahi jaane detey thay. Akeley nahi bhejtay thay, akele mein boht kam waqt milta tha aur itafaqan jab bhi hum bahir jaatey thay Akeley tou hamari boht lariyan hotee theen, shaadi kay shuru mein boht lariyan hotee theen, hamari banteen hee nahi thee, aur phr mein yehi sochtee thee kay meiney shaadi kyun ki lekin jab yeh maafi maang letey thay tou mein kehtee thee kay theek hai. Starting mein boht problem huwi thee, 6 saal tou merey liye boht mushkil thay lekin ubh saheeh theek hai. Aur unki naye naye tabiat zyada kharab huwi thee tou mujhe aur kisi aur ko bhi idea nahi tha kay inko kis tarah say handle karein. Ahista ahista mein inki nature ko samajh gaye aur yeh bhi meri nature ko samajh gaye. Ghar walon ko bhee lekey chal rahay hain

**Interviewer:** aur ubh istarah kay maslay masail nahi hain?

**Interviewee:** Nahi ubh tou aisay koi maslay masail nahi hai, hain hee nahi ubh tou. Mein billkul set hogaye hun. Bilkul adjust hogayee hun aur hum log boht khush hogaye hain aur ubh wahan say aye hain tou tabiat kharab hai pata nahi kya hogaya hai.

**Interviewer:** acha jab aap ko jab boht zyada beezari hotee thee tou aap ne kabhi elaidgi ikhtiar karne ka socha?

**Interviewee:** kabhi nahi socha

**Interviewer:** acha aisee kon see cheez hai jo aap ko rok rahee hai, kya aap ko boht zyada unsiat hai?

**Interviewee:** Jee mein inko nahi chor saktee aur yeh bhi nahi chor saktey aur jab inki tabiat kharab huwi thee tou unko dar hogaya tha kay meiney issay aisee baatein karlee hain kay meri engagement khatam na hua. Ubhi bhi jab yeh thoray theek huay tou phoocha mujhse kay mein 15-20 din itna out hogaya tha tou kya tum mujhe chor tou nahi dogee? Meiney kaha kay meiney itnay saal nahi chorha tou ubh kya choorhungee. Ubh yeh hai kay thora relax hogaya hai

**Interviewer:** Acha aap ke doston ne ya maikay walon ne aap ko elaidgi ka mashwara diya hai?

**Interviewee:** Kabhi mashwara nahi diya hai. Kyunke aisee koi baat nahi thee aur unko itna maloom bhi nahi tha kay yeh kehte kay elaidah hojao. Koi aisee baat nahi thee

**Interviewer:** acha aur aap ko kya lagta hai kay eik husband aur wife key beech mein jo relationship hotee hai woh zyada ahmiat rakhtee hai ya puri family ki zyada ahmiat hotee hai?

**Interviewee:** husband wife ki relationship. Agar woh saheeh hota hai tou sab kuch saheeh hota hai, agar shauhar acha hai tou sab kuch acha hai, agar shauhar hee kharab hai aur agar shauhar hee aap ka nahi hai tou phr koi kuch bhi nahi hota

**Interviewer:** Acha aur aap ko lagta hai kay aap ki relationship achee hai?

**Interviewee:** Boht achee hai.

**Interviewer:** acha aur aap ko kya lagta hai aisee kya soretahal hone chahye kay eik couple ko elaidgi ikhtiar karnee chahye hai? Generally? Aap ko kya lagta hai?

**Interviewee:** agar woh eik dusrey ki izzat na karein bilkul bhi. Tou…. Eik dusre ki izzat karna boht zaroori hai warna rishta nahi chalta.

**Interviewer:** Acha aur aap ko kya lagta hai kay eik pur sukoon khandaan kay liye konsi cheezain zyada important hotee hain?

**Interviewee:** larai kam karu *laughs* jitna kam karu itna faida hai

**Interviewer:** Acha jab aap ki lariyan boht zyada hotee theen tou aap ne kabhi marital counseling kay barey mein sunna hai?

**Interviewee:** Nahi, istarah ka khuch aisa hua hee nahi tha.

**Interviewer:** Acha tou sirf dawai letey hain?

**Interviewee:** Jee, buss dawaiyan letey hain

**Interviewer:** acha tou aap ka kya khayal hai kay marital counseling aap ko madad milsaktee hai kay batayein kay kistarah deal karein?

**Interviewee:** Haan agar shuru mein aisa hojata tou shayad mujhe itnee problem na hotee shaadi mein adjust hone mein.

**Interviewer:** agar jaisay nafsiat kay maslay hun tou aap ko lagta hai marital counseling say faida hoga?

**Interviewee:** Shayad

**Interviewer:** acha aap ko kabhi zehni dabao mahsoos hota hai?

**Interviewee:** Jee hota hai, mein bhi inkay saath reh kar kaafi sensitive hogaye hun, koi bhi baat hotee hai tu mind say nahi jaate hai, boht zyada sochtee hun. Inki baat ko itna nahi sochtee kyunke mujhe pata hai inka 2 minute ka ghussa hai, yeh khud hee bhool jayengee lekin agar susral mein mujhe koi baat pata chaltee hai merey hawalay say tou merey mind sey jaatee nahi hain. Joint family mein rehteen hun tou zahir hee see baat hai hur qism kay log hotee hain, aur phr yeh hota hai kay tumhari nand tumharay barey mein aisay keh rahee hai, aur mein agar shauhar say yeh share karungee tou yeh jayeinge clear karne kay liye, meri nand say phoochain gay tou aur baat bhar jayegee. Tou istarah kay problems kaafi zyada hotee hain. Aur phr naye naye shaadi hoye tou humein alag time bhi nahi milta. aur mujhe boht zyada tha kay hur koi khayal kareinge lekin shaadi mein shuru mein boht kaam thay aur zeemdariyan theen, aur phr log aisay kehtey hain. Aur phr mujhe hota tha tou lariyan shuru hojayege. Aur mein apni bhabhi jo maikay mein hain unsay hur cheez share kartee thee tou kehteen theen kay chor do tu jab mein kisi say baat kartee thee tou mein relax hojatee thee. Kyunke mein insay zyada baatein share nahi kartee thee kyunke zyada hyper na hojayein aur phr baat kharab hojaye aur log kahen kay bhabhi ney kaan bharey waghera. Tou aisee koi baat …tou mein share nahi kartee thee, kuch bhi nahi batati thee

**Interviewer:** Acha jab unkay sleeping patterns disturb hotey thay tou aap kay bhi disturb hojatey hongay?

**Interviewee:** Yeh hota tha kay raat kay 2 baje tak tou yeh sohee jaatey thay, haan kabhi inki neend puri nahi hotee thee tou yeh half day kartay thay jaisay dukaan dair say jaatey thay, dupher ka khaana kha kay chaleygaye. Ubhi tak yeh aisay kartay hain. Maheney mein 1 adh baar tou half day karte hee hain.

**Interviewer:** aur aap ka jo support hai aap kay husband ko madad karta hai?

**Interviewee:** Jee

**Interviewer:** Acha aur iss cheez ko yeh value kartay hain?

**Interviewee:** Jee

**Interviewer:** Acha hamaray sawal complete hogaye hain agar aap kay koi sawalat hain tou aap phooch lain?

**Interviewee:** Nahi buss yeh kab tak theek hojaongee?

**Interviewer:** Aap chahtee hain kay aap ko zyada information de jaye?

**Interviewee:** Jistarah inki ubhi condition hai tou mein chahtee hun kay mujhe support miley aur koi mujhe guide karey.

**Interviewer:** Inko job bhi diagnosis hai iskay barey mein aur information miljaye kay aap behtar deal karsakein?

**Interviewee:** Jab say umrah say aye hun inki yehi halaat hai tou mujhe nahi pata kay aagay inko kaisay lakay chalna hai. Kya pata yeh kya karienge ghar jaakey kyunke wahan per tou inko control karna boht mushkil hogaya tha. Sambalna boht mushkil hogaya tha. Aur kal say kaafi behtri hai lekin mujhe lag raha hai kay yeh kuch chupa rahain hain, inkay mind mein aisee koi cheez hai jo inko tung kar rahee hai. Mind mein hai. 3 ghantee say mein inkay pass hun kay yeh baatein karein aur agar inki baton say koi baat nikley ya koi aisee baat bolein tu humein foran bataye.

**Interviewer:** Kabhi kabhi yeh thori different baatein kartey hain tou mein buss jaana chah raha hun kay koi aap ko explain karay kay kyun aisee baatein kartay hain tou aap ki kuch pareeshani behtar hogee?

**Interviewee:** Thori behtar hogee aur thora idea hoga kay kyun horaha hai, mujhe khud nahi pata kay kyun horaha hai. Yeh kyun aisee baatein kar rahay hain aur mein doctor say baat karna chah rahi hun lekin meri doctor say saheeh tareeqay say baat nahi ho pa rahee hai. Woh apna sirf round. Mein chahtee hun kay doctor say baat karun.

**Interviewer:** Inki umer kya thee jab shaadi huwi?

**Interviewee:** 24 saal.

**Interviewer:** Inkay diagnosis ka naam pata hai?

**Interviewee:** naam yaad thaa…um bipolar disorder

***Interview Ends***
