# Supplementary material for: Exploring effects of severe mental illnesses on marriages: A qualitative study from Karachi, Pakistan
Source: PLOS Glob Public Health. 2025 Dec 23;5(12):e0005652. doi: 10.1371/journal.pgph.0005652 (PMC12725543; doi:10.1371/journal.pgph.0005652)
Supplement: S1 Data — (ZIP) [file pgph.0005652.s001.zip › Transcriptions/Case 2-6 Transcripts/Case 3/C3-3.docx]

**Case 3**

**3^rd^ November, 2015**

**Psychiatric Illness: Obsessive Compulsive Disorder (OCD)**

**Outpatient Clinics: Dr Murad**

**Interviewer:** Okay what do you do?

**Interviewee:** I teach kids like you. I teach at Beaconhouse. Geography is my subject

**Interviewer:** Okay so you have done your post graduate?

**Interviewee:** Yes

**Interviewer:** Umm, okay. So you’re currently employed.

**Interviewee:** Yes I am

**Interviewer:** Aap kitney saal say kaam kar rahay hain?

**Interviewee:** I am working at Beaconhouse since 2000. So 15 years. And before that I had also been working. Since 1995

**Interviewer:** Aap log kahan rehtey hain?

**Interviewee:** Sharafabad mein

**Interviewer:** Aur aap ki age kya hogee?

**Interviewee:** 53

**Interviewer:** Theek hai, aap ki shaadi ko kitna arsa hua hai?

**Interviewee:** Meri shaadi ko hogaye hain 5 saal.

**Interviewer:** Okay aur aap kay ghar mein kon kon rehta hai?

**Interviewee:** meri Mrs hain aur mera eik beta hai. Jo meri pheli wife say hay. And he has done his A levels and he is going to go to university.

**Interviewer:** All right, so earning members?

**Interviewee:** Two of us. My wife and I

**Interviewer:** Okay. Aur if you don’t mind telling me, what is the monthly income?

**Interviewee:** around 130 tak

**Interviewer:** umm, acha. What is your wife’s education?

**Interviewee:** She has done her graduation

**Interviewer:** Where is she working?

**Interviewee:** She is a set designer at PTV

**Interviewer:** Do you have any psychiatric history?

**Interviewee:** No. lekin mujhe ubhi memory loss shuru hogaya hai. Mein cheezain bhool jaata hun.

**Interviewer:** Okay. Theek hai. Aur aap ki family mein koi psychiatric illness hai?

**Interviewee:** No no

**Interviewer:** Inki family mein hai..aapki wife mein?

**Interviewee:** She is the only member.

**Interviewer:**  Umm, and what does she have?

**Interviewee:** OCD

**Interviewer:** aap aur aap ki wife related hain aapis mein?

**Interviewee:** Nahi

**Interviewer:** Okay. Acha mujhe batayein, kay inki beemari ko kitna time hogaya hai?

**Interviewee:** according to her, she was 16 or 17 years old when this started. Haath duh raheen hain, na paak hogaya. Waldeen ko kehtee theen, properly usko treat nahi kya and seriously nahi leya gaya, and woh cheezain develop hote hote ubh kuch zyada hogaya hai

**Interviewer:** aur ubh kya sarey waqt aisee kafiat rehti hai?

**Interviewee:**  Yes most of the time. When she goes to take the bath, she will take more than 3 to 4 hours. Istarah karkey

**Interviewer:** Inki treatment kab sey chal rahee hai?

**Interviewee:** treatment yahan per jo hai iss hawalay..hum ne kaafi koshish ki thi phele lekin saheeh say koi doctor nahi mil paya tha. Phr kisi ne refer kya in kay pass chaleyjaein.

**Interviewer:** and how long has it been since you’re being treated her?

**Interviewee:** hardly two or three months hoye hain

**Interviewer:** But aap ne pheley kisi ko dikhaya tha?

**Interviewee:** Nahi

**Interviewer:** Inhon ne shaadi say phele bhi kisi ko nahi dikhaya tha?

**Interviewee:** No no

**Interviewer:** Kisi hakeem?

**Interviewee:** yes dusri beemarion kay liye but not for this

**Interviewer:** Any manshiat issues?

**Interviewee:** No no nothing

**Interviewer:** Okay as a family, are there are any financial problems?

**Interviewee:** No nothing

**Interviewer:** Any educational issues?

**Interviewee:** No

**Interviewer:** Any health issues other than this?

**Interviewee:** Nothing

**Interviewer:** aur aapis mein are you guys happy? Is it a happy home?

**Interviewee:** Yes

**Interviewer:** and betey kay saath relationship theek hai?

**Interviewee:** yes

**Interviewer:** koi aur maslay masail?

**Interviewee:** Nope

**Interviewer:** Acha but before you guys got married, kya unhon ne yeh problem discuss kit hi? Jo yeh haath dhoney ka masla tha?

**Interviewee:** Yeh actually hamari saath hee rehtee theen. My wife…late wife..jin ka inteqal hogaya hai, she was the eldest sister and this is the younger sister and hum log taqreeban saath saath hee rehtey thay. Father and sister used to live with us

**Interviewer:** So you knew about it?

**Interviewee:** haan very much so. But it was not that extreme

**Interviewer:** Now it has gotten extreme?

**Interviewee:** Yes

**Interviewer:** But you had an idea

**Interviewee:** kay yeh hai, yes

**Interviewer:** okay and jab aap ko pheli dafa yeh pata challa tu aap ka kya reaction tha? Beemari kay taraf say? Aap ko kya lagta tha?

**Interviewee:** yeh lagta tha kay waqt kay saath saath theek hojayega. Itna seriously nahi liya tha.

**Interviewer:** acha aap ko nahi pata tha?

**Interviewee:** yes buss yeh hota tha kay bathroom ja rahee hai so she is taking a long time. Not an issue

**Interviewer:** aur jab aap ki shaadi ki baat huwi..when the suggestion was brought up

**Interviewee:** but chunkey peheley say mujhe pata tha kay yeh itna time lateen hain tu mein itna mind nahi karta tha. Lekin ubh kuch zyada mahsoos honay lag gaya…

**Interviewer:** okay aap ko aisa kya mahsoos honay lag gaya hai kay ubh treatment leni chahye hai iss waja say?

**Interviewee:** number one inki apni health. According to her, mein bari stress mein aajati hun and I have to fight. Mujhe yeh lag raha hota hai kay mujh say koi bol raha hai kay tumharay haath dubara ganday hogaye hain, and tum dubara naahlo and kalmo kay hawalay say. And religious cheezain aatee hain aur uski behurmati aati hai, tu phr yeh stress mein aajati hain. Tu yeh jab bahir aati hain tu fully drained out hotee hain. Tu humein lag raha hota kay kuch masla horaha hai unkay saath

**Interviewer:** jab aap ne shaadi ka faisla kya tha tu kya aap ke dimag mein yeh baat thi kay this could be an issue?

**Interviewee:** No

**Interviewer:** Aur aap ko support tha apne ami abbu ka aur bhen bhai ka?

**Interviewee:** sab ka tha

**Interviewer:** sab encourage kar rahay thay?

**Interviewee:** yes yes

**Interviewer:** Okay, aap key betey ne kaisay react kya to the marriage…

**Interviewee:** actually pheley see hee jaantey thay aur pheley say he ghar mein rahay hun aur that also for almost 15 to 16 years

**Interviewer:** Theek hai, aap dunu apis mein kitna bahir nikaltey hain aur logo say kitna milte hain?

**Interviewee:** Social boht zyada nahi. My first wife was very social. She was like..adbee hawalay say..shero shariye, mehfilain…hamaray eik boht bara circle tha school ka..she was also a Beaconhouse teacher. But unka thora different hai. She doesn’t go out that much. Kabhi nikal gaye tu nikal gayein

**Interviewer:** Do you think her problem plays a role?

**Interviewee:** It does. Eik tu nature mein bhi hota and zahir hai yeh cheez affect tu kartee hai

**Interviewer:** log aap say phoochtay hain aap ki wife ki condition kay barey mein?

**Interviewee:** nahi discuss hee nahi kartay

**Interviewer:** and who knows about it?

**Interviewee:** very close jo jamaray relatives hain. Jin ka ghar mein aana jaana hai

**Interviewer:** and obviously aap kay betey kay liye thori adjustment tu huwi hogi. Tu jaisay compared to how it was before, tu dynamics kaisay change huay hain aur betey kay saath kaisay change huay hain?

**Interviewee:** kis hawalay say mein samjha nahi?

**Interviewer:** Matlab aap kay betey aur aapki relationship mein koi affect hua hai?

**Interviewee:** Hamari relationship….

**Interviewer:** With her and your son?

**Interviewee:** No nothing

**Interviewer:** Okay. And umm.. subah say…

**Interviewee:** lekin mein support kar raha hota hun kay yeh nikal aye issay. Boht koshish karta hun kay choti choti cheezaon ko ignore kardun. Istarah karkay lekin woh jo cheez bethi huwi hoti hai..

**Interviewer:** Theek hai tu aap unko encourage kartay hain..

**Interviewee:** matlab mein unko rokne ki koshish karta hun kay choti choti cheezon ko ignore karu. Ub jaisay dekhain maasi jo hai kapray daal rahi hai, duri per…and wahan haath touch hogaya tu usko saaf karu, baar baar usko kehtee hai..and mein kehta hun kay agar tum ne dekhou tu kaam tu usney waisay hee karna hai *laughs* aur waisay hee kareygee jaisay kay itnay saalo say kar rahee hai. She has been with us since 10 years. Lekin kyunke uskay zehn mein eik cheez bethi huwi hoti hai tu waisay hee kareygee

**Interviewer:** aur aap ka din mein time lagta hai to take care of her? Or to talk to her? And daily routine mein jo aap ki activities hain usmein aap ka time dedicate hota hai?

**Interviewee:** very much

**Interviewer:** thora explain karein

**Interviewee:** our timings are slightly different. Kay when I come back from school, so she is at office and when she is coming back from office, so I have to go to coaching. So istarah ka hota hai *laughs* but anyway sham mein hum saath beth kay chai peetey hain

**Interviewer:** Dawai ka aap khayal rakhtay hain ya yeh khud khayal rakhtee hain?

**Interviewee:** Mein rakhta hun kyunke yeh khud chahtee hain kay mein rakhun

**Interviewer:** theek hai aur aap kartay hain

**Interviewee:** jee

**Interviewer:** Okay theek hai. Aap ka subah say raath tak din kaisa guzarta hai?

**Interviewee:** haan buss subah 6 baje uththa hun phr off to school and by 2 30 I am back and khaana khaya and phr sogaye aur phr shaam mein bachay aatey hain parhney kay liye ya mein chala jaata hun, and raat ko TV dekh liya that’s it. And agar kaheen bahir jaana hota hai tu wahan chaley gaye

**Interviewer:** Theek hai aur inki jo condition hai aap ko pata tha iss barey mein aur aap ne insay shaadi karne ka faisla kya, you knew about it

**Interviewee:** yes

**Interviewer:** aap kay kya factors thay that went into making this decision?

**Interviewee:** Woh..matlab family tha. Inka tu koi aur tha hee nahi. My wife was the only support from her side..maternal..ya jo bhi keh leen, unka bhi inteeqal hogaya tha

**Interviewer:** Okay and if you don’t mind telling me, how did your wife pass away?

**Interviewee:** She had that..you know…kya kehtey hain… Hmmm *pause* lungs ko jo hit karta hai..pneumonia…and she was like..saas ki beemari thi, unkay lungs weak hogaya thay and within 15 days, she expired

**Interviewer:** Okay very sorry to hear that. Umm, jo inki tabiat hai and you had seen it since she was living with you, but was it what you had expected?

**Interviewee:** Nahi waisay hee hai

**Interviewer:** Aap ki expectations kay hisaab say do you feel you are okay with dealing with it or is there something more on your plate? Is it like more than your expectations or do you have to deal with more than what you were prepared for?

**Interviewee:** I lived with her for 16 years. Tu kabhi hum unkay ghar reh rahay hain aur kabhi yeh reh rahi hain, kyunke inkay walid sahib bhi thay tu unka bhi inteeqal hogaya, tu humein unka bhi khayal rakhna parta tha

**Interviewer:** Okay so you knew about it fully?

**Interviewee:** Yes very much so.

**Interviewer:** Theek hai aur aap ko inki beemari ki waja say koi financial constraints hain? For example, appointments waghera?

**Interviewee:** No not at all

**Interviewer:** aap ne kabhi socha in these five years kay like ubh boht mushkil hogaya and ubh khatam kardeni chahye hai?

**Interviewee:** Nahi nahi

**Interviewer:** To separate from her?

**Interviewee:** Never

**Interviewer:** Kisi ne aap ko yeh suggest kya?

**Interviewee:** Nahi

**Interviewer:** Kabhi aisa laga kay boht zyada hogaya hai aur boht pareeshaani hogaye hai ya had say zyada irritation horahee hai?

**Interviewee:** Kabhi kabhir tu irritation hoti hee hai.. but I am used to it now

**Interviewer:** Okay and in your viewpoint, when should a couple seek separation?

**Interviewee:** Boht extreme hojata hai..merey khayal mein aisee koi situation nahi hai, Allah ka shukar hai

**Interviewer:** Do you think generally…ubh aap ki shaadi say bahir aatey hain waisay there are people with mental illnesses. OCD tu phr thora kam hai lekin bipolar..extreme conditions hotee hain, do you think in these cases people can separate or divorce? Or they should support each other if they are married?

**Interviewee:** Support karna chahye life partner ko

**Interviewer:** Commitment ki hai tu

**Interviewee:** Yes very much

**Interviewer:** Theek hai. Aap kay hisaab say eik healthy family kay liye kya cheezain zaruri hai? What are the factors in making a healthy home?

**Interviewee:** This is like you know..live and let live…aap khayal rakhaingay tu woh aap ka bhi khayal rakheyngee.

**Interviewer:** Do you think health is an important component?

**Interviewee:** very much so

**Interviewer:** aap ne kabhi marital counseling ya therapy waghera ka socha hai..kabhi zaroorat pari hai?

**Interviewee:** nahi..kab zaroorat partee hai?

**Interviewer:** kabhi kisi patient kay saath boht mushkil hojaye rehna tu lekin you seem like you’re in your control..

**Interviewee:** yes

**Interviewer:** Okay and when the situation has gotten out of hand, then when should a couple seek help?

**Interviewee:** Yeh tu aap bata saktee hain

**Interviewer:** But do you think it can be helpful?

**Interviewee:** I don’t have much information about it so I can’t say.

**Interviewer:** Okay. Great. We are done with our questions. Do you have anything to add?

**Interviewee:** No. buss jeeti rahain aur khush rahain.

The interviewee was very hesitant in providing his name. We assured him full confidentiality.
