# Supplementary material for: Exploring effects of severe mental illnesses on marriages: A qualitative study from Karachi, Pakistan
Source: PLOS Glob Public Health. 2025 Dec 23;5(12):e0005652. doi: 10.1371/journal.pgph.0005652 (PMC12725543; doi:10.1371/journal.pgph.0005652)
Supplement: S1 Data — (ZIP) [file pgph.0005652.s001.zip › Transcriptions/Case 2-6 Transcripts/Case 3/C3-4.docx]

**Case 3**

**In-patient**

**What’s your family setup like?**

First, we used to live with my mother in law and joint family when she was alive. But when she passed away, we moved away to our own home. There’s me and my husband in our home, and an adopted girl, so in total around 3 people in the house.

**What’s your source of income?**

Breadwinner is my husband, he was in the merchant navy, but after this situation, he doesn’t have a job, but we have a shop which we earn rent from, and that’s how we survive. Our income is about 20000/month. The shop owners are cooperative, they support us and lend us items and cut it from my salary bit by bit, that’s our arrangement.

**Do you have children?**

I don’t have kids, we adopted a daughter, we got her married off, she lives in Saudi. My husband loves her a lot.

**What have your husband and father studied?**

My husband has done BA, but he’s educated from London. My own father has done Inter.

**Are there any psychiatric issues in your or your husband’s family?**

My mother-in-law is my *khala* and it wasn’t a big issue but even her I’ve noticed that she used to get irritated or walked when we used to give her food, or she used to turn in bed, we didn’t understand what it was. But I noticed that. I’m not sure if it was a psychiatric issue, or something else. But I noticed it.

My own family doesn’t have any other psychiatric issues.

**How long have you been married?**

I’ve been married for 20-25 years.

**Did you know about this illness before marriage?**

He had this illness, a little, even before marriage. In fact, he’s very cooperative and loving, and I don’t consider this a disease or think it significant, because I think if one treats a patient right and with care and love, the disease can go away and disappear. My husband has a lot of phases, but I cope well, I give him medication but also love and care, and work it out with him, and it relaxes him, the support he gets from me.

I think if people irritate him and don’t care for him, he will be more in danger. So I try to go out with him, care for his diet, and that helps.

**Did anyone tell you specifically about his disease?**

No one told me he had a disease per se, he’s my cousin, *khala’s* son, so we knew he had an issue. I knew that I should stick with him and maybe I can make him better. But I can’t say whether everyone can tolerate it or not, and I think to tolerate and understand a psychiatric patient is a difficult job, and not everyone can do it. But if one wants to sacrifice, and agree to spend the life with him, it can be done. But if someone can’t bear it or tolerate it, it’s tough. Once, my husband, for 4 days was so sick, that he consistently urinated for 4 days and 4 nights, and he was walking consistently. I stayed up with him, gave up my sleep, walked with him, went to the bathroom with him or to help with his urination.

**What did your parents say when you married him?**

My parents knew before my marriage that he had an issue. And they even told me itll be tough, my sister even now says you had so many better options, yet you said yes to him, now see how many troubles you have. My parents are very loving, my husband may be sick but he is indeed very loving. But I think if there exist people like me who are ready to sacrifice in our country, everyone can be better, but people are selfish they don’t do that.

**Do people ask you about it? Do you get support from people?**

My neighbors always tell me I’m very cooperative, they commend me. Before, people used to object to his being outside, because he used to do strange things. And I explained to them that he was ill, would you do the same to your loved ones if they were sick. They understood then that a psychiatric patient doesn’t control their actions, it is a disease, but to go with it, understand it, and deal with it is not like any other disease.

**Did you ever doubt you could do this after marriage?**

I never thought he was too difficult. Or that he was too depressed, or that I should leave him. Never. Yes, it can get difficult, he has hit me 1 or 2 times, but it isn’t in his control. I made him realize his mistake, he apologized. Usually he’s fine, his phases are bad.

This was my 2^nd^ wedding. First one was with my *chacha’s* son. But we didn’t get along, he was a nice person, but I guess Allah wasn’t okay with it. He had a strange behavior, I just didn’t like the situation, he was a good guy, but after 6 months I realized we weren’t getting along. After that I sat at my home for 2 years, and my siblings said I get good proposals, I don’t have kids, I should remarry. But brother’s insistence was not to get married in the family again. My *khala* used to keep crying, she knew that I can deal with her son, and I’m the only one who can. So she used to be very convincing, wanted me to sacrifice for her son, asked me if I could. My family said no, they said it’ll be very difficult. Marry anyone but him. But in my heart, I had this determination that I could do it.

My husband has been married 2 times before me, I’m his third marriage.

**Do you guys maintain a social life?**

I take him everywhere, we go to every gathering, we talk to everyone, I don’t let him feel that he is different even he stays normal after I give him his meds. Other than the occasional phase.

**Do people ask you about his disease? Do they question you?**

People don’t really ask me as such about him. Once or twice, I noticed that when *he* goes somewhere alone, then some people asked him when they notice his mannerisms. But my sister noticed and she yelled at them to not make fun of him. They apologized.

**Directly, do you tell people what the diagnosis is?**

Yeah, if people ask me, I do tell them he has a psychiatric illness, after I give him meds he’s fine. I tell people.

**Do you ever feel that your family is different from other families?**

No, our family is very close, we are all doctors, we are from a good family.

**Whats your day like, morning to night?**

Usually, my attention is on my husband. I do some housework. I make food for him that he likes. I feel better when I do things for him. I even requested the hospital to allow home food, it relaxes me. Yesterday, they didn’t allow me to meet him or bring my food. It was really distressful. Not everyone has this determination that I have, some get irritated that their husband is sick, let him be, I don’t care, but not me.

**Were there any personal reasons you agreed to marry him and stay with him?**

No, there was no pressure on me, I just had this will in me through Allah. I was happy at home too, when I was at my parents I had a job, my brother, my sisters in law are very good people. There was no reason to marry him or any pressure. I had heard myself and seen from my eyes how difficult his 2^nd^ marriage was, how that woman couldn’t do it, so I knew it would be difficult. But in my heart, there was this determination, to make him better, my *khala* was very worried.

First he used to be normal, told me lots about his life, his work. He used to tell me about his travels. I used to ask him questions about ships, he used to tell me about it, the incidents. Even now, when he’s better he talks a lot.

**What your hopes were, in this marriage, did it meet your expectations?**

Look, every girl has some dreams, some passions. She imagines what her husband, her life will be like. For me, it was all about making my *khala* better, lessening her burdens and worries. I didn’t love him, nor was I interested in him romantically, but I just wanted to see him better. This bond between a husband and wife, it’s about care, about love, siblings can’t do that for each other: me dressing him, me feeding him, this man who was educated, who was so successful, he used to be so independent, he was married to a doctor, but they got divorced, he’s very good.

**How old was he when he was diagnosed?**

I’m not sure. I know it wasn’t in his childhood, it was after his job, he traveled, it was after that. Once, he saw someone jump from the ship. After he saw that, he changed. And also, my husband was in America when his father died so he missed the funeral. Even that had an effect on him.

Yesterday, or day before, I found out that when he was married the first time, the woman didn’t cooperate, that’s why he took this seriously.

**When you face difficulties, how do you face them? What’s your formula?**

Like recently, when he was really sick, my brother asked me, are you ready for this kind of situation. I was a little sad on hearing this. I just know that Allah only gives to people what they can handle, and I can handle it. I just pray for his betterment and health.

**Have you had any financial issues?**

Yeah, these days there’s a lot of expenses. His medicines, his treatment, it is expensive. He doesn’t have any source of income or job. We don’t have any complaints but it is difficult. My khala helps a lot financially.

**Has anyone advised you to end the marriage?**

No one has told me, and no one can try telling me this, they know I won’t end it.

**What do you think is important, for a successful bond, 2 people in a marriage or the whole family?**

I think any marriage is a challenge. After you marry, your parents are no longer there. You have to take your in laws and husband with you forward, if you husband is good and has a good understanding, if the couple between themselves have a good relationship, then it’s great. But if the man isn’t okay, evne the woman alone can handle it.

**When should 2 people divorce, under which circumstances?**

If people don’t have an understanding with each other, or if 2 people are always fighting, and don’t have a passion for each other, then they can consider it.

**What’s needed for a healthy and peaceful family?**

For a good family, you need to make sure you’re good and caring and loving with everyone. With patience, you can be a good family.

**Have you ever heard of marriage counseling? *Didn’t understand her response at all.***

I think just some understanding is enough to deal with these issues. I think that counseling is for issues with intercourse, (???).

**Do you think a third party can come and make a situation better for the couple in this kind of situation?**

Yes

**Is your husband generally caring?**

Yes, normally. When he’s not sick. He never asks me about money, or interrogates me, he’s very cooperative.

**Any questions?**

Yes, I wanted to ask, those people that run away or evade people with psychiatric illnesses or those who consider these patients a burden, how do you people deal with these people.

**We tell them to try to manage. We think our society has a taboo on this topic. Our aim through this research is also to understand what the family etc’s concerns are, so we can address them better to decrease this taboo.**

**Have you yourself ever sought any medical attention?**

Yes, I’m a diabetic, I even have joint aches. I’ve not put insulin for 4 days because I’m so focused on him and his well-being. I forget all my ails. I don’t eat for days.

**Have you yourself wanted any therapist, or someone to talk to like a psychologist, psychiatrist?**

No I always talk to Allah. My in-laws, my *nands* are very nice people. They realize what I do for their brother. They are my confidantes.

**Do you understand why psychiatric issues develop?**

I just know that when someone stresses a person, or if a person ever goes through a difficult time, that can trigger it. My husband, what he’s got, I’ve heard the 2 stressors, the suicide, and his first marriage, his father’s funeral… those things were triggers. My husband’s disease is bipolar? What does that mean?

**Sometimes they’re high, sometimes they’re low. Sometimes it can even be in family.**

He doesn’t smoke or have pan. But yes his elder brother also had a similar issue, and he used to see a doctor.

**Does your husband realize his illness? Does he take his medications compliantly?**

Yes, I give him his meds. If he refuses, I crush the meds and mix with his food or drinks and he takes it easily.
