# Supplementary material for: Exploring effects of severe mental illnesses on marriages: A qualitative study from Karachi, Pakistan
Source: PLOS Glob Public Health. 2025 Dec 23;5(12):e0005652. doi: 10.1371/journal.pgph.0005652 (PMC12725543; doi:10.1371/journal.pgph.0005652)
Supplement: S1 Data — (ZIP) [file pgph.0005652.s001.zip › Transcriptions/Case 2-6 Transcripts/Case 3/C3-5.docx]

**Case 3**

**27^th^ October, 2015**

**Psychiatric Illness: Bipolar Disorder**

The interviewee did not allow the interview to be recorded. Aged 36 years, he has been married to the patient since five years. They have two children. He feels that his wife’s mother also has Bipolar Disorder. Moreover, the grandmother has been diagnosed with a mental illness. He was informed about the mental illness before the marriage but he thought that there was complete cure for it and he did not know the extent of the illness. This is his second marriage and the wife had also been married before. Her brother made the wrong statement by saying that she just has some problem. He stated that the reason why her previous marriage broke off was because of ill-treatment from the ex-husband. He also mentions that she has seven brothers who tend to interfere a lot in his marriage. He got to know about the extent of illness after the engagement. After her marriage to him, she was in her depressed phase for around 1.5 years. And he also mentions that whenever she was in her manic state, she didn’t want to sit at home and always wanted to be out and about. His parents found out about the mental illness after the marriage. He lived with his in-laws after the marriage as well. He has accepted the illness but he feels he has been deceived. They do socialize and people do question about the illness by asking *tabiat kaisi hai*. He feels that the cyclical moods affect him a lot because she can’t perform at all during the down phase. He also feels that he is responsible for most of the things in the house, taking care of the children and at times, also cooking food. The children are too young to understand the illness, one is 4 years old and the other is 3 years old.

He thinks that had he known about the extent of the illness before marriage, he wouldn’t have married her. And he said that he has been deceived in some way because the family didn’t tell the entire detail. He thinks that she is very selfish because normally she acts completely fine with other people. The reasons for staying back in the marriage are because *nibahanay ki koshish karni chahye hai, bacho ka sochna parta ai.* He also added that *bachi hai meri bhi, yeh bhi kisi ki beti hee hain.* His friends don’t know about the illness. He also stated that he feels alone and the fact that he has to take care of everything.
