# Supplementary material for: Exploring effects of severe mental illnesses on marriages: A qualitative study from Karachi, Pakistan
Source: PLOS Glob Public Health. 2025 Dec 23;5(12):e0005652. doi: 10.1371/journal.pgph.0005652 (PMC12725543; doi:10.1371/journal.pgph.0005652)
Supplement: S1 Data — (ZIP) [file pgph.0005652.s001.zip › Transcriptions/Case 2-6 Transcripts/Case 3/C3-6.docx]

**CASE 3- TRANSCRIPTION**

**Marriage duration- 2.5 years**

**Nuclear family system**

**Owns a small tailor shop (8-9 years)**

**Aap ka naam?**

Mera naam hamza hai

**Jee theek hai, aur aap ki umar?**

Meri umar abhi batees saal se upar hai

**Theek, aur aap ne kahan tak perha hua hai?**

Paanch class tak

**Aur abhi aap koi kaam kerte hain?**

Haan meri choti se shop hai, darzi ka kaam kerta hoon mein

**Aur kitne saal hogaye taqreeban ye kaam kerte hue aap ko?**

Aath nau saal hogaye dukaan ka kerte hue way

**Acha, aur shaadi ko kitne saal hue hain?**

Shaadi ko do dhai saal horahe hain

**Aap log akele rehte hain ya bari family, saath sab rehte hain?**

Alag rehta hoon mein , ham do alag hain

**Toh taqreeban aap do log ghar mein hain, aap ke koi bachay waghera?**

Nahin bachay toh koi bhi nahin hain, ham do alag hain, walda log alag hain.

**Theek hai, toh agar batana pasand kereinge toh taqreeban kitni aap ki amdani hai meehene ki?**

Amdani ye hai ke ghar ka kharcha chal sakta hai. Bimaar hoti hain toh kaam pe jaata nahin hoon mein. Uske peechay hona perta hai.

**Taqreeban maheene mein kitna aap kama lete honge?**

Maheene ka toh pata nahin. Karaye ki dukaan hain. Dono cheezein keraye ki hain hamari

**Acha sahi, inko jaise nafsiat ki bimari hai toh iski waja se aap ko hai kuch, jaise koi pereishani?**

Nahin nahin, mere ko toh aisa kuch nahin hain. Mein koi nasha wagherha kerta nahin hoon kuch bhi nahin siwaye chahliya ke elawa. Mein kaam ke andar hota hoon toh kuch pata nahin chalta hai wese

**Theek hai. Aur khandaan mein inki side se ya aap ki taraf se kisi aur ko hai?**

Wese zada tar iske ghar ki tension hai thore bahot dinon ki. Khandaan ke hisaab se lag raha hai mujhe

**Sahi. Theek hai. Toh inki bimari ko aap keh rahe hain shaadi se pehle aap ko maloom tha, toh takreeban kab se hai inko ye?**

Samjhlo ke shadi se pehle tha ye inko. Kamasmkam paanch saal ya chaar saal pehle tha ye inko.

**Theek hai. Toh beech mein ye theek hojaati hain phir thora sa kuch hota ya ye hamesha se aisi hain?**

Shaadi ke baad samjho ke do saal ke baad jakay iski bimaari hogayi wapsi. Mangni hui thi, mangni ke baad do saal ke baad jo isko bimari hui thi jo uske baad jo bimaar pari hai toh abhi tak hee bimar horai hai

**Acha. Aur dawaiyon pe hain ye?**

Abhi dawai wohi hai.

**Kitne salon se takreeban dawaiyon pe hain?**

Mere khayal se doctor ke paas bahot time hogaya. 2008 se chal rahi hai

**Hmmm theek hai. Toh aap ko pehle inki bimaari ka pata tha. Toh hamesha se doctor ke paas aatay hain ya pehle kaheen aur homeopathic ya kaheen aur jaatay thay?**

Koshish toh bahot ki. Kaheen se kerwanay Hyderabad gaye thay. Ab is mein ye hai keh setting aajati hai. Yahi chor deti doctor sahib bolte hain keh iski bimarri aisi hai ke wapsi return ajaye gi. Agar sahi rehege toh kabhi bhi nahin hoga. Khushh rahegi jaise kaam pe hogi toh phir bimaari chali jayegi iski. Bilkul aisi bimari hai chali bhi jaati hai agar ye zada kaam meie dehaan de, khush rahay.

**Hmmm. Toh wese aap kisi aur ke paas jo homeopathic ki dawai dete hain ya koi peer weer jaise ke paas gaye hain?**

Dua kerwayi hai, dua kerwayi hai jaise bimaari chali jaye, ghar mein sukoon hojaye.

**Sahi. Aur ye kisi kism ka nasha ya kuch kerti hain?**

Nahin nasha toh kuch bhi nahin kerteen

**Chahliya, paan?**

Ye chahliye waghera kerti hain. Bahot kum.

**Theek hai. Aur aapko kisi kism ki maali mushkilat ya shaadi ke hawalay se koi mushkilat ya kisi kism ki koi mushkilat ka saamna kerna perta hai?**

Zada yahi ke yahan ki fees zada hoti hai iski zada tension hai baqi goliyon ka ye hai keh mein ker sakta hoon.

MAIN INTERVIEW QUESTIONS-

Sahi. Theek hai. Aap ki shaadi aap ne kaha dhai saal se hui wi hai.

**Theek hai. Aap ki biwi jo hain unhon ne aap ko kab bataya tha apni bimaari ke baaray mein?**

Bimaari ka mujhe pata tha.

**Toh unke ghar walon ne bataya tha?**

Mujhe pata tha. Mere elaake mein rehti hai mere saamne bimaar hui thi. Toh mujhe pata tha sab kuch.

**Theek hai. Toh aapka inki bimaari ke baaray mein kya rad-e-amal tha? Aap kya mehsoos kerte thay?**

Mein ne kaha tha hogaye gi tabiyat sahi. Shaadi hojayegi, Elaaj keraoon ga mein, Allah keray sahi hojayegi.

**Sahi. Aur aap ke waldein ko maloom tha inke baray mein?**

Sab ko maloom tha

**Toh unka iske baray mein kya khayal tha, kabhi unhon ne bola ke nahin kero?**

Unhon ne toh bola tha. Zindagi ka sawal hota hai. Shaadi ka faida kya. Kisi achi larki se kero kamse kam sahi hojaye. Bimari ke peeche lagoge toh gharka kereiga (don’t get what he’s saying) – 4:38mins –

**Toh wo asaani se maan gaye thay?**

Who toh asani se maan gaye thay meri khushi mein usne kerdiya, bola chalo

**Theek hai. Aisa kabhi hua hai in peechle do dhai saalon mein ke aap ne socha ho ke nahin kerta bas choor doon mein ya kuch?**

Chorne mein isko chor ke kya milega mujh ko

**Hmmm. Toh kabhi aisa ke aap ne pachtaya ho ke kyun kiya?**

Nahin. Pachtawa toh nahin hai. Ye dil mein khushi hai ke yahan aakar sahi hojaye.

**Theek. Toh aap logon ka wese bahar aana jaana hota hai, doston mein milna milaana?**

Nahin. Iski tabiyat kharab hoti hai toh mein nahin ja sakta hoon.

**Hmm. Aur wese agar maheenay mein dekhein toh kitni dafa aap log bahar aatay jaate hain saath?**

Ye khud derti rehti hai. Bolti hai mein kaheen aa jaaon wapsi bimaar per jaaon. Toh khud kaheen aajaati bhi nahin hai.

**Aur log jab aap se sawalat kerte hain keh inko kya hai bimarri toh aap kya bolte hain? Batate hain inko sach ke inko kya hai? Kyunke yahan pe jaise logon ko samajh nahin aati? Itni samajh nahin hai is cheez ki toh aap ka kya bolte hain unko?**

Mein bolta hoon uske ghar ki tension hai. Uski wajah se tabiyat sahi nahin rehti hai. Aise kerke masla hall kerdeta hoon. Ab kisi ko pata na chalay

**Jee bilkul. Ab aap ko kaisa lagta hai, kabhi aap ko aisa lagta hai keh aap dusre khandaan walon se mukhtaliff hain? Ya kuch alag alag sa lagta hai?**

Haan alag lagta hai. Mein sab khushiyon se door hoon, iski wajah se.

{*participant slightly distressed*}

Sahi. Theek hain aap? Thoray se hotay hain aisay sawal lekin haam jaana chahtay hain keh aap kya mehsoos kerte hain.

Nahin achi baat hai.

**Toh wese aapka din kaisa guzarta hai? Subha se raat tak aap kya kerte hain?**

Mein 12 bajay tak shop pe jaata hoon phir raat ko 9 bajay aata hoon phir raat ka time iske saath guzar jaata hai

**Toh aap subha kitne baajay tak uthtay hain?**

11 bajay uth ke 12 bajay shop pe jaata hoon

**Aur waapsi kitne bajay?**

9-10 bajay ghar pe

**toh bachon ka aap logon ne khudi faysla liya hai keh abhi nahin jab tak inki tabiyat theek nahin hojati ?**

nahin doctor sahib ne mana kiya hua hai keh abhi inki tabiyat aisi hai

**theek hai. toh aapki apni aisi kya zaati wajoohat theen keh aap ne bimaari ka pata hone ke bawajood itna bara faysla kiya ke nahin kerunga mein shaadi.**

Nahin zidd wali baat toh nahin thi. Lekin ye hai ke ghareeb hain thoray se, iska waalid bhi nahin hai. kisi ka sahara banjaoon toh achi cheez hojayegi

**Aur jaisa aap ne socha tha do dhai saal pehle, bilkul wesa hai ya bilkul alag soch se is shaadi mein aye thay? Banda shadi kerta hai toh kuch cheezein sochta hai keh is tarah hoga?**

Haan khushiyan hoti hai but wo khushiyan mili nahin hain

**Jab aap kabhi pereishan hojaate hain ya akelapan mehsus kerte hain is mushkilat mein toh aap kis tarah usko sahi kerte hain**?

Yahi isko daant deta hun, ghussa aata hai. aur kya ker sakta hoon.

**Sahi. Aur koi tareeqa. Aap thora sa pereishani ko kaise kaam kerte hain?**

Perishaani usko dekhta hun, thora ghussa kerta hun, haath bhi utha leta hoon, phir wapsi mein agar ghussa kaam hojaye toh raazi kerlete hain.

**Theek hai. toh maali mushkilaat hoti hai jab aap kaam pe nahin jaatay toh phir kaise hota hai aap ka saara kaam?**

Bimaar hojati hai toh jaanay nahin deti. Saara din uske paas baitha perta hai. toh ye hai ke saas waghera hoti toh uska khayal kerti.

**Abhi koi kehta hai keh bas chor do, is shaadi ko khatam kerdo?**

Nahin ye nahin bolte. Mazak urate hain jab dekhte hain. Bas kehte hain shadi kerke kya mila tere ko.

**Toh aap ko koi bolta hai keh bas chordo?**

Chorne ka toh nahin bola hai. baqi ye hai ke ghar wale ye bolte hain keh koshish kero achi hojaye. Tabiyat sahi hojaye. Chornay ke toh abhi tak nahin socha hai, bas koshish kerta rehta hun keh sahi hojaye.

**Sahi. Wese toh kuch sawal bachon ke baaray mein. Lekin aapke apne khayal mein shadi do logon ke beech mein zyaada ehmiat rakhti hai ya puray khandaan ki zada ehmiat hoti hai?**

Mil jul ke rehne mein zada acha lagta hai. ye alag hain, toh khud ko kerni perti hai

**Aap ke khayal mein ek shauhar aur biwi ko kin suratehaal mein talaaq leni chahiye?**

Talaaq honi chahiye. Talaaq isliye banayi hai kya pata uske tayvar/tareekay ghalat hon. Najaiz tareekay hotay hain. Kkuch cheezein aisi hoti hain jo bardasht nahin hoti, us waja se honi chahiye. Baqi is wajah se tohnahin honi chahiye. Koshish kerni chahiye, bhalay dusri keray per agli ko uska sath dena chaiye. Koshish ye honi chahiye

**Theke hai. aur aap ke khayal mein. Ek khush aur purskoon khandaan ke liye aisi kya cheezon ki ehmiat hoti hai?**

Zada tar toh bachay hotay hain. Ussay dekh ker biwi ki ghaltiyan bhi hoti hai toh ghar walay saray maaf kerke bachon ki khushi mein usko maaf ker dete hain. Kuch toh apne bête ki khushiyon mein ker lete hain. Bahu aisi hoti hai usko bardasht kerna perta hai.

**Kuch shahaur biwi mein aise nafsiat ke maslon ki wajah se ikhtilafat hojate hain. Toh ek cheez hoti hai marital counseling jis mein doctor dono ke beech mein maslay suljhaatay hain. Toh iski baaray mein aap ka kya khayal hai?**

Woh salah denge, mashwera denge toh kya pata usko samajh mein aajaye toh achi baat hojaati hai.

**Toh aap ko lagta hai issay faida hosakta hai?**

Hosakta hai. samjhayega doctor sahi tarah toh usse samajh mein aasakta hain. Kyun ke ham jo sochrahe hain kya pata ghalat sochte hain. Doctor jo samjhata ho wo sahi ho, aisa ho sakta ho haina.

**Sahi hai. chalein sawal toh saaray pooch liye. Aap ko kuch aur kehna ho jisse hamein faida ho toh batayein..**

Koshish toh ye hai keh ham ghareeb banday hain. Toh kam se kam kuch ghareeb bandon ke liye ho, kyun hamara kiraye ka makaan hai aur dukaan hai.

Aur paison ke elawa kisi kism ki koi cheez.. Pakistan ke hisaab se pooch rahi, kisi aur kism ki madad se aap ko asaani ko- paison ke elawa.

Paison ko elawa toh bas upar walay ki merzi. Tension hoti rehti hai ghareeb bandon ki kirayi ke makaan hotay hain. Doctor sahib toh kam kerdete hain. Koi aisa banda ho jisko maheenay mein 3000 bachtay hon toh khaana khayega kya kereiga kuch nahin kersakta..

**----the end-----**
