# Supplementary material for: Exploring effects of severe mental illnesses on marriages: A qualitative study from Karachi, Pakistan
Source: PLOS Glob Public Health. 2025 Dec 23;5(12):e0005652. doi: 10.1371/journal.pgph.0005652 (PMC12725543; doi:10.1371/journal.pgph.0005652)
Supplement: S1 Data — (ZIP) [file pgph.0005652.s001.zip › Transcriptions/Case 2-6 Transcripts/Case 4/C4-1.docx]

**Case 4**

**Illness:** Bipolar Disorder

Admitted in ward

13^th^ October, 2015

The husband did not know about the illness before the marriage. The patient’s sister accompanied the husband for a while but then left. The husband mentions that she was taking medications before marriage and after their engagement, they used to talk on the phone but she never mentioned that she was ill. Even his parents did not know about the illness. However, he mentions that her family has supported him a lot during this time, and paid for the medical expenses as well. When asked about her symptoms, he mentions that it was mainly *shak-u-shuba.* And that *betuki baatein kartee thee.* However, he also mentions that there were some phases when she was completely normal, and this was when her mood was good. She was admitted in the hospital because she wasn’t eating anything saying someone had mixed poison in her food, and kept on saying that everyone was her enemy. Therefore, he contacted her family and they said that she needs to be admitted in the hospital. When asked about the first reaction to the illness, he said he didn’t think it was actually an illness before but now he has come to the realization that this is an illness. When asked whether he could fix her, he said that *umeed pe dunya qayam hai.*

He says that during the time she has been admitted in the hospital, he faces a lot of hassle. He has faced many difficulties. When asked whether she appreciates the fact that he takes care of her, he said that yes, but only when she is in a good mood. When asked about their socialization patterns, he stated that she wants to be alone and does not want to come out of the house, and he always wanted her to come with him. He just goes out with friends. Everyone knows about the illness especially when she tried to commit suicide by drinking hydrogen.

When asked if he would have married her had he known about the illness before the marriage he stated “*karni bhi chahye hai aur nahi bhi karni chahye. Doubt ho tu nahi karni chahye, and ubh karlee tu zindagi guzar jayegi”*. He then went onto say that he would have still married her.

He feels that the family dynamics have changed because of her behavior. And it was not because of the illness (however, of course it was because of the illness that her behavior was that way!)

He did not mention the family’s reaction stated that he has not talked to anyone in the family.

When asked whether his relationship has changed, he feels that it has not.

He declined to answer when it was asked about the reasons why he wants to stay in the marriage. He said “*aap kuch aur phoochlein”* (note he did not mention his wife was pregnant with their first child).

He mentions that she did not do any of the household work and his mother and sister took care of that but he did not have to take up any kind of additional responsibilities. Says he does not have a lot of leisure time and when he does, he spends it with his friends.

He realizes that she will probably have to take medications in order to be stable. Which means that there is at least some understanding of the illness.

He also feels that it is not her fault to have the illness.

When asked the circumstances for divorce, he stated only when someone is cheating on their spouse, that’s when divorce should be considered, otherwise no.

No one has also suggested divorce to him

He also stated that what is conducive to a healthy family environment includes the fact: *khush raho aur khush rakho’*.
