# Supplementary material for: Exploring effects of severe mental illnesses on marriages: A qualitative study from Karachi, Pakistan
Source: PLOS Glob Public Health. 2025 Dec 23;5(12):e0005652. doi: 10.1371/journal.pgph.0005652 (PMC12725543; doi:10.1371/journal.pgph.0005652)
Supplement: S1 Data — (ZIP) [file pgph.0005652.s001.zip › Transcriptions/Case 2-6 Transcripts/Case 4/C4-2.docx]

**Case 4**

**Psychiatric Illness: Bipolar Disorder**

**Outpatient Clinic: Dr. Hanif Mesiya**

No recording is available. They live outside Karachi and belong to a low-income household. They have been married for the past 3 years and he mentions that they have a troublesome marriage because of her entire attitude.

- His brother got separated from the house because of the patient’s attitude towards her brother-in-law and his wife
- His parents are also quite affected by it.

When asked whether he has any psychiatric history, he mentions that he is now getting affected severely by her illness. The marriage was arranged and they are not related by family. He took her to different doctors and psychiatrists and then one of his cousins who is a doctor told him to come to AKU. He mentions the extent of his illness which is the fact that even the neighbors are disturbed because of her attitude. In fact, one of the neighbors has stated “*mein illaqa chordungee agar aap nahi jayenge*’. He also mentions that he has thought of divorce multiple times *kabhi kabhar halaat aisay hojate hain kay yehi karnay ka dil chahta hai.* But he adds that *mein bhi kisi beti ka baap he hun, kisi aur ki beti ko kaisay yeh karun*. Divorce has been suggested by family members and relatives.

He adds that he realized after a few months into the marriage that she is mentally unstable. After she had her first child, that is when her first episode in front of him occurred but when they consulted a doctor, he stated that this illness was present beforehand.

She does not do much work around the house and will only work whenever she is in the mood. Support system is also present. His relatives do help him a lot. *Inki kisi say nahi banti, mujh sey bhi nahi banti, yeh abnormal nahi hain, buss inka mijazz he aisa hai*

He has to take care of her a lot because she is also not compliant with medications. He also gives her food at times.

And he also adds that taking her to the hospital for check-ups is an issue and they miss appointments because of the expense involved *dur aana tu lazmi hai, majboori hai. Maslay boht hain. Yeh non-stop boltee hain. Meri maano, meri sunu*

Also adds that she wants money all the time. And does not acknowledge that he provides her any kind of support at all.

When asked about socialization he stated *milna julna hota hai lekin inki behavior ki waja say sab kut gaye hain*.

People do know about the illness and he stated that he tries to hide it, but relatives know about it and the neighbors complain about her a lot.

When asked had he married her if he had known about the illness, his answer was a no.

Family dynamics have changed completely since she doesn’t get along well with anyone.

He has a tough life because he works for 12 hours per day. He has taken additional responsibilities because he has to take care of their daughter and also cook and clean.

*Rishta hai, chal raha hai*

When asked what he does in leisure time, he said *farig waqt boht kam milta hai but yeh kabhhi chortee bhi nahi hai.*

*Buss mein sochta hun kay mein eik beti ka baap hun*

When asked whether he can fix her *koshish karsakta hun. Sahi karsakta hun yeh nahi pata, yeh tu uper walay ko pata hai*.

He mentions that in his family, divorce is not a good solution. *Mian biwi ko nibhana chahye hai*.

There is certain amount of violence because she has also beat him up and he has also retaliated.

He also kept on asking the researcher whether he should stay in the marriage and if the researcher would have been in his situation, what would she have done?
