# Supplementary material for: Exploring effects of severe mental illnesses on marriages: A qualitative study from Karachi, Pakistan
Source: PLOS Glob Public Health. 2025 Dec 23;5(12):e0005652. doi: 10.1371/journal.pgph.0005652 (PMC12725543; doi:10.1371/journal.pgph.0005652)
Supplement: S1 Data — (ZIP) [file pgph.0005652.s001.zip › Transcriptions/Case 2-6 Transcripts/Case 4/C4-3.docx]

**Case 4**

**Psychiatric Diagnosis:** Obsessive-Compulsive Disorder

**Outpatient Clinic:** Dr. Murad Moosa

*briefs about the purpose of the interview, signs the consent form*

**Interviewer:** Inka diagnosis kya hai?

**Interviewee:** OCD

**Interviewer:** Agar kisi bhi waqt aap ko nahi jawab dena ho ya interview ko continue nahi karna ho tou please batadijiyega

**Interviewee:** Nahi aap ko jo bhi sawal karna hai aap karein lekin iss ko market mein discuss na karein

**Interviewer:** Nahi nahi, aap ka naam bhi nahi ayega, research mein sirf use hoga.

*fills out the demographic form- the information is in the form (self-employed, has a real estate business, earns around 50,000 to 1 lakh. They have one son. *

**Interviewer:** shaadi ko kitne saal hogaye hain?

**Interviewee:** 6 saal hogaye hain

**Interviewer:** inka jo yeh problem hai?

**Interviewee:** Shaadi say pheley tha

**Interviewer:** aap ko maloom tha?

**Interviewee:** nahi maloom tha

**Interviewer:** tu unki family mein kisi aur ko hai?

**Interviewee:** inko jaisay hee hua hai tou buss foran say shaadi karadee. Baatein kuch dimag mein beth gaye hain. Doctor ne kaafi ilaaj kardiya aur ubh Dr. Murad Moosa kay pass forward kardiya. Aur jo yeh beemari haina yeh khud chahengee tu jayegee. Jo shaadi say phele ghar mein kaafi kaam waghera kartee theen, achanak insaan bara hota hai aur khayalat kaheen aur jaatey hain tou yeh hojata hai. U-turn aata hai na zindagi mein. And mera jo beta hai, isko boht laad mein rakha hai, lekin jab yeh saath saal ka hoga tou mein issey namaz sikhaonga, phr yeh U-turn leyga. Aur unki zindagi mein U-turn hogaya. Issue tou koi bhi nahi hai inka, ghar mein na koi saas hai na susr hai, na nand hai. Hur cheez hai lekin yehi baat hai kay jo puraani baat dimag mein woh nikal nahi pa rahee. Nikal bhi jaate hai, du wajahain hai, eik taleem ka hai, matric tak parha hai, lekin 5 class ki barabar hai. Tu boht bara aisa hota, general knowledge ka koi shauq hai aur apne andar mast hai, idher aakey set nahi hoye. Yeh community ki hain,

**Interviewer:** shehr mein akey set nahi hun?

**Interviewee:** maqsad yeh nahi hota… *stops talking*

**Interviewer:** aap ko inki beemari kay bare mein kab pata challa?

**Interviewee:** Mujhe? Mujhe tou shaadi kay dusre din pata chal gaya.

**Interviewer:** aap ko nahi bataya tha shaadi say phele?

**Interviewee:** Nahi. Jab shaadi huwi thee tu humaray walid ko meiney bataya meiney tu inhon ne kaha kay hamari family mein kabhi divorce nahi huwi hai aur ubh usko chalao. Tu hum ilaaj kara rahay hain jaisay yeh keh raheen hain. Mujhe inka doctor change karna tha kyunke mein 3 saal sey yahan araha hun tu mujhe koi change in response nahi mil raha hai. Iskay muqablay mein Dr. Haroon hain. Meri jo sister haina unko yeh bhi masla hua tha, baradari mein log apnee ana rakhtay hain tu larkay walon ne kaha kay humein housewife chahye hain aur yeh lawyer hai tu unko show piece banadiya. Tu unko yeh masla hogaya aur phr unka 3 saal tak yeh masla chalta raha aur phr Dr Haroon ne eik Sert goli dee and ilaaj hogaya aur woh saheeh hogaye hain and yeh udher nahi jaate hain. Boht acha doctor tha

**Interviewer:** aap logo ki aapis mein rishtedaari hai?

**Interviewee:** haan dur ki hogee. Meri nani aur unkay dada rishtedaar hain. Inkay saath koi masla nahi hai mein samajhta hun buss jhooti parwarish hogaye hai.

**Interviewer:** Theek hai, mein kuch sawal phoochongee. Yeh kitnay saaloon say dawaiyon pe hain?

**Interviewee:** 6 saal say hongee

**Interviewer:** Doctor kay illawa kisi say ilaaj karwaya?

**Interviewee:** Inhon ne kaafi kaha tha kay pani waghera dum karwa dete hain.

**Interviewer:** aur yeh kisi qasm ka nasha karteen hain?

**Interviewee:** Buss chaliya waghera khateen hain

**Interviewer:** Maali muhskilaat ka saamna karna parta hai?

**Interviewee:**  2011 say 2014 tak thora masla tha.

**Interviewer:** ubh jaisay aap ko yahan aana par raha hai tu aap kaam chorkay aye hain

**Interviewee:** mera kaam property ka hai tou itni taqleef nahi hotee. Eik din kay liye hain aye hain phr wapis chaley jayengee

**Interviewer:** aur aap ko apni sahet kay barey mein koi pareeshani hai?

**Interviewee:** Haan, bilkul hota hai. Jee yeh aksar kaam nahi kar patee tu meiney office mein eik peon larka rakha hai tou mein leleta hun aur eik jo hota hai, mijaaz waghera hota hai, woh kami hai.

**Interviewer:** aur shaadi shuda zindagi mein koi maslay masail hai?

**Interviewee:** nahi

**Interviewer:** acha aur aap ke rishtedaroon say inkay kya talooqat hain?

**Interviewee:** hain hee nahi

**Interviewer:** Tu aap kay khayal mein shaadi say phele yeh masla tha?

**Interviewee:** khayal nahi hai, 100 percent yeh hai.

**Interviewer:** Bachpan say?

**Interviewee:** nahi meiney aap ko bataya na kay inki sister ki engagement toot gaye thee.

**Interviewer:** Theek hai aur aap ko shaadi kay dusre din pata challa gaya?

**Interviewee:** jee

**Interviewer:** aur aap ne kaha kay aap kay walid sahib ko pata hai, aur unhon ne aap ko kaha tha kay hamaray yahan talaaq nahi hotee?

**Interviewee:** haan

**Interviewer:** Theek hai tou inhon ne kaha kay isss shaadi ko barqarar rakha hai?

**Interviewee:** Jee

**Interviewer:** Acha aur aap ka support system kaisa hai? Koi aap ko madad miltee hai agar inki waja say ghar mein koi pareeshani waghera ho? Aap kay pass koi hota hai?

**Interviewee:** Nahi

**Interviewer:** kya pareeshani hoti hai?

**Interviewee:** nahi kuch nahi, buss Allah tawakal karkey bethay hain.

**Interviewer:** Buss kuch bhi nahi?

**Interviewee:** Nahi buss complain karna bhee beeqar hai, samjhana bhi beeqar hai. Kyunke mein jo chahoonga woh unki soch mein nahi ayega aur jo inki soch mein ayega woh meri ussmein nahi ayega.

**Interviewer:** Tu uski waja say aap ko koi mayoosi hote hai?

**Interviewee:** Nahi, buss challa rahay hain tu buss chalana hai, iss mein kya hai. Baradiri hai, rishteedaari hai, tou buss chalane wali baat hai.

**Interviewer:** Tu kisi qasm ki koi thakan waghera? Aur agar ghussa aata hai?

**Interviewee:** agar ghussa aata hai tou rest karta hun. Ami kay ghar waghera bhej deta hun. Waisay tou mein rest leleta hun ghar.

**Interviewer:** aur aap ko jo beta hai woh inkay saath hota hai?

**Interviewee:** haan ini kay saath hota hai. Maqsad buniyaadi tor pe yeh mujh say mohabaat bhi karte hai aur hamdardi bhi kartee hai, lekin yeh beemari inki soch ko rok kay bethi hai. Woh usko aage nahi aaney deti. Yeh boht zyada khush hotee hain jisdin meiney inko bataya kay meiney Lakh rupiya kamaya hai. Inko paisay ka kuch pata hee nahi hai. Hum jab Hajj par ja rahay thay tu meiney Gold bhaich diya tha aur ubh meiney wapis dila diya jab merey pass aye. Lekin yeh cheezain tou chaltee rehtee hain lekin jo main masla hai yeh hai kay taqleef ki waja say yeh haath dhotee rehtee hain, for half an hour, aur eik ya du din mein sabun khatam hojata hai. Ubh inkay ghar walon ko batate hain kay beemari hain, mein bhi samajh raha hun kay yeh beemari hai, woh bhi samjahtee hain aur aap bhi samajh sakteen hain, lekin yeh log zeemdari nahi lete.

**Interviewer:** saheeh. Aap inko kaafi samajhtey hain tu kya inko issay madad miltee hai? Jaisay aap kay honay say support miltee hai?

**Interviewee:** haan inka apna ghar hai tou zahir hee see baat hai khushee tou hai. Ma kay ghar kab tak? Du din char din. Akhrajat waghera tou mein hee deta hain. She comes back in 10-15 days

**Interviewer:** unkay kitne bhai bhen hain?

**Interviewee:** 4 bhenain aur 2 bhai hain aur sab shaadi shuda hain

**Interviewer:** aur aap logo ka bahir aana jaana aur ghoomna phirna hota hai?

**Interviewee:** Haan buss jaatey rehtey hain

**Interviewer:** aap ki iss baat ke tension nahi hotee kay yeh bahir jaakey haath dhotee rehengee?

**Interviewee:** Nahi wahan tou mein saath saath hota hun. Saath khaya. Shuru mein tabiat boht zyada bigri huwi thee. Ubh tou behtar hogaye hai. Inka khaana waghera, meiney apa rakhee huwi theen, aur maasian rakheen huwin thee ghar mein. Three times maasi hee khana pakatee theen aur phr yeh ahista ahista set hotee gaye

**Interviewer:** acha log jab aap say sawal phoochtay hain, kay unko kya hua hai tou aap kya jawab dete hain?

**Interviewee:** hum ne kisi ko bataya hee nahi hai

**Interviewer:** log phoochtay hain aap se?

**Interviewee:** Nahi

**Interviewer:** rishteedar waghera?

**Interviewee:** Nahi kisi kee himat nahi hai. Na bhai waghera

**Interviewer:** Acha

**Interviewee:** aur wohi sab ko pata hai lekin koi phoochta nahi hai aur na mein batata hun

**Interviewer:** acha waisay ghar ka mahool kaisa hai? Bachay ki taraf kaisee hain? Baaqi khandoono say mukhtalif?

**Interviewee:** Haan iski parwarish mein apni tareeqay say karna chahta hun aur yeh tu tara say baat kartee hai. Tu mein kehtee hun.

**Interviewer:** Jaisee inki beemari ki waja say kabhi bachay pe kuch karte waghera hain?

**Interviewee:** nahi kabhi kabhar yeh batata hai kay aaj mama nay daanta tha, aur yeh bhi maan bhi lete hain. Aisa hota nahi hai, buss yeh hota hai kay aaj humein Karachi aana tha, 8 baje ki train thee aur humein 7 baje uthna tha. Lekin hum inki waja say late hogaye aur phr dusri van ka intezaar kya aur isski school ka bhi aksar yeh hota hai, aankhein nahi khultee, aur phr chutti waghera hojate hai

**Interviewer:** Acha aur jab aap ko pata challa, aap keh rahay thay kay aap ko shaadi kay dusrey din hee pata chal gaya tha tu phr aap ka kya radeamal tha?

**Interviewee:** Kuch bhi nahi

**Interviewer:** aap ko aisa mahsoos nahi hua kay kyun nahi bataya mujhe?

**Interviewee:** Nahi, usski waja yeh hai kay merey saath bhi yeh masla tha, hamari walda ka inteeqal hogaya tha 1998 mein. Ma ka baad 11 bhen bhai thay aur abbu hamaray akhree umer mein thay aur mein bhi unsay naraz tha, aur mein idher Karachi agaya tha, 1998 say 2004. Warasat waghera mein bhi masla tha. Abbu nay shaadi waghera karwayee. *tells details about his inheritance issue which is quite irrelevant to the interview*

Aisay cases ko combined family nahi challa saktee. Akela hee rehna parta hai. Aik mera dost hai aur uskay saath bhi yehi masla hai, baat yeh hai shauhar hee challa sakta hai. Aur koi bhi nahi challa sakta. Combined family mein tou yeh aur pagal hojatee hai. Aur meiney inki khatir merey 5 bheno aur 6 bhayon say larai hogaye. Meiney aana jaana band karne kay liye ghar mein firing waghera bhi kardee thee. Aur phr meiney sab ko manna liya

**Interviewer:** Yeh kehteen thee?

**Interviewee:** Yeh depression ki shikaar theen tu unmein faisla karnee ke kuwat nahi thee. Tou yeh hum… hum confused hojatee theen. Doctor ne bhi kaha tha kay jin logo ko unsay allergy hai unko unsay dur kardou. Phr meiney dur kardiya aur mein jaata hun apne bhai bhen kay waghera, inko nahi leke jaata hai. Bhai alag waghera say aatey hain aur yeh bhi aate hain aur manna nahi kartee lekin yeh kehteen hain kay koi interfere na karey.

**Interviewer:** Acha. Aap ka beta kitne saal ka hai?

**Interviewee:** 5 saal ka hai

**Interviewer:** Acha aur isko samajh hai kay isski ami ko kya hai?

**Interviewee:** pata ahista ahista par jayega.

**Interviewer:** Phoochta hai?

**Interviewee:**  Nahi

**Interviewer:** Aur jab bhi yeh doctor kay pass aate hain tou aap humeisha saath aatey hain?

**Interviewee:** Haan.

**Interviewer:** Theek hai. Thoray say aur sawal. Aur aap dunu ka jo rishta hai, humeisha say aisa hai ya koi tabdeeli waghera aye hai in 6 saalo mein?

**Interviewee:** Nahi humeisha say aisa hee hai. Shuru mein tou boht zyada disturb tha lekin ub saheeh hogaya hun aur unko kehta hun tu yeh baat mannay ki koshish kartee hain

**Interviewer:** aur jo unka zehni dabao hai kya aap ko pareeshani waghera hotee hai?

**Interviewee:** Mujhe pareshani waghera sirf khaaney peeney waghera ki aatee hai. Badpareezi waghera hojata hai, aur mein inko kehta hun kay istarah kay khaaney nahi banaya karu lekin yeh meri beemari ko beemari nahi samajhtee hain. Yeh samajhtee hain kay chalta hai

**Interviewer:** Theek hai. Aap ka din kaisa guzarta hai?

**Interviewee:** kaam kay baad zyada tar masjid mein hota hun aur Allah ko yaad karta hun. Uskay baat general knowledge ka shauq.

**Interviewer:** Koi routine waghera hai?

**Interviewee:** Nahi

**Interviewer:** Ghar mein zyada rehtay hain ya bahir?

**Interviewee:** depend karta hai. Karobar kuch aisa hai kay 10 baje nikalta hun tou aajata hun aur phr khaana waghera aakey khateey hun. Agar gaari hou tou hum zyada Karachi waghera ayein.

**Interviewer:** Aur aap ko lagta hai kay jo zeemedarian usually jo shauhar uthathay hain woh lee hain inki beemari ki waja say?

**Interviewee:** Haan. Boht zyada hee uthaye hain. *laughs* koi nahi uthatha itnee

**Interviewer:** saheeh aur apne farig waqt mein aap kya karte hain?

**Interviewee:** Buss *pause*

**Interviewer:** bêtey say dosti waghera hai?

**Interviewee:** Jee

**Interviewer:** acha aur doctor aap ko bataye hain kay inko OCD hai tou aap ko iskay barey mein maloomat hai?

**Interviewee:**  depression ki qism hai

**Interviewer:** aap ne kaha na kay aap ko general knowledge hasil karne ka shauq hai, kya kabhi aap ne jaakey parha hai?

**Interviewee:** Haan sunna hai kay depression ki eik qism hai. Usi tarah kee beemari hai, aur agar yeh khud chahye tou khatam hogee

**Interviewer:** aur waisay tou aap kay walid sahib ne kaha tha kay is shaadi ko baraqar rakhna, lekin koi aur zaati wajoohat hain?

**Interviewee:** Nahi kuch bhee nahi. Haan lekin meiney inko keh kay rakha hua hai kay agar achee larki milee tou mein shaadi karlunga dusri. Aur galaat kaam mein parne say behtar hai kay insaan jayez tareeqay say apni zarooriat puri kare. Inko mein kehta hun kyunke aurat ko uskay akhrajat chahye hain, Allah puray karwade tou koi aiteraaz nahi hona chahye hai.

**Interviewer:** Aur bête ke waja say aisa koi hai?

**Interviewee:** Nahi

**Interviewer:** Shaadi ko betey ki waja say tou barqarar nahi rakha hua?

**Interviewee:** Nahi ussay koi talooq nahi hai

**Interviewer:** theek hai aur aap ko lagta hai kay inki jo kaifiat hai inkay control mein hai?

**Interviewee:** asal mein ghar walay jo hain yeh samajhtey nahi thay aur agar samajh jaatey tou iska ilaaj karte. Yehi beemari hamari sister ko thee aur unko theek hogaye. Patient theek hojata hai. Lawazmat pura karne hote hai tou theek hojayega. Mein apni taraf say puri koshish karta hun lekin inki taraf say return nahi horaha hai.

**Interviewer:** aur aap ko kabhi kisi waqt aisa lagta hai kay inki koi galtee ki waja say inkay saath horahay hai?

**Interviewee:** Inki galtee nahi lekin ma baap ki galat parwarish. Mein ne aap ko kaha na. Kay maqsad mein karoor patee hun aur mein aap ko apna aap arab patee bataon. Jo yeh jhooti parwarish hotee hain, tou yeh larkyan waghera susral mein jaatee hain tou phr set nahi ho patee hain. Kyunke aap ko inko housewife bana kay nahi, aur maasi baana kay bhejtey hou kyunke itnay saarey logo kay kaam karne hote hain tou yeh dekh kay aurat pagal hojatee hai. Yeh system hai hamaray mashera mein

**Interviewer:** aap kay khayal mein shaadi shuda zindagi mein jo eik joray kay beech mein relationship jo hotee hai woh zyada ahmiat rakhtee hai ya pura khandaan?

**Interviewee:** Iskay andar tou aik hee cheez hai kay mian biwi ka mizaaj milna chahye hai. Woh hee kamyab hotee hai. Mijaaz matlab agar dunyawee shauhar ho tou dunyawee. Momin shauhar aur momin biwi

**Interviewer:** aur eik pursukoon khandaan kay liye kya zaroori cheezain hain?

**Interviewee:** shahoor. Aurat mein shahoor hona chahye hai, kay woh hur matter ko handle karsake.

**Interviewer:** Aur aap kay khayal mein aisee kya soretahal hone chahye hai kay insaan talaaq kay barey mein sochay?

**Interviewee:** Talaaq ka jo concept hai Islam mein woh eik taqleef cheez hain lekin shahoor waghera walay log hain hum log nahi karte. Hum Quran aur Hadith ko follow karte hain, Usmein talaaq hai hee nahi.

**Interviewer:** Kuch bhi hojaye?

**Interviewee:** Matlab hai, lekin uski koi sharaith hain. Kay yeh bilkul hee out hogaye hai ya kisi ghair aadmi kay saath iskay talooqat hain. Yeh choti choti shikateein tou hum khud hee dur karwadetey hain bol bol kay

**Interviewer:** acha buss eik akhri sawal. Kya aap ne marriage counseling kay barey mein sunna hai? Counselor jo hota hai kay agar mian biwi mein understanding nahi hotee tou madad karte hain aur thora sa bata detey hain kay aap inki baat samjhein aur aap inki baat samjhein? Aap ko lagta hai koi madad hasil hotee hai?

**Interviewee:** Nahi

**Interviewer:** acha kyun?

**Interviewee:** Iskay ghar walay unko agar achee parwarish karein tou yeh masla he na hou. Woh hota hai ghareeb ghar ka aur shaadi walay din qaroor patee ka make up kardetey hain.

**Interviewer:**

**Interviewee:**
