# Supplementary material for: Exploring effects of severe mental illnesses on marriages: A qualitative study from Karachi, Pakistan
Source: PLOS Glob Public Health. 2025 Dec 23;5(12):e0005652. doi: 10.1371/journal.pgph.0005652 (PMC12725543; doi:10.1371/journal.pgph.0005652)
Supplement: S1 Data — (ZIP) [file pgph.0005652.s001.zip › Transcriptions/Case 2-6 Transcripts/Case 4/C4-4.docx]

**Case 4**

He was initially hesitant in getting the interview recorded but after the assurance that his name would not be taken anywhere, he agreed for it to be recorded. The subject was recruited from the out-patient clinics (Dr. Hena).

Schizophrenia

**Interviewer:** How long have you been married for?

**Interviewee:** 1 saal.

**Interviewer:** aap ko shaadi kay kitne arsay baad inki beemari kay barey mein pata chala?

**Interviewee:** Shaadi kay tareeban 1 haftey baad mujhe pata chal gaya.

**Interviewer:** Tou shaadi say phele unko yeh beemari thi?

**Interviewee:** Ji inko thi lekin unhon ney mujhe bataya nahi tha.

**Interviewer:** tou yeh beemari kaafi serious hai ya bus thora boht …

**Interviewee:** serious kabhi kabhi hota hai

**Interviewer:** Acha, aur yeh dawayan kab sey le rahi hain?

**Interviewee:** Dawayan letey huay taqreeban 1 ½ month hua hai.

**Interviewer:** Issay pehle aap nay kisi aur ko dikhaya tha?

**Interviewee:** Nahi yeh log dikha rahay thay

**Interviewer:** Doctor ko dikha rahey thay ya rohani ilaaj tha?

**Interviewee:** Doctor ko hee. Pehel say elaaj chal raha tha.

**Interviewer:** Acha aap ko kisi qism ke mushkilat ka samna karna par raha hai?

**Interviewee:** Nahi bhai. Mein tou pura din job mein busy hota hun. Subah say raat tak. Sunday ko bhi mein Sunday bazaar mein apni shop lagata hun. Bus mayoosi hoti hai kabhi kabhar. Ubhi doctor ne kaha hai kay unkay ilaaj kay doraan unko apne pass rakhun. Insay phoochay kay meinney inko kisi qism ke koi shikait dei ho?

**Interviewer:** kya matlab, pass rakhun? Yeh kya kaheen aur reh rahi hain?

**Interviewee:** Inki walda kay pass rehti hain. 2 haftein merey pass raheen. Jis din inko injection lagna tha tou woh ghar say nikal gaye kisi ko baghair bataye. Yeh kisi bungalow mein lady councilor nahi hoteen area ki unkay pass phucnh gaye. Woh unko jantee bhee nahi thi. Tou unhon ney raat kay 9 baje ghar pe chora hai. Hamari memoni baradari haina. Mein job say bhagta hua aya aur unko leney gaya. Unhon ne phr ghar akey chora. Unhon ney baar baar phucha kay yeh hee aap ki wife hain. Boht tafseesh kay baadh merey hawalay kya. Mein chahta hun kay woh buss jald theek hojayein.

Boht dafa aisa bhee hota hai kay buss mein thak jata hun, aur mein kekhta hun buss …seedhi baat hai ..mein thak gaya hun aur mein chordun lekin nahi. Ubhi mein yahan say jaonga aur masjid mein jaonga aur istakhara karwaonga aur phuchoonga kay unpe koi asrat tou nahi hain. Agar woh kahaingay kay blkul clear hai tou mein yehi ilaaj karta rahun ga aur cooperate karunga.

**Interviewer:** Hmm, acha aap ko koi andaza hai kay aap ki wife ko shaadi kay kitnay arsay pheley tak beemari rehi thi?

**Interviewee:** Asal mein yeh meri shaadi dusri hai aur unki bhee dusri hai.

**Interviewer:** Acha acha.

**Interviewee:** Tou meri jo pheli shaadi huwi thi, divorce hogaye. Aur unki bhee divorce hogaye aur mujhe lagta hai kay uski bhee yehi waja thi. Unki iss halat ki waja say divorce huwi thi

**Interviewer:** Tou aap ko yeh baat pata thee kay iss waja say thee.

**Interviewee:** hmmm

**Interviewer:** Aap ne yeh socha tha kay unki divorce kiss waja say huwi thi?

**Interviewee:** Meiney socha kay hosakta hai shaadi kay baadh ghar ka mahul waghera change hota hai usswaja say waghera waghera. Adjustment issue hua hoga ya koi aur issue …saas bahu ka tou problem hota hee hai aaj kal. Lekin agar aap meri walda ko dekaingee tou aap kaheengi kay farishta hai. Unko duaon say hee mein agay chal raha hun.

**Interviewer:** Tou aisa hua kya tha kay aap ko pata chala kay beemari huwi hai?

**Interviewee:** shaadi kay eik haftay baad hee unko jhateky aaney lage. Haath pair bilkul akar jana ..istarah kay mamlat honay lagay. Tou meiney kaha kay koi problem hoga. Isski walda ney kaha kay yeh dawaiyan le rahi hain..shaadi kay 6 meheney baadh yeh baat samney aye kay yeh nafsiati masla hai.

**Interviewer:** Acha, tou aur kya problems thay? Unko awazein sunai deti hain aur..?

**Interviewee:** Jee yeh masla tha. Unko awazein sunayee deti hain. Agar aap unkay saath akeli beth jayein tou aap ko pata chaleyga kay woh bilkul khamoosh hee bethi huwi hongi. Jahan woh bhaag kay chali gaye theen na unhon ne bataya kay meiney unkay saath 2 ghantee sar khapaya lekin unho ney kuch nahi bula.

**Interviewer:** Aap ki walda aap key saath rehti hain? Aur unko inki beemari kay barey mein pata hai?

**Interviewee:** Ub pata chala hai

**Interviewer:** Acha, aap ne nahi bataya tha?

**Interviewee:** Nahi buss saath hee pata chala tha.

**Interviewer:** Acha, aap ko kisi qism kee madad milti hai, walda wagher say ?

**Interviewee:** Mein kisi kay kehne mein nahi aata

**Interviewer:** Nahi koi bhee qism ka support..

**Interviewee:**  mein kisi ka support nahi leta

**Interviewer:** Paiso kay hawalay say nahin.

**Interviewee:** Jee family ke support miljati hai. Istarah mujhe koi support nahi milta

**Interviewer:** Acha aur jab pershani hoti hai?

**Interviewee:** kisi ko bhee nahi batata

**Interviewer:** Acha aur agar aap pareshaan hotay hain?

**Interviewee:** Apne aap ko masroof rakh deta hun. Kaheen chaley jaonga.

**Interviewer:** Acha mujhe yeh batayein kay jab unki halaat aise hoti hai tou aap ko kaafi pareshani ka saamna karna parta hoga. Tou kitni pareeshani hoti hai?

**Interviewee:** Boht zyada. Meri job hai Mujhe job say bhag kar aana hota hai. Zahir hee see baat hai aaj kal ki jobs aisee hain kay seth tou bolangay kay chaley jao. Isstarah ki boht pareshaani hai. Bachi ko raat ko mein sambhalon.

**Interviewer:** Acha aur doctor kay pass aap hee lekey aatey hain?

**Interviewee:** Haan mein hee lekey aata hun.

**Interviewer:** Regularly aap hee lekey aatey hain? Unki walda waghera?

**Interviewee:** jee unki walda aur unkay bhai bhe aatey hain.

**Interviewer:** Lekin aap bhee saath aatey hain

**Interviewee:** Jee mein bhee saath hee aata hun.

**Interviewer:** Thakan waghera mahsoos hoti hai?

**Interviewee:** Jee

**Interviewer:** Aur aap rest kab kartay hain?

**Interviewee:** rest karta hee nahi huwi. Sukoon tou bilkul khatam hogaya hai. Mein ubhi bhii jis tareeqay sey betha hun meri qamr mein itnee takleef hai. Mera saara din kaam hai kharay rehna ka. Subah 9 baje say raat 9 baje tak. Sarey waqt kharey rehta hun. Bethney ka bilkul waqt nahi hota. Aur agar raat ko neend agaye tou 3 ya 4 baje aati hai and phr subah 8 baje uth jaana hojati hai

**Interviewer:** Acha aap ko lagta hai aap apni biwi kee madad kartay hain jab unki tabiat kharab hoti hai?

**Interviewee:** Jee

**Interviewer:** Acha aur aap ko lagta hai aap ka support unko madad karta hai?

**Interviewee:** Karta hai…

**Interviewer:** Acha aur aap dunu ka rishta kaisa hai? Aap log baat cheet waghera kartey hain?

**Interviewee:** Depend karta hai. Kabhi unhon ne baat karli tou karli warna nahi karengee. Woh awaaz hee nahi sunegeen.

**Interviewer:** Hmm matlab aisa nahi hai kay aap bahir gaye aur aap ne enjoy waghera kya, aur bahir gaye..bahir aana jaana hota hai dosto waghera mein

**Interviewee:** Nahi hota bilkul bhi

**Interviewer:** aur khandaan waghera mein?

**Interviewee:** nahi agar shaadi waghera hogaye tou theek hai warna waise nahi

**Interviewer:** Aur aap say log inki beemari kay barey mein phoochtay hain kay unko kya hai?

**Interviewee:** Nahin. Ghar mein kisi ko nahi pata

**Interviewer:** Acha agar farz karein koi phoochay tou aap kya jawab deinge?

**Interviewee:** Mein tou yahi kahoonga kay yeh behtar hain. Aur dua karsaktey hain..iskay ilawa kuch nahi

**Interviewer:** Yeh isliye kyunk log ajeeb ajeeb batein kartay hain? Humaray mahushira mein yeh hota haina?

*interruption in the interview*

**Interviewee:** Waise tou unlogo yeh mujhe batadena chahye tha kay yeh aisay mamlaat hain. Takey pareshaani na ho jaise kay mujhe horahi ho woh kal kisi kay saath na ho. Iss sey behtar hai kay batadena chahye kay unki life bhee barbaad na ho.

**Interviewer:** Aap ko aisa mahsoos hota hai kay dhoka diya gaya hai? Kahbhi ghussa nahi aata kay nahi bataya?

**Interviewee:** Mein tou buss yeh samajhta hun kay meri qismat mein yeh tha.

**Interviewer:** Hmm acha aur agar aap ko pata hota aur agar aap ko batadiya hota tou aap ko lagta kay aap unsay shaadi kartey..matlab generally?

**Interviewee:** Nahi mein tou nahi karta.. seedhi see baat hai. Hmm kyunke life barbaad hojati hai…

**Interviewer:** Jab aap ko pata chala beemari kay barey mein tou aap ka kya radeamal tha? Kya zehn mein aya?

**Interviewee:** Merey zehn mein aya kay mein cooperate karun aur jakey ilaaj karaun khud jakey. Tou mein karwa rahi hun. Takey issay unko bhee thori support milege kay merey saath bhee koi hai. Warna mein unko agar unki walda kay pass chordunga tou yeh samjhein geen kay merey saath koi nahi hai. Takey usko support miley.

**Interviewer:** Acha is beemari ki waja say aap kay khandaan kay mahul mein kya farq aya hai?

**Interviewee:** Meri family say meri baat cheet…seedhi see baat hai agar koi baat hoti hai tou mein kisi say baat nahi karta..mein apne bhen bhayon say kat chukka hun. Unsay meri narazgi chal rahi hai

**Interviewer:** Pheley say chal rahi hai ya ubhi huwi hai?

**Interviewee:** Iss cheeez kee waja say..meray yahan aisa tha kay mein agar raat kay time bhee kuch hota tha tou walda ko saath lekey chala jaata. Tou mein nikal jata tha lekin ub nahi.

**Interviewer:** Issliye kay aap pareshani zahir nahi karna chahtey kay..?

**Interviewee:** Pareshani zahir nahi karna chahta.

**Interviewer:** Aap kay bhen bhayo ko pata hai kay unki beemari kay barey mein?

**Interviewee:** Haan unko pata hai.

**Interviewer:** Theek hai. Phr unka kya radeamal tha?

**Interviewee:**  unhon ne kaha kay bhai saari zemedari aap kay uper hai…aap kya chahtey hain? Ubh kya karna chahtey hain..but mera dil nahi hai chorney ka. Mujhe umeed hai kay yeh behtar hojayein.

**Interviewer:** Acha aur aap ka jo bacha hai..woh 1 saal say kam ka hai. Woh khayal rakh leti hain?

**Interviewee:** nahi. Bilkul bhee nahi. Unhon ne yeh bhee doctor ko kaha kay mujhe awazein sunaye deh rahi hain kay bachay ko mardein. Doctor ne kaha hai kay bachey kee zeemadari koi aur uthaye. Mujhe bhee nahi lagta kay family mein mera bacha mahfooz hai. Inki family mein walid sahib hotay hain, walda hoti hai, bhen hoti hain..kabhee woh kisi waqt bhee kuch karlein..pata thori chaleyga…

**Interviewer:** Hmm, acha aur aap inko regularly appointments pe lekey aatey hain?

**Interviewee:** jee

**Interviewer:** Aur aap ko kya lagta hai kay jo shaadi kay pheley haftay unki tabiat theek rahi the tou uss waqt kay baadh say aap ka rishta kis tareeqay say badla hai?

**Interviewee:** *pause* yeh du haftay raheen merey pass tou samjhay hur cheez mein meiney unka khayal rakha hai..ghumaya phiraya hai. Khilaya pilaya hai. 3 chutyan jo moharram ki ayeen theen ussmein mein ney unko ghummaya hai. Sab kuch kiya hai lekin phr bhee waisa hee hai.

**Interviewer:** Hmm acha aap bata rahey thay kay aap ki bhai bhen say ub nahi banti. Tou usske waja say aap kay dosray rishto pe farq par raha hai?

**Interviewee:** Haan

**Interviewer:** Kisi ne aap ko talaaq ka mashwara diya?

**Interviewee:** Nahi

**Interviewer:** Kabhi waldeen ne bola ho?

**Interviewee:** Nahi.

**Interviewer:** Acha aur unki fees doctor kee aap ko hee deni parti hogi?

**Interviewee:** Nahi inkay waldeen detey hain. Mein nahi. Mujh say le hee nahi rahay. Khud hee aatey hain.

**Interviewer:** Acha, aap ko lagta hai kay inki nafsiati beemari ki waja say aap stress mein agaye hain ya aap ko koi nafsiati problem hogaye hai?

**Interviewee:** Nahi.

**Interviewer:** Ghussa waghera?

**Interviewee:** Ghussa aata hai.. lekin pee jata hun. Woh zyada behtar hai. Yaqeen karein ussdin tou itna bara phadda hogaya tha meineny sheesha tor diya hai..meiney kaha kay usko lejao.. Lekin kuch dair kay baad mera ghussa thanda hua tou dubara wapis bulwa liya.

**Interviewer:** Kahbhi aisa hua hai kay inhon nay ghussay mein haath uthaya ho ya cheez mari ho?

**Interviewee:** unhon ney meri walda ko boht kuch bola tha aur phr mera haath uth gaya tha..seedhi baat hai mera haath uth gaya tha. Lekin phr mein khamoosh hogaya tha.

**Interviewer:** aap ko kya lagta hai kay aisee konsi zimdarian aap ne uthaye hain jo aap ki wife ki honi chahyein hain?

**Interviewee:** sari zeemedari meri hee hai. Bachay kee parwarish bhee mein kar raha hun. Bachay ko sambhal bee mein raha hun. Aur bachay ko feed aur khila bhee mein hee raha hun… yeh taqreeban na leney kay barabaar hain.

**Interviewer:** Aur acha aap subha 8 baje office..dukaan chaley jatey hain… aur phrr atey kab hain?

**Interviewee:** 9 baje

**Interviewer:** phr aap dekhtay hain bachay ko?

**Interviewee:** Haan merey pass aajkal tou hai nahi. Unki walda kay pass hai.

**Interviewer:** theek. Unki beemari ki waja say aap ne kya extra zeemadariyan lee huwi hain? Kahna pakana waghera?

**Interviewee:**  Woh meri walda hee kar rahi hain.

**Interviewer:** Aur aap apney farig waqt mein kya kartey hain?

**Interviewee:** Milta hee nahi hai

**Interviewer:** Acha aur kya shauq hai karney ka?

**Interviewee:** Ub tou kuch bhe nahi hai. Yeh saheen hojayein woh hi boht hai

**Interviewer:** Agar aap mind na karein tou mein yeh phooch sakteen hun kay aap ki peheli divorce kiss waja say huwi thi?

**Interviewee:** Meri divorce…meri mohabbat ki shaadi thi. Woh hamari baradari ki nahi theen. Baradari say bahir ki theen.

**Interviewer:** Yeh baradaari ki hain?

**Interviewee:** jee baradari kee hain. Woh baradari say bahir ki theen. Divorce iss liye huwi thee kay unkay aisay demands theeen kay mein du haftey mein apni walda key saath rahun..apne bhen kay bachay bhee mein apne ghar le aon aur unko rakhun bhee mein hee aur sambhalon bhee mein hee…istarah ki mamlaat ke waja say hamari divorce huwi.

**Interviewer:** Acha aap ko lagta hai kay aap ko iss beemari kay barey mein enough pata hai? Matlab aap ko kya maloomat hain aur doctor ne kya bataya hai?

**Interviewee:** Mein iss beemari kay barey mein zyada nahi janta.

**Interviewer:** Aap ne kabhe suna hai nafsiat kee beemari kay barey mein?

**Interviewee:** Nahi.. meiney aaj tak kisi ko isstarah kee harqateein kartey nahi dekha.

**Interviewer:** Inki phupo jo theen woh bhee aap ki baradari kee hungi phr. Tou unmein yeh nahi dekha tha?

**Interviewee:** Nahi meiney kaha na kay mein innki family mein sirf chand logo ko janta hun warna mein kisi ko janta hee nahi ho. Meiney suna hai kay inki phupion waghera mein nafsiat thee.

**Interviewer:** Acha, aap ko shauq hai iss beemari kay barey mein aur pata karney ka?

**Interviewee:** Mein tou kehta hun kay jissko bhi ho..Allah Pak usko khatam karde.

**Interviewer:** Acha, aisee konsi zaati wajoohat hain jiske waja say aap ney shaadi ko barqarar rakha hua hai even though inhon ne aap say chupaya waghera?

**Interviewee:** Buss eik hamdardi hai…

**Interviewer:** hmm aap ko yeh hota hai kay agar aap chordaingay tou yeh kahan jayeinge?

**Interviewee:** Nahi… masla yeh hai kay agar mein chor bhee deta hun inko tou bachay ki problem hai. Mein isstarah sochta hun kay yeh meri bachay ki maa hain. Mein sochta hun kay eik ma bachey say juda ho. Hmm bacha tou chalo meri pass ajayega aur meri walda sambhal leingee aur sub kuch karleinge. Lekin yeh tou juda hojayeinge na..kal ko mera bacha bara hoga aur phoochay ga kay meri ma kahan hai. Aur log tou aaj kal waise bhee batein bananey mein mahir hai..bachpan say hee uskay kaan mein bharney lageinge kay tumhari ma aisee theen.. mein nahi chahta kay aisa ho.

**Interviewer:** aap ko lagta hai kay aap ki biwi ko koi galti thee jis ki waja say unko yeh beemari huwi hai? Kabee aisa feel hua hai aisa aap ko?

**Interviewee:** Kabhee nahi

**Interviewer:** Aur aap ko kya lagta hai aap inko kisi tareekay say bhee inko sahi karsaktey hain?

**Interviewee:** Support tou meri puri hai lekin baqi Allah pak hee karsaktein hain

**Interviewer:** Aap keh rahay thay kay aap ghussay mein inko chornay ka sochtay hain aap ne kabhi talaaq kay barey mein socha hai?

**Interviewee:** Haan socha hai. Boht baar socha hai. Lekin phr dil nahi manta. Ghussa mein akey.

**Interviewer:** dost waghera ya family ne bola kay dedo?

**Interviewee:** Hmm no

**Interviewer:** Acha aap kay khayal mein two logo ko kab talaaq leni chahye hai? Aisee kya soritahaal ho kay buss …?

**Interviewee:** Talaaq aisee sorithaal mein deni chahye agar aurat ki koi galti ho. Tou talaaq dedein. Zahir hee see baat hai galtyan aadmi mein bhee hoti hain aur aurto mein bhee… dunu apne jaga barabaar hotay hain..

**Interviewer:** Sahi.. aur aap ko lagta hai agar aap ko government support miley ya family say support miley …family say tou milta hai..,tou aap ka burden kam hota hai?

**Interviewee:** Sab kuch meri walda sab kartee hain…haan tou support hai

**Interviewer:** Acha aur aap ko kya lagta hai kay eik shaadi shoda joray kay beech mein rishta ya family zyada ahm hota hai?

**Interviewee:** Rishta zyada ahm hota hai. Kyunke woh kisi aur mahul say aati hain. Unko zyada support milna chahye.

**Interviewer:** Aap ko kya lagta hai kay eik pursukoon khandaan kay liye kya cheezain zaroori honi chahyein?

**Interviewee:** Khush…. Khushali …mil jul kay bethna ..batein karna …ghoomna phirnaa. Bahir enjoy karney jaana .. istarah ki khushali…

**Interviewer:** Marital counseling kay barey mein suna hai aap ne?

**Interviewee:** Hmm nahi

**Interviewer:** Matlab yeh kay agar mian biwi kay beech mein boht misunderstanding horahi ho tou zyada tar nafsiati mein … aap keh rahay thay na woh baat nahi sunteen tou kuch log counselor hotay hain shaadi kay ..tou aap ko kya lagta hai kay aap ko madad mil saktee hai..jab koi tisra (3^rd^) party jaisey kay doctor thori help karey

**Interviewee:** nahi

**Interviewer:** Theek hai. Aur kuch aur add karna chahye gain?

**Interviewee:** Nahi…

**Interviewer:** Acha thank you. And sorry hum ne aap ka itna time liya.

***Interview Ends***
