# Supplementary material for: Exploring effects of severe mental illnesses on marriages: A qualitative study from Karachi, Pakistan
Source: PLOS Glob Public Health. 2025 Dec 23;5(12):e0005652. doi: 10.1371/journal.pgph.0005652 (PMC12725543; doi:10.1371/journal.pgph.0005652)
Supplement: S1 Data — (ZIP) [file pgph.0005652.s001.zip › Transcriptions/Case 2-6 Transcripts/Case 4/C4-5.docx]

**Case 4**

**Psychiatric Disorder:** Schizophrenia

**In-patient**

*fills out the consent form and the demographic form* (age 33 years old, has done his MBA, and he is currently working since 5-6 years. Has been married since ___. Lives in joint family set-up. There are 7 people who live in the household and there are three earning members in the family. Has an average income of Rs. 2 lakh per month, and they have one daughter, and his wife is an alima, and has done her graduate. His father is a Double M.A. He does not have a psychiatric history himself but he mentions that his wife’s family does have a psychiatric history, including her uncle and cousins. And she has had this illness since 2011. So the problem was there before marriage but he did not know. Mentions that she has a psychiatric disorder. After the wedding, she used to take her medications hiding it from him and her condition got worsened. All the symptoms came out. She also tried to kill her child. There is no history of addiction and there are no current financial problems. He does not report any health issues. There are marital issues in the marriage. Mentions that his family is extremely cooperative.)

**Interviewer:** acha jab aap ko inki beemari kay barey mein pata challa tou aap inko doctor kay pass lekey gaye thay?

**Interviewee:** Jee mein inko neurologist kay pass lekey gaya tha. Yeh inkay father waghera say baat huwi tou unhon ne kahan kay dimag mein chemicals kam hojate hain. Dr. Rizwan say yeh ilaj kar raha theen aur medicine waghera lete theen.

**Interviewer:** acha aap ko beemari ka shaadi key kitne arsay baad pata challa?

**Interviewee:** Jab inki pregnancy chal rahee thee aur jab unki tabiat kharab hone lagee. Yeh bolna shuru kardete hain aur phr inhon ne eik raat khud bataya kay mujhe yeh mental problem hai. Mera ilaaj chal raha hai, aur phr mein ney inki family say information gather ki aur phr mein Dr. Rizwan kay sath khud bhi milne gaya. Lekin unhon ne file nahi dikhaye

**Interviewer:** Dr. Murad kay pass aap lekey aye?

**Interviewee:** Jee. Mein lekey aye.

**Interviewer:** Theek hai aur yeh pheli dafa hospitalize huwi hain?

**Interviewee:** Jee phehli martaba huwi hain. Issay pheley pregnancy kay waqt huwi theen.

**Interviewer:** Theek hai aur aap ne apne parents ko foran batadiya tha inki beemari kay barey mein?

**Interviewee:** Nahi meiney foran nahi bataya. Mein khud hee face karta raha. Eik saal thak nahi bataya lekin jab beti paida huwi tou …samajh tou gaye thay, itnay saalon ka experience hai. Saari life ka aur uskay baad meiney unko saari cheezain batadeen

**Interviewer:** Kya aap logo ke arranged marriage hai?

**Interviewee:**  Jee arranged marriage hai

**Interviewer:** Shaadi say pheley aap log baat waghera karte thay?

**Interviewee:** Haan kabhe kabhar, sometimes regularly aisee koi baat nahi hotee thee.

**Interviewer:** Tu ussmein unhon ne kabhi nahi bataya?

**Interviewee:** Nahi. Buss kabhi kabhar baat hotee thee

**Interviewer:** aur aap ko help miltee hai, financially nahi, but emotionally waghera, kay aap ko support kar rahay hain, family walay inko manage karne walay?

**Interviewee:** Yeh case prolong hogaya hai tou eik ego ki baat thee, kay yeh patient hai aur hamari family kay liye hard time diya. Lekin meiney realize kya kay yeh beemari hai aur Allah ki taraf say. Tou meiney chaha kay proper elaaj hojaye. And there was resistance from her family but kuch barey logo ne family mein samjhaya. Phr yeh admit huween

**Interviewer:** Aur aap ko iss beemari ki waja say pareeshani ka saamna karna parta hai?

**Interviewee:** Haan definitely

**Interviewer:** Kistarah kee pareeshani ka saamna karna parta hai?

**Interviewee:**  24/7 care karni partee hai aur jab shaadi hotee hai tou mian biwi gari kay du paye hotey hain aur hur tarah kay halaat ko saath face karna hota hai, tou 1 ½ saal meiney inko bilkul just like a child rakha. Inka brain bilkul eik child kee tarah tha. Koi baat nahi samajhtee aur eik biwi wali baat nahi thee. Tou boht zyada cheezain face karnee partee hain aur dukh hain pareeshai hai, lekin aap share nahi karsaktay aur phr koi cheez samjhna nahi aur hur cheez mein apni marzi karna.

**Interviewer:** aur bachay kay woh khayal rakh paate hain?

**Interviewee:** Han bachay ka khayal rakh letee hain. Proper rakhteen hain. Qudrati mothers hee zyada kartee hain aur kabhi kabhar lekin yeh out of control hojatee hain kyunke sense khatam hojata hai aur eik du dafa bachi ko bhi phainka hai. Eik haath say pakar kar uthana aur uper lejena

**Interviewer:** acha aur aap ko sab say zyada kon see cheez boht zyada mushkil mein daaltee hai? Doctor kay pass lekey aana ya inko manage karna?

**Interviewee:** Inko manage karna hai aur phr job ko manage karna kyun apna tou hai nahi, job hee hai. Kabhi kabhar late bhi hojata hun lekin yeh log cooperate kartay hain aur aksar unko agar zid aajatee hai tou phr mein inko no nahi keh sakta. Jaisay agar kabhi inko bahir jaana hai tou bahir hee jaate hain. Yeh saari cheezain karne partee hain aur phr beti ki kaafi fikar rehte hai

**Interviewer:** acha aur ghar mein aap kay ami abbu khayal rakh letey hain?

**Interviewee:** haan

**Interviewer:** acha aur aap logo kay family aur doston me aana jaana hota hai?

**Interviewee:** family mein hota hai, but doston mein itna nahi hota. Merey itnay dost waghera nahi hain

**Interviewer:** acha khandaan mein logo ko inki beemari kay barey mein maloom hai? Kya kehte hain?

**Interviewee:** asal mein jo hai inki taraf kay jo log hain woh zyada tar illiteracy mein hain. Mujhe shaadi kay baad yeh saari baatein pata chali hain, yeh log dam aur pani wgahera pe..aap ko pata haina. Non-professional log hain, theek hai, Allah kehta hai Quran mein hain

**Interviewer:** acha yeh peer alim waghera kay pass gaye hain?

**Interviewee:** Jee kaafi dafa gaye hain, saari cheezain raheen hain. Illiterate hain waghera rishteedar, aur phr yeh kehtey hain pir ki taraf lejao waghera

**Interviewer:** Acha agar aap ko inki beemari kay barey mein maloom hota tou kya aap unsay shaadi karte?

**Interviewee:** Nahi

**Interviewer:** acha aur jab aap ko inki beemari kay barey mein phehli dafa maloom hua tou aap ka kya radeamal tha?

**Interviewee:** inki taraf say mein..mera first experience tha istarah kee cheez say tu mera kaafi sudden reaction tha. Tu mujhe der saal laga aisee cheezain karne mein aur samajhne mein bhi kaafi time laga. Yeh mental disorder hai lekin inkay walid sahib itnay saal say yeh realize nahi kar rahay thay jiski waja say yeh naubat aye hai

**Interviewer:** Dam wagera ley jaatey thay ?

**Interviewee:**  Aur sirf eik doctor say consult kartey thay aur woh psychiatrist nahi thay aur phr jab mein doctor kay pass lekey jaata tha tou pheechay say phone kartey thay kay iss doctor kay pass lekey jao, aur isskay pass nahi lekey jao. Aur phr inkay dimag mein eik cheez beth jaate hai tou phr yeh aggressive hojatee hain. Tou yeh situation thee

**Interviewer:** acha aap ko lagta hai kay inki beemari ki waja say aap kay ghar kay mahol mein koi farq para hai?

**Interviewee:** beinteha. Puray ghar ka atmosphere hee kharab hogaya hai, merey chotay bhai ki bhi shaadi huwi hai. Unki wife ko yeh bilkul bardasht nahi karpatee hain aur hum ne aisay guzara kya hai.

**Interviewer:** Acha aur isski waja say aap kay bhai aur aap key beech mein koi maslay masail?

**Interviewee:** Nahi Allah ka shukar, aisa koi issue nahi hua

**Interviewer:** acha aur aap kay dusre family members ka iss beemari ko letey huay kya radeamal tha?

**Interviewee:** dekhain opinion hur koi deta hai. Mujhe iss ki condition pata hai aur agar mein koi deicison leta hun tu uski mazeed tabiat kharab hojayege. Yahnee kay yeh bilkul hee aur mein nahi chahta kay mein koi decision lun, insaanyat kay naatey ya hamdardi ki waja say kay isski waja say isski aur tabiat kharab hojaye

**Interviewer:** Kaisa decision matlab?

**Interviewee:** Dekhain Islam mein boht tarah kay options diye huay hain, yahnee aap ki bhi life hai, aur aap samajh rahi hongee kay mein kya keh raha hun. Boht saarey options hain jo kay banda lesakta hai. Lekin mein yeh nahi chahta aur na meiney socha hai issbarey mein. Meri aulaad hai aur ma jaisee bhi ho isski tou ma haina. Koi aur tu nahi khayal rakheyga

**Interviewer:** Acha aur aap ko kya lagta hai kay jab say aap ko inki beemari kay barey mein maloom hua hai tou aap dunu ki relationship change huwi hai kisi tarah say?

**Interviewee:**  relationship tou establish hee nahi huwi hai kyunke common sense hee yeh chor bethee hain. Yeh tou biljul istarah ki position mein hain hee nahi kay yeh understand karsakein kay kon baap hai aur kon shauhar hai, kon bhai hai

**Interviewer:** aur isski waja say aap kay dusrey rishto pe farq para hai?

**Interviewee:** Nahi farq nahi parta

**Interviewer:** Aap inko doctor kay pass lekey jaatey hain?

**Interviewee:** Jee pheley Dr. hussain kay pass aur ubh yahan. Lekin sometimes yeh hota tha kay inka appointment mila Tuesday ya Wednesday tou mein nahi ja pata tha tu merey walid sahib lejatey thay, aur mujhe office waghera manage karna parta hai

**Interviewer:** Acha aur aap ko inki beemari ki waja say koi zehni dubao waghera mahsoos hota hai?

**Interviewee:** Jee jee

**Interviewer:** Koi depression waghera horaha hai?

**Interviewee:** Jee boht zyada depression hota hai aur tension bhi hotee hai. Lekin theek hai yeh karna hai

**Interviewer:** Acha aap ka din kaisa guzarta hai aur aap subah sey raat tak kya karte hain aur kitna khayal rakhe hain aur inko kitna time detey hain?

**Interviewee:** Mein inki kisi baat ko na nahi kehta, yeh ko kehtee hain mein karta hun. 1 ½ saal mein meiney yehi kaha. Log yeh bhi kehtey hain kay kyun kar rahay ho jo condition nahi jaantey thay, jo log nahi samajhtey kay yeh beemari hai, tu ustarah ki cheezain bhi face kee hain. Lekin mujhe pata tha kay yeh beemari hai

**Interviewer:** acha tou foran hee pata chal gaya tha? Aap ne bataya tha kay jab yeh pregnant thi tou uswaqt?

**Interviewee:** Jab shaadi huwi hai, tou mein samajhta tha kay premature shaadi hogaye hai, inki jaldi shaadi hogaye hai. Mein yehi samajhta lekin jab yeh pregnancy huwi tou phr khatarnak situation hogayee. Aur jitney doctors say raabta kya tou medications nahi detey thay pregnancy ki waja say. Tou humein wait karna para tha. Tu mein ghoomana waghera lejata tha, lekin yeh cheezain painktee hain. Inkay ghar walon ne kaha pregnancy ki waja say horaha hai tou unkay dimag mein yeh beth gaya tou yeh baby ko marne ki koshish karne lageen aur kehtee theen kay abortion karwaleti hun aur mujhe pata challa. 7-8 kism kay symptoms huay tou mein yehi samjha kay yeh symptoms hain. Phr yeh saheeh hogayee jab dawaiyan shuru huwi

**Interviewer:** Aap ko lagta hai kay inki beemari ki waja say jo inko zeemedarian leni chahye hain woh aap ne lelein hain?

**Interviewee:** Bilkul. Mein eik baap ka bhi role ada kar raha hun aur eik ma ka bhi.

**Interviewer:** Saheeh. Tou aap baby ka bhi khayal kartay hain?

**Interviewee:** haan lekin ubhi tou baby inki walda kay pass hai

**Interviewer:** Aap apne farig mein kya karte hai?

**Interviewee:** Koi game waghera khel leta hun

**Interviewer:** Acha aur kitna leisure time milta hai?

**Interviewee:** 1-2 ghanta

**Interviewer:** hur roz?

**Interviewee:** Sunday walay din

**Interviewer:** Acha aap ko lagta hai kay aap ko inki beemari kay barey mein eik had tak maloom hai? Aap ne internet waghera pe search kya hai?

**Interviewee:** yeh beemari jo hai, mujhe maloom tou hogaya tha jab symptoms waghera hogaye thay. Tou mujhe ubh kaafi had tak pata chal gaya hai

**Interviewer:** aap ne doctors waghera say consult kya?

**Interviewee:**  doctors waghera say bhi kya, aur phr common sense bhi keh raha hota hai. Merey zehen mein yeh doubt bhi tha kyunke inkay khandaan mein istarah kay patients thay jo ajeeb harkatay kartay thay. And phr unka tumor diagnose hua tha tou shayad iski waja say harqatay kartay thay istarah ki. Ganday kapray phen letey thay aur paiso ki barbaadi shuru kardee. Yeh saari cheezain waghera shuru hojateen theen. Yeh mujhe doubt tha aur ubhi tak yeh doubt hai.

*interruption in the interview*

**Interviewer:** acha aap ko kya lagta hai kay aisee kya zaati wajoohat hain jiski waja say aap ne iss shaadi ko barqarar rakha hua hai?

**Interviewee:** Jo wajoohat hain kay yeh meri baby hain aur agar merey saath bura hua hai tou mein apnee aulaad kay saath tou bura nahi karsakta aur phr mein yeh bhee dekhta hun kay inki condition mazeed kharab nahi hojaye, agar meiney decision istarah ka koi liya. Agar 10 rupay bhi gir jayein tou inkay dimag mein tension rehtee hai aur yeh handle nahi karsakteen. Aur agar mein koi decision lunga tou yeh mukamal tor per. Mein insaaniyat kay naatey apni zindagi istarah guzarnay pe tyaar hun. Aur phr Allah ne baby bhi dedi hai aur job hi hai ma eik ma hoti hai aur baap baap hota hai aur istarah koi kisi ki jaga nahi lesakta. Chahye woh mentally disturbed hain, lekin hain ma

**Interviewer:** acha aur aap ko lagta hai kay aap ki biwi ki koi galtee waghera hai jiski waja say inko yeh beemari hai?

**Interviewee:** Nahi aisa tou kuch nahi lekin inhon ne aalma ka course kya tha tou inkay uper burden boht tha. Phr inki shayad walid sahib ka attitude..boht sarey log kehtey hain kay iski waja yeh bhi hai. Hadd say zyada extra burden and zahir hee see baat hai aalim bana koi asaan baat tou nahi hai aur phr inki mujh say magnee bhi huwi thee aur phr toot gaye thee aur log kehtey hain kay magnee tootnay ki waja say inhon ne zyada sadma leliya. Jab tak dimag mein pheley say beemari thee, yeh chronic hoti hai, kisi bhi aisay situation mein zyada bigar jaati hai tabiat jokay insaan zyada bardasht nahi karpata hai. Aur pheley sey hoti hai lekin phr zyada bhar jatee hain aur baqol inkay inka ilaaj 3-4 meheney chalta raha. Medicine khayein. Aur inhon ne meri cousin say contact rakha hua tha, magnee tootnay kay baad bhi. Aur phr dubara say rishta daalne mein meri cousin ka hee haath tha.

**Interviewer:** Phr wapis say mgnee huwi?

**Interviewee:** Jee

**Interviewer:** Acha aap ko lagta hai kay aap inko theek karsaktey hain?

**Interviewee:**  Alhumdillah, mein koshish tou karsakta hun lekin mein saheeh tou nahi karsakta kyunke saheeh karnay wali zaat tou Allah ki hai. Zahir hee see baat hai, aur phr mein koshish karsakta hun, aur mein 1 ½ saal say saari koshish kar raha hun

**Interviewer:** acha aap ko kisi ne elaidgi ya talaaq ka kisi ne mashwara diya?

**Interviewee:** Boht saarey logo ne diya kyunke situation aisee hogaya hai ubh kay yeh boht common see baat hogaye hai. Koi fashion bana diya tha, aur phr boht sarey logo ney mashwara diya, kyunke yeh boht bari qurbaani hai, lekin saari cheezon ko madenazar rakhte huay, decision akhir mera hee hai

**Interviewer:** acha lekin kabhi aap ne socha?

**Interviewee:** Insaan sochta tou boht kuch hai, lekin hota tou wohi hai jo Allah ko manzoor hota hai. Kyunke eik patient hai, as a patient hee lene chahye hai, aur mein patient ki tarah hee leta hun

**Interviewer:** acha aap ne kaha kay aksar yeh kaafi ghussay mein aajatee hain aur cheezain utha kay phainktee hain, kabhi ghussay mein aap ko mara waghera?

**Interviewee:** Nahi, marna waghera nahi hai, iss had tak nahi

**Interviewer:** Kabhi aap ne unpe haath uthaya?

**Interviewee:** Nahi kabhi bhee nahi, Alhumdillilah. Tashadud nahi kya, lekin jab aisee haalat phele dafa huwi thee tou inkay walid sahib ne unko bandh kay rakha hua tha. Kyunke inki zabaan jo hai jab chalna shuru hotee hai tou control mein nahi rehtee aur abusive language shuru hojatee hai. Tou normal patient nahi lagteen aur phr abusive language ko control karne kay liye bandh diya, baqool unkay lekin meiney sunna hai kay inko maara bhi tha inkay walid sahib ney. Walid sahib ko bardassht nahi kartee. Inka koi bhi family membe jayega tou yeh shor sharaba shuru kardetee hain. Zaleel karna shuru kardetee hain

**Interviewer:** Aap ko kya lagta hai aisee kya soretahal ho jissmein eik couple soch sakta hai kay hum elaidgi ikhtiar karlete hain?

**Interviewee:**  Aisee nahi hona chhaye, yeh hosakta hai kay yeh apni jaga hain, apne ghar mein rehen, lekin divorce mein kabhi nahi desakta

**Interviewer:** lekin aap kay illawa, generally koi aisee situation ho?

**Interviewee:** Situation pe depend karta hai, wife nahi rehna chah raheen ya had say zyada aisa ho kay bilkul naukar bana liya ho. Meiney itna kabhi socha nahi iss topic kay barey mein

**Interviewer:** aap ko kya lagta hai kay eik shaadi shuda joray key beech mein jo relationship hoti hai woh zyada important hoti hai ya family zyada important hotee hai?

**Interviewee:** Jo relationship zyada important hoti hai woh husband aur wife key beech mein jo hotee hai. Family hai lekin theek hain, wife ke jaga family nahi lesaktee aur family ki jaga wife nahi lesaktee, zahir he see baat hai. Aisa relationship boht important hai lekin aisa relationship ban nahi paya 1 ½ saal mein.

**Interviewer:** aap ko kya lagta hai kay eik pur sukoon khandaan ki parwarish kay liye kya cheezain zaroori hain?

**Interviewee:** sirf inko tension nahi de jaye, pura sukoon rehna chhaye hain, aur agar financial problems hon tou bacho kay saamne izhaar nahi karna chahye hai, achee say achee taleem de jaye, waldeen maturity ka muzahira karein aur depression walay mahol mein, protect karna chahye, bacha depressed na rahay, bachay complex ka bhi shikar hojatay hain, waldeen ko yeh cheezain lekey chalna chahye, aur bacho pe asr nahi hona chahye. Tou yeh situation jo kharab huwi hai, inho ne yeh saari cheezain apnee family mein dekhi hai. Jab yeh hyper hotee hain tou previous baatein apne walid sahib ki batatee hain, paisay lekey bhag gjayein, istarah kee cheezain. Repeat karne shuru kardetey hun

**Interviewer:** Acha merey sawal complete hogaye hain, aap ko koi sawal karna hai?

**Interviewee:** *asks about the mental illness in specific*

***Interview Ends***
