# Supplementary material for: Exploring effects of severe mental illnesses on marriages: A qualitative study from Karachi, Pakistan
Source: PLOS Glob Public Health. 2025 Dec 23;5(12):e0005652. doi: 10.1371/journal.pgph.0005652 (PMC12725543; doi:10.1371/journal.pgph.0005652)
Supplement: S1 Data — (ZIP) [file pgph.0005652.s001.zip › Transcriptions/Case 2-6 Transcripts/Case 4/C4-6.docx]

**Case 4**

**Psychiatric Illness: Bipolar Disorder**

The wife did not provide her name since she works at AKU so she was a bit hesitant. She also did not allow the interview to be recorded.

The couple has been married for 11 years. The illness has been present since 1996. However, they got married in 2004. They had a love marriage, or marriage of choice, and they had known each other before getting married. However, her husband did not tell her about his condition before getting married.

She got to know about the illness in 2006 when her first daughter was born. She mentioned that she did not know about the illness, hence she did not know how to handle it which meant that she was very stressed during her pregnancy and took out her stress on him, which may have triggered the episode.

Her parents don’t know about the illness primarily because she didn’t want them to know. *Zarurat mahsoos nahi huwi.* She feels that they do not understand the concept of mental illness so she does not want to explain it to them. His parents of course know about the illness and have provided excellent support to her, financial and emotional. Her husband is unable to keep a job for too long because he gets these phases where he is quite inactive. Therefore, in that case, her sister in-law who is married and lives in America often sends money. They are also able to tell her how to manage the illness, therefore she has excellent support.

Her parents however do have an idea that there is something wrong because he often leaves jobs so they ask her as to why that happens. However, she has always hidden the illness.

She also mentions that her mother in-law is quite ill and is in America currently but she hasn’t told about the gravity of the illness to her husband because this can actually trigger his symptoms.

When asked about how stressful it gets, she mentions that it gets extremely stressful at times, and it can become quite frustrating. She gave a long pause before answering about the most frustrating thing and then later on said that she has long hours at work and when she has to come back home and cook and take care of children and do errands, that is what bothers her. She also mentions that he complains that she does not give him time.

In addition, they do go out to socialize but mostly with family. However, no one knows about the illness and she mentions that it is due to the stigma and the fact that there isn’t much understanding about mental illness.

She also went onto say that his illness does not really show when they are out socializing and he is extremely superb in social gatherings. *Jo bhi kehtey hain ghar mein kehtey hain.*

When asked had she married him had she known about the mental illness, she answered after a long pause and said that even if she had been told before, she would not have understood it completely so it would not have mattered as much. And she also said *pyaar tu andha hota hai,* but she quickly added that she did see that the family was well-known and well-respected, which is why she did get married. The family is also religious which is something that she wanted, as well. However, she laughed and said that sometimes when they are having a fight, and fights usually happen once a day, she says to him *mujhe bataya nahi tha.* She said that it was a form of *dhokai.* However, she maintained that the decision would not have changed. In fact, she said she would have preferred to know because she would have been more guarded had she known about the illness.

The family dynamics did not change and the in-laws have been very supportive.

The older child is 10 years old and she teachers her how to handle the father.

He is quite compliant with medications and takes them himself so she does not have to worry about that.

The relationship has not changed since she has found out about the illness.

She says that her frustration manifests in the form of aggression at times because she has too many responsibilities. She also adds that they fight on silly things. He exhibits childish behavior at times which irritates her. But when he is working, then he does not bother her that much. Also, when her in-laws were still in Pakistan, then they also provided support to her husband, so he did not bother her too much.

She has taken up additional responsibilities which she feels that a husband should take care of. She works and has been working for a long time. She picks up and drops children to community school in the evening. She also helps them out with homework and that gets too much for her. She also has to cook but sometimes he also does cook but she has to motivate him to do so. Sometimes, even the payment of bills gets delayed so she has to do it. She also does grocery shopping.

In leisure time, she first stated that *leisure ka time hota hee kahan hai* but also added that sometimes she would be on the Internet or sometimes talk on a WhatsApp group but she also makes a point that he does not even like that much.

She feels she knows enough about the illness; she manages the symptoms and she has researched online.

When asked about the personal reasons for staying back in the marriage, she stated that she would not say that it is because of her children, but rather because she feels that she made that choice and it was simply her luck that he had a mental illness. In fact, she feels that she has more of an important role to play now that she knows that he has an illness. *I can keep him stable.* Social reasons were the fact that her in-laws have been supportive; emotionally and financially.

She does not feel that it is his fault to have the illness and also feels that she can fix him.

In addition, things which may lead to divorce could be the familial matters but she feels that she has never considered divorce for her marriage. At times, when they are fighting she would say that *mein ja rahi hun* but she never does it. And she mentions that whenever she talks to her friends, she always says *mein Hussain ko kabhi nahi chorungee* because she knows the backdrop of the story. He is not normal but that is acceptable to her.

She also states that everyone romanticizes marriage and *du teen suhanay din hotay baad mein haqeeqat pata chaltee hai’.*

Family is more important than the relationship that exists between two individuals.

The essential building blocks for raising a healthy family include trust and understanding and she adds that it is present in her marriage.

She then later concludes in the interview that there should be more awareness about mental illness and it should not be stigmatized. She feels that whenever she is even coming to the clinics over here, she feels a bit odd and she does not want anyone to see her. Moreover, she also quotes an incident where she feels torn between her job and her family. Often her kids would call her up and say that they are hungry and her husband is not going out to get anything for them because he is too inactive. She gets bothered by this inactivity.

Overall, she seemed quite positive but obviously stressed out. Her reasons stemmed from the fact that she believed it was her duty to do so, and that it was quite altruistic to help out someone suffering from mental illness and also because she does have attachment with him. The family support actually really helps a lot which just adds to the hypotheses that joint family systems may be extremely helpful in keeping the marriage intact in the Pakistani scenario.
