# Supplementary material for: Exploring effects of severe mental illnesses on marriages: A qualitative study from Karachi, Pakistan
Source: PLOS Glob Public Health. 2025 Dec 23;5(12):e0005652. doi: 10.1371/journal.pgph.0005652 (PMC12725543; doi:10.1371/journal.pgph.0005652)
Supplement: S1 Data — (ZIP) [file pgph.0005652.s001.zip › Transcriptions/Case 2-6 Transcripts/Case 4/C4-7.docx]

**Case 4**

**Psychiatric Illness:** Schizophrenia

(Has done Bachelors in Mechanical and Electrical Engineering, is currently retired but worked for 43 years. Has been married since 1967 so around 48 years. They have two daughters and they are married. He is living in a separate portion with his wife, but the downstairs portion belongs to his younger daughter. He has pension of around Rs. 60,000. He mentions that he has distributed all of the money he had earned over the course of the years (for his grand-children’s education) and also mentions that he helped out his brothers who weren’t that well-off. And he kept enough for himself that medical treatment and other basic costs would be covered. His wife has done her MBBS, which she completed in 1962. Psychiatric history in family is not present. Mentions that wife does have a psychiatric illness from a long time. They are not related through family.

**Interviewer:** Aap ko pata hai kay aap ki wife kab sey beemar hain?

**Interviewee:** Nahi ub tu, pheley tu bari hyper hoteen theen. Matlab ghusssa aur yeh woh aur…. Uhh lekin face karna para.

**Interviewer:** Tu shaadi huwi thi usi waqt say ya thoray arsay baad?

**Interviewee:** Nahi nahi thoray arsay baad. Kaafi arsay baad. Merey Saudi jaaney kay baad

**Interviewer:** Aur aap Saudi Arabia kis year mein gaye thay agar aap ko yaad ho tou?

**Interviewee:** Haan 1969 mein

**Interviewer:** Tu matlab shaadi kay 2 saal?

**Interviewee:** jee

**Interviewer:** aap ko andaaza hai kay inki umer kitni hogee behavior mein jab change aya?

**Interviewee:** Behavior mein change…

**Interviewer:** Acha unki kitni umer thee jab aap logo ki shaadi huwi?

**Interviewee:** Hum mian biwi mein approximately 2 saal ka farq hai

**Interviewer:** Kabhi yeh hospital mein admit huwi hain apni beemari ki waja say?

**Interviewee:** nahi treatment leti hain. Unko dusri problems huwi hai, UTI hua tha aur dengue hua tha

**Interviewer:** nafsiati beemari ki waja say nahi huwi?

**Interviewee:** Nahi, time to time, Dr. Hanif Mesiya kay treatment mein theen aur phr Dr. Musarat Hussain… tu unkay treatment mein theen aur Hanif Mesiya ne hum say kaha tha kay yahnee woh bhi dawaiyan de rahay thay lekin faida nahi horaha tha, kaafi visits huay thay, tu …

**Interviewer:** kabhi psychiatry ward mein admit huwi hain?

**Interviewee:** Nahi hua yeh kay psychiatry ward mein admit karne ki koshish ki ..unhon ne kaha kay agar unko ECT diya jaye tu guarantee, unhon ne guarantee kaha ..mein tu guarantee pe heeran reh gaya kyunke guarantee tu hotee nahi hai kisi life aur kisi cheez ki koi guarantee nahi tu mujhe woh word bara prick kara…kay yeh kya keh rahain. Agar 3-4 ECT dedengey tu yeh bilkul theek hojayengee. And then I said this is very well and good and then 3-4 say lakay 13 ECT dediyee. And said that eik din kay liye admit kardain aur phr hum ne maan bhi liya kay hum jo hain admit hojatee hain tu unhon ne kaha kay aap paisay commercial department say bharwa lijiye aur phr 15000 bhar diyee. And mein unsay ulhaj para kay doctor sahib 15000 eik din kay liye, kis cheez kay liye, woh bhi ECT kay liye, 4-5 seconds kay liye hota hai, and ECT kay liye anesthesia diya jaata which is only a minor thing, tu usne kaha kay anesthetist ayega. And then he said theek hai mein 5000 kardeta hun tu mujhe yeh bhi ajeeb laga kay itni bhi knowledge nahi hai kay ECT kya hota hai, at least he should know about this. Pay kardiya, wife ko admit kardiya. And phr mujhe khayal aya kay and I asked him kay aap admit kyun kar rahay hain tu he said only sahoolat kay liye, yaheen …. Meiney kaha doctor sahib yeh tu chal phir raheen hain mein sab karwadeta hun aur aap ko aglay din aap ko computer pe sab kuch miljayega. Phr unhon ne kaha kay aap meeting room mein agaye and phr assistant bhi agayee. And assistants ne mera favor kya kay admit karne ki koi zaroorat nahi hai. Beharal …yeh around 5 years.

**Interviewer:** Tu aap kab say unko psychiatrist ko dikha rahay hain? Jab say aap log Saudi Arabia mein thay aur unko yeh shuru hua tha?

**Interviewee:** Jee

**Interviewer:** Treatment karrahay thay?

**Interviewee:** Yes she was taking tricylic drugs at that time and she was quite stable

**Interviewer:** aap ko waisay pata hai kay inki beemari ka kya naam hai?

**Interviewee:** suicidal tendencies hain.

**Interviewer:** aap directly psychiatrist kay pass hee gaye?

**Interviewee:** Jee.

**Interviewer:** Faith healers waghera?

**Interviewee:**  aisa belief hee nai hai

**Interviewer:** health issues waghera hain?

**Interviewee:** Psychic problem hai aur mein dusra mein diabetic hun since 30 years. (he himself suffers from depression)

**Interviewer:** acha aur inki beemari ki waja say dusre rishte darrun waghera say maslay masail?

**Interviewee:** Nahi buss yeh kaafi hyper hojate theen. Tu yeh masla tha hyper istarah hoteen theen mujhe marne kay liye bhi uthtee theen aur mein samajhta tha aur yeh psychic case hain aur mein kya karun inka sirf ilaaj hee karana tha

**Interviewer:** Tu unko jab 69 mein shuru hua tu aap ka kya radeamal tha?

**Interviewee:** Nahi jab unko shuru hua tu istarah kay doctors nahi milte thay. Eik hee doctor mila tha jokay MRCP tha. Psychiatry mein. Tu hamare office ki jo bari clinic thee tu unko diazepam detey thay tu….lekin ussay theek nahi hua, unko samajh nahi aya, and bethay bethay yeh hua kay eik book rakhi huwi thee, eik diagnostic book thee woh meiney parhna shuru kya aur psychic chapter parha. Merey dimag mein aya kay yeh ajeeb ajeeb harkatain kar rahee hain tu yeh psychological problem hosakta hai aur phr meiney pura chapter parha aur koi 100 200 pages thay. So ussmein woh akey yeh jo baatein karteen theen na schizophrenic type keen aur yeh chapter parhne kay baad mujhe yeh khayal aya kay inko sawaye psychiatrist kay pass lekey jaanay kay illawa koi chaara nahi hai. Phr meiney woh kya kay ummm….search out kya kay phoocha kay …fallah hospital mein psychiatrist hai, MRCP hai…Dr Saeed ubh tak mujhe naam yaaad aya, aur unhon ne foran admit karliya…15 din admit kya aur Etivol shuru kardee, aur phr 2-3 dinn kay baad hee inki tabiat theek hogaye …expenses company basis pe thee and phr woh theek hokay agayein and phr stable raheen

**Interviewer:** acha kab tak stable raheen?

**Interviewee:** jab tak hum Saudia mein thay aur dawai miltee rahee

**Interviewer:** acha aur aap Saudia sab wapis kab aye?

**Interviewee:** 2002 mein aye thay. Tu yahan ayeen tu phr yeh dawai nahi milli, shortage hogaye thee aur ….. inko yehi dawai suit karee. Koi suit nahi karrahee aur even ECT suit nahi kya

**Interviewer:** aur jab aap Pakistan wapis ayein tu phr Dr. Hanif kay pass aye?

**Interviewee:** Hanif kay pass say hum chale gaye jab ECT wala chakar hua. Tu hum ne kaha kay yeh saheeh nahi hain aur 2 saal tak ilaaj karwaya tha aur theek hee nahi hua

**Interviewer:** acha tu woh waisee hee rehteen theen, cheekna chilana aur aggressive waghera?

**Interviewee:** Haan aggression, hyper, bipolar..kabhi soft side pe chalee jaatee theen aur kabhi high side pe. Bipolar depression type hogaya tha baad mein..Tu phr mein Dr. Musarat Hussain kay pass gaya tu unhon ne mukhtalif dawaiyan shuru kareen aur change waghera ki aur aakhir woh Respidrol par jakey set huwin. Epival dee aur pata nahi …5-7 dawaiyan ki list thee, aur plus… revitrol. 2 mg.

**Interviewer:** acha yeh bataein, 69 mein hua tha, aur aap ki shaadi ko kuch arsa hee hua tha

**Interviewee:** nahi 69 mein nahi hua tha..yeh hua tha unko 2-3 saal baad 69 kay..

**Interviewer:** Tu shaadi ko 4-5 saal hogaye thay?

**Interviewee:** jee aur acha issay pheley khud batatee theen kay shaadi say pheley ..jawani mein, shaadi say pheley, psychic ilaaj hochuka hai, Dr. Zaki Hassan say

**Interviewer:** acha tu aap ko maloom tha?

**Interviewee:** Nahi mujhe nahi maloom tha, yeh khud batatee hain

**Interviewer:** Matlab iska matlab shaadi say pheley inko problem tha?

**Interviewee:** Jee jee bilkul psychic problem tha aur woh bhi ilaaj karwaye, peer waghera ka ilaaj bhi karwaya inkay parents ne

**Interviewer:** acha humein zyada help hogee agar aap sarey facts bataye kay shaadi say pheley bhi kya tha..beemari say pheley

**Interviewee:** beemari tu nahi thee unko but she was very sensitive.

**Interviewer:** Tu matlab psychiatrist key pass nahi lekey gaye thay?

**Interviewee:** Nahi lekey gaye thay, Zaki Hassan kay pass. Shaadi say pheley waldeen lekey gaye thay

**Interviewer:** aur 5 saal kay baad jab yeh hua tu kya aap ne apne waldeen ko yeh bataya?

**Interviewee:** walid tu merey thay nahi. Walid tu jab meri education khatam hoye thee, death hogaye thee unki. Walda *pause* walda zinda theen tu hum yahan per thay bhi nahi tu walda ko pata hee nahi tha, aur phr walda ke death hogaye

**Interviewer:** acha tu aap ne apnay family members ko bataya tha? Bhai waghera ko?

**Interviewee:** Haan unko tu sab ko maloom hai…

**Interviewer:** acha tu unka kya radeamal tha?

**Interviewee:** nahi kuch khaas radeamal nahi tha

**Interviewer:** acha aur aap ki jo kafiat hai udaasi aur pareshaani ki, pheley mahsoos hotee thee ya sirf ubh mahsoos hotee hai?

**Interviewee:** nahi nahi mujhe pheley kuch nahi tha, suddenly hua tha. Yeh mujhe suddenly hua… *pause* 10-15 saal pheley

**Interviewer:** Acha tu aap uswaqt Saudi mein hee thay? Pakistan mein nahi thay?

**Interviewee:** Saudi mein hee thay. 15 saal pheley

**Interviewer:** retirement kay aas paas?

**Interviewee:** Nahi nahi retirement say 10 saal pheley

**Interviewer:** Tu aap ki Saudi mein job tough thi?

**Interviewee:** Nahi mujhe boht interest tha

**Interviewer:** Mera matlab hai kay long hours thay?

**Interviewee:** Sometimes mujhe karna parta tha

**Interviewer:** Acha tu yeh akelee hoteen theen tu bacho ka yeh khayal rakhtee theen?

**Interviewee:** Nahi bachian yaheen theen, apni nani kay pass. Education ki waja say. Problem wahan yeh tha kay 70s mein education itni achee nahi thee Saudi mein.

**Interviewer:** Theek hai, aap ka support system kaisa hai? Aap ko madad miltee hai? Kay kabhi inka khayal rakhna ho ya dawaiyan ka? Doctor kay pass laanee ka?

**Interviewee:**  Zyada tar mujhe hee khayal rakhna parta hai waisay meri beti waghera karletee hai aur kabhi doctor waghera kay pass jaana hota hai tu lejatey hain. Mein gari tu chala leta hun lekin yahan ki traffic itni kharaab hai kay apni zabaan kharaab karne wali baat hai. Wrong side sey araha hai, mein driving boht kam karta hun. Boht hee necessary hota hai tu mein tab ke karta hun. Ubhi yahan traffic jam tha aur humein paidal aana para. Aur traffic jam kyun tha aap sunein zara. Paper yeh jo home economics haina, jo paper hai woh dupher mein tha aur subah mein unhon ne paper nikal diya. Larkyan saari sarak pe bethi huwi hain. Aur hum ne kaha kay yeh larkiyan ubh uthenge nahi. Eik larki ja rahee thee ussay pata challa.

**Interviewer:** acha kabhi unki boht zyada tabiat kharab hotee hai tu zyada pareeshani ka saamna karna parta hai?

**Interviewee:** Nahi ubh itni pareeshani nahi hoti hai.

**Interviewer:** Matlab iss beemari kay hawalay say? Aap keh rahay thay na aggressive hojatee hain, chikna chilana tu ussmein pareeshan

**Interviewee:** Haan bilkul ussmein boht pareeshani hoti hai

**Interviewer:** Kya woh uncontrollable hojatee hain ….

**Interviewee:**  haan

**Interviewer:** Tu aap kya kartey thay?

**Interviewee:** Mein bhi thora cheekh pukaar karta tha. Kyunke mujhe yeh nahi pata tha kay yeh itna hyper hojayengee

**Interviewer:** Kabhi boht udaasi hotee thee, rona aata tha, ghussa boht aata tha?

**Interviewee:** Ghussa boht aata tha

**Interviewer:** acha, kabhi ghussay mein aap ne haath uthaya hai?

**Interviewee:** Nahi kabhi nahi uthaya

**Interviewer:** acha aap ko lagta hai kay aap kay support say inko madad miltee hai? Jaisay aap kay honay say hosla milta hai?

**Interviewee:** Haan boht hosla milta hai, balkey kabhi mein bahir chaley jaona , bread waghera lena kay liye tu yeh pareeshan bethi rehtee hain jab tak mein wapis nahi aajata

**Interviewer:** acha aap logo ka bahir milna milana hai? Logo mein

**Interviewee:** nahi boht kam

**Interviewer:** uski waja kya hai?

**Interviewee:** tabiat nahi chahtee

**Interviewer:** dunu ki?

**Interviewee:** nahi matlab aaj kal jo system hogaya hai yahnee aaj kal jo system hogaya hai kay aap ko parosee ka nahi pata hota kay kon barabar mein rehta hai aur usska naam kya hai.

**Interviewer:** aap rehtay kahan hai?

**Interviewee:** Gulshan Iqbal mein

**Interviewer:** acha agar log koi sawal karte hain kyunke hamaray yahan tu log kaafi sawalat kartey hain tu inkay barey mein koi sawal karta hai kay inko kya problem hai, tu aap ka kya jawab hota hai?

**Interviewee:** Nahi…. Mein kuch nahi kehta

**Interviewer:** Acha tu koi aap say kuch phoochta hai?

**Interviewee:** nahi koi nahi phoochta

**Interviewer:** Hmm, jaisay rishtedaar waghera?

**Interviewee:** sab ko pata haina

**Interviewer:** koi yeh log kehtey hain kay phr chordou ..aisa kuch waghera?

**Interviewee:** Nahi aisa kuch nahi hua. Aisee koi koshish nahi ki

**Interviewer:** eik aisay hee sawal karna chahoonge kay agar shaadi karne say pheley agar kisi ko maloom ho kay jin say shaadi honi waali hai kay inko nafsiat ki problem hai tu shaadi karni chahye hai phr unko?

**Interviewee:** Bilkul karni chahye hai

**Interviewer:** Acha aur agar aap ko pata hota shaadi say pheley kay inko nafsiat ka problem hai tu kya aap shaadi karte phr unsay?

**Interviewee:** Bilkul karta kyunke mujhe jo haina mein itni tough life say guzra apni education tak, balkey yahan reh kay bhi meiney Karachi electric mein kaam kya, bari tough life thee. Tu…uss tough life ne mujhe bara dilli tor per soft kardiya. Baaz waqt yeh hota haina kay tough life guzaar kar banda ghussay mein aajata hai ya irritable hojata hai lekin uss saaf life ne mujhe boht soft kardiya hai. Yahnee itna soft kardiya kay merey career mein excellent kay illawa aur koi cheez nahi hotee theee.

**Interviewer:** Hmm

**Interviewee:** balkey KESC mein bhi meiney itni tough life guzaari lekin jab mujhe certificate diya tou ussmein likha hua tha kay he is very honest and he is very calm and it is difficult to replace his position

**Interviewer:** Hmm. Theek hai. Acha. Waisay ghar ka mahool kaisa hai..aap ko lagta hai kay aap ka ghar dusrey ghar kay mahol say different hai?

**Interviewee:** Nahi agar aap dekhain tu aap compare karengey tu dekhain mein humeisha apne neechay walay logo say compare karta hun. Uper say nahi karta hun halankha meri family mein boht well to do log hain, merey eik chacha hai woh eik boht baray surgeon thay, unhon ne eik aurat ko mard banaya tha, lekin meiney aap ko kabhi compare nahi kya. Merey jo walid thay, he was a headmaster of a school. Tu oriental languages parhatey thay, arbi, farsi…uswaqt farsi India mein kaafi woh thee. Urdu tou..meiney kabhi compare nahi kya kay merey chacha kay pass itna paisa hai

**Interviewer:** hmm jee lekin hamara matlab yeh tha kay nafsiat ki beemari ki waja say farq parta hai family life pe. Zyada responsibilities bhar jaate hain, dawai dena aur careful rehna kay kya kahain

**Interviewee:** Nahi shuru mein tou mein unko mein hee dawai deta tha, and wahan Saudi Arabia mein mujhe doctor na kaha tha kay aap hee unko time mein dawai dein. Tu woh tu mujhe karna parta tha and baad mein yeh khud used to hogayee theen and for a few weeks, mujhe dena para tha

**Interviewer:** acha aur ubh khud leletee hain?

**Interviewee:** Jee ubh aisa koi problem nahi hai

**Interviewer:** acha aap yeh batayein kay iss beemari ki waja say aap kay bacho pe koi asr para hai? Kyunke maa hain aur aap ki betian hain….kya radeeamal hain?

**Interviewee:** nahi para itna kyunke mein aap ko bata raha hun na kay yeh yahan rehtee theen

**Interviewer:** Tu unki attachment hain apni ami say?

**Interviewee:** jee hai

**Interviewer:** aur apni ami ka khayal rakhteen hain?

**Interviewee:** Haan rakhteen hain

**Interviewer:** acha inko yeh nafsiati beemari kay barey mein maloom hai aur understanding hain?

**Interviewee:** Jee kyunke jiswaqt unko problems huwi theen tu yeh log kuch nahi bolteen theen kyunke meiney inko bata diya tha kay this is a problem and it is just like a disease tu yeh eik disease hai, aur isko tu suffer karna hai aur phr meiney inko educate kardiya tha. Tu kisi qasam ki bhi kuch nahi kartay thay.

**Interviewer:** Acha aur jab aap log young thay aur aap nahi shaadi shuda huay tou tu uswaqt aap logo ka logo say milna milana kaisa tha?

**Interviewee:** Boht tha, hotelling karna, idher udher jaana hai, young life mein jistarah enjoy karte hai

**Interviewer:** waisay tu aap ki shaadi ko kaafi arsa hogaya hai lekin agar aap batana chahain tu shaadi shuda zindagi nafsiati beemari kay hawalay say tu aap kis tarah describe karengee kay kistarah shaadi shuda zindagi guzri hai?

**Interviewee:** *pause* achee guzri hai. Mein itna matlab hai…sensitive nahi tha iss mamlay mein jo cheez hai usko face karna hai. Kyunke mujhe yeh shuru say hee adaat thi face karne kee. Meiney shuru say hee itna face kya hai, mein fees nahi desakta tha, matric mein itni kam fee thee. Meiney woh maafi karali thee kyunke mein acha student tha. And phr mujhe best college mein admission mila kyunkay merey achay marks aye.

**Interviewer:** acha theek hai yeh tou boht achee baat hai. Waisay aap ko lagta hai kay inki tabiat ki waja say aap ko depression hua hai?

**Interviewee:** inkay haalat ki waja say nahi hua. Pata nahi suddenly kaisay hogaye aur bari ajeeb shakal mein hua, buss aisa hua kay aisa lagta tha kay mein kisi ko mardunga aur aisa hota tha kay mein namaaz mein kharah hota tha tu mujhe lagta tha kay jo merey pass hai mein ussay mardunga.

**Interviewer:** acha nahi aisa tou nahi hua tha kay boht zehni dabao hua tha unka khayal rakhte hoye?

**Interviewee:** nahi eik dum sudden hua aur phr eik dose kay baad mein bilkul calm down bhi hogaya. Unhon ne mujhe diya tricyclic drug..

**Interviewer:** acha aap kis kay pass ja rahay thay?

**Interviewee:** Koi Egyptian doctor thay

**Interviewer:** Acha aur yeh Saudi mein hee hua tha?

**Interviewee:** Saudi mein hee hua tha

**Interviewer:** acha aur ubh aap ki tabiat kaisi rehtee hai?

**Interviewee:** jee theek rehta hun lekin kabhi kabhi jisko aap kehtey hain OCD yeh hojata hai. Atak gaya khayal tu nikalna usko bara mushkil hota hai

**Interviewer:** acha aur aap ka din kaisa guzarta hai? Subah say shaam tak?

**Interviewee:** Acha subah uthta hun aur nashta karta hun aur nashta karne kay baad computer pe beth jaata hun. Du ghante computer kay beethnay kay baad khanay ka waqt hota hai. Khaana kha liya, namaz parhlee, sogaya. Eik ghanta, der ghanta, chaar bajey utha. Aur walking karke hee challa jaata hun namaaz parhne. Walking karke aur phr shops waghera mein. Baat cheet karta hun aas pass logo kay saath aur phr maghrib ki naamaz parhta hun aur phr TV dekhta hun eisha ki naamaz kay baad … raat ka khaana khaya aur phr TV dekha. Aur TV mein yeh nahi dekhta hai, jhagray waghera nahi dekhta… *laughs* mein jakey phr woh English movie waghera dekhlee. Tu jab dawa kha leta hun, Rivitrol waghera aur phr mujhe neend aatee hai aur mein bistar mein chalay jaata hun

**Interviewer:** Acha aur aap ko lagta hai kay aap ko inki beemari kay barey mein achee khaasi maloomat hai?

**Interviewee:** maloomat hai, chunkey parha tha aur diagnose bhi meiney hee kya tha *laughs* yahnee general doctors tu diagnose nahi kar paye

**Interviewer:** Acha tu aap ko kya lagta hai kay inko kya problem hai?

**Interviewee:** Inki life bhi bari tough guzri hai.

**Interviewer:** Diagnosis kya hai?

**Interviewee:** Bipolar depression

**Interviewer:** acha aur aap computer use karte hain tu kabhi online waghera search kya hai kay yeh kya hota hai?

**Interviewee:** zyada tar medical hee jaata hun aur jahan emails diye hain, associations waghera aur jo bhi healthcare, apne diabetic kay lihaaz say, kya naye research horahee hai

**Interviewer:** acha aur shaadi kay 4-5 saal baad hua tu kabhi kabhi aap ne yeh socha kay mein shaadi ko na chalaon aur chordun? Kabhi aisa koi khayal aya? Kabhi kisi ne bola? Kay dusri shaadi karlo?

**Interviewee:** nahi boht puraani baat hai, balkey yeh….*pause* shaadi kay baad 1-2 saal baad hee aur uswaqt inki halaat tu theek thee tu pata nahi walda ko kya hua tha unka kya khayal tha kay buss woh keh raheen thee kay mujhe yeh discontinue kardena chahye hai.

**Interviewer:** Unki kya wajoohat theen?

**Interviewee:** buss you know pheley kay, walid tu kher parhay likhay thay, walida ne tu koi taleem waghera nahi le thee aur aaj kal tu taleem yafta log bhi aisee cheezain kar rahay hain. Zara say hua tu biwi ko chordu. Aaj kal divroce kitnee common hogaye

**Interviewer:** Acha tu aap ne kabhi bhi divorce kay barey mein nahi socha?

**Interviewee:** Nahi

**Interviewer:** acha tu aisee kya wajoohat hain kay aap ne yeh nahi socha? Aisee kya wajoohat hain? Kaafi log sochtey hain

**Interviewee:** Kyunke mein issko eik bura fail samajhta hun

**Interviewer:** acha aap ko kya lagta hai, kis soretahal mein du logo ko talaaq kay barey mein sochna chahye hai?

**Interviewee:** sochna hee nahi chhaye hai. Mein tu kehta hun

**Interviewer:** Koi bhi soretahal?

**Interviewee:** haan compromise karna chahye

**Interviewer:** acha aap ko kabhi aisa lagta hai kay inki ko kafiat hai issmein inki koi galtee hai?

**Interviewee:** Inki galtee..galtee tu nahi hai, inki life bhi bari hard guzri hai, parhai bhi mushkil say, jaisay yeh batati hain, inki life boht tough thee. And parents itnay well to do nahi thay

**Interviewer:** acha yeh batayein, kay shaadi aur jo aapis mein mian biwi ka rishta hota hai woh zyada important hota hai ya pureey khandaan ki?

**Interviewee:** pheley tu husband wife ki aur phr ghar.

**Interviewer:** acha aur aap ko kya lagta hai kay eik pursukoon khandaan kay liye kya cheezain zaruri hotee hain?

**Interviewee:** apne aap mein sukoon hona chahye. Aap tolerate karein inlogo ko.

**Interviewer:**

**Interviewee:**

**Interviewer:**

**Interviewee:**

**Interviewer:**

**Interviewee:**

**Interviewer:**

**Interviewee:**

**Interviewer:**

**Interviewee:**

**Interviewer:**

**Interviewee:**

**Interviewer:**

**Interviewee:**

**Interviewer:**

**Interviewee:**

**Interviewer:**

**Interviewee:**
