# Supplementary material for: Exploring effects of severe mental illnesses on marriages: A qualitative study from Karachi, Pakistan
Source: PLOS Glob Public Health. 2025 Dec 23;5(12):e0005652. doi: 10.1371/journal.pgph.0005652 (PMC12725543; doi:10.1371/journal.pgph.0005652)
Supplement: S1 Data — (ZIP) [file pgph.0005652.s001.zip › Transcriptions/Case 2-6 Transcripts/Case 4/C4-8.docx]

**Case 4**

**Your age?**

28

**Studied?**

I’ve studied bachelors. I’m working right now. Part time. I’m a software engineer. I’ve been working since before marriage, but after marriage it is just my 6^th^ month. I quit because of his condition.

**Did you find out after marriage?**

Been married for 3 years, found out after.

**Was he diagnosed before marriage or after?**

Before.

**His family never told you?**

No

**Did he visit a doctor?**

His episodes, before marriage had a 2 year gap, after marriage, another 2 year gap, so in total a 4 year gap. So 2 years before marriage, he followed with a doctor, and took medications for 1.5 year, then he stopped the doctor, but took his meds. The doctor said he’ll increase follow ups depending on the number of episodes. So because of the gap he wasn’t on meds, nor did he do any follow up.

**What’s your family set up?**

Before, we were in a joint family system. But after he got diagnosed, his thing is genetic, his mother had it too, she used to get angry, her behavior used to get ignored. But with her, when he was in the hospital, and she used to act up, and we didn’t get along, and I had no idea what was happening. And no one used to be home, everyone was in the hospital with him. 1 year ago, everyone was gone for hajj, his dad, mom and brother were gone for hajj. At home it was me, *jethani*, and just him. Who knows? Maybe he felt a lot of anxiety, even though he wasn’t stressed. I used to tell him to work, he’s lazy, doesn’t take responsibility, he’s the youngest. Maybe he felt anxiety, the barrier broke, and then he had a lot of psychotic effects. I didn’t know, but my *jethani* had seen his episode during her own wedding days, and she immediately identified, I had no idea, he had a phase in and out, this was 2 years after marriage. We had a baby by then.

Now, I tried very hard to bring him to my parents’ home. Because his mother is also sick, she is difficult. So I moved away for a year, like a separation. Because for a year or so, he was almost not conscious. The episode came 2 years after marriage. So for the last year, he had severe episodes, I tried to go back and resettle with him, but even his own mother was anxious and acting up, I couldn’t handle. Then slowly things resettled, and I went back.

**Where you live right now, how many people are there?**

Downstairs, his parents. Upstairs, his brother, jethani, their two kids, and the three of us.

**How many earners?**

2, brother and him. Monthly about 1 lac after all the cuts and taxes.

**How old is your son?**

2 years 3 months.

**What has your husband studied?**

Bachelors in Computer Science. My father is a Civil Engineer.

**Are there any psychiatric issues in your family?**

I’ve seen depression a little bit over issues here and there, but nothing that required medication. Therapy yes, but nothing more. I’ve realized what their issues were now.

**So you think he was sick for a few years before his marriage?**

Yes, he was sick before.

**How many episodes after marriage?**

The big one was 2 years after marriage. But I feel the hints were always there. He used to get angry, beat himself up, he used to do weird things. I guess I never picked it up, strange as it is. We used to fight, I used to think he hurts himself out of guilt. Couples always fight. I never thought it was symptoms or episode. So after 2 years, saw the big episode. He was in hospital for 1 month. He completely lost control, weird things he was saying, got paranoid, he used to feel he was a superhuman, felt electricity through him. The medications weren’t working. And so every 2-3 months he kept getting lots of episodes. Because his mother was always shouting at him, plus we weren’t so good. So lots of stresses.

**He has been on medication since?**

Since 2007.

**Have you ever tried homeopathic or hakeem?**

Yes, even now, his dad is a homeopath. First it was really regular homeopathic medications. I had no idea about them. They’ve tried *pir* and faith healers. Actually, the episodes began as extremism, Islam-related issues he got very passionate about, so they thought it was *jinn.* I’m not sure, but I think they did take him to some *baba* or *pir.*

**Does he drink or have any addictions?**

No

**Do you have any other concerns? Health or financial?**

Yes, I had some hormonal issues due to stress. I’m still a little sick, have a lot of bleeding vaginally due to hormonal issues. Now I’m taking medication. I lost a lot of weight and I’m still not gaining any. After feeding my child, I was very stressed, not paying attention to myself.

**How are your relations with the rest of your family?**

Yes, not good with his mom. But no financial issues.

**You had no idea in those 2 years? Any suspicion?**

No, I never even thought at all about this. I just knew *pagal*, nothing more, had no idea that this existed, I read up on it later as I found out more.

**Your parents know?**

About 20 days after they returned from hajj, he was admitted in the hospital. His brother’s friend helped out with that, I had no file, no idea how to deal. When his parents returned, I told my folks.

**What was their reaction?**

They were angry… At first, shock, then anger, the explanation we got from their side, about why we were kept in the dark, was rubbish. That we didn’t ask, that’s why they didn’t inform us. So I left to my house, for 15 days of the hospital stay. Even after he returned, my family told me not to return then, even now, they’re rigid about my decision. They don’t listen to my complaints, because they didn’t think I should return.

**Did your parents suggest a divorce?**

Yes, everyone said with your child, you can’t continue like this. He won’t take care of the child. He’s sleepy, doesn’t go to office regularly, how will you deal? That’s why, I restarted my job. I rebuilt myself after this huge crash, took me 6 months to get back up. I restarted my job, recovered quickly after that. I wanted a little stability, it’s part time, I could become full-time when I wanted. My concern is my son, I want to be able to support him financially. Even now, we’re comfortable. I have 4 brothers, money is not an issue. My elder brother is still upset with me for going back to that home.

**Why are you in the marriage?**

I’m attached to my husband, that, and my baby.

**Parent’s support?**

Luckily, everyone is supportive. I’m educated, financially we’re okay, I can support care here.

**Do you get any help or support when you’re very worried?**

I don’t make my issues very obvious. I keep it inside, and bear. I do have support, but I have a son, I can’t succumb due to depression. I have to care for him, feed him. I can’t give up. My husband only supports financially, otherwise he doesn’t do anything. He can’t help with groceries, I have to do his food, everything, his weight gain, diet, I have to take care of all that. I don’t take care of the medication. I’ve returned after 3 months, so I’m not doing that. His father does that. I’m not involved in that. His father and brother. He has 1 brother, 1 sister. She’s married.

**Do you get angry or frustrated?**

Yes, a lot. Even at my son. But not more than yelling. Even at him, I do vent out, I do yell at him. But only to a certain level. But it falls on deaf ears.

**Do you bring him to the doctor?**

I started now. Before his brother did.

**Are you ever tired of it?**

Not yet.

**Do you think your support helps him in any way?**

No. In fact, I think it makes him a little more lazy. I felt he would feel more pressure when I gave up and returned. But he didn’t. He got even worse in his attitude. Maybe he’s a little relaxed now. Because I control his diet etc. His disease has improved. His graph is good. Episodes decreased in frequency.

**Social life?**

He’s not very social. I drop my son off at my mom’s when I go to work. He picks us up so that’s a little socialization. He gets worried or paranoid in crowds. Some psychological issues. He hesitates when I ask him to go out. But if he wants to go somewhere, he does so.

**Do people get curious or ask?**

In his family, other than immediate, there isn’t a lot of interaction after his episode. I don’t interact with them, and I know people will ask a lot of questions, they’ll give me a negative vibe. I don’t feel good. No one really asks me. I think initially, whoever wanted to ask and whoever knew, they still know. I feel when people do ask questions, or even my brother asks me how life is, I feel troubled, I don’t like being asked about my troubles.

**Do you think if someone knows before marriage that someone has a mental illness, should they get married?**

No. Well, even if you do get married, you shouldn’t start a family or have a kid.

**Would you have married?**

No, had I known, I wouldn’t have.

**What was your reaction in the beginning?**

I was helpful. I supported him. I wasn’t thinking very long term, I wasn’t scared or anything, but in those days, I used to be brave and commanding, I wasn’t with in laws, they were away, I was the authority and felt responsible. I couldn’t give up and run away. My *jethani* was in town, but she used to be with her family more. She herself kept out of my way because she felt her 2 kids would be a bigger stress. Initially, I didn’t react very badly. I thought he needed medications, maybe I thought he would go to the hospital and come back in a bit. I had no idea.

**Do you wish someone sat with you and explained the disease and its impact on you?**

Yes, I knew I had to take a big decision. I delayed my decision a lot, I ignored it for a bit, I decided to leave him, to not take the risk. Then other times I thought don’t think right now. I tried to patch up a lot. Asked him to live with me in my parents’ house. Suggested different living arrangements. I tried living with him again, but there were too many fights. And I gave up and went back to my parents’. He wasn’t in his consciousness. Kept delaying my decision. Stayed at home. Later on, my decision changed. But even when I decided to return, he wasn’t better, but some compromises were made. We shifted upstairs, away from his mother.

**How were your first 2 years, before the episode?**

I didn’t know exactly, had no idea about what was happening. Joint family system. After my son was born, I noticed his laziness, he didn’t socialize.

**Did you know him before?**

No

**Does your son ask?**

No he’s just 2 years old.

**You mentioned you get angry, does it ever get too much?**

I felt it more when I was alone. I used to be depressed. When I returned to his home, I feel a little better because at least society won’t question me about why I’m separated. I never want to leave him, but sometimes when we fight, I question my decision.

**What’s your day like?**

I make food for him. Take my son, and leave, return. When he’s sleeping, I wake him up. Or I do my own freelance work. I go to my mom’s, rest there. Chores here and there.

**What do you do in your leisure time?**

Computer, social media, friends etc. My kid takes a lot of my time.

**Do you think you know about this disease?**

Now, I’ve read a lot about it online. I can recognize symptoms. I’ve even seen it on forums, seeing people share their experiences. I think it’s more common in women.

**Do you think this disease is his fault?**

No, not really. He knows a lot about his disease. Too much, in fact, he even self-medicates and dictates it sometimes. He talks about sleeping, etc, he knows the right things. He’s aware. But you do need a doctor, so sometimes he goes in the wrong direction. Lots of times he rejects medications. Throws little tantrums.

**What do you think is important in a successful marriage, the couple or the whole family?**

In this kind of unique situation, you need the whole family. Both families. For a couple to be self-sufficient, one person has to be very compromising and sensible. My husband, eg, is not diplomatic nor is he politically correct.

**What do you think are important elements for a happy family?**

Peaceful environment, not to fight in front of your kids.

**What are your thoughts about marital counselling?**

It could have been very helpful in my case, but lots of people would have to be involved. Everyone needed counseling, the whole family, because everyone was affected because of the kind of set up we were living in.

**Which situations warrant a divorce?**

When you think you can’t create a happy environment in your home, because of your troubles, your spouse and your child can’t be happy. So better to create a better environment and move on.

**Do you think religion played a role in the development of his disease?**

Yes, initially in 2006-2007 he was very angry and very passionate about religion and these hate crimes against Islam and the idea of Jihad. I don’t think there was any jinn or saaya, I think it is a genetic and medical thing.

**Anything you’d like to add?**

Yes, a little about the sexual thing. Because he’s bipolar, sometimes he’s really desperate for sex. Other times, he’s completely depressed sexually.

**Do you think your decision to stay or not would be different if you didn’t have a child?**

Yeah I think so. If I did stay with him, then I wouldn’t reproduce. Don’t want to pass it on to my child. It’s genetic.
