# Supplementary material for: Exploring effects of severe mental illnesses on marriages: A qualitative study from Karachi, Pakistan
Source: PLOS Glob Public Health. 2025 Dec 23;5(12):e0005652. doi: 10.1371/journal.pgph.0005652 (PMC12725543; doi:10.1371/journal.pgph.0005652)
Supplement: S1 Data — (ZIP) [file pgph.0005652.s001.zip › Transcriptions/Case 2-6 Transcripts/Case 4/C4-9.docx]

**Case 4**

**Bipolar Disorder**

**Dr Murad**

*briefs about the study*

**Interviewer:** Tu aap ko shaadi kay kitne arsay baad pata challa?

**Interviewee:** unhon ne shaadi kay eik meheney baad mujhe bataya

**Interviewer:** theek hai tu kis tarah bataya tha?

**Interviewee:** bataya istarah tha kay mujh say chup kay medicine letey thay

**Interviewer:** theek

**Interviewee:** aur shaadi kay baadh he both violently behave kartay thay, kamray say bahir nikal detey thay. Kaafi cheezain abnormal horahee theen. Boht zyada shout kartey thay. Chotay chotay bachu ki tarah rotay thay, tu ussay mujhe realize hua kay koi problem nahi hai tu unhon ne bataya kay mujhe yeh problem hai. Parents say unko boht sunatey hain aur boht lartay bhi thay. Aur yeh batatay thay kay merey ghar walon ne merey saath boht zyadtian keen, tab mujhe challa. Buss time guzar gaya

**Interviewer:** hmm aur aap ki kitne umer mein shaadi huwi thee?

**Interviewee:** inter kay first year mein thee tab shaadi huwi thee. 18 years old thee

**Interviewer:** theek hai, aap kay waldeen ko inki beemari kay barey mein maloom hai?

**Interviewee:** nahi, ubh jakey jab problem huwi hai tu tab pata challa hai

**Interviewer:** ussay pheley kissi ko nahi pata tha?

**Interviewee:** nahi, meiney inkay hawalay say inkay ghar walon say bhi kabhi baat nai kee thee, na beemari ki na addiction kay hawalay say na drugs kay hawalay say. Na unhon ne phoocha kabhi

**Interviewer:** hmm lekin inkay parents ko tu pata tha na?

**Interviewee:** jee obvious baat hai sab ko pata tha

**Interviewer:** aur aap ne kabhi apne ami abbu ko nahi bataya?

**Interviewee:** nahhi sawal he nahi paida hota hai. Kaisay baatatee?

**Interviewer:** kya wajoohat theen kay aap ne unko nahi bataya?

**Interviewee:** awal baat tu yeh thee kay mein mentally prepared nahi the shaadi kay liye. Aur phr jab aap ko shaadi karkay bhej diya gaya aur phr shaadi kay baad aap ko yeh milla tu merey liye intahi shocking tha, aur shaadi kay baad isstarah ki problems theen. Yeh eik inteehai extremist aadmi thay, jo husband aur wife kay beech mein jo relationship hota hai physical..ussay ..woh hamara 7-8 months baad hua. Woh boht muskhil time period tha hamaray liye kyunke mein nahi kar pa rahee thee aur hum dunu boht pareeshan thay. Aur phr eik counseling kay doctor nay guide ka. Lekin jo hamara process tha woh hamaray liye boht aziat naak tha, merey liye boht mushkil tha, aur jab inki shaksiat ka pata challa, waise yeh boht achay aadmi hain no doubt. Magar mein isliye soch rahi theen kay meri family itne well-off thee, aur itnay bhai thay. mashAllah, parhay likhay hain achi posts pe hai, parents ne jaldi mein merey liye kyun bewaqoofana faisla kya? Dekh bhaal kar karna chahye tha.

Aur phr mein apne ghar walo say completely cut off hogaye, na mein jaatee thee na mein discuss kartee thee. Sab saheeh. Aur na woh hamaray ghar aatey thay, kyun mein pathan hun, hamaray yahan yeh hota hai kay betio kay ghar nahi aatey. Aur jab maheeno baad mulaqaat hotee thee tu kehtey thay kay kamzoor horahi ho, khush nahi ho kya aur phr wohi jhoot, jhooti muskharahat kay saath. Kay nahi sab theek hai, boht khush hun, Allah ka shukr hai. Aur issi tarah zindagi guzarte gaye.

Phr meiney socha kay shayad bachay hojayein tu shayad life change hojaye. 3 saal baad Allah ne kya aur phr uswaqt pata bhee nahi tha kay pregnancy kya hoti hai. Miscarriage huwi hai, aur infection hogaya. Mujhe pata bhi nahi challa kay miscarriage hogaya tha aur phr complications hogayeein. Aur phr 3 saal baad bacha hua, aur phr innmein change aya.

**Interviewer:** acha theek hai. Aap keh raheen theen kay extremist kis tareeqay say thay woh?

**Interviewee:** shayad woh beemari kay uss phase say guzar rahay thay. Dawa nahi khaatey thay uswaqt merey saamney. Boht zyada shout kartay thay. Meiney zindagi mein na kabhi bike mein betha tha na taxi rickshaw bus waghera mein. 3 dafa hamara accident hua tha jab merey abbaya motor cycle mein agaya tha aur unko pata hee nahi challa tha. Phr jab beththay thay aur ghar say nikltay khushi khushi thay aur phr aatay ghar rotay huay. Daant partee thee. Unko sense nahi hota tha kay kahan bethay hain aur kis environment mein bethay, shuru hojatay thay, and hur cheez mein shuru hojatey thay, merey father oil company mein kaam kartay thay British Petroleum, and kehtey thay kay tumharay parents ko kaisay nahi pata, tum kya bhooj thee so phaink diya, boht saari cheezain, chahey aap ka waqt kyun hee acha nahi ajaye, magar woh time aur waqt aap kay pass kabhi nahi aata wapis aur na aap bhula saktey hain. Tu bari cheezain, Allah ka shukar hai, agay ki zindagi buss ache ho

**Interviewer:** kya aap ne kabhi doston waghera ya khandaan waghera say kabhi madad mange. Jab unka boht bura phase tha

**Interviewee:** mein ney kabhi madad nahi mangee, eik unka boht hee bura phase tha, shaadi hogaye thee aur bachay bhi 2 tay, doctor bhi boht hee jahil tha unka magar boht hee majboori bhi thee. Kay unko jab woh medicine ka overdose letey thay tu high phase mein chalay jaatey thay tu doctors unko down karne kay liye boht saarey injections laga detey thay. Aur phr yeh tablet bhi boht saari letey thay tu usskay baad jab woh uththay thay tu unko sense hee nahi hota tha kay mein kiss duniya mein hun. Hosh hee nahi hota tha tu saarey kaam beth kar kartay thay, us waqt merey bachay boht chotay thay. But mera beta ubhi tak woh baatein yaad rakhta hai kay ami aap baba ko lekey ja raheen theen aur baba aap ko mar rahay thay tu boht saari cheezain aisee saheeen hain jokay aap nahi bhula saktey.

**Interviewer:** Tu aap ki husband kay family walay aap ko iss time mein nahi support kartay thay?

**Interviewee:** kisi ko kuch nahi pata. Mujhe lagta tha kay mein he dunya mein jo iss maslay say guzar raheeh hun, halankay boht saarey log aur worse problems say guzar chukay hain. Sab sabr say, kay aane wala waqt acha hoga aur Allah deta bhi hai.

**Interviewer:** Hmm theek hai, tu aap ki zindagi kaafi mushkil raheeh hai. Tu sab say zyada aap ko inki beemari kay barey mein buri lagtee thee ya boht thaka deti thee?

**Interviewee:** waisay tu boht achay aadmi hain lekin jo addiction ka problem haina. Drug ki ho, chahe kisi ki bhi ho..kaafi cheezain theen, woh merey zindagi kay din sab say badtareen hote thay. Mein kuch bhi nahi kehsaktee thee, aur meri itni majal nahi thee kay mein inkay ghar walon say kahoon aur agar mein apne ghar walon say kehtee tu woh mujhe ghar bitha leingay aur woh koi solution nahi hai, aur mein jaantee thee kay yeh iss masoom aadmi ka koi qasoor nahi hai. Inki ma aur inki beheney inka koi bhi nahi hai aur agar mein inko chordungee tu yeh tou pagal khaaney mein jaama hojayaingay tu inka kya qasoor hai. Aur mein chup chaap kattee gaye aur Allah ka shukar hai iss muqaam mein phuncha diya hai

**Interviewer:** hmm jee tu zahir hee see baat hai kay aap kaafi madad karteen hongi unki

**Interviewee:** mein hur muqaam pe unkay saath hoti thee

**Interviewer:** tu aap ko lagta hai kay aap ka support unko madad karta tha

**Interviewee:** boht zyada! Aur inki aisee eik adat hai mein kaheen 5 minute say zyada idher say udher nahi hotee hun. Woh hur jaga meri presence chahtey hain aur mere raye chahtey hain. Yanee khud koi decision nahi letey balkey kehtay hain kay faislay karne mein tum meri madad karu. aur meiney bahir ki dunya nahi dekhee na meiney aur na mujhe business ka know-how hai lekin inkay saath reh kay meiney sab cheezain samjheen jaisay stock exchange mein kitna mushkil hota hai aur mujhe math say nafrat thee aur saari calculations waghera meiney aisay seekheein kay mujhe pata bhi nahi challa kay mein kaisay seekhtee chalee gaye aur merey khayal mein eik successful zindagi guzarne kay liye eik aurat ko hee first step lena chahye baghair apne husband ka tamasha banate huay ya apna. Aur kuch bhi nahi hosakta ub tou tu ub compromise karkay behtar zindagi guzaari jasaktee. Bajaye yeh kay taqleef mein zindagi guzaari jaye. Dunu hee soretahal mein zindagi tu guzaari jayegee hee nah. Achee baat yeh hai kay khushi kay saath guzaari jaye

**Interviewer:** hmm jee, theek. Tu aap ka khandaan waghera mein milna julna aur aana jaana waghera hai?

**Interviewee:** nahi

**Interviewer:** kya wajoohat hain?

**Interviewee:** meri family say tu bilkul cut-off hoye hain aur phr phichlay saal hum behtri ki taraf gamzan hoye tu inki family ne inko kick-off kardiya meri waja say, boht saari problems create ken tu phr hum elaidah hogaye. Mein aur merey husband jaado waghera pe believe nahi kartay lekin yeh log kaafi believe kartay hain tu usski waja say issue hua tha. Unhon ne mujh pe ilzaam laga diya kay meiney jaadoo karwadiya unkay bacho pe, unki eik beti abnormal hai, bari sab say aur mein jaado karwateen hun, unki eik bhen hain unkay betay kay andar bhi abnormality hai, tu woh meiney bhi jaado kya kyunkay inkay 3 betey thay. Tu isswaja say boht problems horahee theen aur mera boycott hogaya tha mein eik kamray mein band hogaye thee, mein aur merey husband. Na merey bachay kaheen aasaktey thay na jasaktey thay, tu phr majbooran mujhe yeh faislay lena para

**Interviewer:** kya aap log cousins waghera thay?

**Interviewee:** nahi nahi, kuch nahi

**Interviewer:** aur inki jo bheno waghera ki shaadiyan hain woh cousins mein hoye hain?

**Interviewee:** nahi

**Interviewer:** theek hai, aur aap ko pata hai hamari society mein nafsiati beemari ka kaisa ha kay log kehtey hain oh, tu log aap say phoochtay hain unki beemari kay barey mein? Kabhi agar kisi ko pata chal gaya ho tu?

**Interviewee:** mein tu misaleen deti hun kay Hitler aur itnay barey barey logo ko beemari thee, Bill Clinton ko bhee the tu phr koi maslay ki baat nahi hai

**Interviewer:** tu yeh aap ka jawab hota hai?

**Interviewee:** jee mein yehi kehtee hun. Mein kehteen hun kay beemariyan hotee hain logo ko aur ussay bhar kay aap logo ko kyun batao? Yeh cheez aisee tu nahi haina kisi ko bataye jaye, nazar tu nahi arahee na? Buss!

**Interviewer:** saheeh aur acha, agar aap ko inki beemari kay barey mein shaadi say pheley pata hota tu aap shaadi karteen?

**Interviewee:** *pause* nai kartee, sachi baat hai, jhoot kyun bolun. Kyun zindagi mushkil mein daaltee?

**Interviewer:** jee saheeh baat hai

**Interviewee:** bilkul honest baat hai.

**Interviewer:** theek hai aur aap ko jab beemari kay barey mein pheli dafa pata challa tu aap ka kya radeamal tha?

**Interviewee:** mein chup hogaye thee, aur 3 din tak bilkul chup thee aur chohtey din (4^th^ day) mein itni depressed thee kay jab mein kaam kar rahee thee aur mein..baqool merey husband kay aur inki mother kay kay mujhe fits parey thay. Beintiha mein toot hogaye thee. Mujhe waqai ussdin laga kay merey father ko Allah ne betiyan kyun deen. Agar hum itnay bhooj thay, taqleef ki baat haina kay jaantey bhoojtay aur phr position bhai aur father ki aur baghair information collect kye sirf baap kay naam pe beti ko phaink diya, zulm nahi hai kya?

**Interviewer:** acha tu aap apnay parents say cut off huwi hain tu woh kya phoochtay nahi hain kya?

**Interviewee:** nahi phiclee dafa jab pata challa tu phoocha aur meiney bataya lekin wohi baat hai kay kya karsaktein hain. Ghar mein tu nahi beth saktee na bachay lekey

**Interviewer:** haan saheeh, aap kay bachon ko pata hai aap ka eik beta tu kaafi bara hogaya hai?

**Interviewee:** kya pata hai?

**Interviewer:** inki beemari kay barey mein?

**Interviewee:** nahi nahi kabhi bhi nahi. Father…betey kay liye father ki personality ko itna strong hona chahye kay usko kuch farq hee na parey kyunke beta hur cheez father ki follow karta hai.

**Interviewer:** acha tu aap doctor waghera kay appointments mein inkay saath hur waqt aatee hain?

**Interviewee:** jee hur waqt aatee hun aur hur jaga. Buss last time nahi aye thee kyunkay bachay chotay hain aur Akeley thay aur separate hotay huay difficult hojata hai

**Interviewer:** jee jee zarur. Acha aur aap ko lagta hai kay aap ki relationship mein koi tabdeeli aye hai jab say aap ko illness kay barey mein pata challa hai?

**Interviewee:** nahi, ub hum dunu iss stage pe hain, hum dunu ki jo physical relationship hai, usko 2 ½ -3 saal hogaye hain, ussmein kaafi dafa unhon ne kaha lekin meiney mana kardiya kyunke mujhe iss cheez say shaded tar chir aate hai, bachay hogaye kaafi hai, ub zaroorat nahi hai, saaf baat. Aur kabhi kabhar ubhi bhi hota hai tu mein kehteen hunk ay abhi chordain. Aur phr woh bhi maan jaatey hain kyunke woh kehtey hain kay mein zinda laash kay saath nahi karsakta. Woh khud kehtay hain kay jab tak tum nahi aogee tu mein haath nai lagaonga. Aur meiney aap yaqeen karein, hum dunu boht behtreen dost hain. Yeh bolne say pheley mujhe pata chal jaata hai inki body language say kay kya soch rahay hain aur kya karne walay hain, dosti ka jo relationship hai, aur isskay illawa dosti ka aisa relation hai kay hum ghunto ghunto beth kay chat pe bethtay hain, chai peetay hain aur baatein kartay hain, eik eik mozoo per, boht zyada. Bacho kay saath itnay friendly hain kay aap na poochain mein tu bacho kay saath strictness kartee hun, yeh nahi kartay. Boht friendly hain, boht achay dost hain, allah ka shukar hai aur Allah bus qaim kare

**Interviewer:** acha aap ko lagta hai kay inki beemari ki waja say aap kay dusre rishto mein farq para hai? Dusre logo say jo aap kay rishtee hain?

**Interviewee:** *pause* mujhe nahi lagta. Mujhe yeh lagta hai kay yeh positive baat hai kay inhon ne family ko chorha. Tu yeh zyda behtar hogaye hain, aur jab unhon ne job say resign kya aur retirement lele tu inmein meiney dekha kay tabdeeli aye hai. Office mein tu sab ko pata tha kay woh beemari hain. Agar aap eik aadmi hain aur mein aap say kahoon kay mein psychiatrist hospital mein aye hun aur aap say phoochun kay aap ko awaazein aatee hain, aap ko woh khayalat aatey hain jo aap ko manfi lagtay hun? Tu na chahtey huay bhi aap ko lagta hai aur aap feel kartay ho. Aur aap khud ba khud feel kartay ho. Aur ghar mein hum iss barey mein baat nahi karte aur kabhi kabhar teezi wali kefiat hoti hai tu phoochtay hain kay mein taiz horaha hun tu mein kehteen hun kay mujhe tu feel nahi hua. Aap sorahay hain, aur aap saamaan nahi lakay de rahay hain tu kya teezi hai. Istarah say divert karna parta hai. Chewing gum ki tarah khainchna nahi chahye, usi waqt topic ko khatam kardena chahye. Who zyada behtar hai

**Interviewer:** aap ko lagta hai kay inki beemari ki waja say, waise tu aap ne bataya kay aap stressed out hogaye theen aur depression mein chaleengaye theen tu kya aap ne doctor ko dikhaya? Dawai waghera li?

**Interviewee:** nahi *laughs*

**Interviewer:** tu kabhi koi maali mushkilaat ka saamna karna para inki beemari ki waja say? Job pe jaatey thay?

**Interviewee:** nahi boht dafa.. buss zindagi guzar gaye, merey bhai thay jo khamooshi say hamari madad kardetey thay, paisay detey thay

**Interviewer:** tu aap kay bhaiyon ko pata tha?

**Interviewee:** nahi unko yeh pata tha kay yeh job pe nai ja rahay hain

**Interviewer:** beemari kay barey mein nahi pata tha?

**Interviewee:** nahi beemari kay baaray mein nahi meiney bataya tha lekin yeh feel kar rahay thay kay kaheen na kaheen garbar waghera hai jo maslay horahay hain

**Interviewer:** aur aap ka din guzarta hai?

**Interviewee:** hur roz mujhe bed tea dekey uthatay hain aur kam az kam du ya teen cup hote hain, aur phr mein uthtee hun tu kehtay hain kay uth kay mu haath dho lo aur mujhe nashta bana du. Warna kabhi kabhar khud bhi bana detey hain. Phr kehtey hain kay ao computer pe beth jaatey hain, stock exchange start hone wala hai.

**Interviewer:** pheley ja aap log saath rehtay thay apne in-laws kay saath tu kaisee zindagi guzartee thee?

**Interviewee:** sakht. 6 baje uthee thee aur phr unko office bethji thee aur neechay aajatee thee. Sab ka nashta waghera.

**Interviewer:** aur phr jab unka aisa phase aata tha tu aap ki zeemardian bhar jaatee hongee tu din mein kitnay ghantay aap unka dihaan rakhteen theen?

**Interviewee:** mein sarey kaam time sey pheley finish kartee the. Mein dupher ka khana subah bana leti thee. Kyunke jab woh low phase mein jaatey hain tu depression mein matlab jaatey hain tu yeh ubhi bhi bachay ki tarah hain kay mujhe attention du. Ubhi bhi mujhe kehtey hain kay bacho kay pass bethi rehti ho, merey pass kyun nahi aatee ho? Tu yeh bachay say zyada time mangtay hain. Buss kehtay hain kay mujhe time du aur kisi aur ko nahi.

**Interviewer:** aur aap ne inki beemari kay baad aap ko lagta hai koi aisee zeemedarian lelein hain jokay normally shauhar letey hain? Ya ghar ka jo aadmi hota hai woh aap kar rahi hain?

**Interviewee:** woh pheley tha, ub nahi. Ghar kay saarey kaam, drop karna, fees jama karwana, raat ko mein 12 30 am baje bhi nikli hun aur cheezain laye hun. Dekhtee thee hasrat say kay yeh kaam unkay hain aur jo father hota hai, woh bacho per strictness karta hai, dekhta hai, check and balance karta hai, aur progress leta hai, yeh responsibility father ki hotee hai tu mujhe boht bura lagta tha, lekin ub yeh sab kuch kartay hain, tutor say lekey, qari sahib say lekey, aur bacho kay kapro waghera ka mashAllah, mera bhi boht kartay hain.

**Interviewer:** aap ko lagta hai kay aap ko inki beemari kay barey mein enough knowledge hai?

**Interviewee:** mujhe? Bilkul? Net say lakay mein hur cheez search kar chunkee hun. Dr Murad say mili aur phr unka opinion lya. Farig waqt mein hum yehi kar rahay hotay hain

**Interviewer:** acha. Dr Murad ka aap ko kis ne bataya tha?

**Interviewee:** meri cousin ne

**Interviewer:** tu aap ki cousin say aap ne mention kya tha?

**Interviewee:** jee meiney apni cousin say phoocha tha aur unhon ne kaha tha kay Dr. Murad boht achay doctor hain tu …last November mein mera boht khatarnaak jhagra hua tha tu mein ghar chalee gaye thee. Unhon ne mujhe boht maara pita tha aur divorce tak baat chalee gaye thee tu uss waqt yeh kahanee uthee thee kay tum Murad Moosa ko kyun nahi dikhatee. Beeqaar jahil doctorun kay pass kyun dikha rahee ho

**Interviewer:** aur phr kaafi improvement aye?

**Interviewee:** phr Allah ka bara shukar hai

**Interviewer:** aisee kya wajoohat hain jin ki waja say aap ne shaadi ko barqarar rakha hua hai? Zahir hee see baat hai kay aap ne tough time dekha hai?

**Interviewee:** kyunkay sab say bhar kar issmein meray husband ka koi qasoor nahi hai. Woh eik chohtay masoom bachay ki tarah hain aur Allah kay illawa mein sab say zyada insay close hun apne parents say bhi nahi hun. Aur unkay saath time spend karne mein mujhe boht maza aata hai

**Interviewer:** theek hai, aur aap ko lagta hai kay aap inko saheeh karsaktee hain?

**Interviewee:** bilkul. Itnay buray phase say guzar kar itni ache jaga laya hai tu agay hee lekey jaongeee

**Interviewer:** acha aur last time jab aap ka jhagra hua tha jab divorce tak baat chalee gaye thee, tu hua kyat ha?

**Interviewee:** buss wohi hua tha kay overdose lee theen medications keen, sotay nahi thay, eik eik haftay hojata tha aur abnormality horahee thee, tu yeh feel kartay thay kay hur koi mujhe dekh raha hai aur merey khilaaf soch raha hai, mein baat kar raheen hun kisi say tu sochtay thay kay inkay khilaaf baat kar rahee hun tu boht zyada shout kartay thay. Aur bed pe nahi bethnay detey thay, light nahi off karne detey thay aur phoochtay thay kay wahan kyun ja rahi ho, yahan kyun ja rahi ho, aur phr baat chaltee chali gaye..

**Interviewer:** aur maara waghera bhi tha?

**Interviewee:** jee beintaha, aur yeh sab say khatarnaak mar pithi thee kay bacho kay saamney galay say pakar kar aur unn mein itni taqaat agaye thee kay mujh jaisee aurat ko utha kay upper tak legaye thay. Buss eik dafa…aur phr ussay pheley bhi chal raha tha, eik meheny tak aisa silsila challa tha. Mein keh rahee thee kay theek hojayeinge, theek hojayeinge, unhon ne mujhe hanger say maara tha, belt say maara tha, tu mujhe boht taqleef huwi thi jab first time belt say maara tha tu mein boht royee thee. Magar aisa roye thee kay uskay baad bekhabar soyee thee. Pitnay kay baad aur yeh chaley gaye thay ghar say tu mein bekhabar soye thee, mein diary likhtee hun tu mein yahee soch rahee thee kay mein itnay sukoon say tu kabhi nahi soye, jab itnay pitnay kay baad tu mein sukoon say sogaye tu meiney socha kay pitnay mein bari taqat hoti hai. Khair boht cheezain hotee hain

**Interviewer:** tu phr aap ami abbu kay ghar chale gayee theen?

**Interviewee:** jee wahan mein eik hafta rahi thee

**Interviewer:** tu phr talaaq ka aap ne socha tha?

**Interviewee:** haan merey parents ne kaa

**Interviewer:** lekin phr?

**Interviewee:** merey saath merey 3 baje thay aur mein kaisay bharoosa karsaktee thee. Jab karnay ka time tha uss time kuch nahi kya, tu ubh kaisay karsakte thay, aur ache baat hain nahi kya

**Interviewer:** acha aur aap ko kya lagta hai kay eik shaadi shuda joray kay beech mein jo rishta hota hai woh zyada important hota hai ya puri family zyada ahmiat rakhtee hai?

**Interviewee:** aap ka husband aur aap kay beech mein jo rishta hota hai wohi sab say zyada ahmiat rakhta hai.

**Interviewer:** aur aap kay kya khayal mein eik shaadi shuda joray ko kis soretahal mein talaaq leni chaye hai?

**Interviewee:** kamazkam jab bachay hun tu nahi leni chahye, agar bachay nahi hun tu soch lena chahye. Lekin agar bachay hun tu nahi lena chahye aur agar aurat ho tu kabhi nahi karni chahye

**Interviewer:** aur aap ko kya lagta hai kay eik purskoon khandaan ko parwarish karne kay liye kis cheez ki zaroorat hoti hai?

**Interviewee:** Compromise

**Interviewer:** acha aur aap ne marital counseling kay barey mein suna hai?

**Interviewee:** jee shaadi kay foran baad counseling ki thee eik khatoon ne. uskay baad say meiney kuch nahi kya

**Interviewer:** acha aur aap ko lagta hai kay agar mental illness problem ho tu help karsaktee hai?

**Interviewee:** Bilkul. Jab hamaray say intercourse nahi horaha tha tu bara pareshaan thay aur mein tu kehtee thee kay merey qareeb na ao.

**Interviewer:** acha merey sawaal complete hogaye hain tu aap ko koi sawal karna hai?

**Interviewee:** nahi buss mujhe yeh kehna hai kay yeh waqt larki kay liye boht mushkil hota hai aur yeh golden period hota hai yeh palat kay wapis nahi aata. Buss wohi baat hai kay parents ko dekh kay sab kuch karna chahye hai, boht dekh kay. Especially betion kay liye

***Interview Ends***
