# Supplementary material for: Exploring effects of severe mental illnesses on marriages: A qualitative study from Karachi, Pakistan
Source: PLOS Glob Public Health. 2025 Dec 23;5(12):e0005652. doi: 10.1371/journal.pgph.0005652 (PMC12725543; doi:10.1371/journal.pgph.0005652)
Supplement: S1 Data — (ZIP) [file pgph.0005652.s001.zip › Transcriptions/Case 2-6 Transcripts/Case 5/C5-1.docx]

**Case 6**

**Psychiatric Illness: Bipolar Disorder**

*fills out the demographic questionnaire*

**Interviewer:** How long were you married for?

**Interviewee:** since 13 years

**Interviewer:** Do you have any children?

**Interviewee:** I have two boys. 11 and 8.

**Interviewer:** Which year did you get married?

**Interviewee:** 2003

**Interviewer:** Divorced?

**Interviewee:** This year, 2015

**Interviewer:** So you have been under treatment since?

**Interviewee:** Since I was 18 years old

**Interviewer:** Is that when you got diagnosed?

**Interviewee:** Yes

**Interviewer:** Okay so. And when you got married, did your wife know about the illness?

**Interviewee:** Yes. I briefed her in full detail about the disease.

(the mother also added by saying that the doctors said that there is no problem and one should go ahead with the marriage)

**Interviewer:** Okay. So she knew about it?

**Interviewee:** Yes very well.

**Interviewer:** So you sought help from a psychiatrist immediately?

**Interviewee:** Yes

**Interviewer:** Have you ever been admitted in a hospital?

**Interviewee:** Many times.

**Interviewer:** So you got divorced this year?

**Interviewee:** Yes

**Interviewer:** and I mean, what were some of the reasons?

**Interviewee:** We did not get along well with each other.

**Interviewer:** all right, was it always this way?

**Interviewee:** No it was not always this way. We were very much in love when we got married.

**Interviewer:** Okay

**Interviewee:** I don’t know…I still l--- I can’t say that I still love her but I did love her until recently. And I think my disease finally got to the point where it became too much for her to handle.

**Interviewer:** Okay did anything in specific happen this year?

**Interviewee:** Not anything specific but a lot of incidents. Sporadic incidents. They kept happening. But it was just a matter of stone in the camel’s back.

**Interviewer:** So things kept on building up basically.

**Interviewee:** Yeah yeah basically

**Interviewer:** So during the time when you were married, were there periods of normality?

**Interviewee:** Yes there were many periods

Mother states: last year by chance, he was diagnosed with something else. And it was another disease or condition, which made his condition severe. And that became very difficult for everyone to handle.

**Interviewer:** okay what kind of a change in the condition?

**Interviewee:** I started having manic symptoms and episodes.

**Interviewer:** First you were just on antidepressants?

**Interviewee:** yes. And then I started showing manic symptoms and therefore, my aggression increased and her patience decreased. And that’s a lethal combination.

**Interviewer:** Okay did her parents know about the illness?

**Interviewee:** Yes yes

**Interviewer:** All right. Okay you were 18 years old when you were first diagnosed?

**Interviewee:** Yes.

**Interviewer:** Okay, so when you were first diagnosed, what was your first reaction to the illness? I mean if you remember, it has been a long time.

**Interviewee:** I mean I remember I was very depressed. I mean I was bed-ridden. I had a breakup with my girlfriend and I was very sad. I was very down. I was very low. And then I was taken to a child psychiatrist and he diagnosed me. And he put me on anti-depressants. And I remember feeling very sad and very upset. It was a very tough time

**Interviewer:** and your manic symptoms started sometime last year?

**Interviewee:** Yes two years ago actually

**Interviewer:** And what were the manic symptoms like?

**Interviewee:** anger, rage, fury

**Interviewer:** Did you ever shout at her?

**Interviewee:** Shout. Hit. Push. Shove. Swear. Abuse. Scream. Yell. Everything

**Interviewer:** Even towards the children?

**Interviewee:** No just her

**Interviewer:** Okay and what was her reaction to the illness?

**Interviewee:** She took it. As long as she could. And then she could not bear it anymore and then she left.

**Interviewer:** Okay and what was the other family member’s reaction to the illness?

**Interviewee:** Hers

**Interviewer:** Hers and yours both

**Interviewee:** My family has been very supportive. My mother has been a pillar of support. My father has been very supportive, as well. And even my wife’s family wanted to keep the marriage more than anything else

**Interviewer:** Okay they were?

**Interviewee:** Yes

**Interviewer:** They were against it?

**Interviewee:** Yes they are very good people. They were against it.

**Interviewer:** Okay and what was the children’s reaction to the divorce?

**Interviewee:** Relief

**Interviewer:** They were relieved?

**Interviewee:** Yes

**Interviewer:** are they with you?

**Interviewee:** No they are with my wife. I tried for custody but courts in Pakistan don’t give custody to fathers. So I get to see them on weekends. Every Saturday and Sunday. I have them for two days.

**Interviewer:** Okay. And do you have any kind of support from your family in terms of emotional support. Your mother obviously

**Interviewee:** Immense. My mother and father..I have amazing support

**Interviewer:** Okay do you have any sisters or brothers..siblings?

**Interviewee:** No I have one brother but we are not close

**Interviewer:** So as a couple, did you and your wife use to go and socialize?

**Interviewee:** All the time. We had a great social circle. We loved entertaining and we used to have friends over and we used to go out and we used to meet friends. We used to go to parties, hoteling, cinemas, dining, restaurants. You name it. There was no night we spent at home

**Interviewer:** And how was it like socializing as a couple?

**Interviewee:** Brilliant. We loved it

**Interviewer:** Okay and did people question her about your illness? Especially after your manic symptoms had started?

**Interviewee:** No no my friends had known this since my childhood.

Mother: Actually even some of his friends are bipolar *laughs* they don’t take medicine.

**Interviewee:** They all knew about it from the start, so they didn’t really question her about it.

**Interviewer:** Also you got diagnosed during school so they must have known about it

**Interviewee:** Yes.

**Interviewer:** Okay and do you think your family dynamics have changed after your divorce?

**Interviewee:** Immensely.

**Interviewer:** In what ways?

**Interviewee:** In the sense that I have lost my children. I come home to an empty house. It’s just my parents. I used to come home to a family and now they are not there anymore. I come home to an empty room and I sleep in an empty bed. And umm. My wife was the very fabric of my life and now it is shattered

**Interviewer:** Okay was it her idea for divorce?

**Interviewee:** Yes she wanted khula.

**Interviewer:** You didn’t want it?

**Interviewee:** No, I fought it till the very end

**Interviewer:** Okay. Did your children know about your illness?

**Interviewee:** Yes they do

**Interviewer:** Do they also know why the divorce took place?

**Interviewee:** Yes they do.

**Interviewer:** They are old?

**Interviewee:** Yes

**Interviewer:** Do you feel that your illness led to any mental health problems for your wife?

**Interviewee:** Yes I am sure. She sought therapy

**Interviewer:** She sought therapy?

**Interviewee:** Yes

**Interviewer:** For depression

**Interviewee:** For multiple reasons

**Interviewer:** For stress?

**Interviewee:** For stress and depression. Relief from the environment. Dealing with anxiety.

**Interviewer:** All right. How was the day like for her?

**Interviewee:** She works. So she keeps busy

**Interviewer:** All right

**Interviewee:** She loves her work. It keeps her active

**Interviewer:** Did she take a lot of time taking care of you?

**Interviewee:** No

**Interviewer:** Did she take any responsibility especially during your manic symptoms?

**Interviewee:** Um. A little

**Interviewer:** What kind of responsibility?

**Interviewee:** She used to manage my medication

**Interviewer:** You weren’t compliant with your medications?

**Interviewee:** I am compliant. I am very compliant.

**Interviewer:** But during your manic phase?

**Interviewee:** Yes I wasn’t

**Interviewer:** Okay and what do you think are some of the personal reasons for the divorce? Your illness?

**Interviewee:** I am not a nice person

**Interviewer:** You’re not a nice person?

**Interviewee:** Okay. I….*pause* I let her down. I disappointed her. I broke her heart. I did not offer the love and security and the comfort which a spouse and a companion should. I failed at marriage. It’s as simple as that. If there is anyone to blame for the breakdown of the marriage, it is me. It is simple

**Interviewer:** All right. Do you feel you could have saved your marriage?

**Interviewee:** I tried my best.

**Interviewer:** You fought until the end?

**Interviewee:** Right uptill the end

**Interviewer:** Okay, so in your opinion, why do you think the illness occurred to you?

**Interviewee:** It’s genetic.

**Interviewer:** So you’re pretty clear on that?

**Interviewee:** Yes yes

**Interviewer:** Because you know there are all these reasons in the society

**Interviewee:** of course

**Interviewer:** Do you feel that you could have eased your spouse’s burden at any point in time?

**Interviewee:** Yeah I could have but I didn’t

**Interviewer:** Were there any kind of financial issues during the marriage?

**Interviewee:** Sometimes yes

**Interviewer:** So she managed to take care of that?

**Interviewee:** No, I kept that end of the weight myself. Financial burdens were there, but they were on me and not on her. And I never let her feel the crunch of it.

**Interviewer:** Okay and when your bipolar struck last year, you were still going to work?

**Interviewee:** I have been going to work consistently

**Interviewer:** okay that’s a good thing.

What did your spouse say when someone asked of your illness?

**Interviewee:** They would not know about it so she didn’t have to say anything and those who did know about it, she didn’t have to explain it to them. It was not like she had to say anything. We kept it quiet. It wasn’t a public thing. You don’t tell people about this but yeah that’s it

**Interviewer:** Okay was it an arranged marriage?

**Interviewee:** No we fell in love. We were very much in love.

**Interviewer:** All right. And in your opinion, when do you think a couple should seek divorce?

**Interviewee:** Never

**Interviewer:** All right. Why do you think, despite your partner being aware of your mental illness, why did the divorce happen?

**Interviewee:** Between theory and reality, things are very different. She knew the theory of it and when reality hit, she couldn’t handle it

**Interviewer:** Do you think people can handle it?

**Interviewee:** I am sure there are people who are more tolerant than others. So yes

**Interviewer:** All right. Was it suggested to you by anyone to seek divorce?

**Interviewee:** No

**Interviewer:** Did you guys take up marital counseling?

**Interviewee:** Yes

**Interviewer:** Did it work?

**Interviewee:** No

**Interviewer:** Not at all?

**Interviewee:** No

**Interviewer:** You did it here only. At AKU?

**Interviewee:** Yes

**Mother:** The counselor told her that run as fast as you can.

**Interviewee:** No she didn’t

**Mother:** Yes she did

**Interviewee:** She had her own counselor as well. And we tried. We did therapy. We did family therapy. Exercises. All of it. But it all clearly failed

**Interviewer:** Okay and what do you think are some of the essential building blocks for raising a family?

**Interviewee:** that word in itself, healthy. This is the essential building block. If you don’t have that, then there is no point

**Interviewer:** Okay. I am done with my questions. Do you have anything to add?

**Interviewee:** I am getting married again. On 5^th^ of December and I realize now that ummm…

**Mother:** He is lonely

**Interviewee:** I need a companion. I need to move on.

**Interviewer:** Do you know her from before?

**Interviewee:** Yes. She is actually my cousin.

**Interviewer:** She knows about the illness?

**Interviewee:** Yes she is very well aware of the illness. And I want to move on with the rest of my life. I don’t want to fixate on my ex wife and just linger there. I have to move on

**Interviewer:** Yes that’s a very practical approach.

I just have a couple of questions. Umm, sorry I am asking this when your mother is here. But how was your sexual life?

**Interviewee:** Amazing. Sex life was amazing

**Interviewer:** It wasn’t affected? During your manic phase?

**Interviewee:**  Sometimes, it was affected. Premature ejaculation or delayed orgasm. That was there but other than that, it was amazing and if anything, if a good sex life is able to put together a marriage, then there would not have been any problems

**Interviewer:** Did you guys fight before the bipolar struck?

**Interviewee:** Yes we did

**Interviewer:** And do you think it was because of the illness?

**Interviewee:** I think it had a lot to do with how I was as a person.

**Interviewer:** What makes you say that?

**Interviewee:** I have a personality disorder other than bipolar disorder

**Interviewer:** Which is diagnosed?

**Interviewee:** Which is diagnosed. Which is..I have mood fluctuations

**Interviewer:** Borderline?

**Interviewee:** I have mood fluctuations. I go from being a nice person to being an extremely vicious one. I can be your best friend or your worst enemy. I have an extremist personality and that does not sit very well with most people.

**Interviewer:** Yeah. But she knew about it?

**Interviewee:** Yes

**Interviewer:** Did you guys date?

**Interviewee:** Yes we dated for quite some time

**Interviewer:** Okay so you’re getting married again. Do your kids know about it?

**Interviewee:** Yeah

**Interviewer:** And what do they feel about it?

**Interviewee:** They have mixed emotions. I am still trying to work it out with them. They understand the concept of a wife. They don’t like it very much. I am trying to explain the concept of a companion but it is hard when they are 11 or 8 years old

**Mother:** They say why don’t you keep a cat?

*laughter all around*

**Interviewer:** Okay and is your ex-wife planning to get married?

**Interviewee:** No not that I know of, but somewhere down the road, yes she might. And I will not begrudge her for it.

**Interviewer:** Of course. Okay, thank you so much. Do you have anything to ask us?

**Interviewee:** Not really. Cheers.

***Interview Ends***
