# Supplementary material for: Exploring effects of severe mental illnesses on marriages: A qualitative study from Karachi, Pakistan
Source: PLOS Glob Public Health. 2025 Dec 23;5(12):e0005652. doi: 10.1371/journal.pgph.0005652 (PMC12725543; doi:10.1371/journal.pgph.0005652)
Supplement: S1 Data — (ZIP) [file pgph.0005652.s001.zip › Transcriptions/Case 2-6 Transcripts/Case 5/C5-2.docx]

**Case 5**

**Psychiatric Illness: Bipolar Disorder**

Patient did not allow the interview to be recorded.

Maheen was diagnosed with depression when she was 18 years old, after she had finished her A levels. She did her A levels privately and then she proceeded to college, but later dropped out because of her depressive phase. She was seeking treatment under Dr. Eshan at AKU before. She got married when she was 24 years old and right now, she is 38 years old. Her marriage last for 1 year and 2 months. Her parents had informed her ex in-laws that she suffered from depression, and they seemed to be completely okay with it. However, after marriage, their attitude changed, especially because the psychiatrist informed her ex in-laws that she had bipolar disorder. When they realized that it was not depression, but bipolar disorder and after searching on the Internet about this disorder, they did not like it. Maheen mentions that she didn’t like the fact that the psychiatrist initially did not tell her of the diagnosis and told her ex in-laws. Her father in law had passed away, but her mother in law was alive and knew about the illness fully. Her parents of course knew about the illness. When asked about her reaction towards the illness, she mentioned that she already knew something was wrong with her, so she did not have any reaction. She knew that there was some problem with her and the doctor just needed to confirm. She mentioned that she had thyroid issue which was not diagnosed initially, which led to her depression. When asked about her husband’s reaction to the illness, she mentioned that he knew about it since the beginning so there was no reaction as such.

Asking about her support system, she says *“my siblings were very accommodating. My father was also quite supportive. However, my mother I have issues with because she also has some psychiatric illness I believe. She is also upset because all her three children are divorced. My brother and sister are also divorced”*. When asked about the support system after divorce, she mentions that everyone was quite okay with it because *they wanted my happiness.* She has friends, with whom she talks to about her problems, as well.

When asked about her life during marriage, she said that her mother in law made her life quite difficult. She always used to come along with them whenever the couple went out, which made it difficult to bond with her husband. She never went alone with him apart from a few weddings.

She mentions that her family is quite liberal, since her father was abroad for a while. When asked whether she found it hard to adjust in her in-laws especially because of the illness, she said that she didn’t find it hard to adjust. Her mother in law became possessive and she made it bothersome. She didn’t get any space. She also spoke about the fact that there were people who said that she adjusted so well that it was not expected since her ex in-laws were quite different.

When asked about care-giver’s burden, she said that her husband took great care of her and supported her a lot. He was very helpful and was very gentle with her. They hardly had any fights. Therefore, I questioned her as to what led to divorce, and she said that it was entirely because of her mother in law, because she created so many issues. Her husband then said that *I have the choice to be on your side or on your mother’s side, so he found it easier to be on his mother’s side, and this is why I have to give divorce’.*

She also said that her husband did not take any additional responsibilities and she was teaching at that time, and was quite stable. Only her mother in law made it difficult for her.

When asked about the reasons for the divorce, she said that her illness was used as an excuse; her mother in law could not bear her son being so close to her, which is why she created so many issues.

She feels that she could have saved her marriage for sure, but it would not have lasted any longer because the mother in law would have made it difficult anyway. She also mentioned that her mother in law passed away three months after her divorce, because she could not see the state that her son had put himself in after the divorce. Maheen said that she heard that her ex husband had not done well following divorce.

She also said that the illness occurred to her primarily because of thyroid. She also said that her in-laws knew about the illness and were good about it apart from her mother in law.

She said that a couple should only seek divorce when one of them is cheating on the other. And she said that the divorce was his idea, and she did not want divorce at all. But it would have broken down anyway because it was too late and things got out of proportion and there was no time. In fact, her mother in law’s sister told her that she can save the marriage, but it was too late.

When asked as to what led to her marriage breaking down, despite prior knowledge of the illness, she said that the problem was not the illness but was used as an excuse to break it off.

To the question about marital counseling and whether couples should seek it, she said *I don’t know, I am divorced.*
