# Supplementary material for: Exploring effects of severe mental illnesses on marriages: A qualitative study from Karachi, Pakistan
Source: PLOS Glob Public Health. 2025 Dec 23;5(12):e0005652. doi: 10.1371/journal.pgph.0005652 (PMC12725543; doi:10.1371/journal.pgph.0005652)
Supplement: S1 Data — (ZIP) [file pgph.0005652.s001.zip › Transcriptions/Case 2-6 Transcripts/Case 5/C5-3.docx]

**Case 5**

**January 2015**

**Psychiatric Illness: Psychosis/ schizophrenia**

**Interviewer:** How old are you?

**Interviewee:** I am 41

**Interviewer:** Theek hai and your education?

**Interviewee:** I have a Bachelors in Economics and Finance and I am currently training to be a therapist

**Interviewer:** are you currently working anywhere?

**Interviewee:** No

**Interviewer:** Were you working previously?

**Interviewee:** No never

**Interviewer:** When did you acquire your education?

**Interviewee:** 1996 I guess. I have been doing stuff for myself but never formally though. I would like to work though

**Interviewer:** and what was the duration of your marriage?

**Interviewee:** *inaudible*

**Interviewer:** Currently where do you live? Do you live on your own?

**Interviewee:** With my other.

**Interviewer:** So it is the two of you?

**Interviewee:** Yes the two of us

**Interviewer:** If you don’t mind me asking, in one month, how much is your income?

**Interviewee:** Per month… *pause* through stocks?

**Interviewer:** Everything

**Interviewee:** I think around 2-3 lakhs

**Interviewer:** and do you have any kids?

**Interviewee:** No

**Interviewer:** What was your ex-husband’s education?

**Interviewee:** He did his masters in Finance.

**Interviewer:** And what was your father’s education?

**Interviewee:** He was a very good banker

**Interviewer:** And in your family, is there any psychiatric history in your family?

**Interviewee:** Yes father.

**Interviewer:** And anyone else?

**Interviewee:** Father’s side basically yes. Mother too at times

**Interviewer:** Since how long have you been ill?

**Interviewee:** I think I have this problem since I was 19 years old or 20..back when I was in college, I had a few episodes. And due to..it was awful. I did go to doctors. And that kind of made me freak out and other than that, not really. And I tend to be doing better now.

**Interviewer:** Okay have you had episodes where you have been out of control and someone had to take care of you? How many such episodes have you had?

**Interviewee:** Once, but that was because of my mother. And then I went to a therapist. And then I had to be injected because I was very much out of control. Instead of talking, I was injected. I am scared of the repercussions now. Now I have started painting to control my symptoms

**Interviewer:** Right. You paint?

**Interviewee:** Yes

**Interviewer:** Okay and how many such episodes have you had?

**Interviewee:** Then after my divorce, I went to a counselor.

**Interviewer:** So how long have you been on medications?

**Interviewee:** See my mother started medicating me without my knowledge. I don’t know about the names of the medication. I will find out in case the side effects could be due to that- the psychosis- I never had it. The hallucinations etc. So I have taken a lot of medications

**Interviewer:** how old were you back then?

**Interviewee:** I was 28 or 29 and then I was on Seriquel ..you name the pill, I have had it. I had a cocktail. None that suited me. But now I am better. I don’t like psychiatry much

**Interviewer:** We also are psychologists in the making?

**Interviewee:** psychologist or psychiatrist?

**Interviewer:** Psychologists

**Interviewee:** Okay okay. I have a strange aversion to psychiatrists

**Interviewer:** Because of medication

**Interviewee:** Yes because of medication and I have been to so many but who have never understood the core condition. So I said mein khud hee Seriquel band kardete hun

**Interviewer:** Have you ever sought help from anyone else apart from a psychiatrist because there are people who go to faith healers?

**Interviewee:** Yes I am going to a homeopath and I have to tell that to Murad. And I also have a spiritual healer because woh tou karna hota hai na.

**Interviewer:** And hakeem or anyone?

**Interviewee:** Homeopath now

**Interviewer:** Do you do any drugs?

**Interviewee:** Nothing now. Chai, caffeine. I have started nutrition. Caffeine can lead to schizophrenia.

**Interviewer:** We have so much chai. It is an addiction.

**Interviewee:** The book dance of something in which a schizophrenic patient gets addicted to caffeine.

**Interviewer:** So do you have financial problems or health problems?

**Interviewee:** No my mother was controlling my finances for a long time. She thought I would spend it but she was being presumptuous but now that I am 40 – 41 years old, I don’t feel the need to overspend. My desires have decreased significantly. I mean kisi ka dil khush karne ko kardiya

**Interviewer:** Otherwise, do you have any financial difficulties?

**Interviewee:** No, not at all

**Interviewer:** Any other health problems?

**Interviewee:** No

**Interviewer:** Do you have problems in relationship with other family members?

**Interviewee:** I feel that… It is because of the family.. the core sanity is family. Unki baatein sunkay deewana hoye hain, so I am trying to develop a more inner you know..peace so I do have issues with my family. They interfere too much I think especially with medicating me without my knowledge etc. But now I am not blaming anyone so now I am more in charge of myself

**Interviewer:** there are a few more questions. Alright, when were you diagnosed with your current problem?

**Interviewee:** See the previous diagnosis I don’t consider correct. I consider Murad Moosa’s diagnosis to be correct. So it should be about 3-4 years

**Interviewer:** and when did you separate from your husband?

**Interviewee:** around that time, divorce

**Interviewer:** When did you get divorced then?

**Interviewee:** I think I got divorced in around 2010

**Interviewer:** okay when did you start seeing Dr. Murad?

**Interviewee:** I came back to Karachi and I started seeing him. And I was pretty annoyed with my mother when she made me go to Dr. Shaheen. But what I didn’t like about her was that whatever I talked to her about, it was reported to my mother and it was a complete freak house. Anything I did at home, my mother used to call Dr Shaheen that usne yeh kardiya waghera waghera.

**Interviewer:** Hmm okay. When did the actual symptoms start that your mother got concerned and took you to the psychiatrist?

**Interviewee:** There were no symptoms. All of these symptoms are because of medication that they put me on.

**Interviewer:** When did you feel that you should go to a psychiatrist like you came to Dr. Murad?

**Interviewee:** When I was given anti-depressants and during my divorce time, I was very anxious. My whole life fell apart when my divorce happened. And then I think that’s when I came but now I am very functional. Because ultimately because I wanted to get better

**Interviewer:** How many years after your marriage did you get divorced?

**Interviewee:** This was during my divorce

**Interviewer:** No , how long were you married for?

**Interviewee:** 7-8 years

**Interviewer:** alright, did your spouse parents know about the problem?

**Interviewee:** You know *pause* see I had clearly told my ex-husband that I was going to a psychiatrist. He didn’t want to find out what the condition was. What the symptoms were. He didn’t want to know. Shaadi tou karnee hai, and then he said that oh your mother lied to me and you guys kept it a secret. So that was like a blame on me

**Interviewer:** So you had told your husband that you had some issues?

**Interviewee:** Yes, I told him everything. And he was also taking Ritalin

**Interviewer:** okay so he had ADHD?

**Interviewee:** He claimed to have ADD. I tried to get him off Ritalin because in America, Ritalin just is over prescribed. I was off the pills at that time and I was going to a clinical psychologist. And I was not doing well overall, with my drinking habit, smoking habit, nutrition. And I need to look after myself. Ultimately it is me before my mother even though I am supposed to take care of my mother but you see, these people have caused me so much grief.

**Interviewer:** So when you told her ex-husband about the illness what was his reaction?

**Interviewee:** Oh he said that you should go back. You have a temper and a mouth that doesn’t stop. Go back on the medication you were on

**Interviewer:** Was it a marriage of choice or an arranged marriage?

**Interviewee:** Marriage of choice because I had told Allah mian that the next person who asks me to get married, I would say yes, so when he did I said yes. So whatever it was, choice or anything. Some good came out of it. I got American passport. I learned nutrition and whole health. Let’s see where the road takes me now

**Interviewer:** and what was your family member’s reaction to the illness?

**Interviewee:** My mother carried a lot of shame because my father was bipolar. She created issues for him, and she never fully accepted it. She medicated me. She has a lot of ugly paintings in the house and what I have learned and studied is that empowerment is very important. Emotional, spiritual and physical environment. So the environment is suffocating in my house. There are a lot of nanga nanga paintings in my house. Kaali shakal kay log. Mujhe ghabrahat hotee thee. Yeh sab ubh mujhse nahi hota. Mein garden mein bethee hun, phool hotay hain aur mein apnee painting banatee hun.

**Interviewer:** Hmm. Alright, I remember that your mother forced you to get separated?

**Interviewee:** No he was creating a lot of issues so my mother, my older sister and they all said that chorou, chorou. I used to speak ill of my husband and that was my foolishness. And they all capitalized on me and said chorou, and buss. And I do hold a grudge against them and I have learned a lesson for my future relationships. It is about me and not about the others.

**Interviewer:** Okay and what ill did you speak of your husband? Did he treat you badly?

**Interviewee:** Yes there was a lot of verbal and physical hurt. Emotional abuse and other problems

**Interviewer:** okay and did he ever say to you that oh you have a problem?

**Interviewee:** He kept on saying that I will divorce you. He never gave me the stability I needed and he would leave the house for one month, and he would treat me badly. And it took me at least three years to realize that this marriage is not going to work. Partly it was my fault, as well. And I wanted to divorce him. He created issues with my passport as well.

**Interviewer:** But what I mean is that oh you have a problem and you have a mental illness

**Interviewee:** Of course that was his trump card.

**Interviewer:** Did you ever feel that he never tried to understand what the actual problem is?

**Interviewee:** You know woh eik shair haina unhon ne chahat kay bajaye baat chahee, hum ne saalam kya aur izzajat chahye. Tou aisaa hee kuch tha, He would never be happy with me. If I would put on weight, he would call me fat. And he said that he would not want to be seen with me in public. So you know mean things. I was just his trophy wife to just play the role and I was sick of it. Baal banay huay tou humeisha, kapray unkay skirt phen liya tou Pakistan woman banu, tou wahan mera dimag saheeh nahi chal raha tha. You know a lot of control and ultimately, you know, you cannot change a man. I don’t think I had the will or desire to change anyone but myself

**Interviewer:** and did you have any kind of support especially when you had support or you know when you couldn’t take care of yourself?

**Interviewee:** Yes I had a friend who was an alcoholic and he was very nice to me. He would take care of me

**Interviewer:** Here or back there?

**Interviewee:** In America. I saw very people. I lost a lot of ummm… *pause* faith in superficial acts. I don’t like it.

**Interviewer:** Alright, so now we are going to ask you about the stigma regarding mental illnesses in society. So did you ever socialize with your ex-husband? Were you ever scared that people would turn around and say what is wrong with her or was he hesitant in taking you out?

**Interviewee:** Yeah he never wanted to be seen with me in public.

**Interviewer:** Hmm okay and how was it socializing as a couple?

**Interviewee:** Oh he used to keep saying that it would never work for us to be seen together in public. I don’t know..ghar mein kon bethay ga. I used to go out myself.

**Interviewer:** Over here, in Pakistan, do you like going out? Do you feel people are constantly on the watch? Do you feel stigma over here?

**Interviewee:** Yeah. The people that I meet…see in America or anywhere in the world, I was meeting a lot of people, who were into drugs or alcohol. So my mother used to say that can you not find any normal people to talk to? Or normal people to meet? That kind of resonated with me. Now I am selective about who I hang out with

**Interviewer:** SO over here, when you meet people? Do you think they understand you?

**Interviewee:** hmmm.

**Interviewer:** Kind of relate to what you’re saying?

**Interviewee:** You know koi saath nahi hota when you’re down. I have no interest in what people think. I have found a high road. I only offer what I can offer. I have no desire to do more than it is required. I don’t belong in this world anymore. I mean I can play the role, I can dress up and smile, but I cannot enjoy there truly. And that is the society I am generally a part of where I am …. I do go out, lekin mujhe khokla lagta hai. Like last night, I went to a friend’s dinner, and everybody was drinking and smoking. I was just have a Diet coke and I belong there. I dressed up I got a lot of compliments but right now I want to focus on me and start on some work. And not do things like that.

**Interviewer:** Makes sense. And do you feel that you know the fact that you had a diagnosis, did you feel that your ex-spouse’s mental health was affected in any way?

**Interviewee:** Yes. He was traumatized. And I was very abused

**Interviewer:** Was he abusive? Was he the one who started?

**Interviewee:** Yes and then I started abusing him back

**Interviewer:** Do you feel that he started it?

**Interviewee:** He was very messed up in himself. He used to self-medicating and he used to take Ritalin just like that

**Interviewer:** Alright, so do you feel because of your diagnosis, do you feel that because of that, he had to take any additional responsibilities?

**Interviewee:** I was very baby-ied all my life and in the sense I was not working and he wanted to start a family and I didn’t want children and I used to go out and what not thinking he would put up with my moods. But nobody does.

**Interviewer:** Alright

**Interviewee:** It was unfair on him, as well. And I do owe him an apology. I do take the blame for the marriage ending. There is a lot of guilt in me and sadness in me that probably I did not behave the way I was meant to behave.

**Interviewer:** and was it his decision to take the divorce or was it yours?

**Interviewee:** He kept on threatening me and one day, I told him that give it

**Interviewer:** Do you ever feel that some of the things that you were doing were because of the fact that they were not in your control and you didn’t mean to do it intentionally?

**Interviewee:** No no I was very childish enough to believe that he would put up with it. I mean I can blame it on a jinn and get away it. I can blame it on anyone. But I have to take full responsibility of it.

**Interviewer:** Previously, you did not have enough control, or you did not have enough insight that you did things that you really couldn’t control?

**Interviewee:** *pause* I could have controlled my lifestyle and I could have controlled my temper. And unresolved tantrum issues and unconsidered anger. And I couldn’t understand what was happening to me. Ghar ka ghussa tha na woh sub udher jakey nikala

**Interviewer:** So do you ever feel that you did not get adequate help like you’re happy right now with Dr. Murad’s help but previously?

**Interviewee:** Well, she was different in America. She told me to stop talking to my family and to my friends and to go on a mountain and do yoga, and I did that. I quit smoking and I was.., part of it was different. There was a lot of chanting so I couldn’t do it. And I came back and I started doing it.

**Interviewer:** And what do you think were some of the personal reasons behind your divorce?

**Interviewee:** It was my fault. Immaturity and arrogance on mine and on his part, it would be ….*pause* no idea.

**Interviewer:** DO you think it was your fault?

**Interviewee:** You know my Sufi teacher told me that it’s a tendency to blame yourself, but taali dunu haath say baajtee and it’s time that you stop blaming yourself. So I am trying to do that

**Interviewer:** And what do you think were some of the social reasons behind your divorce?

**Interviewee:** Yeah my mother perhaps. Because she really pushed me to take divorce. I should have been more of a grown-up. And I was not doing well financially over there in America. I should have worked because we were in a rented house and had a rented car. Pata nahi.

**Interviewer:** Did your ex-husband approach you through family or friends?

**Interviewee:** Friends

**Interviewer:** Okay didn’t your family approve of him?

**Interviewee:** No they thought and said that oh he is too stiff for you. Pata nahi ….. Backgrounds were different

**Interviewer:** The guy lived in America?

**Interviewee:** Initially in Pakistan

**Interviewer:** Do you feel that you could have saved your marriage?

**Interviewee:** Yes with the right attitude

**Interviewer:** and if you could have gotten the help that you’re getting from Dr. Murad right now, if you would have gotten it there would it have helped with your anger issues there?

**Interviewee:** *pause* perhaps

**Interviewer:** and do you feel that it was because of your anger issues which is basically a symptom of your illness….

**Interviewee:** It was because of child abuse, physical abuse and I need to solve that. It’s because of childhood, I could never accept myself because of my mother since she is a dominant personality. It started manifesting in psychosis and I would talk to myself. As a child I used to mutter to myself and my mother would scream at me and I could never say anything to her so it came to the point where I started muttering. So because I couldn’t say it out aloud and when I grew up, when I used to get angry, I would lash out. But I need to be more calm now.

**Interviewer:** so you feel from what I could gather is that your physical abuse as a child by your mother and her dominating and over powering attitude led to this illness to a certain extent?

**Interviewee:** Yes

**Interviewer:** and what do you think are some of the reasons behind your illness?

**Interviewee:** My lack of self-care. My lack of self-esteem

**Interviewer:** do you feel that it could be genetic in any way?

**Interviewee:** Perhaps.

**Interviewer:** Also did you feel that your self-esteem issues could be because of your illness rather than the other way around?

**Interviewee:** The chicken or the egg? I don’t know.

**Interviewer:** We are asking too many questions. Was your spouse ever asked about the illness in any way? Like when you guys were together obviously?

**Interviewee:** *pause* I don’t know.

**Interviewer:** Okay when do you feel a couple should seek divorce in your opinion?

**Interviewee:** when all respect has broken down and there is nothing left anymore. You don’t even …. Even if you don’t have sex with him, that’s alright but if he is constantly calling you damaged and whatever, then yeah. When your esteem is no longer yours. But I see divorce as a last resort and something that is allowed.

**Interviewer:** Do you feel that there is stigma associated with divorce?

**Interviewee:** No not in my circle

**Interviewer:** But you know overall society mein?

**Interviewee:** Eik zamana tha

**Interviewer:** alright, and the fact that your husband already knew you had a problem..

**Interviewee:** No but he says that my mother hid it from him.

**Interviewer:** But you had already told him?

**Interviewee:** Yes I did

**Interviewer:** Did he believe in it? You know some people completely don’t believe that this exists.

**Interviewee:** He used to say that mujhe pagal aurat milgaye.

**Interviewer:** he never thought it was a genuine issue

**Interviewee:** Bhai pata nahi.

**Interviewer:** We just have to wrap up some of the questions. What do you think are some of the essential building blocks for raising a healthy family?

**Interviewee:** *pause* mutual respect. That’s it I guess

**Interviewer:** DO you feel that marital counseling could have helped?

**Interviewee:** Depends on Western and Eastern

**Interviewer:** For you?

**Interviewee:** Western

**Interviewer:** Okay if mental illness is the issue do you think that marital counseling can help?

**Interviewee:** *pause*

**Interviewer:** To give the relatively healthier person more perspective?

**Interviewee:** usne khud apnee dawai chordee.

**Interviewer:** Do you think religion plays a role in mental illness?

**Interviewee:** yes it does. I come from a very orthodox family. Hur cheez mein dozak and what not.

**Interviewer:** Also, do you know about your actual diagnosis?

**Interviewee:** it’s not bipolar, it’s not schizophrenia, it’s a form of psychosis.

**Interviewer:** Okay.

***Interview Ends***
