# Supplementary material for: Exploring effects of severe mental illnesses on marriages: A qualitative study from Karachi, Pakistan
Source: PLOS Glob Public Health. 2025 Dec 23;5(12):e0005652. doi: 10.1371/journal.pgph.0005652 (PMC12725543; doi:10.1371/journal.pgph.0005652)
Supplement: S1 Data — (ZIP) [file pgph.0005652.s001.zip › Transcriptions/Case 2-6 Transcripts/Case 6/C6-1.docx]

**26^th^ May, 2015**

**Case 6**

**Illness:** Schizophrenia

He did not allow the interview to be recorded. Also, the patient was diagnosed before marriage with schizophrenia. And he did not tell his wife. And his family did not tell the wife’s family either. And now he is divorced.

**Interviewer:** When did you get divorced?

**Interviewee:** 6-7 months before

**Interviewer:** How long did your marriage last?

**Interviewee:** 1.5 years

**Interviewer:** When were you first diagnosed?

**Interviewee:** A couple of years back in 2005. And we had changed different doctors, but then we came back to Dr. Hanif Mesiya.

**Interviewer:** Do her parents know about the illness?

**Interviewee:** Yes, her parents found out late on.

**Interviewer:** What happened when you first were diagnosed with your mental illness?

**Interviewee:** I used to throw things and get very angry. I still get angry now but I do not. I used to also beat other people, but this had stopped after I had started taking medications. My anger is now under control

**Interviewer:** Okay and when did your spouse find out about the illness after marriage?

**Interviewee:** Around 4-5 months later. She took me to Peer and Aamil and I told her I have this illness but she did not listen

**Interviewer:** Do people in your family question about the illness?

**Interviewee:** Some people know but not everyone knows

**Interviewer:** Why do you think your wife took a “khulaa” from you?

**Interviewee:** She used to say “*psycho hai, abnormal hai”* Moreover, usko alag ghar mein rehna tha and meiney kaha kay mein apney ami abbu kay baghair nahi rahunga. Unki tabiat theek nahi rehti.

**Interviewer:** Did you want to give the divorce?

**Interviewee:** No. She took it herself through court. I was not giving it at all.

**Interviewer:** What is your reaction to divorce?

**Interviewee:** *Mein sukoon mein agaya hun. Woh mujhe boht tung karteen theen. Mein office mein hota tha tu mujhe ghar mein bulateen theen aur meri ami say kaamo waghera pe lartee bhee boht theen”*

**Interviewer:** Did you two as a couple go out and socialize?

**Interviewee:** Yes we used to go

**Interviewer:** Acha and when she found out about the illness, did your relationship change?

**Interviewee:** *Unka rawaya tabdeel hogaya. Ajeeb ghareeb batein karteen theen. Apnee ami kay ghar chali jaati theen*

**Interviewer:** Who took you to the doctor when you were diagnosed?

**Interviewee:** My mother

**Interviewer:** Did your ex wife ever come to the doctor with you?

**Interviewee:** *nahi shayad eik ya du dafa aye hun*

**Interviewer:** What do you think were the reasons behind taking the divorce?

**Interviewee:** Her elder sister took a divorce from her husband two or three days after getting married, so that’s why she took a divorce. She also said that I am a psycho. *Unko batein nahi mani theen meri tu isliye divorce le li.*

**Interviewer:** Aap ko lagta hai kay beemari ki waja say unhon ne divorce le thee?

**Interviewee:** *Haan yeh bhee eik reason tha*

**Interviewer:** Do you feel you could have saved your marriage?

**Interviewee:** *Mein divorce nahi deh raha tha. Ussay aur zyada kya hosakta hai*

**Interviewer:** Why do you think this illness occur to you?

**Interviewee:** *Pata nahi. Meri ami kehtee hain kay mein chat per boht rehta tha isliye merey uper saya hogaya hai. Mujhe bhee yehi lagta hai. Ub mein chat pe nahi jaata tu mein theek hogaya hun.*

**Interviewer:** Do you feel marital counseling could have helped your marriage?

**Interviewee:** Yes, if the problems would have been discussed, then yes.

**Interviewer:** Why didn’t you tell her about the illness?

**Interviewee:** *Ami ne mana kyat ha kay beemari kay barey mein kisi ko nahi batana”*

***Interview Ends***
