# Supplementary material for: Exploring effects of severe mental illnesses on marriages: A qualitative study from Karachi, Pakistan
Source: PLOS Glob Public Health. 2025 Dec 23;5(12):e0005652. doi: 10.1371/journal.pgph.0005652 (PMC12725543; doi:10.1371/journal.pgph.0005652)
Supplement: S1 Data — (ZIP) [file pgph.0005652.s001.zip › Transcriptions/Case 2-6 Transcripts/Case 6/C6-2.docx]

**Case 6**

**Dr. Hanif’s clinic**

**Psychiatric Illness:** Bipolar Disorder

The husband did not know about the illness before the marriage. However, they are currently separated because her mother brought her home because she was unable to live at her in-law’s. Throughout this time, the subject did not speak at all and it was her mother who provided the details of the marriage and the overall situation. The subject is 8 months pregnant and she had gotten married in February 2015. Her mother had brought her mom from her husband’s house in July 2015. She was first diagnosed when she was 18 years old, during her exams when she was unable to study for the exams and threw loads of tantrums, and used to get angry. Previously, she was engaged to her khala’s son, and the khala broke off the engagement because she saw the subject’s odd behavior. This was taken by the subject quite seriously and she went into a complete depressive phase and had to be taken to the doctor because of her aggressive behavior combined with depression. A couple of months later, another proposal came and she was married off. However, her in-laws did not treat her right and used to taunt her. She had no privacy at all and even her husband did not take her side. (This was the mother’s account). However, she mentions that the husband never hit her and she had to take the decision very properly before deciding to separate. Her husband came to pick her up on Eid but said some nasty things which is when the mother said that she will not send her daughter away. The subject was asked to clean the entire house and do things she had never done so, and even then she was called *sust.* This is why she was unable to live in the house. The mother kept on repeating throughout the interview that this should not be leaked anywhere because no one knows about the illness and also the separation in the extended family.

It was Nazia’s decision to separate because she said that *mein agay zindagi nahi guzar saktee’* and *ubh koi faida nahi hai, yeh shaks dil say utar gaya hai* because her mobile was also taken away from her, and she was not allowed to even go to her mother’s house and her nani’s house. They did go for their honeymoon but overall, they did not socialize. When asked as to why they did not tell about the illness before the marriage, the mother said that she was completely fine when she got married. She got unstable because of the behavior of her in-laws towards her. The husband used to take care of her on and off but never supported her completely. He also did not buy things for her and used to say *tumhara boht kharcha hai*. Even to this day, her in-laws do not know about the psychiatric illness nor did her husband know (however, we can safely assume that they were bothered by the symptoms that manifested which means that the illness did indirectly cause a negative effect on the marriage). Nazia also mentions that she was threatened by her husband that he would lock her in the room. She has suicidal ideation as well and at baqra eid, she kept on saying *mujhe kaat dein.*

When asked whether the husband asked about the unborn child, she said that no he didn’t and in fact while initially he was quite happy when the urine test came out, he later on asked “*hai ya giradiya’* indicating that she might have aborted the child.

**Note:**

A whole lot of questions were missed because the mother and the subject both were obviously distressed which means that it would have been unethical to press for more details. The mother constantly wanted the reassurance that she does not want anyone to find out about the illness and that the details should not be disclosed to anyone regarding the interview.
