# Supplementary material for: Exploring effects of severe mental illnesses on marriages: A qualitative study from Karachi, Pakistan
Source: PLOS Glob Public Health. 2025 Dec 23;5(12):e0005652. doi: 10.1371/journal.pgph.0005652 (PMC12725543; doi:10.1371/journal.pgph.0005652)
Supplement: Attachment — Submitted filename: Response to Reviewers.docx [file pgph.0005652.s004.docx]

**Response to Reviewers**

We would like to thank the reviewers for their extensive and insightful comments on our study.

**Reviewer 1**

| **Sr.no** | **Comment** | **Response and Changes** |
| --- | --- | --- |
| 1 | The study title is okay. | Thank you. |
| 2 | The abstract needs minor revision. The abstract, which highlights the cultural dynamics of marriage and mental illness in Pakistan, is interesting and pertinent to the context. It is too detailed, though; readability and scholarly impact would be improved by more concisely expressing the research gaps, methodological support, and implications. | The abstract has been revised in light of the recommendations. |
| 3 | The introduction also needs minor revision. With a strong emphasis on empirical data and pertinent South Asian context, the introduction offers a thorough summary of the connection between marriage and mental health. Its readability is hampered by its excessive length and some redundancy, though. Although there are many citations, some of the statements are still general and would be better served by critical synthesis as opposed to descriptive listing. Although the justification for concentrating on Pakistan is given, it could be strengthened by clearly contrasting it with previously published works of Indian and Western literature. The justification would be strengthened if the research gaps were more clearly stated. Improve the study rationale by including g the following studies in the introduction:  Tiwari, G. K., Singh, A., Choudhary, A., Shukla, A., Macorya, A. K., Pandey, A., & Singh, A. K. (2025). Forgiveness in Later Life: Attributes and Consequences for Older Adults in Indian Families. Marriage & Family Review, 61(7), 710–736. https://doi.org/10.1080/01494929.2025.2484381  Tiwari, G. K., Tiwari, R. P., Pandey, R., Ray, B., Dwivedi, A., Sharma, D. N., Singh, P., Tiwari, A. K., & Singh, A. K. (2024). Perceived Life Outcomes of Indian Children During the Early Phase of the COVID-19 Lockdown: The Protective Roles of Joint and Nuclear Families. Journal of Research and Health, 14(1), 43–54. https://doi.org/10.32598/JRH.14.1.1992.4 | We have revised the introduction to include specific studies from India and Pakistan. (see lines 74-76, line 106) These studies have also been used to highlight the gap in literature particularly from a qualitative methods perspective. Please see the rationale towards the end of the introduction (lines 108-110)  We also believe that while the citations provided by the reviewer are helpful in building knowledge base, these do not fit well into our Introduction. |
| 4 | The methods section also needs improvements. The methods section is thorough and shows a solid theoretical framework, careful application of qualitative design, and obvious ethical considerations. But the sampling procedure seems unduly complicated, and changing the criteria in the middle of the study might cause issues with transparency and consistency. For many participants, taking notes rather than recording audio could jeopardize the reliability and richness of the data.  Although Braun and Clarke's framework was used, credibility would be increased with more information on inter-coder dependability and dispute resolution procedures. Although there is methodological rigor overall, it could use more explanation and clarity. | We appreciate the reviewer’s careful review of our methods section. With respect to the sampling procedure, since we decided on theoretical sampling (which does not require a prior selection of the sample), we believe that adding more cases during data collection actually adds more depth to our findings.  This has been mentioned in Lines 151-152. We have added an additional reference to support our assertion.  We have also added dispute resolution procedures (Line 219-220) |
| 5 | The results section also needs revision. Although the results section provides a thorough and in-depth explanation of the sociodemographic traits of the participants as well as thematic findings, it is excessively descriptive and occasionally repetitious, which makes interpretation challenging. Large tables and lengthy narratives run the risk of overwhelming the reader. The section would benefit from a more succinct synthesis of patterns rather than a long list of cases, even though the use of participant quotes increases credibility. Stronger analytical depth and improved clarity could result from a more thorough integration of demographic data with thematic insights. It is also advised to make a clearer connection between the findings and the goals of the study | After making the relevant changes in the Introduction along with some modifications in the findings section, we have highlighted a clearer connection between the findings and goals of the study.  However, we believe that retaining quotes and providing extra depth allows for rigor and trustworthiness in the study, and as the reviewer themselves state, increases credibility. We would like to retain these especially since we are well within the word limit. |
| 6 | The discussion section also needs revision. The discussion section is thorough, organized, and provides profound cultural insights into how marriage dynamics and severe mental illness interact in Pakistan. Sometimes, though, it runs the risk of restating descriptive findings without critically integrating them with more comprehensive theoretical frameworks. Although the rationale for utilizing data that is ten years old is clarified, contextual validity remains a concern. Despite their richness, some themes might benefit from tighter synthesis to prevent repetition. Scholarly rigor would be improved by more interaction with cross-cultural literature and more precise explanation of the implications for policy and practice. | We have made changes to the discussion section by adding more cross-cultural literature (Lines 655-656, line 673).  We have added a separate heading to explain implications for policy and practice to highlight this aspect. |
| 7 | The references are ok. Please correct it by following the journal’s guidelines. The tables and figures should be prepared following standard guidelines. | We have provided linked references in Vancouver style for easy readability. The journal accepts Vancouver styled references. |

**Reviewer 2**

| **Sr.no** | **Comment** | **Response and Changes** |
| --- | --- | --- |
| 1 | The study's aim and findings address important dimension of how mental illnesses influence marriages in Pakistan given the nature of patriarchal society and the pervasive stigma around mental illnesses, unmarried status and divorce. Although the use of decade old data is concerning, the authors have addressed and justified its relevance describing the lack of existing literature addressing this dimension, the continued stigma, and the lack of contextual changes with respect to norms and laws around marriages in Pakistan. However, given other kinds of rapid advancements including dissemination of information through digital platforms with implications for mental health awareness and the current economic problems in Pakistan, the use of a decade old dataset needs to be emphasised and acknowledged for where it might indicate gaps in creating knowledge representative of the present-day marital lives in Pakistan. Would also strongly advice revising the use of the term "broken marriage(s)" or marital outcome as intact or broken to move away from stigmatising separation or divorce. Instead, terms like marital status as separated or divorced can be used. | Thank you for appreciating the importance of our study findings.  With respect to use of decade old findings, we have added a section on limitations which addresses this specific concern. (See Lines 728-734)  We have revised the use of the term “broken” marriages throughout our manuscript. Thank you for pointing this out. |
| 2 | Title: Since the entire manuscript uses the term 'severe mental illnesses', hence, would suggest keeping it consistent and changing 'severe mental disorders' in title to 'severe mental illnesses'. | Thank you for pointing this out. We have made this change. |
| 3 | Introduction: Provides a concise brief introduction. Line 54 mentions 'studies' but only cites one study (3). Line 57-59 mentions 'one study' but cites two. Line 60 mentions 'existing literature' but cites the previously cited study only. Would suggest giving a richer introduction citing more existing studies especially from South Asia, not just repeat the same citation (2). If any statistics are available or qualitative research about the quality of life or problems faced in daily living by people with SMIs in Pakistan are available, would suggest including those here. in Line 63, would suggest revising the use of "disturbance in one member (such as mental illness)" since it situates and limits the mental illness within the individual ignoring its complex etiology. The use of word 'disturbance' can be replaced by the use of a more empathetic term like suffering. | Thank you for pointing out these inconsistencies. We have addressed these.  As we have mentioned in our rationale, there are no studies using qualitative methods in Pakistan. However, we have added a quantitative survey that looked at quality of life among those with severe mental illnesses. Other studies have also been added.  In addition, we have replaced the word ‘disturbance’ with suffering. See Line 68-69. |
| 4 | Participants: The section mentions that "This study included individuals diagnosed with severe mental illnesses in the case of divorce or separation, and spouses of those diagnosed with these conditions in the case of an intact marriage." However, Case 4 in Table 1 includes spouses of patients in the case of "broken" marriage. | Case 4 referred to those in an intact marriage. We have made the change in Table 1. |
| 5 | Setting: Check grammar in line 133 ("initial patients.-") | We have amended the phrase so that it reads better grammatically. |
| 6 | Data Collection Procedure: Line 176 needs clarification about how data saturation was defined for this study. | The sentence about data saturation has been elaborated. We hope that the addition will now make it clear. |
| 7 | Table 2: under the column titled 'Marriage Duration", it needs to be clarified whether the duration is specified in months or years. The unit needs to be uniform across all rows. | We would prefer keeping the units in months and years as has been specified in the table. |
| 8 | Table 3: The use of the word 'universal' in Table 3 and in Results section to determine the trustworthiness of themes, seems problematic since qualitative research does not claim to produce universal findings anyways. Using the word 'consistency' for themes may be more appropriate when referring to the consistency of the themes within the dataset for the present study.  Although confidence levels are not required in qualitative research especially when ethical rigour and practices like reflexivity, use of audit trails, inter-coder relaiability have already been mentioned. CerQual is not necessary here since it is not a synthesis of evidence from different qualitative research. But it may be acceptable given the authors' justification for it when presenting each theme under the Results section. | We have removed the word universal in Table 3 and in the Results section.  Thank you for the comments on CerQual—we believe that it would be helpful in demonstrating the consistency of themes across different cases. |
| 9 | Rigor and Trustworthiness: I appreciate the authors' attempts to transparently detail the ethical considerations and practice of rigour across the manuscript. Please check punctuations in line 225. | Thank you for appreciating this section. We have checked the punctuation. Please see Lines 236-240. |
| 10 | Results: In Line 494, would suggest to add "multiple marriages *for men* are allowed in Islam". In Line 620, specify the full form of 'HIC' (high income countries) since it is used here for the first time in the manuscript. | Added.  We have replaced HIC with Global North. |
| 11 | Discussion: Discussion section can be enriched with discussion of the present findings in light of more existing literature from South Asian countries or contrast with high-income counties or with findings from other nations with similar settings. Quantitative data from national-level surveys about family structure, age-wise and gender-wise distribution of SMIs, marital status of people with SMIs, etc. can also be discussed with reference to the present findings. Line 640 requires citation(s) of the "existing corpus of literature" mentioned.  Line 669 and 678- The phrases "children anchor marital stability" and "hold marriages together" can sound reductive and problematic when phrased as declarative statements.  Line 699- use of words like 'in fact' is problematic for something written without substantiating it with appropriate citations. | We have added more cross-cultural literature ((Lines 655-656, line 673). There are no nation-wide surveys in Pakistan which have age-wise and gender-wise distribution of SMIs with reference to the present findings. There are isolated surveys that present these findings however adding many references risks overwhelming the reader, as has been pointed by the other reviewer.  We have added citations for Line 640 (now Line 683). The phrases that you refer to have been altered slightly to ensure that they no longer appear reductive.  We have added citations on Line 699 (now Line 725) to provide evidence for our statement. |
| 12 | The last paragraphs mentioning the implications of the research should be put under a separate section for implications and future directions for use to separate them from the discussion of findings from the data.  I would suggest including a separate section on limitations to highlight the caution in using decade old data and data sourced from only one tertiary level hospital. The number of spouses (35) and participants (10; people with SMIs) interviewed also seems to be disproportionate and hence might highlight more caregiving burdens in marriages compared to the marital experiences of people with SMIs. | We have taken these suggestions into account, and have added a paragraph on study implications and study limitations. |
